# Predicting the total PAHs concentrations in sediments from selected congeners using a multiple linear relationship

**DOI:** 10.1038/s41598-022-07312-2

**Published:** 2022-02-28

**Authors:** Weiwei Wang, Huaping Xu, Xiaolei Qu, Kun Yang, Daohui Lin

**Affiliations:** 1 Department of Environmental Science, Zhejiang University, Hangzhou, 310058 China; 2 Key Laboratory of Environmental Pollution and Ecological Health of Ministry of Education, Hangzhou, 310058 China; 3 Zhejiang Provincial Key Laboratory of Organic Pollution Process and Control, Hangzhou, 310058 China; 4 Mathematics Teaching and Research Section, College of Pharmaceutical Sciences, Zhejiang Chinese Medical University, Hangzhou, 310053 China; 5 State Key Laboratory of Pollution Control and Resource Reuse, School of the Environment, Nanjing University, Jiangsu, 210023 China; 6 Zhejiang University-Hangzhou Global Scientific and Technological Innovation Center, Hangzhou, 311200 China

**Keywords:** Environmental sciences, Environmental chemistry, Environmental monitoring

## Abstract

In this study, we observed that four congeners, including naphthalene (Nap), acenaphthylene (Acy), phenanthrene (Phe), and benz(a)anthracene (BaA), are the characteristic congeners for predicting the emission and the sediment concentrations of polycyclic aromatic hydrocarbons (PAHs). A novel multiple relationship of the total PAHs concentrations (C_∑PAHs_) in sediments with the concentrations of four congeners was established (*p* < 0.01, R^2^ = 0.95) using published data over the past 30 years. Moreover, the multiple linear relationship of the total PAHs emission factors with the emission factors of four congeners was also established (*p* < 0.01, R^2^ = 0.99). Interestingly, the ratio of multicomponents coefficient from the multiple linear relationship in sediments to that from the multiple linear relationship in emission sources correlated positively with octanol–water partition coefficient (log*K*_ow_) (*p* < 0.01, R^2^ = 0.88) of the four PAHs congeners. Therefore, a novel model was established to predict C_ΣPAHs_ in sediments using the emissions and log*K*_ow_ of the four characteristic PAHs congeners. The percent sample deviation between calculated C_∑PAHs_ and their observed values was 54%, suggesting the established model can accurately predict C_ΣPAHs_ in sediments.

## Introduction

Polycyclic aromatic hydrocarbons (PAHs) are a group of persistent organic contaminants that originate from the incomplete combustion of organic matter (such as biomass and coal) and non-combustion emissions of petrogenic processes^1–3^. United States Environmental Protection Agency (USEPA) and European Union (EU) have identified sixteen PAHs (Table S1) as priority pollutants due to their toxicity and risks to human health and the environment^4–7^. For example, PAHs in sediments can pose detrimental effects on benthic organisms and pelagic organisms^8,9^. Therefore, PAHs concentrations in sediments have been widely investigated in the past decades for assessing their risks^10–18^. The common method for the quantification of PAHs in sediments is chromatography, including gas chromatography or liquid chromatography, coupled with mass spectrometry^19–21^. However, the whole process of chromatographic analysis for PAHs in sediments is tedious and cost-consuming. Moreover, this method may be potentially damaging for the environment because the analysis typically requires a pre-concentration procedure, which may use large volumes of organic solvents for extraction and clean-up^22–24^. For example, the frequently used organic solvents, dichloromethane^19–21^, can damage human nervous system and even the functions of liver and kidney through skin mucosa and nasal breathing^25,26^. Therefore, it is necessary to establish correlations that can be applied to predict concentrations of PAHs in sediments for cutting costs and saving time in laboratory analysis^27,28^.

**Figure 1:**
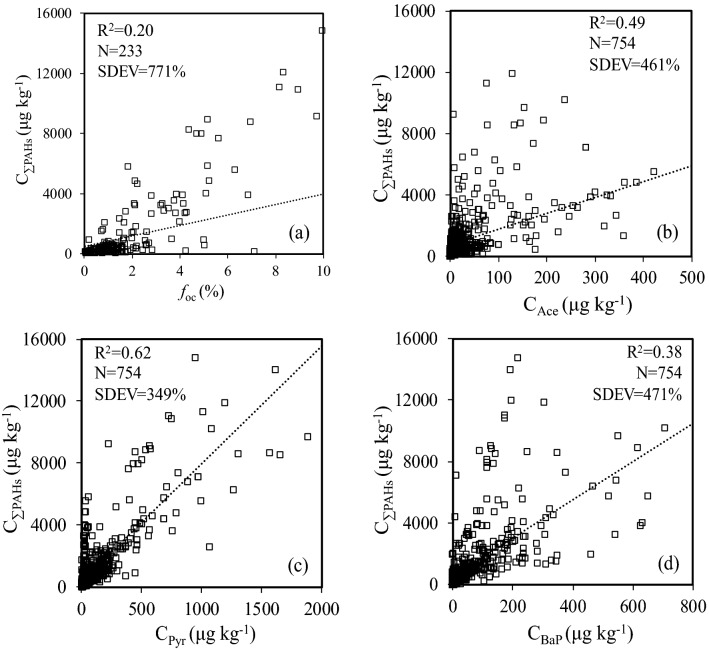
Linear relationships of C_ƩPAHs_ with *f*_oc_ (**a**), C_(Ace)_ (**b**), C_(Pyr)_ (**c**) or C_(BaP)_ (**d**). Dashed lines in the plots are linear regressions.

In recent literatures, significantly positive correlations between the total concentration of PAHs (C_ƩPAHs_) and the content of total organic carbon (*f*_*oc*_) in sediments were established to predict C_ƩPAHs_^17,29–31^, built on the premise that the distribution of PAHs between sediments and water is largely depended on the partitioning of PAHs into sediment organic matter^32^. However, our preliminary work indicated that C_ƩPAHs_ predicted using *f*_*oc*_ (Eq. 1) does not hold true for additional C_ƩPAHs_ and *f*_*oc*_ data of 233 global sediment samples (Fig. 1a, Table S2), presented by the low determination coefficients (R^2^ = 0.20) and the great percent sample deviation (SDEV = 771%). One possible reason for the insignificant correlation between C_ƩPAHs_ and *f*_*oc*_ when more data was introduced is that the difference in emissions of PAHs in various regions is ignored. Another possible reason is that the dependence of PAHs partitioning in sediment organic matter and their polarity is ignored^4,32,33^. Furthermore, nonlinear sorption of PAHs on sediments organic matter is also ignored^34,35^.

Intrinsic quantitative relationships between C_ƩPAHs_ and the concentrations of single PAHs congener were also established to predict sediment C_ƩPAHs_ in previous studies^36–39^. For example, the concentration of benzo(a)pyrene (C_BaP_)^36,37^, pyrene (C_Pyr_)^38^ or acenaphthene (C_Ace_)^39^ was suggested to predict C_ƩPAHs_ (Table S3). However, when relationships of C_ƩPAHs_ with C_BaP_ (Eq. 2), C_Pyr_ (Eq. 3) and C_Ace_ (Eq. 4) were established using the additional sediment concentration data of PAHs from China (Table S4), it was found that the relationships were less significant with greater deviation (Eqs. 2–4). For example, R^2^ of the linear relationship between C_ƩPAHs_ and C_Ace_ (Eq. 4) reduced from 0.82^39^ to 0.49 (Eq. 4) with SDEV increased from 27%^39^ (Table S3) to 461% (Eq. 4), when sediment sample numbers (N) increased from 10^39^ (Table S3) to 754 (Eq. 4), respectively. A possible reason for these relationships (Eqs. 2–4) predicted with less accuracy is that the difference in emission factors (EFs) of PAHs congeners for various sources is ignored. For example, at the equivalent emission factors of the total PAHs (EF_ƩPAHs_), which is 4.39 g t^−1^ for iron sintering and 4.51 g t^−1^ for gasoline combustion (Table 1), EF of Ace (Table 1) from iron sintering is 0.079 g t^−1^, about 2 orders of magnitude larger than that 0.00046 g t^−1^ of gasoline combustion^40,41^. Therefore, the concentrations of single PAHs congener cannot be used to accurately predict C_ƩPAHs_ in sediments on a large scale. The characteristic PAHs congeners in emission sources and in sediments should be explored to develop an accurate model for predicting C_ƩPAHs_ in sediments.

1 $$\begin{aligned} {\text{C}}_{{\Sigma {\text{PAHs}}}} = & { 351}.{75}\left( { \pm {39}.{67}} \right) \, \times f_{oc} + { 455}.{13}\left( { \pm {132}.{48}} \right) \\ & \left( {{\text{R}}^{{2}} = \, 0.{2}0,p = \, 0.{13},{\text{ N }} = { 233},{\text{ SDEV }} = { 771}\% } \right) \\ \end{aligned}$$

2 $$\begin{aligned} {\text{C}}_{{\Sigma {\text{PAHs}}}} = & { 12}.{5}0\left( { \pm 0.{59}} \right) \, \times {\text{ C}}_{{{\text{BaP}}}} + { 512}.{96}\left( { \pm {76}.{53}} \right) \\ & \left( {{\text{R}}^{{2}} = \, 0.{38},p < \, 0.0{1},{\text{ N }} = { 754},{\text{ SDEV }} = { 471}\% } \right) \\ \end{aligned}$$

3 $$\begin{aligned} {\text{C}}_{{\Sigma {\text{PAHs}}}} = & { 7}.{16}\left( { \pm 0.{15}} \right) \, \times {\text{ C}}_{{{\text{pyr}}}} + { 34}0.{26}\left( { \pm {45}.{2}0} \right) \\ & \left( {{\text{R}}^{{2}} = \, 0.{62},p < \, 0.0{1},{\text{ N }} = { 754},{\text{ SDEV }} = { 349}\% } \right) \\ \end{aligned}$$

4 $$\begin{aligned} {\text{C}}_{{\Sigma {\text{PAHs}}}} = & { 1}0.{48}\left( { \pm 0.{41}} \right) \, \times {\text{ C}}_{{{\text{Ace}}}} + { 765}.{57}\left( { \pm {71}.{31}} \right) \\ & \left( {{\text{R}}^{{2}} = \, 0.{49},p < \, 0.0{1},{\text{ N }} = { 754},{\text{ SDEV }} = { 464}\% } \right) \\ \end{aligned}$$

**Table 1:** Coefficient of determination (R^2^) and significance (p) of emission factors (EFs) of individual PAHs congeners with the total EFs of PAHs congeners in four subgroups for various emission sources.

EFs	PAHs	R^2^	*p* value
Ʃ 1st subgroup	Nap	1.00	< 0.01
Ʃ 2nd subgroup	Acy	1.00	< 0.01
Ʃ 3rd subgroup	Ace	0.87	< 0.01
	Flo	0.97	< 0.01
	Phe	0.98	< 0.01
	Ant	0.96	< 0.01
	Flu	0.97	< 0.01
	Pyr	0.91	< 0.01
Ʃ 4th subgroup	BaA	0.98	< 0.01
	Chr	0.97	< 0.01
	BbF	0.94	< 0.01
	BkF	0.92	< 0.01
	BaP	0.94	< 0.01
	IcdP	0.88	< 0.01
	BghiP	0.87	< 0.01
	DahA	0.77	< 0.01

In this study, a multiple linear relationship between EF_ΣPAHs_ and the EFs of characteristic congeners was established by identifying characteristic PAHs congeners in emission sources. Moreover, another multiple linear relationship between C_ΣPAHs_ and the concentrations of characteristic congeners in sediments was established by identifying characteristic PAHs congeners in sediments. Finally, an accurate model for predicting C_ΣPAHs_ in sediments was established by exploring the correlation between the sediment concentrations and the emissions of characteristic PAHs congeners. The established model can cut costs and save time in PAHs analysis for risk assessing of PAHs in the sediment environment.

## Result and discussion

### Characteristic congeners of PAHs in emission

**Figure 2:**
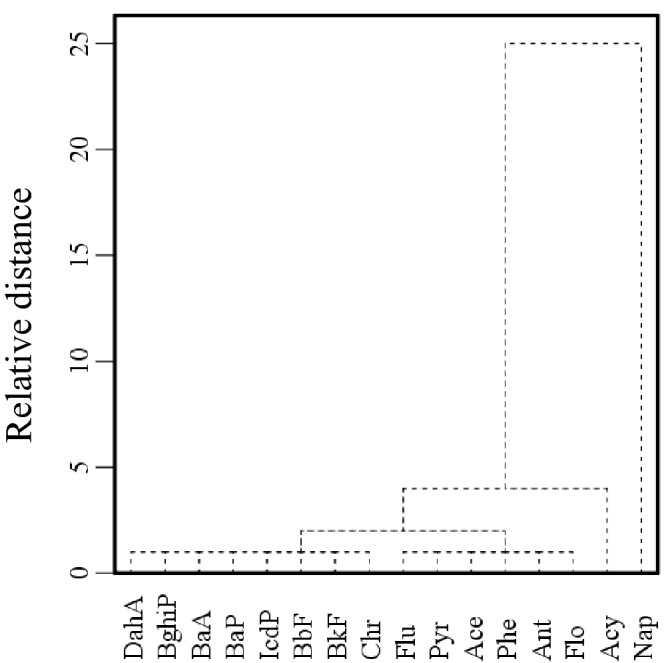
Hierarchical clustering analysis dendrograms of emission factors of sixteen PAHs in emission sources using average linkage clustering between clusters.

**Figure 3:**
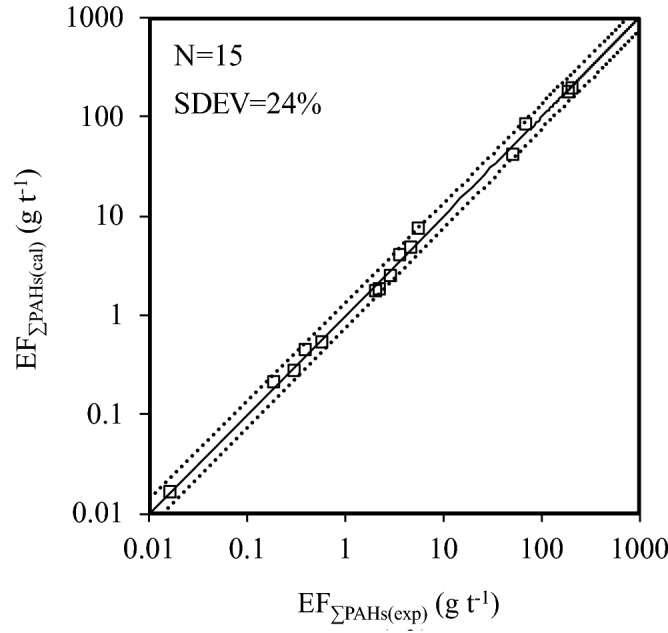
Fitted EF_ƩPAHs(cal)_ versus EF_ƩPAHs(exp)_ from nine PAHs emission sources. The y = x line (solid line) indicates a 1:1 relationship between EF_ƩPAHs(cal)_ and EF_ƩPAHs(exp)_. Dashed lines in the plot indicate the SDEV values from the reference line.

Hierarchical clustering analysis (HCA) and classifications for relative similarities^42,43^ of PAHs emission factors (Table S5) show that sixteen PAHs can be divided into four groups (Table 1, Fig. 2). The first subgroup is Nap (Table 1, Fig. 2) because of its highest EFs in most emission sources (Table S5). Acy is the second subgroup (Table 1, Fig. 2) with significant lower EFs than that of Nap but higher than other PAHs congeners in most emission sources (Table S5). The third subgroup comprises four 3-ring PAHs (Ace, Flo, Phe and Ant) and two 4-ring PAHs (Pyr and Flu) (Fig. 2, Table 1). In this subgroup, the EFs of Phe (EF_Phe_) has the best linear correlation with the total EFs of this subgroup, showing the maximum R^2^ of 0.98 (N = 15, *p* < 0.01) (Table 1). Thus, the total EFs of the third subgroup can be expressed by EF_Phe_ with the largest degree of accuracy^43,44^. The last subgroup is composed of the other eight PAHs congeners (Fig. 2, Table 1), including 4-ring PAHs (BaA and Chr) and 5, 6-ring PAHs (BbF, BkF, BaP, IcdP, DahA and BghiP). The EFs of BaA (EF_BaA_) correlates best with the total EFs of the eight PAHs in this subgroup with the maximum R^2^ of 0.98 (N = 15, *p* < 0.01) (Table 1). This indicates that the total EFs of the last subgroup can be presented by EF_BaA_. Moreover, the total EFs of sixteen PAHs (EF_ƩPAHs_) are well related with EF_Nap_, EF_Acy_, EF_Phe_, and EF_BaA_ in a multilinear relationship (Eq. 5 and Fig. 3), having R^2^ of 0.99, high F values of 5257, and low SDEV of 24%. Therefore, Nap, Acy, Phe, and BaA can be employed as characteristic congeners of sixteen PAHs in emission sources (Fig. 3).

5 $$\begin{aligned} {\text{EF}}_{{\sum {\text{PAHs}}}} = & - 0.{45}\left( { \pm 0.{46}} \right) \, + \, 0.{98}\left( { \pm 0.{12}} \right) \, \times {\text{ EF}}_{{{\text{Nap}}}} + { 1}.{12}\left( { \pm 0.{11}} \right) \, \times {\text{ EF}}_{{{\text{Acy}}}} \\ & + { 2}.{82}\left( { \pm 0.{23}} \right) \, \times {\text{ EF}}_{{{\text{Phe}}}} + { 4}.0{4}\left( { \pm 0.{47}} \right) \, \times {\text{ EF}}_{{{\text{BaA}}}} \\ & \left( {{\text{N}} = {15},{\text{ R}}^{{2}} = 0.{99},{\text{ F}} = {5257},{\text{ SDEV}} = {24}\% } \right) \\ \end{aligned}$$

### Characteristic congeners of PAHs in sediments

**Figure 4:**
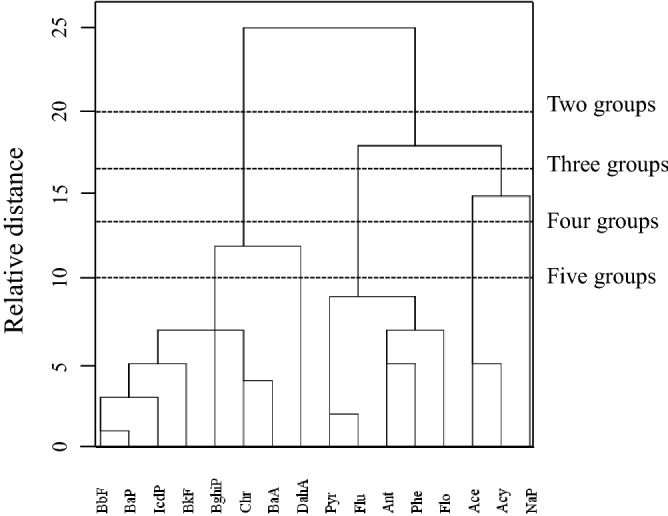
Hierarchical clustering analysis dendrograms of sixteen PAHs concentrations in sediments using average linkage clustering between clusters.

**Figure 5:**
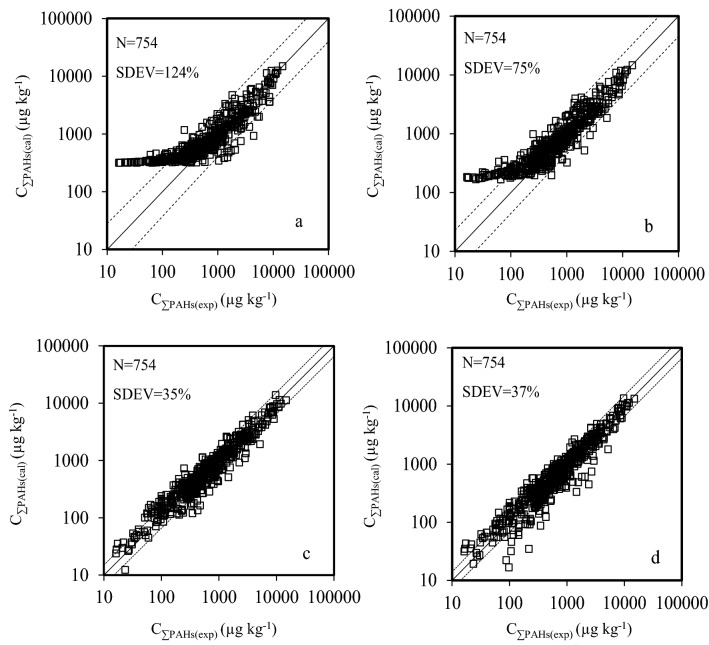
Fitted C_ƩPAHs(cal)_ values from two groups (**a**), three groups (**b**), four groups (**c**) and five groups (**d**) versus C_ƩPAHs(exp)_ values in sediments sampled in China. The y = x line (solid lines) indicates a 1:1 relationship between C_ƩPAHs(cal)_ and C_ƩPAHs(exp)_. Dashed lines in the plots indicate the SDEV values from the reference lines.

The HCA dendrogram for correlations of sixteen PAHs in sediments of China is shown in Fig. 4. When PAHs are classified into two groups, the first subgroup is composed of 2, 3-rings PAHs and two 4-rings PAHs (Table 2). In this subgroup, the concentration of Phe (C_Phe_) correlates best with the total concentration of the eight congeners with the maximum R^2^ of 0.83 (N = 754, *p* < 0.01) (Table 2). The other two 4-rings PAHs and 5, 6-rings PAHs are divided into the second subgroup (Table 2). In the second subgroup, the maximum value of R^2^ was found between the concentration of BaA (C_BaA_) and the total concentration of the eight congeners (R^2^ = 0.79, N = 754, *p* < 0.01) (Table 2). Thus, the total concentration of PAHs in two subgroups can be expressed by C_Phe_ and C_BaA_ with the largest degree of accuracy, respectively^43,44^. Relationship of C_Phe_ and C_BaA_ with C_ƩPAHs_ was established in Eq. (6). Similarly, when 16 PAHs were classified into three groups, the concentration of Nap (C_Nap_), Phe (C_Phe_), and BaA (C_BaA_) correlate best with the total concentrations of congeners in corresponding subgroup (Table 2). Relationship of C_Nap_, C_Phe_, and C_BaA_ with C_ƩPAHs_ was established in Eq. (7). When sixteen PAHs were classified into four groups, C_Nap_, C_Acy_, C_Phe_, and C_BaA_ correlate best with the total concentrations of congeners in corresponding subgroup (Table 2). Relationship of C_Nap_, C_Acy_, C_Phe_, and C_BaA_ with C_ƩPAHs_ was established in Eq. (8). When sixteen PAHs were classified into five groups, C_Nap_, C_Acy_, C_Phe_, C_BaA_, and the concentration of DahA (C_DahA_) correlate best with the total concentrations of congeners in corresponding subgroup (Table 2). Relationship of C_Nap_, C_Acy_, C_Phe_, C_BaA_ and C_DahA_ with C_ƩPAHs_ was established in Eq. (9). Correlations between the calculated C_ƩPAHs_ (C_ƩPAHs(cal)_) and the experimental value (C_ƩPAHs(exp)_) are presented in Fig. 5. SDEV values between C_ƩPAHs(cal)_ and C_ƩPAHs(exp)_ in Fig. 5a–d were 124%, 75%, 35% and 37%, respectively. SDEV values decreased significantly from Fig. 5a–c, while almost remained constant from Fig. 5c, d. Intercepts in equations presented the same tendency to SDEV value (Fig. 5). C_ƩPAHs_ can’t be accurately predicted using two (Fig. 5a) or three congeners (Fig. 5b), especially when C_ƩPAHs_ in sediments lower than the intercepts in Eqs. (6–7). Four (Fig. 5c) or five congeners (Fig. 5d) can accurately predict C_ƩPAHs_. However, more work needs to be done to complete the prediction of five congeners than four congeners. In summary, C_ƩPAHs_ can be well predicted from the concentration of Nap, Acy, Phe, and BaA using the linear relationship of Eq. (8) (Fig. 5c).

6 $$\begin{aligned} {\text{C}}_{{\Sigma {\text{PAHs}}}} = & { 245}.{62}\left( { \pm {28}.{67}} \right) \, + { 3}.{92}\left( { \pm 0.{1}0} \right) \, \times {\text{ C}}_{{{\text{Phe}}}} + { 4}.{71}\left( { \pm 0.{2}0} \right) \, \times {\text{ C}}_{{{\text{BaA}}}} \\ & \left( {{\text{N}} = {754},{\text{ R}}^{{2}} = 0.{83},{\text{ F}} = {219}0,{\text{ SDEV}} = {124}\% } \right) \\ \end{aligned}$$

7 $$\begin{aligned} {\text{C}}_{{\Sigma {\text{PAHs}}}} = & { 124}.{27}\left( { \pm {25}.{88}} \right) \, + { 1}.{81}\left( { \pm 0.{11}} \right) \, \times {\text{ C}}_{{{\text{Nap}}}} \\ & + { 3}.{43}\left( { \pm 0.{14}} \right) \, \times {\text{ C}}_{{{\text{Phe}}}} + { 4}.{67}\left( { \pm 0.{23}} \right) \, \times {\text{ C}}_{{{\text{BaA}}}} \\ & \left( {{\text{N}} = {754},{\text{ R}}^{{2}} = 0.{89},{\text{ F}} = {2348},{\text{ SDEV}} = {75}\% } \right) \\ \end{aligned}$$

8 $$\begin{aligned} {\text{C}}_{{\Sigma {\text{PAHs}}}} = & { 22}.{62}\left( { \pm {11}.{78}} \right) \, + \, 0.{84}\left( { \pm 0.0{7}} \right) \, \times {\text{ C}}_{{{\text{Nap}}}} + { 1}.{23}\left( { \pm 0.{13}} \right) \, \times {\text{ C}}_{{{\text{Acy}}}} \\ & + { 3}.{78}\left( { \pm 0.{15}} \right) \, \times {\text{ C}}_{{{\text{Phe}}}} + { 5}.{97}\left( { \pm 0.{22}} \right) \, \times {\text{ C}}_{{{\text{BaA}}}} \\ & \left( {{\text{N}} = {754},{\text{ R}}^{{2}} = 0.{95},{\text{ F}} = {274}0,{\text{ SDEV}} = {35}\% } \right) \\ \end{aligned}$$

9 $$\begin{aligned} {\text{C}}_{{\Sigma {\text{PAHs}}}} = & { 33}.{34}\left( { \pm {19}.{89}} \right) \, + \, 0.{76}\left( { \pm 0.{14}} \right) \, \times {\text{ C}}_{{{\text{Nap}}}} + { 1}.{31}\left( { \pm 0.{32}} \right) \, \times {\text{ C}}_{{{\text{Acy}}}} \\ & + { 3}.{43}\left( { \pm 0.{12}} \right) \, \times {\text{ C}}_{{{\text{Phe}}}} + { 4}.{54}\left( { \pm 0.{3}0} \right) \, \times {\text{ C}}_{{{\text{BaA}}}} + { 1}.{5}0\left( { \pm 0.{51}} \right) \, \times {\text{ C}}_{{{\text{DahA}}}} \\ & \left( {{\text{N}} = {754},{\text{ R}}^{{2}} = 0.{96},{\text{ F}} = {2557},{\text{ SDEV}} = {37}\% } \right) \\ \end{aligned}$$

**Table 2:** Correlation coefficient (R^2^) between the concentration of single congener and total congeners in each subgroup when sixteen PAHs congeners were divided into two-five groups.

Two groups	PAHs	R^2^	Three groups	PAHs	R^2^	Four groups	PAHs	R^2^	Five groups	PAHs	R^2^
Ʃ 1st subgroup	Nap	0.45	Ʃ 1st subgroup	Nap	0.71	Ʃ 1st subgroup	Nap	1.00	Ʃ 1st subgroup	Nap	1.00
	Acy	0.58		Acy	0.62	Ʃ 2nd subgroup	Acy	0.62	Ʃ 2nd subgroup	Acy	0.62
	Ace	0.26		Ace	0.49		Ace	0.53		Ace	0.53
	Flo	0.73	Ʃ 2nd subgroup	Flo	0.69	Ʃ 3rd subgroup	Flo	0.69	Ʃ 3rd subgroup	Flo	0.69
	Phe	0.83		Phe	0.88		Phe	0.88		Phe	0.88
	Ant	0.72		Ant	0.66		Ant	0.66		Ant	0.66
	Flu	0.67		Flu	0.75		Flu	0.75		Flu	0.75
	Pyr	0.66		Pyr	0.78		Pyr	0.78		Pyr	0.78
Ʃ 2nd subgroup	BaA	0.79	Ʃ 3rd subgroup	BaA	0.79	Ʃ 4th subgroup	BaA	0.79	Ʃ 4th subgroup	BaA	0.77
	Chr	0.75		Chr	0.75		Chr	0.75		Chr	0.76
	BbF	0.66		BbF	0.66		BbF	0.66		BbF	0.68
	BkF	0.75		BkF	0.75		BkF	0.75		BkF	0.74
	BaP	0.64		BaP	0.64		BaP	0.64		BaP	0.64
	IcdP	0.57		IcdP	0.57		IcdP	0.57		IcdP	0.58
	BghiP	0.74		BghiP	0.74		BghiP	0.74		BghiP	0.73
	DahA	0.59		DahA	0.59		DahA	0.59	Ʃ 5th Subgroup	DahA	1.00

### Prediction of total PAHs concentrations in sediments with characteristic PAHs congeners

**Figure 6:**
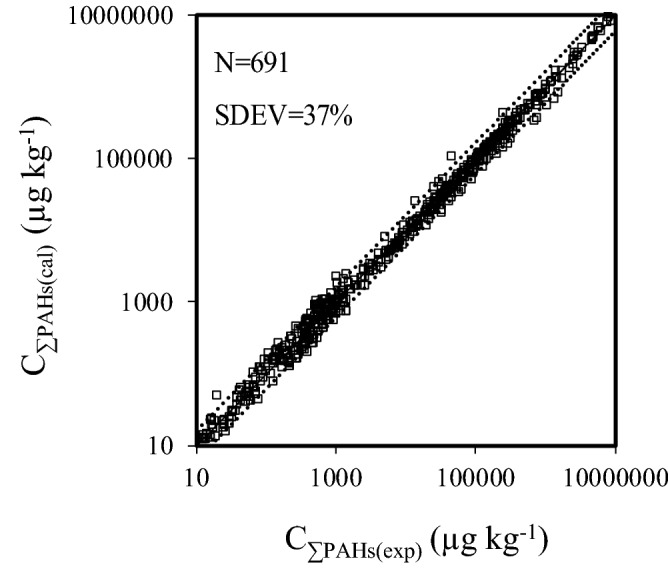
Fitted C_ƩPAHs(cal)_ versus C_ƩPAHs(exp)_ in sediments sampled in globe excluding China. The y = x line (solid line) indicates a 1:1 relationship between C_ƩPAHs(cal)_ and C_ƩPAHs(exp)_. Dashed lines in the plots indicate the SDEV values from the reference lines.

With the established multiple linear relationship (Eq. 8), we found that the total PAHs concentrations (C_∑PAHs_) in sediments can be predicted from the concentrations of four characteristic congeners with high R^2^ but low SDEV value. Figure 5c shows that C_∑PAHs(cal)_ are consistent with C_∑PAHs(exp)_ in sediments sampled in China. Scatterplots of unstandardized residuals (the difference between C_ƩPAHs(cal)_ and C_ƩPAHs(exp)_) with C_Nap_, C_Acy_, C_Phe_ or C_BaA_ in sediments distributed regularly on both sides of the horizontal line and no obvious positive or negative trend existed (Fig. S1). This indicates the significant stability of the established linear relationship^45^. Furthermore, C_ƩPAHs(cal)_ also agreed well with C_ƩPAHs(exp)_ using additional concentration data (Table S6) in sediment samples from elsewhere around the globe (N = 691) (Fig. 6), suggesting that the relationship can be applied to predict C_ƩPAHs_ in sediments that are not only localized in China. Therefore, Nap, Acy, Phe, and BaA can be also employed as characteristic congeners of sixteen PAHs in sediments.

The same characteristic congeners observed for PAHs in sediments and in emission sources indicates that the concentration of PAHs in sediments are largely depended on their emission, which was consistent with the reported results^46–48^. In previous studies^17,29–31^, PAHs emissions were not involved in the relationship of predicting PAHs concentration in sediments using *f*_oc_ (Eq. 1), which does not hold true in some cases. For example, for a given *f*_oc_, C_ƩPAHs_ in sediments can vary by 1–3 orders of magnitude (Fig. 1a) because of the difference in PAHs emissions in various regions. For another example, although the mean *f*_oc_ (6.4%) in sediments from the upper reach of Huaihe River^50^ was higher than that in sediments from the lower reach of Huaihe River (*f*_oc_ = 4.1%)^51^, C_ΣPAHs_ in sediments from the lower reach of Huaihe River (mean value = 1721.7 µg kg^−1^)^50^ were higher than that from the upper reach (mean value = 400.5 µg kg^−1^)^51^. This can be attributed to the higher emission intensity of PAHs in lower reach of Huaihe River region than that in the upper reach region^41,52,53^. Moreover, a significantly positive correlation between mean concentrations of thirteen PAHs (except for Nap, Acy, and Ace) in sediment samples derived from globe (N = 1445) and their mean EFs in fifteen emission divisions was also observed (Fig. S2), indicating that the concentration of PAHs in sediments also mainly depends on the PAHs emission. The deviation of Nap, Acy, and Ace from the linear relationship in Fig. S2 can be attributed to their relatively low log*K*_ow_ but high *S*_w_ (Table S1), making them not be readily adsorbed by organic matters in sediments but tend to be more readily dissolved in water^48^.

### Relationship between PAHs concentrations in sediments and EFs in emission sources

**Figure 7:**
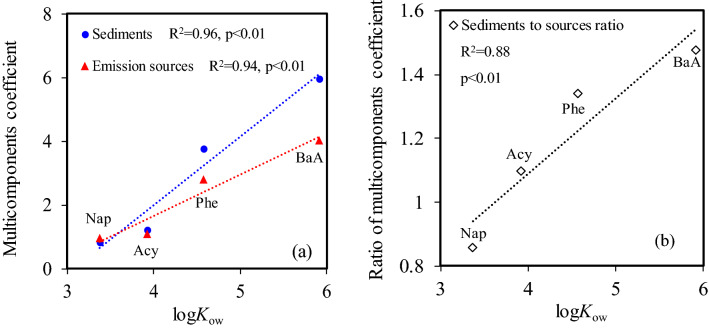
Relationships of multicomponents coefficients in Eq. (8) and that in Eq. (5) (**a**), as well as ratios of multicomponents coefficient in Eq. (8) to that in Eq. (5) (**b**) with the log*K*_ow_ of four characteristic PAHs congeners. Dashed lines in the plots are linear regressions.

Significance of multicomponent coefficients in Eqs. (5) and (8) were less than 0.05 (*p* < 0.05), which are statistically significant. However, significance of intercept in Eq. (5) was greater than 0.05 (*p* > 0.05), which is statistically insignificant. The significant intercept (*p* < 0.05) in Eq. (8) can be assigned to background concentrations of PAHs in sediments^54,55^. Moreover, significantly positively linear relationships of the multicomponent coefficients in Eq. (5) and that in Eq. (8) with the log*K*_ow_ of four characteristic congeners were observed (Fig. 7a). Interestingly, the coefficient of characteristic congeners with larger log*K*_ow_, such as BaA, in Eq. (8) are higher than that in Eq. (5) (Fig. 7a). This could be attributed to the influence of sorption of PAHs in sediments and their biodegradation in the environment. For PAHs with larger log*K*_ow_, they tend to be more readily adsorbed in sediments organic matter by partitioning than the PAHs with smaller log*K*_ow_^56,57^. Meanwhile, PAHs congeners with relatively low log*K*_ow_ tend to be more readily degraded than those with relatively high log*K*_ow_^58,59^, presented by the positively linear relationship of PAHs log*K*_ow_ with their biodegradation half-life (Fig. S3). Therefore, a positively linear relationship between the ratio of multicomponents coefficient from the multiple linear relationship in sediments (Eq. 8) to that from the multiple linear relationship in emission sources (Eq. 5) and the log*K*_ow_ of four PAHs congeners can be observed in Fig. 7b. This suggests that the distribution of PAHs in sediments could also be dependent on their environmental behaviors including sorption and biodegradation in addition to their emissions. In previous study^46^, significant linear relationships between the concentrations of sixteen PAHs in sediments (C_PAHs_) with their emissions (E_PAHs_) were established (Eq. 10). Moreover, positive and negative relationships of **K** (Eq. 11) and **L** (Eq. 12) with log*K*_ow_ were established, respectively.

10 $${\text{C}}_{{{\text{PAHs}}}} = {\mathbf{K}} \times {\text{ E}}_{{{\text{PAHs}}}} + {\mathbf{L}}$$

11 $$\begin{aligned} {\mathbf{K}} = & {\text{ 3E}} - 0{6 } \times {\mathbf{log}}K_{{{\mathbf{ow}}}}^{{{8}.{24}}} \\ & \left( {{\text{N }} = { 16},{\text{ F }} = { 427},{\text{ R}}^{{2}} = \, 0.{93},p < \, 0.0{1}} \right) \\ \end{aligned}$$

12 $$\begin{aligned} {\mathbf{L}} = & \, - {117}.{41}\left( { \pm {12}.{48}} \right) \, \times {\mathbf{log}}K_{{{\mathbf{ow}}}} + { 416}.{16}\left( { \pm {58}.{13}} \right) \\ & \left( {{\text{N }} = { 16},{\text{ F }} = { 384},{\text{ R}}^{{2}} = \, 0.{86},p < \, 0.0{1}} \right) \\ \end{aligned}$$

**Figure 8:**
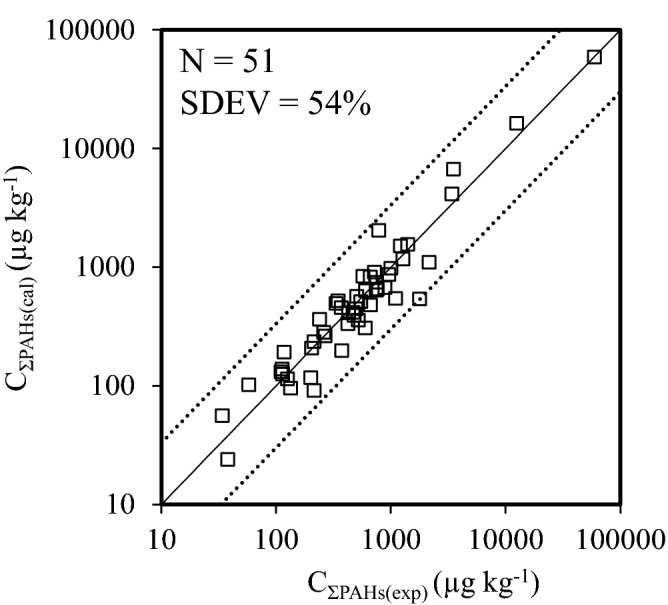
Fitted C_ƩPAHs(cal)_ using E_PAHs_ and log*K*_ow_ of four characteristic PAHs congeners versus C_ƩPAHs(exp)_ in sediments sampled in investigated provinces of China and other countries. The y = x line (solid line) indicates a 1:1 relationship between C_ƩPAHs(cal)_ and C_ƩPAHs(exp)_. Dashed lines in plots indicate the SDEV values from the reference lines.

In this study, a multilinear relationship of C_ΣPAHs_ with C_Nap_, C_Acy_, C_Phe_, and C_BaA_ was established (Eq. 8). The concentration of four characteristic PAHs congeners in sediments can be calculated using their emissions and log*K*_ow_ (Eqs. 10–12). Therefore, C_ΣPAHs_ in sediments can be predicted using the emissions and log*K*_ow_ of four characteristic PAHs congeners, in which log*K*_ow_ can be accounted for PAHs partition ability. Mean C_∑PAHs(exp)_ in surface sediments sampled in investigated provinces of China (N = 30) and other countries (N = 21) versus C_∑PAHs(cal)_ predicted using E_PAHs_ (Tables S7 and S8) and log*K*_ow_ of four characteristic PAHs congeners is presented in Fig. 8. The SDEV value between C_∑PAHs(exp)_ and C_∑PAHs(cal)_ is 54%, suggesting that C_∑PAHs(cal)_ are well consistent with C_∑PAHs(exp)_. Therefore, the established model in this study can be used to predict C_ΣPAHs_ in sediments with high accuracy, resulting in decreasing cost of laboratory analysis.

The correlations that have been previously described in literature of C_ƩPAHs_ with C_BaP_^36,37^, C_Pyr_^38^ or C_Ace_^39^ could be attributed to the PAHs emissions in the investigated region from one emission source or emission sources with similar EFs (Table 1). For example, PAHs in sediment samples of Norway were mainly from manufactured gas plants and aluminum smelters^38^, in which Pyr is the dominant congener with relatively high EFs^40,41,52^. However, the multiple linear relationship established herein (Eq. 6) gives a useful way to predict the C_ƩPAHs_ in sediments using PAHs emissions emitted from major emission sources around the world as seen by the good correlation with the large and diverse sample size. Therefore, this relationship would be valuable for predicting total PAHs concentrations and assessing their risks in sediments.

## Conclusion and perspectives

A multiple linear relationship of C_∑PAHs_ with C_Nap_, C_Acy_, C_Phe_, and C_BaA_ in sediments was established employing the reported data in the past 30 years. This suggested the selected four PAHs congeners, including Nap, Acy, Phe, and BaA, are the characteristic congeners in sediments. Moreover, the multiple linear relationship of EF_∑PAHs_ with the EFs of the four congeners was also developed. The same characteristic congeners observed for PAHs in sediments and in emission sources indicates that the concentration of PAHs in sediments are largely dependent on their emissions. Additionally, the ratio of multicomponents coefficient from the multiple linear relationship in sediments to that from the multiple linear relationship in emission sources correlated positively with log*K*_ow_ of the four congeners. Therefore, a model for predicting C_ΣPAHs_ in sediments was established using the emissions and log*K*_ow_ of four PAHs congeners. The SDEV value between C_∑PAHs(exp)_ and C_∑PAHs(cal)_ was 54%, suggesting the established model can accurately predict C_ΣPAHs_ in sediments.

Although the relationship established in this study could be used to predict total sixteen PAHs concentration in surface sediments of China and other countries, the application of this method for predicting additional PAHs in sediment, such as alkylated-PAHs, needs further verified.

## Methodology

### Literature search

Concentration data of sixteen parent PAHs (Table S4 and Table S6) in global bottom sediments of fresh water reported in the past 30 years were collected. A systematic literature retrieval was performed using the ISI Web of Science database, Google®Scholar, WanFang Data of E-Resources and China Knowledge Resource Integrated Database including master/doctoral dissertation using the terms of “polycyclic aromatic hydrocarbons” or “PAHs” and “sediment/sediments” as the primary keywords^60^. Articles were then examined individually to ensure that the duplicates and irrelevant articles were excluded from further analysis. In addition, articles without individual PAHs concentration data and/or articles that did not report QA/QC procedure and limits of detection (LODs) were also excluded from further analysis^60^. In order to perform the required comparisons in this study, it was assumed that there were no significant differences in the sampling process and analysis among the investigating groups/laboratories^60,61^. In total, 22,349 individual PAH concentrations from 1445 sediments samples were collected from 1184 publications and then used for meta-analysis (Table S4 and Table S6).

### PAHs emissions

According to the previous study^40^, PAHs emissions in globe was primarily emitted from coking production, petroleum refineries, domestic and industrial coal combustion, straw and firewood burning, iron-steel industry, transport petroleum, and primary Al production, which accounting for more than 92% of all source contributions. Therefore, PAHs emissions from these nine sources were calculated using a previously reported approach for analysis^40,41^. Emission factors (EFs, g t^−1^) of sixteen PAHs from above nine emission sources were summarized in Table S5. Provincial and national PAHs emissions (E_PAHs_, t a^−1^) were calculated using Eq. (13)^40,41,52^:

13 $${\text{E}}PAHs = \sum\limits_{k,l} {EF{\text{i}},j,k \times Xj,k \times Ak}$$

where i, j, and k represent each PAH congener, sources, and technology, respectively.

EF (g t^−1^) is the emission factor of the PAHs congener i (Table S5). X is the fraction of the activity rate contributed by a given technology j, which was calculated using the technology split method^40,41,52^. Activity data (A, 10^4^ t a^−1^) in source j, from China, were obtained directly from China Statistical Yearbook (2001–2018) and China Energy Statistical Yearbook (2001–2018), edited by National Bureau of Statistics^40^. The data from other countries were derived from Food and Agriculture Organization of the United Nations, International Yearbook of Industrial Statistics (2004–2018), and International Energy Agency World Energy Statistics and Balances^40^.

For the technology splitting approach, six sources (coking production, industrial coal combustion, indoor straw and firewood burning, iron-steel industry, and primary Al production) were divided into two or three divisions with or without different emission mitigation measures^40,41^. For the remaining three sources, fixed EF_PAHs_ without divisions were used^40,41^. The time-dependent fractions of technology divisions were calculated using a series of S-shaped curves (Eq. 14, Table S9).

14 $${\text{X}}\left( {\text{t}} \right) = \left( {X_{0} - X_{f} } \right)e^{{ - \frac{{\left( {t - t_{0} } \right)^{2} }}{{2s^{2} }}}} + X_{f}$$

where *X*_0_ and *X*_f_ are initial and final fractions of a certain technology division, respectively. *t*_0_ is the start time of technology transition, and *s* is a rate. The *X*_f_, *X*_0_, *t*_0_, and *s* were illustrated in Table S9^40^.

### Data analysis

11,937 individual concentrations data from 754 sediments sampled from China were used to establish the relationship between C_ƩPAHs_ and the concentration of selected congeners in sediments (Table S4). To validate the established relationship, 10,412 individual concentrations data from 691 sediments sampled from globe (excluding China) were used as a test set (Table S6). Concentrations that were reported to be below the method LODs were assigned half the value of the reported LODs^61,62^. Concentration unit of PAHs were set uniformly to micrograms per kilogram (µg kg^−1^) of dry weight. Prior to statistical analysis, a histogram with normal curve was viewed and a Kolmogorov–Smirnov test were performed to verify the normality of variables^38^. If the significance (*p*) is greater than 0.05 (*p* > 0.05), it can be judged that the variables are normal^38^. Hierarchical clustering analysis (HCA) and classifications were performed using the SPSS Statistics 19.0 software (Version 19.0, Chicago, IL, USA) according to relative similarities of EFs in emission sources and concentrations in sediments of sixteen PAHs^42,43^. Statistical analysis, including linear and multilinear regression, were also performed using the SPSS Statistics 19.0 with a critical significance (*p*) up to 0.05 to check significance. Provincial or national PAHs emissions combined with their observed C_∑PAHs_ in surface sediments were used to evaluate the established model between C_∑PAHs_ and emissions of the four characteristic congeners. Moreover, C_∑PAHs_ in surface sediments sampled in the same province or nation at the same year were expressed by the geometric mean value^63^.

Percent sample deviation (SDEV, Eq. (15)) was calculated based on the relative error between the experimental values (C_exp_) and the calculated value (C_cal_)^64^. In addition to the SDEV, significance of F test (*p*) and correlation coefficient (R^2^) were used to evaluate the goodness of the fitting and the established correlations by regression analysis^64^.

15 $${\text{SDEV }} = \sqrt {\frac{{\sum\limits_{{}}^{{}} {\left( {\frac{{{\text{C}}_{cal} - C_{\exp } }}{{C_{\exp } }}} \right)^{2} } }}{{{\text{N}} - k}}} \times 100$$

where N is the number of experimental values and k is the number of predictors for linear regression.

## Supplementary Information

Supplementary Information.
