# Supplementary material for: Predicting the total PAHs concentrations in sediments from selected congeners using a multiple linear relationship
Source: Sci Rep. 2022 Feb 28;12:3334. doi: 10.1038/s41598-022-07312-2 (PMC8885927; doi:10.1038/s41598-022-07312-2)
Supplement: Supplementary file 1 — Supplementary Information. [file 41598_2022_7312_MOESM1_ESM.docx]

**Supporting Information**

Predicting the Total PAHs Concentrations in Sediment from selected Congeners Using a Multiple Linear Relationship

Weiwei Wang^a,b,c^, Huaping Xu^d^, Xiaolei Qu^e^, Kun Yang^a,b,c^^,f*^, Daohui Lin^a,b,c^

^a^Department of Environmental Science, Zhejiang University, Hangzhou 310058, China;

^b^Key Laboratory of Environmental Pollution and Ecological Health of Ministry of Education, Hangzhou 310058, China;

^c^Zhejiang Provincial Key Laboratory of Organic Pollution Process and Control, Hangzhou 310058, China;

^d^Mathematics Teaching and Research Section, College of Pharmaceutical Sciences, Zhejiang Chinese Medical University, Hangzhou 310053, China;

^e^State Key Laboratory of Pollution Control and Resource Reuse, School of the Environment, Nanjing University, Jiangsu 210023, China

^f^Zhejiang University-Hangzhou Global Scientific and Technological Innovation Center, Hangzhou 311200, China

^*^Corresponding author (Kun Yang). E-mail: [kyang@zju.edu.cn](mailto:kyang@zju.edu.cn)

**Caption of Tables and Figures**

**Table S1.** Physico-chemical characteristics of sixteen PAHs listed by USEPA.

**Table S2.** Concentrations of ∑PAHs and the content of total organic carbon (*f*_oc_) in sediment samples from globe.

**Table S3.** The information about previous methods used to predict C_∑PAHs_ in sediments, including equations, SDEV, sample numbers (N), R^2^, and references.

**Table S4.** Concentrations of sixteen individual PAH congeners and ∑PAHs in sediments sampled from China.

**Table S5.** Emission factors of sixteen PAHs for fifteen emission divisions from nine sources (g t^-1^).

**Table S6.** Concentrations of sixteen individual PAH congeners and ∑PAHs in sediments sampled from globe excluding China.

**Table S7.** Emission (t a^-1^) of individual PAHs in China from 2000 to 2017.

**Table S8.** Emission (t a^-1^) of individual PAHs in other countries emitted in the year corresponding to the sampling time of surface sediments (Table S6).

**Table S9.** List of emission sources with technology splits.

**Fig. S1.** Relationships of C_Nap_ (a), C_Ace_ (b), C_Phe_ (c) and C_BaA_ (d) in sediments sampled from China with unstandardized residuals.

**Fig. S2.** Relationship between mean concentrations of thirteen PAHs congeners in sediments sampled from globe and their mean EFs in fifteen emission divisions. Dashed line in plot is the linear regression.

**Fig. S3.** Relationships between log*K*_ow_ and biodegradation half-life of four characteristic PAHs congeners. Dashed line in the plot is linear regressions.

# Table S1 Physico-chemical characteristics of sixteen PAHs listed by USEPA

| Name | Abbreviation | Molecular Weight (g/mol) | S_w_ (mg L^-1^) (25℃) | ^a^log*K*_ow_ (25℃) | ^b^Biodegradation half-life (day) |
| --- | --- | --- | --- | --- | --- |
|  |  |  |  |  |  |
| Naphthalene | Nap | 128 | 31.5 | 3.37 | 3 |
| Acenaphthene | Ace | 154 | 3.93 | 3.92 | 39 |
| Acenaphthylene | Acy | 152 | 3.93 | 4.00 | 38 |
| Fluorene | Flo | 166 | 1.69 | 4.18 | 44 |
| Anthracene | Ant | 178 | 0.08 | 4.54 | 42 |
| Phenanthrene | Phe | 178 | 1.15 | 4.57 | 123 |
| Pyrene | Pyr | 202 | 0.13 | 5.18 | 147 |
| Fluoranthene | Flu | 202 | 0.21 | 5.22 | 237 |
| Chrysene | Chr | 228 | 0.002 | 5.86 | 286 |
| Benz(a)anthracene | BaA | 228 | 0.009 | 5.91 | 378 |
| Benzo(b)fluoranthene | BbF | 252 | 0.002 | 5.80 | 282 |
| Benzo(k)fluoranthene | BkF | 252 | 0.001 | 6.00 | 351 |
| Benzo(a)pyrene | BaP | 252 | 0.004 | 6.04 | 224 |
| Indeno(1,2,3-cd)pyrene | IcdP | 276 | 0.001 | 6.50 | 330 |
| Benzo(ghi)perylene | BghiP | 276 | 0.0003 | 6.50 | 562 |
| Dibenzo(a,h)anthracene | DahA | 278 | 0.0006 | 6.75 | 420 |

^a, b^ Data cited from https://pubchem.ncbi.nlm.nih.gov/

# Table S2 Concentrations of ∑PAHs and the content of total organic carbon (*f*_oc_) in sediment samples from globe.

| **Sites** | **S1** | **S2** | **S3** | **S4** | **S5** | **S6** | **S7** | **S8** | **S9** | **S10** | **S11** | **S12** | **S13** | **S14** | **S15** | **S16** | **S17** | **S18** | **Reference** |
| --- | --- | --- | --- | --- | --- | --- | --- | --- | --- | --- | --- | --- | --- | --- | --- | --- | --- | --- | --- |
| **C_ΣPAHs_** | 1768 | 2615 | 635 | 2025 | 3312 | 2836 | 2673 | 2094 | 2781 | 2677 | 4824 | 3989 | 3919 | 2707 | 3201 | 3307 | 3194 | 2999 | 1 |
| ***f*_OC_(%)** | 2.16 | 2.84 | 0.94 | 1.67 | 2.12 | 3.36 | 4.22 | 4 | 1.75 | 3.83 | 5.24 | 5.1 | 3.86 | 4.3 | 2.14 | 3.28 | 3.22 | 3.54 |  |
| **C_ΣPAHs_** | 787.19 | 425.72 | 990.25 | 878.09 | 839.24 | 795.96 | 816.81 | 799.57 | 939.18 | 852.5 | 486.36 | 855.83 | 520.15 | 469.19 | 374.57 | 295.75 | 422.81 | 728.28 | 2 |
| ***f*_OC_(%)** | 2.09 | 1.87 | 1.78 | 1.97 | 1.57 | 2.01 | 1.95 | 1.67 | 1.88 | 2.25 | 1.04 | 3.82 | 1.25 | 2.31 | 1.95 | 1.56 | 1.22 | 2.15 |  |
| **C_ΣPAHs_** | 119.3 | 239.6 | 311.6 | 288.3 | 271.8 | 214 | 197.9 | 222.3 | 61.7 | 76.6 | 142.3 | 160.8 | 227.6 | 53.4 | 238.7 | 128.3 | 220.8 | 149 | 3 |
| ***f*_OC_(%)** | 0.45 | 1.02 | 0.94 | 0.92 | 1.01 | 0.8 | 1.07 | 0.93 | 0.61 | 0.63 | 0.84 | 0.62 | 1.02 | 0.21 | 0.85 | 0.68 | 0.92 | 0.86 |  |
| **C_ΣPAHs_** | 98.29 | 119.78 | 202.63 | 80.81 | 59.09 | 192.14 | 80.47 | 74.04 | 47.4 | 112.18 | 50.74 | 139.63 | 74.04 | 41.72 | 71.61 | 327.53 | 137.72 | 231.85 | 4 |
| ***f*_OC_(%)** | 0.86 | 1.23 | 2.85 | 0.97 | 1.43 | 1.04 | 1.58 | 1.74 | 0.93 | 1.84 | 1.07 | 1.27 | 1.44 | 1.56 | 1.1 | 1.47 | 1.22 | 1.15 |  |
| **C_ΣPAHs_** | 40.2 | 972.6 | 20.96 | 16.6 | 221.7 | 34.36 | 17.36 | 36.9 | 51.4 | 58.8 | 21.76 | 85.62 | 68.9 | 56.16 | 93 | 143.9 | 341.6 | 88.4 | 5 |
| ***f*_OC_(%)** | 0.13 | 2 | 0.18 | 0.13 | 1.38 | 0.12 | 0.13 | 0.51 | 0.49 | 0.5 | 0.78 | 0.3 | 0.23 | 0.1 | 0.12 | 0.11 | 1.2 | 0.18 |  |
| **C_ΣPAHs_** | 388.7 | 439.2 | 432.4 | 99.6 | 199.2 | 285.2 | 341.9 | 392.1 | 258.8 | 453.9 | 536.1 | 556.1 | 684.4 | 341.1 | 372.1 | 388.1 | 432.5 | 348.5 | 6 |
| ***f*_OC_(%)** | 1.02 | 1.08 | 0.96 | 0.99 | 0.89 | 0.86 | 1.26 | 1.63 | 0.86 | 1.27 | 1.26 | 1.19 | 1.37 | 0.69 | 0.64 | 0.68 | 1.25 | 1.19 |  |
| **C_ΣPAHs_** | 1077 | 487 | 818 | 3684 | 806 | 1026 | 1018 | 274 | 276 | 758 | 2090 | 903 | 5370 | 2413 | 947 | 421 | 676 | 436 | 7 |
| ***f*_OC_(%)** | 3.1 | 1.4 | 0.4 | 1.3 | 0.8 | 0.5 | 2.1 | 2.2 | 0.9 | 2.1 | 1.8 | 1.5 | 1.5 | 1.1 | 0.7 | 0.8 | 1.4 | 0.9 |  |
| **C_ΣPAHs_** | 3828.58 | 358.54 | 777.29 | 299.91 | 460.09 | 448.71 | 286.41 | 424.91 | 361.78 | 507.07 | 212.91 | 537.6 | 290.57 | 326.93 | 217.38 | 126.08 | 119.49 |  | 8 |
| ***f*_OC_(%)** | 2.83 | / | / | 0.33 | 1.18 | 1.37 | 0.93 | / | 1.06 | 1.08 | 1.06 | 1.35 | 0.68 | 0.98 | 0.71 | 0.22 | 0.09 |  |  |
| **C_ΣPAHs_** | 216.9 | 596.6 | 877.5 | 95.2 | 238.2 | 143 | 428.3 | 230 | 386.2 | 521.2 | 867.7 | 338.7 | 85.7 | 935.2 | 144.8 | 303.2 |  |  | 9 |
| ***f*_OC_(%)** | 2.46 | 2.63 | 5 | 0.88 | 1.93 | 4.27 | 0.69 | 0.27 | 17.81 | 5.06 | 15.64 | 2.18 | 7.14 | 3.68 | 0.32 | 32.94 |  |  |  |
| **C_ΣPAHs_** | 34.1 | 331.7 | 129.6 | 238.2 | 148.1 | 249.2 | 8.3 | 504.3 | 387.5 | 6.7 | 492.6 | 1585.7 |  |  |  |  |  |  | 10 |
| ***f*_OC_(%)** | 0.6 | 0.4 | 0.7 | 0.7 | 0.6 | 1.4 | 0.1 | 0.9 | 1.2 | 0.1 | 2.2 | 0.8 |  |  |  |  |  |  |  |
| **C_ΣPAHs_** | 380.14 | 338.55 | 84.69 | 110.9 | 61.91 | 524.03 | 233.65 | 212.69 | 334.1 | 840.53 | 123.5 | 203.3 |  |  |  |  |  |  | 11 |
| ***f*_OC_(%)** | 0.88 | 0.68 | 1.16 | 0.15 | 0.69 | 2.6 | 0.41 | 0.88 | 0.7 | 2.56 | 0.41 | 0.86 |  |  |  |  |  |  |  |
| **C_ΣPAHs_** | 133 | 31 | 78.9 | 103 | 117 | 43.7 | 903 | 17 | 162 | 401 | 20.5 |  |  |  |  |  |  |  | 12 |
| ***f*_OC_(%)** | 0.72 | 0.1 | 0.07 | 0.05 | 0.24 | 0.07 | 0.23 | 0.11 | 1.05 | 1.43 | 0.42 |  |  |  |  |  |  |  |  |
| **C_ΣPAHs_** | 904.81 | 759.09 | 378.54 | 1397.45 | 4584.61 | 707.70 | 438.23 | 5770.30 | 1406.48 | 1009.41 |  |  |  |  |  |  |  |  | 13 |
| ***f*_OC_(%)** | 1.64 | 0.87 | 2.06 | 2.64 | 2.25 | 2.73 | 1.63 | 1.86 | 2.06 | 1.70 |  |  |  |  |  |  |  |  |  |
| **C_ΣPAHs_** | 2131 | 551 | 757 | 4323 | 2134 | 290 | 6118 | 757 | 1162 |  |  |  |  |  |  |  |  |  | 14 |
| ***f*_OC_(%)** | 1 | 2 | 2 | 7 | 4 | 2 | 4 | 1 | 1 |  |  |  |  |  |  |  |  |  |  |
| **C_ΣPAHs_** | 347.4 | 2061.5 | 1462.6 | 460 | 770.5 | 62.9 | 2322.4 |  |  |  |  |  |  |  |  |  |  |  | 15 |
| ***f*_OC_(%)** | 1.54 | 0.88 | 0.75 | 0.94 | 1.02 | 0.54 | 1.48 |  |  |  |  |  |  |  |  |  |  |  |  |
| **C_ΣPAHs_** | 94.53 | 196.99 | 67.29 |  |  |  |  |  |  |  |  |  |  |  |  |  |  |  | 16 |
| ***f*_OC_(%)** | 0.47 | 0.87 | 0.77 |  |  |  |  |  |  |  |  |  |  |  |  |  |  |  |  |
|  | **S19** | **S20** | **S21** | **S22** | **S23** | **S24** | **S25** | **S26** | **S27** | **S28** | **S29** | **S30** | **S31** | **S32** | **S33** | **S34** | **S35** | **S36** | **Reference** |
| **C_ΣPAHs_** | 3308 | 3496 | 4805 | 3872 | 5534 | 3849 | 5809 | 8191 | 11060 | 14806 | 12040 | 10893 | 9088 | 8734 | 7619 | 7929 | 7941 | 8886 | 1 |
| ***f*_OC_(%)** | 4.17 | 3.78 | 2.13 | 4.13 | 6.33 | 6.86 | 5.18 | 4.39 | 8.16 | 9.96 | 8.35 | 8.96 | 9.73 | 6.98 | 5.63 | 4.73 | 4.91 | 5.19 |  |
| **C_ΣPAHs_** | 755.24 | 598.62 | 592.83 | 414.3 | 395.84 | 322.39 | 696.36 | 228.13 | 506.19 | 353.91 | 435.22 | 755.99 | 796.84 | 696.43 |  |  |  |  | 2 |
| ***f*_OC_(%)** | 3.04 | 1.72 | 1.99 | 2.11 | 1.78 | 2.59 | 2.24 | 1.45 | 2.33 | 2.22 | 0.89 | 1.8 | 1.89 | 1.97 |  |  |  |  |  |
| **C_ΣPAHs_** | 125.7 | 357.1 | 117.1 | 215.4 | 60.4 | 1369.1 | 280.1 | 247.3 | 450.1 | 145.6 | 91.6 | 118.1 | 390.5 |  |  |  |  |  | 3 |
| ***f*_OC_(%)** | 0.7 | 0.68 | 0.54 | 0.81 | 0.49 | 1.49 | 0.44 | 0.94 | 0.98 | 0.79 | 0.28 | 0.26 | 0.94 |  |  |  |  |  |  |
| **C_ΣPAHs_** | 100.83 | 23.03 | 40.56 | 112.82 | 292.65 | 117.14 | 152.38 | 199.03 | 152.84 | 67.26 |  |  |  |  |  |  |  |  | 4 |
| ***f*_OC_(%)** | 1.03 | 1.31 | 1 | 0.7 | 1.42 | 1.22 | 0.86 | 1.13 | 0.96 | 1.27 |  |  |  |  |  |  |  |  |  |
| **C_ΣPAHs_** | 634.5 | 56.8 | 155.3 | 40 | 1358 | 32.1 | 16.28 | 68.1 |  |  |  |  |  |  |  |  |  |  | 5 |
| ***f*_OC_(%)** | 1.73 | 0.2 | 0.8 | 0.12 | 2.01 | 0.14 | 0.48 | 0.11 |  |  |  |  |  |  |  |  |  |  |  |
| **C_ΣPAHs_** | 404 | 396.9 | 414.3 | 424.4 | 406.9 | 500.9 |  |  |  |  |  |  |  |  |  |  |  |  | 6 |
| ***f*_OC_(%)** | 1.4 | 1.2 | 1.45 | 1.25 | 1.62 | 1.58 |  |  |  |  |  |  |  |  |  |  |  |  |  |

# Table S3 The information about previous methods used to predict C_∑PAHs_ in sediments, including equations, SDEV, sample numbers (N), R^2^, and references.

| **Equations** | **R^2^** | **SDEV** | **N** | **Ref.** |
| --- | --- | --- | --- | --- |
| C_∑PAHs_ = 8.257 × C_BaP_+13.98 | 0.51 | 104% | 51 | 50 |
| C_∑PAHs_ = 34.70 × C_BaP_ +0.15 | 0.81 | 34% | 8 | 48 |
| C_∑PAHs_ = 110.68 × C_Ace_ + 63.36 | 0.83 | 27% | 10 | 58 |
| logC_∑PAHs_ = 0.943×logC_pyr_+1.401 | 0.95 | 38% | 245 | 79 |

# Table S4 Concentrations of sixteen individual PAH congeners and ∑PAHs in sediments sampled from China

| **Sites** | **NaP** | **Acy** | **Ace** | **Flo** | **Phe** | **Ant** | **Flu** | **Pyr** | **BaA** | **Chr** | **BbF** | **BkF** | **BaP** | **IcdP** | **DahA** | **BghiP** | **ΣPAHs** | **Reference** |
| --- | --- | --- | --- | --- | --- | --- | --- | --- | --- | --- | --- | --- | --- | --- | --- | --- | --- | --- |
| Site 1 | 771.09 | 41.77 | 344.24 | 245.17 | 289.16 | 52.72 | 6.72 | 239.06 | 71.86 | 77.46 | 194.85 | 42.01 | 90.4 | 78.22 | 22.09 | 89.83 | 2656.65 | 17 |
| Site 2 | 657.56 | 15.16 | 77.51 | 79.4 | 103.49 | 15.59 | 68.19 | 55.64 | 7.18 | 17.62 | 16.85 | 0.02 | 6.72 | 2.23 | 0.51 | 2.71 | 1126.38 |  |
| Site 3 | 582.06 | 24.72 | 51.62 | 129.82 | 312.03 | 60.03 | 256.16 | 272.13 | 42.41 | 117.84 | 83.22 | 52.08 | 105.78 | 59.36 | 14.32 | 52.93 | 2216.51 |  |
| Site 4 | 126.68 | 10.66 | 51 | 208.65 | 54.23 | 10.54 | 93.34 | 77.43 | 15.08 | 13.9 | 25.53 | 16.11 | 6.29 | 6.37 | 1.33 | 4.84 | 721.98 |  |
| Site 5 | 90.56 | 4.19 | 22.86 | 27.88 | 37.76 | 4.86 | 32.67 | 24.52 | 3.66 | 7.22 | 15.83 | 9.91 | 3.82 | 2.65 | 0.54 | 2.72 | 291.65 |  |
| Site 6 | 145.79 | 7.83 | 33.48 | 81.69 | 215.01 | 41.23 | 275.56 | 220.19 | 75.73 | 171.9 | 106.85 | 66.88 | 136.3 | 130.04 | 5.64 | 94.66 | 1808.78 |  |
| Site 7 | 75.69 | 4.08 | 33.98 | 40.79 | 56.15 | 9.69 | 55.69 | 46.16 | 3.35 | 12.73 | 21.32 | 13.34 | 7.44 | 6.3 | 1.21 | 4.97 | 392.89 |  |
| Site 8 | 85.16 | 42.83 | 21.09 | 37.79 | 83.57 | 29.46 | 326.82 | 194.15 | 34.37 | 84.18 | 84.79 | 53.07 | 30.1 | 17.85 | 2.98 | 11.19 | 1139.4 |  |
| Site 9 | 76.97 | 3.91 | 27.04 | 28.04 | 31.26 | 6.08 | 32.44 | 27.22 | 3.04 | 7.56 | 13.95 | 8.73 | 4.27 | 2.89 | 0.65 | 0.01 | 274.06 |  |
| Site 10 | 226.21 | 13.99 | 358.71 | 255.55 | 134.74 | 27.11 | 2.41 | 78.34 | 30.47 | 36.5 | 86.15 | 20.19 | 36.76 | 38.89 | 9.57 | 1.28 | 1356.87 |  |
| Site 1 | 6 | 3.9 | 4.2 | 7.4 | 48 | 8 | 33.1 | 23.5 | 12.2 | 15.1 | 33 | 7.1 | 5.6 | 4.1 | 9.4 | 11.3 | 231.9 | 18 |
| Site 2 | 7.3 | 2.2 | 5.4 | 9.4 | 40.7 | 10.9 | 46.9 | 36.9 | 19.2 | 26.9 | 53.9 | 13.6 | 12.7 | 5.8 | 13.8 | 17 | 322.6 |  |
| Site 3 | 21.7 | 5.6 | 4.9 | 10.5 | 52.2 | 9.6 | 43.9 | 32.7 | 16.9 | 20.4 | 43.7 | 10 | 9.2 | 5.4 | 11.1 | 13.5 | 311.3 |  |
| Site 4 | 8.9 | 4.2 | 0.8 | 7.5 | 21.6 | 1.9 | 15.8 | 10.6 | 3.3 | 5.9 | 12.5 | 0.4 | 1.5 | 1 | 4.2 | 4.5 | 104.6 |  |
| Site 5 | 9.7 | 4 | 0.8 | 7.2 | 20.5 | 1.8 | 15.1 | 10.1 | 3.1 | 5.5 | 12.5 | 0.6 | 1.3 | 0.9 | 3.7 | 4.5 | 101.3 |  |
| Site 6 | 7.2 | 4.3 | 0.8 | 7.3 | 27.7 | 2.2 | 13.9 | 12.8 | 3.7 | 5.5 | 13.4 | 0.3 | 1.2 | 0.8 | 3.6 | 4.7 | 109.4 |  |
| Site 7 | 10 | 3.9 | 1.1 | 10.3 | 36.6 | 3 | 19.4 | 14.7 | 5.2 | 7.9 | 18.1 | 2 | 3.2 | 1.3 | 5.4 | 6.2 | 148.3 |  |
| Site 8 | 75 | 17.7 | 12.9 | 91.1 | 183.1 | 41 | 184 | 148.9 | 82.4 | 71 | 245.5 | 79.8 | 86.5 | 22.1 | 71.9 | 82.1 | 1495 |  |
| Site 1 | 23.37 | 13 | 0.85 | 7.72 | 75.7 | 0.85 | 20.26 | 37.85 | 49.52 | 10.49 | 62.94 | 8.4 | 16.48 | 14.56 | 10.51 | 13.46 | 365.96 | 19 |
| Site 2 | 25.56 | 13.32 | 0.85 | 11.76 | 92.07 | 0.85 | 40.09 | 64.59 | 135.42 | 23.55 | 137.62 | 28.78 | 32.37 | 47.51 | 31.98 | 36.47 | 722.79 |  |
| Site 3 | 16.25 | 12.84 | 0.85 | 5.56 | 72.07 | 0.85 | 18.28 | 27.53 | 46.22 | 8.71 | 50.11 | 8.56 | 14.02 | 13.22 | 9.91 | 12.08 | 317.06 |  |
| Site 4 | 16.54 | 12.84 | 0.85 | 7.91 | 94.19 | 0.85 | 28.78 | 46.74 | 54.77 | 10.74 | 64.26 | 9.73 | 20.58 | 21.87 | 11.43 | 19.29 | 421.37 |  |
| Site 1 | 50.4 | 6.2 | 10.3 | 27.6 | 157 | 19.6 | 163 | 135 | 86.4 | 92.7 | 90.2 | 45.1 | 76.6 | 81 | 20.1 | 85.9 | 1147.1 | 20 |
| Site 2 | 57.5 | 7.9 | 12.9 | 18.8 | 161 | 15.5 | 249 | 198 | 143 | 152 | 190 | 91.6 | 116 | 145 | 49.9 | 115 | 1723.1 |  |
| Site 3 | 89.6 | 15.9 | 12.1 | 42.7 | 277 | 45.5 | 401 | 338 | 183 | 142 | 301 | 113 | 182 | 193 | 38.2 | 221 | 2595 |  |
| Site 4 | 57.1 | 7.42 | 8.75 | 26 | 152 | 20.3 | 119 | 99.4 | 40.1 | 39.6 | 80.3 | 23.1 | 30.6 | 35.2 | 5.75 | 48.8 | 793.42 |  |
| Site 5 | 45.6 | 29.1 | 154 | 258 | 2810 | 341 | 5.74 | 1880 | 789 | 748 | 604 | 370 | 551 | 538 | 45.7 | 527 | 9696.14 |  |
| Site 6 | 552 | 152 | 25.3 | 78.9 | 748 | 86.4 | 795 | 559 | 114 | 211 | 156 | 47.1 | 52 | 19.1 | 57.4 | 61.4 | 3714.6 |  |
| Site 1 | 46.84 | 2.81 | 2.71 | 22.33 | 67.38 | 7 | 36.7 | 27.22 | 17.14 | 30.27 | 30.27 | 0.06 | 15.33 | 15.4 | 1.23 | 14.77 | 337.46 | 21 |
| Site 2 | 198.43 | 2.77 | 3.72 | 26.67 | 69.66 | 4.54 | 25.32 | 20.18 | 9.29 | 16.4 | 18.18 | 0.06 | 11.93 | 4.35 | 0.73 | 10.18 | 422.41 |  |
| Site 3 | 30.03 | 3.97 | 3.27 | 51.48 | 109.55 | 12.99 | 95.19 | 64.59 | 34.92 | 64.16 | 75.5 | 0.06 | 35.94 | 21.24 | 3.71 | 40.14 | 646.74 |  |
| Site 4 | 56.23 | 6.38 | 5.08 | 74.18 | 155.71 | 15.86 | 121.8 | 77.09 | 36.33 | 76.52 | 82.66 | 0.06 | 44.99 | 59.32 | 9.25 | 48.33 | 869.79 |  |
| Site 5 | 98.1 | 7.14 | 5.12 | 74.71 | 147.27 | 18.54 | 152.61 | 94.36 | 51.59 | 96.72 | 105.53 | 0.06 | 65.79 | 69.83 | 10.65 | 54.95 | 1052.97 |  |
| Site 1 | 43.2 | 4.8 | 3.8 | 24.3 | 79.5 | 12.2 | 55.5 | 48.4 | 30.3 | 33 | 26.8 | 0.55 | 17.2 | 7.9 | 2.1 | 7.7 | 397.25 | 22 |
| Site 2 | 7.6 | 1.2 | 1.1 | 6.1 | 25.2 | 3.8 | 17.7 | 17 | 10.4 | 13.1 | 10.8 | 0.55 | 7.1 | 1.6 | 0.4 | 3.9 | 127.55 |  |
| Site 3 | 29.7 | 3 | 4.8 | 9.2 | 23.1 | 5.4 | 9.2 | 7.9 | 8.1 | 10.5 | 13.6 | 9.5 | 13.9 | 14.6 | 0.55 | 13.1 | 176.15 |  |
| Site 4 | 11.6 | 2.6 | 3 | 2.8 | 6.7 | 4.9 | 4.9 | 4.6 | 6.6 | 7 | 7.9 | 8.2 | 0.55 | 10 | 0.55 | 0.55 | 82.45 |  |
| Site 5 | 40.4 | 2.6 | 2.6 | 15.1 | 35 | 4.2 | 37.5 | 28.3 | 16.7 | 27.9 | 38.5 | 0.55 | 27 | 25.9 | 5 | 25.2 | 332.45 |  |
| Site 6 | 96 | 6.7 | 5.1 | 35.2 | 97.4 | 9.2 | 89 | 60.8 | 37.7 | 69.6 | 89.7 | 0.55 | 53.7 | 50.7 | 10.3 | 54.3 | 765.95 |  |
| Site 7 | 46.8 | 6.6 | 4.3 | 56.2 | 133.9 | 18.6 | 134.9 | 99.1 | 61.8 | 106.1 | 131 | 0.55 | 80.5 | 77.1 | 14 | 69 | 1040.45 |  |
| Site 8 | 39 | 6.1 | 4.4 | 67.9 | 171.4 | 16.1 | 137.9 | 88.1 | 47.3 | 95.4 | 110.4 | 0.55 | 66.7 | 73.2 | 10.5 | 51.5 | 986.45 |  |
| Site 1 | 980 | 17 | 59.5 | 43.2 | 461 | 612 | 408 | 394 | 1033 | 344 | 372 | 373 | 232.73 | 114.52 | 38.48 | 123.71 | 5606.14 | 23 |
| Site 2 | 51.59 | 5.56 | 27.12 | 27.69 | 125.03 | 76.38 | 94.77 | 99.26 | 120.4 | 69.84 | 76.58 | 21.76 | 27.31 | 38.88 | 12.73 | 38.82 | 913.72 |  |
| Site 3 | 37.44 | 2.51 | 13.86 | 18.11 | 64.75 | 6.44 | 37.44 | 52.45 | 17.67 | 28.8 | 27.87 | 8.22 | 16.93 | 16.06 | 5.06 | 16.4 | 370.01 |  |
| Site 4 | 167.92 | 4.81 | 25.02 | 24.38 | 186.65 | 33.07 | 149.91 | 155.17 | 116.98 | 65.28 | 95.88 | 25.61 | 45.58 | 42.84 | 15.21 | 47.31 | 1201.62 |  |
| Site 5 | 330.61 | 68.93 | 163.5 | 245.31 | 922.93 | 107.42 | 175.76 | 312.01 | 68.36 | 106.22 | 37.46 | 7.48 | 21.95 | 11.06 | 7.07 | 17.49 | 2603.55 |  |
| Site 6 | 187.13 | 7.26 | 18.28 | 65.67 | 204.82 | 31.56 | 226.47 | 68.3 | 91.76 | 97.94 | 79.52 | 20.64 | 42.9 | 31.27 | 11.2 | 31.66 | 1216.38 |  |
| Site 7 | 79.42 | 3.96 | 25.76 | 37.65 | 128.7 | 14.66 | 81.03 | 91.92 | 32.32 | 52.67 | 42.57 | 11.48 | 35.67 | 18.19 | 6.14 | 14.26 | 676.4 |  |
| Site 8 | 132.64 | 21.65 | 107.8 | 76.79 | 568.11 | 111.49 | 460.97 | 433.14 | 261.48 | 423.42 | 399.01 | 101.1 | 197.29 | 159 | 77.77 | 208.3 | 3739.95 |  |
| Site 1 | 101.42 | 13.82 | 22.37 | 27.62 | 44.14 | 75.99 | 88.67 | 91.41 | 54.1 | 36.2 | 26.63 | 26.46 | 36.53 | 37.62 | 16.75 | 107.98 | 807.71 | 24 |
| Site 2 | 77.5 | 14.15 | 19.79 | 19.78 | 27.52 | 15.7 | 55.05 | 44.18 | 24.95 | 25.1 | 23.62 | 23.52 | 25.77 | 35.9 | 13.78 | 159.25 | 605.56 |  |
| Site 3 | 195.03 | 14.32 | 24.18 | 29.34 | 47.09 | 80.44 | 99.86 | 30.96 | 120.27 | 90.26 | 88.6 | 12.64 | 35.88 | 21.33 | 7.51 | 269.08 | 1166.79 |  |
| Site 4 | 53.9 | nd | 19.69 | 15.37 | 27.06 | 50.2 | 58.96 | 51.69 | 9.99 | 10.06 | 9.96 | 10.18 | 24.83 | nd | 2.57 | 23.35 | 367.81 |  |
| Site 1 | 56.2 | 172 | 2.5 | 16.5 | 2.5 | 2.5 | 21 | 32 | 2.5 | 2.5 | 32 | 20 | 124 | 26 | 2.5 | 2.5 | 517.2 | 25 |
| Site 2 | 32.02 | 10.09 | 4.74 | 41.62 | 111.61 | 11.47 | 97.71 | 76.64 | 31.45 | 30.76 | 92.31 | 24.17 | 30.91 | 55.37 | 9.21 | 53.85 | 713.93 |  |
| Site 3 | 8.57 | 6.51 | 1.69 | 9.51 | 50.55 | 5.95 | 65.7 | 54.28 | 41.67 | 22 | 60.79 | 15.53 | 3.98 | 33.67 | 5.62 | 30.72 | 416.74 |  |
| Site 4 | 12.44 | 7.99 | 3.02 | 12.6 | 78.46 | 9.85 | 94.77 | 87.36 | 37.85 | 31.48 | 94.53 | 24.16 | 38.51 | 66.95 | 10.66 | 56.08 | 666.71 |  |
| Site 5 | 4.71 | 7.22 | 1.83 | 10.95 | 62.19 | 24.22 | 78.88 | 68.14 | 33 | 29.89 | 74.45 | 18.76 | 27.31 | 40.17 | 7.04 | 39.33 | 528.09 |  |
| Site 6 | 7.3 | 8.46 | 3.58 | 9.77 | 30.75 | 4.31 | 40.59 | 32.7 | 13.48 | 12.63 | 45.07 | 11.24 | 14.86 | 27.87 | 4.76 | 26.9 | 294.27 |  |
| Site 7 | 5.72 | 6.54 | 1.35 | 13.25 | 44.11 | 3.3 | 42.72 | 29.42 | 14.87 | 11.19 | 42.15 | 9.93 | 11.07 | 24.48 | 4.08 | 23.33 | 287.51 |  |
| Site 8 | 4.78 | 6.95 | 3.02 | 12.79 | 61.9 | 6.27 | 37.97 | 32.7 | 23.82 | 12.02 | 34.17 | 8.89 | 13.46 | 19.69 | 3.38 | 20.61 | 302.42 |  |
| Site 1 | 39 | nd | nd | nd | 111 | 54 | 55 | 15 | 258 | 188 | 112 | 84 | 114 | 188 | 168 | 379 | 1765 | 1 |
| Site 2 | 313 | 388 | nd | nd | 539 | 215 | 46 | 22 | 111 | 161 | 111 | 91 | 176 | 118 | 147 | 175 | 2613 |  |
| Site 3 | 46 | nd | nd | nd | 81 | 23 | 25 | 7 | 51 | 79 | 34 | 25 | 38 | 85 | 65 | 73 | 632 |  |
| Site 4 | 296 | 227 | 126 | 85 | 145 | 91 | 56 | 13 | 76 | 282 | 58 | 42 | 46 | 139 | 158 | 185 | 2025 |  |
| Site 5 | 397 | 437 | nd | 232 | 516 | 343 | 72 | 15 | 91 | 289 | 144 | 116 | 186 | 156 | 316 | nd | 3310 |  |
| Site 6 | 442 | 371 | nd | 196 | 236 | 123 | 88 | 23 | 111 | 311 | 151 | 111 | 135 | 147 | 241 | 149 | 2835 |  |
| Site 7 | 244 | 287 | nd | 111 | 191 | 82 | 89 | 33 | 198 | 411 | 151 | 111 | 159 | 149 | 241 | 215 | 2672 |  |
| Site 8 | 333 | 269 | nd | 89 | 126 | 67 | 61 | 19 | 111 | 221 | 147 | 75 | 123 | 131 | 148 | 173 | 2093 |  |
| Site 9 | 417 | 224 | nd | 116 | 185 | 79 | 79 | 23 | 189 | 416 | 156 | 114 | 181 | 216 | 157 | 228 | 2780 |  |
| Site 10 | 374 | 584 | nd | 93 | 241 | 113 | 77 | 19 | 198 | 395 | 98 | 69 | 23 | 265 | 126 | nd | 2675 |  |
| Site 11 | 317 | 417 | 386 | 312 | 856 | 631 | 119 | 31 | 436 | 319 | 155 | 88 | 94 | 139 | 136 | 388 | 4824 |  |
| Site 12 | 291 | 359 | 326 | 245 | 587 | 514 | 91 | 26 | 362 | 259 | 137 | 64 | 83 | 122 | 174 | 349 | 3989 |  |
| Site 13 | 359 | 344 | 333 | 211 | 488 | 481 | 99 | 27 | 347 | 264 | 142 | 41 | 81 | 119 | 251 | 332 | 3919 |  |
| Site 14 | 359 | 344 | 132 | 93 | 291 | 288 | 99 | 17 | 147 | 263 | 42 | 41 | 11 | 118 | 251 | 211 | 2707 |  |
| Site 15 | 288 | 414 | 264 | 111 | 288 | 213 | 81 | 22 | 398 | 412 | 71 | 46 | 56 | 145 | 199 | 193 | 3201 |  |
| Site 16 | 399 | 318 | 254 | 199 | 315 | 234 | 69 | 21 | 331 | 328 | 57 | 43 | 67 | 173 | 287 | 212 | 3307 |  |
| Site 17 | 345 | 316 | 231 | 113 | 287 | 267 | 74 | 21 | 488 | 342 | 66 | 65 | 51 | 167 | 215 | 146 | 3194 |  |
| Site 18 | 213 | 366 | 176 | 118 | 248 | 246 | 69 | 22 | 519 | 358 | 49 | 56 | 41 | 151 | 192 | 175 | 2999 |  |
| Site 19 | 296 | 426 | 126 | 85 | 291 | 249 | 56 | 13 | 745 | 382 | 58 | 42 | 46 | 133 | 145 | 215 | 3308 |  |
| Site 20 | 343 | 238 | 216 | 189 | 318 | 376 | 56 | 29 | 611 | 415 | 62 | 55 | 49 | 141 | 231 | 167 | 3496 |  |
| Site 21 | 517 | 98 | 361 | 362 | 514 | 624 | 115 | 26 | 585 | 515 | 121 | 75 | 79 | 168 | 431 | 235 | 4826 |  |
| Site 22 | 233 | 217 | 291 | 238 | 258 | 387 | 88 | 57 | 565 | 456 | 137 | 97 | 114 | 192 | 354 | 188 | 3872 |  |
| Site 23 | 257 | 343 | 422 | 567 | 515 | 311 | 136 | 33 | 856 | 641 | 214 | 128 | 151 | 257 | 355 | 348 | 5534 |  |
| Site 24 | 311 | 313 | 291 | 212 | 249 | 181 | 96 | 45 | 613 | 423 | 114 | 113 | 98 | 197 | 326 | 267 | 3849 |  |
| Site 25 | 341 | 73 | 139 | 111 | 235 | 97 | 244 | 57 | 1175 | 811 | 395 | 231 | 521 | 314 | 579 | 486 | 5809 |  |
| Site 26 | 421 | 183 | 979 | 319 | 1413 | 984 | 568 | 499 | 535 | 532 | 439 | 447 | 114 | nd | 313 | 444 | 8190 |  |
| Site 27 | 574 | 218 | 1235 | 427 | 1679 | 1439 | 745 | 722 | 899 | 776 | 581 | 651 | 175 | nd | 424 | 514 | 11059 |  |
| Site 28 | 711 | 352 | 1963 | 598 | 2218 | 1877 | 988 | 944 | 1158 | 841 | 748 | 843 | 216 | nd | 624 | 724 | 14805 |  |
| Site 29 | 512 | 274 | 1161 | 415 | 1988 | 1561 | 878 | 811 | 924 | 879 | 541 | 776 | 197 | nd | 453 | 669 | 12039 |  |
| Site 30 | 311 | 226 | 1387 | 351 | 1819 | 1327 | 779 | 744 | 859 | 774 | 649 | 542 | 175 | nd | 326 | 623 | 10892 |  |
| Site 31 | 436 | 258 | 1256 | 212 | 925 | 899 | 666 | 564 | 922 | 779 | 479 | 621 | 126 | nd | 319 | 625 | 9087 |  |
| Site 32 | 535 | 221 | 989 | 311 | 1721 | 968 | 574 | 444 | 528 | 678 | 397 | 464 | 89 | nd | 235 | 579 | 8733 |  |
| Site 33 | 457 | 198 | nd | 298 | 1749 | 1474 | 542 | 386 | 396 | 513 | 315 | 422 | 115 | nd | 211 | 541 | 7617 |  |
| Site 34 | 576 | 143 | 715 | 198 | 1764 | 989 | 479 | 424 | 376 | 514 | 413 | 399 | 138 | nd | 222 | 578 | 7928 |  |
| Site 35 | 518 | 168 | 714 | 337 | 1386 | 948 | 647 | 463 | 499 | 582 | 322 | 417 | 115 | nd | 337 | 487 | 7940 |  |
| Site 36 | 614 | 175 | 1471 | 318 | 1376 | 1138 | 538 | 529 | 463 | 514 | 369 | 413 | 131 | nd | 315 | 521 | 8885 |  |
| Site 37 | 158 | 44 | 33 | 71 | 91 | 54 | 49 | 17 | 37 | 64 | 19 | 41 | 11 | 19 | 51 | nd | 759 |  |
| Site 38 | 113 | 16 | 53 | 69 | 79 | 35 | 38 | 11 | 24 | 29 | 12 | 31 | 24 | 11 | 31 | 39 | 615 |  |
| Site 39 | 67 | 11 | 35 | 69 | 52 | 31 | 24 | 22 | 17 | 25 | 13 | 32 | 18 | nd | 18 | 42 | 476 |  |
| Site 40 | 71 | 13 | 46 | 73 | 67 | 26 | 34 | 19 | 23 | 35 | 11 | 29 | 27 | nd | 22 | 57 | 553 |  |
| Site 41 | 54 | 15 | 21 | 49 | 37 | 21 | 21 | 11 | 21 | 25 | 8 | 11 | 29 | 11 | 13 | 25 | 372 |  |
| Site 42 | 87 | 16 | 38 | 79 | 77 | 32 | 29 | 14 | 21 | 31 | 16 | 32 | 29 | 8 | 29 | 61 | 599 |  |
| Site 43 | 176 | 66 | 68 | 82 | 144 | 81 | 43 | 21 | 41 | 32 | 24 | 36 | 24 | 11 | 49 | 55 | 953 |  |
| Site 44 | 96 | 12 | 48 | 59 | 68 | 27 | 35 | 18 | 19 | 23 | 11 | 21 | 23 | 11 | 21 | 56 | 548 |  |
| Site 45 | 96 | 11 | 71 | 89 | 54 | 54 | 36 | 16 | 21 | 37 | 16 | 25 | 23 | 14 | 45 | 1 | 609 |  |
| Site 46 | 114 | 15 | 65 | 72 | 81 | 32 | 31 | 19 | 24 | 31 | 17 | 29 | 29 | 8 | 37 | 57 | 661 |  |
| Site 47 | 111 | 22 | 33 | 88 | 71 | 33 | 41 | 29 | 28 | 47 | 11 | 17 | 47 | nd | 28 | 67 | 673 |  |
| Site 48 | 37 | 9 | 21 | 31 | 48 | 16 | 12 | 9 | 19 | 18 | 6 | nd | 21 | nd | 7 | 25 | 279 |  |
| Site 49 | 58 | 13 | 32 | 41 | 57 | 22 | 29 | 12 | 21 | 29 | 8 | 7 | 35 | nd | 12 | 33 | 409 |  |
| Site 50 | 31 | 7 | 15 | 25 | 34 | 8 | 11 | 8 | 8 | 11 | 4 | nd | 15 | nd | 5 | 11 | 193 |  |
| Site 51 | 49 | 9 | 26 | 38 | 41 | 18 | 27 | 13 | 15 | 21 | 6 | nd | 21 | nd | 11 | 21 | 316 |  |
| Site 52 | 61 | 11 | 31 | 51 | 59 | 18 | 31 | 18 | 19 | 29 | 9 | nd | 21 | nd | 8 | 38 | 404 |  |
| Site 53 | 127 | 18 | 59 | 98 | 79 | 31 | 41 | 35 | 31 | 51 | 12 | 7 | 39 | nd | 31 | 71 | 730 |  |
| Site 54 | 86 | 14 | 27 | 91 | 67 | 23 | 33 | 22 | 21 | 38 | 11 | 15 | 34 | nd | nd | 46 | 528 |  |
| Site 55 | 77 | 15 | 28 | 67 | 56 | 19 | 28 | 26 | 21 | 33 | 9 | 17 | 29 | nd | nd | 52 | 477 |  |
| Site 56 | 185 | nd | 53 | 71 | 91 | 13 | 18 | 13 | 24 | 34 | 9 | nd | 47 | nd | 28 | 54 | 640 |  |
| Site 57 | 115 | 9 | 24 | 45 | 141 | 31 | 17 | 13 | 19 | 31 | 31 | nd | 24 | 9 | nd | 128 | 637 |  |
| Site 58 | 119 | 13 | 62 | 71 | 133 | 29 | 29 | 5 | 14 | 11 | nd | 33 | nd | nd | 25 | 17 | 562 |  |
| Site 59 | 126 | 7 | 59 | 68 | 134 | 21 | 14 | 8 | 21 | 15 | nd | 24 | nd | nd | 38 | 41 | 577 |  |
| Site 60 | 192 | nd | 179 | 91 | 166 | 73 | 33 | 13 | 83 | nd | 33 | 31 | 312 | nd | 115 | 31 | 1352 |  |
| Site 61 | 149 | nd | 31 | 51 | 119 | 13 | 8 | nd | 6 | 41 | 9 | nd | 11 | nd | nd | 38 | 477 |  |
| Site 62 | 127 | 7 | 59 | 69 | 79 | 31 | 11 | 7 | 18 | 13 | nd | 36 | nd | nd | 31 | 71 | 560 |  |
| Site 63 | 157 | 7 | 157 | 131 | 216 | 85 | 97 | 17 | 12 | 38 | 26 | nd | 43 | 8 | nd | 191 | 1185 |  |
| Site 64 | 123 | 7 | 59 | 67 | 155 | 34 | 21 | 21 | 13 | 13 | nd | 32 | nd | nd | 31 | 16 | 592 |  |
| Site 65 | 229 | 37 | 225 | 161 | 358 | 96 | 126 | 33 | 11 | 53 | nd | 35 | nd | nd | 81 | 133 | 1578 |  |
| Site 1 | 0.45 | 20.5 | 68.7 | 2 | 6.6 | 7.2 | nd | 4.8 | 11.8 | 2.4 | 2.5 | 1.4 | 2.8 | 2.2 | 2.1 | 6.1 | 141.55 | 26 |
| Site 2 | 0.45 | 10.4 | 42.6 | 2.3 | 5.2 | 11.7 | 2.5 | 7.4 | 7.7 | 2.6 | 1.4 | 2.9 | 1.7 | 2.6 | 0.9 | 5.7 | 108.05 |  |
| Site 3 | 0.45 | 8.9 | 25.6 | 2.5 | 9.3 | 10.9 | 1.4 | 9.2 | 8 | 5 | 5.4 | 1.8 | 6.2 | 4.1 | 4.8 | 6.9 | 110.45 |  |
| Site 1 | 37.1 | nd | 1.73 | 14.2 | 79.9 | 10.1 | 121 | 102 | 54.4 | 63.3 | 102 | 30.2 | 57.3 | 163 | 6.2 | 63.8 | 906.23 | 27 |
| Site 2 | 25.3 | nd | 1.44 | 10.8 | 39.4 | 3.28 | 43.2 | 37.5 | 22 | 22.8 | 48.6 | 13.9 | 25.4 | 81.7 | 3.13 | 33.8 | 412.25 |  |
| Site 3 | 41 | nd | 1.53 | 14.3 | 49.1 | 2.9 | 32.5 | 22.6 | 15.1 | 12 | 50.9 | 11.6 | 13.6 | 83.9 | 2.37 | 36.2 | 389.6 |  |
| Site 4 | 35.7 | nd | 1.13 | 12.9 | 43.1 | 2.64 | 25.1 | 17.4 | 11 | 9.18 | 26.6 | 6.2 | 8.47 | 40.3 | 1.38 | 17 | 258.1 |  |
| Site 5 | 31.6 | nd | 1.81 | 14 | 69.9 | 4.6 | 94.8 | 75.4 | 45.3 | 42.8 | 123 | 32.5 | 47 | 188 | 7.76 | 85.6 | 864.07 |  |
| Site 6 | 27 | nd | 1.92 | 13.7 | 78.1 | 6.42 | 87.9 | 71.6 | 40.2 | 46.5 | 84.7 | 23.5 | 40.2 | 142 | 4.96 | 52.2 | 720.9 |  |
| Site 7 | 34.2 | nd | 1.03 | 10.1 | 40.2 | 2.26 | 29.6 | 23 | 15.1 | 11.5 | 46.1 | 11.1 | 12.6 | 78 | 1.73 | 35.1 | 351.62 |  |
| Site 1 | 186.09 | 59.62 | 100.3 | 46.69 | 51.44 | 48.7 | 108.77 | 72.34 | 10.3 | 9.49 | 3.47 | 0.15 | 0.79 | 8.97 | 12.55 | 55.12 | 774.77 | 28 |
| Site 2 | 296.21 | 70.18 | 30.02 | 77.21 | 44.32 | 53.2 | 165.61 | 52.85 | 12.27 | 9.47 | 0.82 | 0.65 | 1.75 | 14.52 | 16.34 | 13.62 | 859.04 |  |
| Site 3 | 306.47 | 88.22 | 14.46 | 81.13 | 52.16 | 48.26 | 165.61 | 52.85 | 12.27 | 9.47 | 0.82 | 0.65 | 0.36 | 9.11 | 7.51 | 8.45 | 857.8 |  |
| Site 4 | 845.31 | 227.01 | 280.5 | 906.3 | 1062.44 | 983.09 | 1522.74 | 967.02 | 67.67 | 56.6 | 25.02 | 10.75 | 11.56 | 16.74 | 25.55 | 95.74 | 7104.04 |  |
| Site 5 | 1302.51 | 242.49 | 55.91 | 572.12 | 186.2 | 78.95 | 1065.49 | 683.45 | 42.36 | 26.02 | 21.52 | 11.51 | 8.99 | 13.27 | 27.26 | 85.33 | 4423.38 |  |
| Site 6 | 235.12 | 132.14 | 47.56 | 25.65 | 80.53 | 38.52 | 134 | 79.11 | 6.51 | 4.02 | 2.46 | 6.23 | 4.51 | 10.12 | 18.02 | 62.35 | 886.85 |  |
| Site 1 | 1230 | 63.1 | 147 | 527 | 1520 | 192 | 1240 | 1560 | 266 | 620 | 257 | 239 | 250 | 387 | 62.1 | 102 | 8662.2 | 29 |
| Site 2 | 720 | 45.5 | 92.5 | 374 | 1230 | 239 | 968 | 1260 | 196 | 205 | 212 | 197 | 221 | 273 | 11.5 | 51.5 | 6296 |  |
| Site 3 | 812 | 39.1 | 104 | 382 | 1150 | 110 | 757 | 987 | 119 | 367 | 161 | 171 | 115 | 229 | 17.5 | 41.5 | 5562.1 |  |
| Site 4 | 274 | 22.5 | 89.1 | 210 | 684 | 149 | 783 | 788 | 281 | 430 | 298 | 281 | 139 | 268 | 20.5 | 68.1 | 4785.2 |  |
| Site 5 | 776 | 50.1 | 132 | 406 | 1310 | 308 | 1320 | 1650 | 335 | 726 | 409 | 480 | 144 | 397 | 21.5 | 96.5 | 8561.1 |  |
| Site 6 | 516 | 27.1 | 77.1 | 226 | 955 | 182 | 1060 | 1300 | 492 | 867 | 768 | 716 | 350 | 756 | 59.1 | 237 | 8588.3 |  |
| Site 1 | 4.08 | 3.65 | 45.85 | 18.38 | 80.46 | 5.75 | 17.15 | 17.66 | 5.44 | 11.12 | 5.35 | 4.36 | 3.36 | nd | 6.96 | 4.68 | 234.25 | 30 |
| Site 2 | 1.32 | 1.91 | 29.11 | 10.82 | 53.36 | 3.73 | 14.01 | 15.68 | 5.43 | 10.11 | 6.72 | 5.92 | 3.36 | 19.94 | 2.28 | 5.06 | 188.76 |  |
| Site 3 | 0.76 | 1.83 | 35.36 | 15.55 | 67.99 | 6.34 | 17.45 | 19.12 | 6.32 | 10.54 | 7.27 | 6.48 | 4.87 | nd | 4.53 | 5.54 | 209.95 |  |
| Site 4 | 1.81 | 3.03 | 28.92 | 22.66 | 97.24 | 9.89 | 1.48 | 36.72 | 2.56 | 24.45 | 18.97 | 18.4 | 8.3 | 138.57 | 6.73 | 10.72 | 430.45 |  |
| Site 5 | 2.28 | 1.12 | 32.47 | 7.88 | 28.21 | 2.75 | 13.17 | 14.92 | 5.65 | 9.74 | 6.83 | 5.54 | 5.43 | 11.39 | 4.5 | 5.1 | 156.98 |  |
| Site 6 | 0.78 | 4.39 | 25.19 | 26.99 | 122.04 | 23.42 | 2.47 | 53.27 | 21.43 | 31.24 | 20.48 | 19.94 | 12.31 | 142.9 | 5.18 | 10.64 | 522.67 |  |
| Site 7 | 2.18 | 1.29 | 32.17 | 8.41 | 41.44 | 2.12 | 5.84 | 5.9 | 2.15 | 3.73 | 16.16 | 15.54 | 1.96 | 0.045 | 4.81 | 0.81 | 144.555 |  |
| Site 8 | 0.81 | 2.2 | 25.05 | 9.4 | 44.32 | 5.58 | 24.66 | 29.15 | 10.78 | 16.56 | 13.06 | 11.12 | 8.92 | 155.84 | 5.7 | 11.78 | 374.93 |  |
| Site 9 | 3.93 | 3.19 | 57.19 | 26.05 | 127.16 | 25.22 | 1.15 | 36.52 | 13.15 | 20.79 | 6.22 | 5.36 | 5.62 | nd | 8.45 | 5.11 | 345.11 |  |
| Site 10 | 2.41 | 2.58 | 36.41 | 9.89 | 37.86 | 2.53 | 12.42 | 12.65 | 4.5 | 8.6 | 4.57 | 3.7 | 1.88 | nd | 3.44 | 0.46 | 143.9 |  |
| Site 11 | 0.09 | 2 | 26.07 | 6.04 | 30.79 | 2.99 | 6.27 | 7.1 | 2.17 | 4.11 | 26.05 | 25.58 | 105 | nd | 3.98 | 0.51 | 248.75 |  |
| Site 12 | 0.75 | 4.86 | 30.88 | 15.93 | 78.79 | 6.43 | 36.08 | 41.33 | 14.96 | 22.61 | 18.59 | 17.65 | 10.39 | 184.49 | 4.74 | 12.55 | 501.03 |  |
| Site 13 | 1.15 | 4.51 | 41.46 | 20.15 | 71.53 | 10.37 | 27.87 | 30.62 | 8.7 | 18.28 | 10.67 | 10.54 | 5.25 | nd | 2.2 | 6.89 | 270.19 |  |
| Site 14 | 2.25 | 2.78 | 39.28 | 11.85 | 53.52 | 4.52 | 12.07 | 11.8 | 4.02 | 7.45 | 4.03 | 3.09 | 1.65 | nd | 3.17 | 0.47 | 161.95 |  |
| Site 15 | 1.08 | 3.08 | 31.28 | 13.98 | 63.18 | 7.54 | 28.57 | 33.57 | 11.41 | 19.34 | 15.25 | 14.58 | 7.98 | 62.39 | 3.42 | 11.47 | 328.12 |  |
| Site 16 | 1.27 | 2.13 | 29.98 | 13.3 | 66.42 | 4.73 | 13.79 | 12.44 | 4.12 | 6.32 | 4.86 | 3.89 | 2.55 | nd | 4.6 | 3.17 | 173.57 |  |
| Site 17 | 1.54 | 2.56 | 32.24 | 20.47 | 86.13 | 5.45 | 0.93 | 24.1 | 7.64 | 16.96 | 11.45 | 10.64 | 4.33 | 123.23 | 5.38 | 7.11 | 360.16 |  |
| Site 18 | 1.13 | 2.45 | 35.13 | 14.57 | 66.29 | 5.49 | 20.59 | 1.01 | 9.2 | 15.19 | 14.73 | 14.09 | 6.71 | 138.89 | 5.51 | 12.75 | 363.73 |  |
| Site 19 | 2.69 | 2.2 | 26.66 | 13.42 | 65.57 | 2.89 | 9.68 | 10.7 | 2.87 | 4.59 | 57.87 | 57.81 | 1.48 | nd | 5.42 | 0.61 | 264.46 |  |
| Site 20 | 8.74 | 0.05 | 3.18 | 3.14 | 12.51 | 0.26 | 11.55 | 10.53 | 2.40 | 6.38 | 9.48 | 4.74 | 7.49 | 8.26 | 1.24 | 9.41 | 99.36 |  |
| Site 1 | 25.03 | 2.05 | 2.12 | 16.56 | 34.64 | 1.59 | 3.36 | 1.62 | 2.57 | 1.52 | 1.14 | 0.98 | 2.15 | 1.00 | 0.50 | 3.13 | 99.96 | 31 |
| Site 2 | 30.88 | 5.15 | 2.40 | 6.88 | 20.35 | 1.99 | 9.02 | 7.02 | 2.99 | 3.52 | 1.13 | 1.14 | 2.13 | 2.62 | 0.20 | 2.54 | 99.96 |  |
| Site 3 | 43.58 | 4.46 | 2.18 | 10.32 | 22.08 | 0.88 | 2.10 | 6.14 | 1.59 | 0.82 | 0.34 | 0.79 | 0.99 | 1.75 | 0.10 | 1.79 | 99.91 |  |
| Site 4 | 31.68 | 3.97 | 2.44 | 12.77 | 29.52 | 1.38 | 4.60 | 2.63 | 1.53 | 1.72 | 0.35 | 0.60 | 2.56 | 1.87 | 0.17 | 2.27 | 100.06 |  |
| Site 5 | 28.54 | 3.01 | 1.79 | 14.88 | 33.54 | 1.43 | 4.64 | 3.89 | 1.52 | 1.60 | 0.19 | 0.53 | 1.49 | 1.18 | 0.19 | 1.74 | 100.16 |  |
| Site 6 | 32.76 | 2.93 | 2.50 | 9.63 | 33.05 | 1.28 | 4.97 | 3.84 | 2.29 | 1.71 | 0.53 | 0.45 | 1.06 | 0.70 | 0.13 | 2.18 | 100.01 |  |
| Site 7 | 35.75 | 3.41 | 2.28 | 12.80 | 27.60 | 0.90 | 3.10 | 3.43 | 0.90 | 1.06 | 0.32 | 1.96 | 3.84 | 1.45 | 0.10 | 1.09 | 99.99 |  |
| Site 8 | 9.55 | 0.79 | 2.32 | 18.46 | 48.51 | 1.72 | 6.43 | 3.95 | 1.34 | 1.54 | 0.61 | 0.60 | 1.05 | 1.09 | 0.11 | 1.79 | 99.86 |  |
| Site 9 | 26.05 | 2.13 | 2.74 | 17.99 | 31.98 | 1.70 | 4.84 | 3.54 | 1.85 | 1.34 | 1.00 | 0.47 | 0.93 | 1.03 | 0.11 | 2.24 | 99.94 |  |
| Site 10 | 37.35 | 3.21 | 2.96 | 11.61 | 26.59 | 2.58 | 5.21 | 3.47 | 1.56 | 1.46 | 0.59 | 0.46 | 1.12 | 1.08 | 0.17 | 1.32 | 100.74 |  |
| Site 1 | 23.74 | nd | nd | nd | 0.97 | 1.70 | 0.16 | 0.95 | 2.02 | 0.07 | 0.60 | nd | 0.07 | 0.18 | 0.13 | 0.14 | 30.73 | 32 |
| Site 2 | 27.71 | 0.26 | nd | 0.22 | 1.21 | 2.51 | 0.15 | 2.04 | 3.47 | 1.13 | 6.54 | nd | 13.28 | 0.11 | 0.08 | 1.51 | 60.22 |  |
| Site 3 | 28.49 | nd | nd | nd | 1.61 | 3.81 | nd | 4.48 | 5.12 | 2.17 | 9.36 | nd | 15.11 | 2.28 | nd | 3.24 | 75.67 |  |
| Site 4 | 41.80 | 5.26 | 19.63 | 3.29 | 63.47 | 8.13 | 2.71 | 1.93 | 2.56 | 0.66 | 3.62 | nd | 5.40 | 1.38 | nd | 1.96 | 161.8 |  |
| Site 5 | 628.68 | 5.55 | nd | 8.81 | 29.15 | 63.57 | 5.83 | 50.38 | 76.50 | 38.10 | 159.71 | nd | 284.07 | 4.32 | 3.34 | 51.14 | 1409.15 |  |
| Site 6 | nd | 2.16 | nd | nd | 7.29 | 12.83 | 1.77 | 14.92 | 13.88 | 8.98 | 23.07 | nd | 7.89 | 7.86 | 1.01 | 12.14 | 113.8 |  |
| Site 7 | 49.97 | 2.01 | nd | 1.90 | 5.79 | 24.33 | 4.13 | 35.57 | 31.43 | 16.27 | 15.95 | 23.40 | 19.02 | 19.87 | 2.75 | 31.60 | 283.99 |  |
| Site 8 | 119.01 | 4.23 | nd | nd | 9.20 | 26.98 | 0.01 | 28.56 | 32.34 | 9.31 | 27.30 | nd | 13.09 | 11.52 | nd | 0.07 | 281.62 |  |
| Site 9 | 127.76 | nd | nd | 10.22 | 35.67 | 35.65 | 4.94 | 40.99 | 47.66 | 18.15 | 49.67 | nd | 15.64 | 9.51 | nd | 5.79 | 401.65 |  |
| Site 10 | 16.17 | nd | nd | nd | 5.43 | 35.66 | 15.82 | 47.21 | 35.56 | 15.33 | 19.18 | 9.78 | 10.07 | 6.49 | nd | 8.25 | 224.95 |  |
| Site 11 | 23.12 | 0.81 | nd | 1.64 | 5.07 | 16.92 | 1.82 | 18.28 | 18.98 | 7.53 | 16.36 | 8.00 | 7.16 | 7.56 | 0.75 | 10.22 | 144.22 |  |
| Site 12 | 75.83 | nd | nd | nd | 7.03 | 21.72 | 3.43 | 35.33 | 34.30 | 19.18 | 85.70 | 28.53 | 20.34 | 25.77 | 3.88 | 33.91 | 394.95 |  |
| Site 13 | 49.11 | nd | nd | nd | 8.63 | 35.65 | 5.79 | 46.50 | 40.89 | 19.00 | 61.10 | 22.09 | 23.25 | 15.13 | nd | 24.96 | 352.1 |  |
| Site 1 | 4.6 | 4.3 | 5.2 | 11.2 | 85.8 | 11.9 | 54.2 | 48.9 | 5.3 | 10.7 | 1.9 | 0.8 | 0.7 | 1.4 | nd | 2.1 | 249 | 33 |
| Site 2 | 4.9 | 4.7 | 6.8 | 11.8 | 89 | 12.4 | 64.9 | 53.6 | 6.6 | 9.9 | 8.5 | 9.5 | 3.6 | 1.8 | nd | 1.2 | 289.2 |  |
| Site 3 | 5.1 | 3.4 | 7.6 | 15.5 | 135.7 | 20.7 | 190.8 | 130.2 | 5 | 9.1 | 7.9 | 6.4 | 3.2 | 1.6 | nd | 1.5 | 543.7 |  |
| Site 4 | 3 | 2.2 | 4.2 | 8.8 | 106.4 | 16.4 | 167.3 | 152.4 | 4.9 | 8.9 | 0.5 | 0.7 | 0.9 | 2.1 | nd | 1.6 | 480.3 |  |
| Site 5 | 6.3 | 6.4 | 6.4 | 20 | 209.2 | 20.3 | 386.5 | 291.3 | 10.5 | 12.3 | 17.6 | 9.5 | 3.4 | 3.7 | nd | 4.3 | 1007.7 |  |
| Site 6 | 6.7 | 5.6 | 7.2 | 16.8 | 242.8 | 20.3 | 236.6 | 226.3 | 6.4 | 18.7 | 14.8 | 13.8 | 8.5 | 5.3 | nd | 4.6 | 834.4 |  |
| Site 7 | 1.5 | 1.4 | 4.4 | 11.5 | 81.8 | 7.8 | 72.2 | 56.1 | 7.5 | 1.2 | 5.5 | 6.5 | 4.2 | 0.8 | nd | 1 | 263.4 |  |
| Site 8 | 4.6 | 3.2 | 6.1 | 12 | 126.8 | 19.8 | 191.3 | 112.5 | 4.2 | 10.3 | 4.9 | 4.1 | 3.5 | 1.1 | nd | 0.9 | 505.3 |  |
| Site 9 | 3.7 | 2.4 | 4.3 | 9.7 | 98.5 | 15.8 | 178.1 | 99.9 | 2.6 | 9.4 | 4 | 3.6 | 2.9 | 0.9 | nd | 0.6 | 436.4 |  |
| Site 10 | 2.4 | 1.2 | 3 | 12.9 | 72.4 | 20.1 | 65.8 | 43.9 | 7.8 | 11.1 | 5.4 | 5 | 4.3 | 0.5 | nd | 0.1 | 255.9 |  |
| Site 11 | 3 | 3.4 | 5.6 | 13.5 | 78.8 | 22.2 | 82.9 | 51.6 | 10.6 | 13.4 | 5.9 | 6.3 | 6.1 | 2.6 | nd | 0.4 | 306.3 |  |
| Site 12 | 2.3 | 1.2 | 2.9 | 13.6 | 39.2 | 4 | 14.2 | 10.8 | 1.2 | 0.7 | 0.6 | 0.3 | 0.2 | 0.1 | nd | 0.1 | 91.4 |  |
| Site 13 | 2 | 1.4 | 4.2 | 11.8 | 88.6 | 9.6 | 78.4 | 57.4 | 5.8 | 9.4 | 5.3 | 4.9 | 3.8 | 0.2 | nd | 0.6 | 283.4 |  |
| Site 14 | 1.8 | 1.6 | 3.9 | 12.5 | 89.8 | 9.9 | 78.4 | 58.1 | 6.7 | 10.2 | 5.7 | 5.5 | 4.1 | 0.6 | nd | 1 | 289.8 |  |
| Site 15 | 1.3 | 1.6 | 3.2 | 9.4 | 54.6 | 19.5 | 4.6 | 23.3 | 1.4 | 0.8 | 0.9 | 0.4 | 0.7 | 0.1 | nd | 0.1 | 121.9 |  |
| Site 16 | 1.3 | 1.9 | 3.6 | 9.6 | 85.9 | 28.6 | 5.4 | 32.8 | 2.8 | 1.3 | 1.7 | 1.1 | 1.6 | 0.5 | nd | 0.3 | 178.4 |  |
| Site 17 | 1.3 | 2 | 3.6 | 9.5 | 95 | 29.7 | 6.3 | 45.5 | 4.7 | 1.1 | 2.1 | 1.4 | 2.3 | 0.8 | nd | 0.7 | 206 |  |
| Site 18 | 1.6 | 1.5 | 3.1 | 5.4 | 3.6 | 1.3 | 3.7 | 2.9 | 1.8 | 1.1 | 0.6 | 0.4 | 0.2 | 0.1 | nd | 0.1 | 27.4 |  |
| Site 19 | 2 | 1.7 | 3.4 | 5.6 | 3.7 | 1.4 | 4.2 | 3.7 | 1.6 | 1.2 | 0.7 | 0.3 | 0.3 | 0.2 | nd | 0.1 | 30.1 |  |
| Site 1 | 5.00 | 0.90 | 3.50 | 7.50 | 43.00 | 14.80 | 56.40 | 41.60 | 18.10 | 25.10 | 21.80 | 21.90 | 24.80 | 23.80 | 3.30 | 20.30 | 331.8 | 10 |
| Site 2 | 19.30 | 1.50 | 0.90 | 10.80 | 26.50 | 1.90 | 8.90 | 8.10 | 4.00 | 10.50 | 8.40 | 7.60 | 6.00 | 5.20 | 1.00 | 9.00 | 129.6 |  |
| Site 3 | 21.70 | 3.30 | 2.30 | 16.70 | 46.30 | 7.00 | 26.50 | 19.10 | 9.30 | 15.90 | 17.20 | 14.70 | 12.20 | 12.60 | 1.80 | 11.60 | 238.2 |  |
| Site 4 | 10.40 | 1.10 | 0.80 | 6.70 | 27.30 | 2.50 | 17.20 | 13.90 | 6.10 | 12.80 | 13.10 | 10.50 | 7.30 | 8.10 | 1.10 | 9.30 | 148.2 |  |
| Site 5 | 22.70 | 1.80 | 2.00 | 11.40 | 39.40 | 2.60 | 23.30 | 16.60 | 9.00 | 23.50 | 23.50 | 17.80 | 13.80 | 20.50 | 2.90 | 18.60 | 249.4 |  |
| Site 6 | 33.30 | 7.40 | 4.20 | 31.00 | 84.00 | 15.80 | 59.60 | 46.80 | 21.70 | 37.30 | 40.60 | 27.80 | 28.60 | 29.20 | 5.20 | 31.90 | 504.4 |  |
| Site 7 | 14.70 | 1.80 | 1.70 | 24.80 | 39.70 | 3.70 | 39.60 | 24.00 | 12.20 | 24.80 | 58.60 | 41.50 | 15.30 | 43.50 | 5.50 | 34.30 | 385.7 |  |
| Site 8 | 20.60 | 4.90 | 2.40 | 23.60 | 68.60 | 6.40 | 46.10 | 29.90 | 14.80 | 38.80 | 61.00 | 33.80 | 25.50 | 57.80 | 10.00 | 48.50 | 492.7 |  |
| Site 9 | 87.10 | 5.10 | 21.70 | 40.30 | 151.10 | 28.70 | 154.40 | 124.90 | 71.90 | 102.40 | 177.80 | 109.70 | 152.30 | 180.80 | 33.80 | 143.80 | 1585.8 |  |
| Site 1 | 85.83 | 29.43 | 166.0 | 190.08 | 52.96 | 270.98 | 5.17 | 32.65 | 26.14 | 17.22 | 12.49 | 41.36 | 5.52 | 16.71 | 4.13 | 20.25 | 976.91 | 34 |
| Site 2 | 74.45 | 61.36 | 139.68 | 276.63 | 47.11 | 413.07 | 78.27 | 460.23 | 279.37 | 183.55 | 253.75 | 233.86 | 95.05 | 42.9 | 5.32 | 44.89 | 2689.49 |  |
| Site 3 | 15.7 | 28.2 | 19.73 | 117.32 | 98.52 | 596.46 | 13.06 | 207.38 | 206.27 | 122.04 | 104.51 | 198.87 | 38.49 | 16.75 | 4.22 | 28.96 | 1816.48 |  |
| Site 4 | 53.09 | 26.08 | 196.5 | 426.68 | 47.8 | 205.43 | 21.85 | 449 | 206.2 | 182.18 | 431.69 | 86.36 | 35.09 | 14.06 | 5.19 | 11.2 | 2398.35 |  |
| Site 5 | 20.57 | 46.31 | 12.32 | 107.53 | 48.54 | 74.52 | 3.38 | 31.22 | 14.45 | 5.18 | 5.98 | 33.53 | 17.32 | 16.83 | 4.83 | 20.4 | 462.91 |  |
| Site 1 | 295 | 8.27 | 58.1 | 73.4 | 296 | 51.3 | 441 | 455 | 188 | 231 | 174 | 174 | 165 | 165 | 32.2 | 248 | 3055.27 | 35 |
| Site 2 | 34.9 | 14.6 | 47 | 77.9 | 282 | 60.2 | 427 | 386 | 211 | 242 | 202 | 178 | 201 | 184 | 29 | 264 | 2840.6 |  |
| Site 3 | 42.5 | 5.89 | 33.3 | 51.4 | 166 | 38.1 | 246 | 215 | 133 | 153 | 141 | 104 | 104 | 113 | 14.8 | 130 | 1690.99 |  |
| Site 4 | 51.4 | 14.8 | 50.3 | 86.9 | 450 | 114 | 1060 | 880 | 562 | 639 | 682 | 490 | 544 | 516 | 37.8 | 625 | 6803.2 |  |
| Site 5 | 97.5 | 18.9 | 300 | 250 | 465 | 86.8 | 677 | 440 | 291 | 320 | 333 | 202 | 200 | 201 | 33.6 | 244 | 4159.8 |  |
| Site 6 | 29.3 | 4.52 | 33.8 | 42.4 | 183 | 35.3 | 316 | 233 | 179 | 184 | 202 | 142 | 143 | 175 | 22.9 | 168 | 2093.22 |  |
| Site 7 | 56.8 | 6.99 | 30.3 | 51.1 | 225 | 44.4 | 349 | 283 | 207 | 248 | 2810 | 184 | 173 | 245 | 29.1 | 255 | 5197.69 |  |
| Site 8 | 56 | 5.99 | 41.8 | 79.4 | 302 | 58.9 | 552 | 414 | 264 | 337 | 326 | 254 | 219 | 251 | 24 | 296 | 3481.09 |  |
| Site 9 | 0 | 5.08 | 26.2 | 61 | 424 | 79.7 | 1070 | 705 | 546 | 594 | 670 | 455 | 467 | 529 | 104 | 704 | 6439.98 |  |
| Site 10 | 27.9 | 3.36 | 17.7 | 33.9 | 279 | 37.8 | 688 | 518 | 361 | 403 | 437 | 323 | 310 | 375 | 66.4 | 500 | 4381.06 |  |
| Site 11 | 135 | 1.34 | 4.63 | 16.5 | 41.8 | 4.42 | 88.6 | 61.7 | 32 | 46.9 | 48.4 | 34.2 | 22.8 | 41.1 | 6.72 | 60.9 | 647.01 |  |
| Site 12 | 0 | 0.34 | 1.51 | 5.26 | 21.7 | 1.43 | 17.9 | 15.4 | 8.08 | 13.2 | 8.76 | 6.15 | 3.85 | 7.17 | 0.05 | 7.75 | 118.55 |  |
| Site 13 | 138 | 2.43 | 28.1 | 28.6 | 142 | 20.5 | 270 | 209 | 130 | 158 | 165 | 109 | 109 | 135 | 25.1 | 182 | 1851.73 |  |
| Site 14 | 189 | 6.3 | 26.4 | 46.8 | 211 | 37.3 | 357 | 343 | 167 | 197 | 185 | 138 | 137 | 162 | 32.3 | 223 | 2458.1 |  |
| Site 15 | 67.5 | 5.06 | 17 | 85 | 483 | 75.3 | 699 | 510 | 389 | 455 | 545 | 300 | 323 | 365 | 76.7 | 585 | 4980.56 |  |
| Site 16 | 65.6 | 4.98 | 11.3 | 33.8 | 194 | 30.2 | 485 | 330 | 256 | 290 | 324 | 228 | 236 | 275 | 51.2 | 359 | 3174.08 |  |
| Site 17 | 39.2 | 1.37 | 18.7 | 71.9 | 142 | 19.3 | 254 | 155 | 116 | 138 | 132 | 102 | 93.9 | 113 | 22.2 | 144 | 1562.57 |  |
| Site 18 | 86.5 | 6.29 | 20 | 45.9 | 224 | 44 | 390 | 320 | 169 | 227 | 203 | 161 | 143 | 195 | 20.5 | 202 | 2457.19 |  |
| Site 1 | 87 | 3.3 | 9.9 | 17.1 | 150.8 | 19.6 | 165.4 | 171.3 | 168.7 | 154.3 | 241.6 | 65.9 | 243.1 | 58.5 | 40 | 128 | 1724.5 | 36 |
| Site 2 | 88.8 | 2.1 | 3.7 | 16.5 | 70 | 9.7 | 47.6 | 45.5 | 33.2 | 37.5 | 67.1 | 16.5 | 22.8 | 7.1 | 10.6 | 34.2 | 512.9 |  |
| Site 3 | 89.3 | 2.3 | 5 | 23.9 | 134.3 | 12.4 | 48.5 | 44.2 | 25.7 | 33.1 | 55.9 | 12.7 | 40.7 | 7.9 | 7.1 | 27.1 | 570.1 |  |
| Site 4 | 60.8 | 3.1 | 12.3 | 23.2 | 132.5 | 30.2 | 113.7 | 104 | 80.7 | 78.8 | 118.6 | 31.1 | 106.7 | 46.7 | 17.5 | 58 | 1017.9 |  |
| Site 5 | 83.4 | 0.75 | 0.75 | 8.1 | 86.8 | 2.3 | 6.1 | 3.6 | 0.75 | 0.75 | 3.1 | 0.75 | 0.75 | 0.75 | 0.75 | 1.5 | 200.9 |  |
| Site 6 | 21.2 | 0.75 | 3.3 | 8.1 | 43.3 | 7.2 | 34.4 | 38.7 | 27 | 29.1 | 46.2 | 10.9 | 40.9 | 4.7 | 6.3 | 25.2 | 347.25 |  |
| Site 7 | 137.8 | 24.9 | 51.1 | 67.4 | 302.6 | 99 | 396.3 | 498.7 | 387.9 | 344.7 | 506.5 | 144.7 | 631.4 | 36.8 | 89.6 | 329.6 | 4049 |  |
| Site 8 | 24 | 0.75 | 0.75 | 3 | 25.3 | 0.75 | 3.7 | 0.75 | 0.75 | 0.75 | 0.75 | 0.75 | 0.75 | 0.75 | 0.75 | 0.75 | 65 |  |
| Site 9 | 108.3 | 8.5 | 20.6 | 30.6 | 154.6 | 35.8 | 136.8 | 160.9 | 121.5 | 110 | 177.3 | 50.5 | 210.1 | 26.6 | 32.2 | 75.8 | 1460.1 |  |
| Site 10 | 100.1 | 5.6 | 14.6 | 31.3 | 113.8 | 29.9 | 110 | 120.9 | 92.4 | 93.4 | 138.7 | 36.9 | 138.6 | 12.2 | 22.2 | 75.8 | 1136.4 |  |
| Site 11 | 94.2 | 0.75 | 2.3 | 13.7 | 110 | 5.9 | 10.8 | 6.4 | 0.75 | 4.2 | 4.1 | 0.75 | 0.75 | 0.75 | 0.75 | 2 | 258.1 |  |
| Site 12 | 62.9 | 2.1 | 4 | 12.7 | 177.2 | 8.5 | 47 | 43 | 34.5 | 34.9 | 51.2 | 13.4 | 45.2 | 14 | 7.7 | 26.1 | 584.4 |  |
| Site 13 | 46 | 0.75 | 2.7 | 15.5 | 40.4 | 5.6 | 20.7 | 17.1 | 10 | 17.8 | 23.3 | 4.3 | 11.5 | 3.3 | 3.3 | 10.2 | 232.45 |  |
| Site 14 | 72.2 | 1.5 | 3.5 | 15.4 | 62.8 | 9.7 | 24.5 | 22.3 | 15.5 | 18.2 | 25.5 | 6.9 | 22.2 | 6.6 | 4 | 13.7 | 324.5 |  |
| Site 1 | 3.8 | 0.35 | 10 | 2.1 | 3.9 | 5.2 | 0.3 | 0.17 | 79.1 | 3.4 | 1.2 | 99.5 | 0.22 | 3.6 | 1.4 | 3.7 | 217.94 | 9 |
| Site 2 | 86.4 | 32.29 | 22.3 | 25.2 | 12 | 16 | 3.5 | 4.27 | 164.1 | 24.4 | 30.9 | 104.8 | 24 | 16 | 16 | 14.4 | 596.56 |  |
| Site 3 | 30.6 | 4.38 | 54.6 | 9.6 | 109.9 | 114.1 | 8.2 | 14.92 | 419.9 | 27.5 | 11.5 | 15 | 9.7 | 24.5 | 13.9 | 9.3 | 877.6 |  |
| Site 4 | 0.5 | 0.35 | 4.4 | 0.39 | 1.7 | 2.1 | 0.3 | 0.17 | 34.5 | 2.7 | 1.2 | 44.9 | 0.22 | 2.3 | 0.19 | 1.6 | 97.52 |  |
| Site 5 | 19 | 0.35 | 14 | 0.39 | 7.1 | 7.4 | 3.2 | 2.14 | 110 | 7.6 | 12.7 | 35.2 | 6.4 | 5.6 | 3.9 | 3.8 | 238.78 |  |
| Site 6 | 0.5 | 0.35 | 7.8 | 1.5 | 2.7 | 3.6 | 2.5 | 1.41 | 55.5 | 8.1 | 11.4 | 34.8 | 9 | 3.5 | 1.2 | 0.22 | 144.08 |  |
| Site 7 | 44.2 | 0.35 | 27.1 | 30.2 | 10.8 | 18.4 | 0.3 | 2.29 | 147.8 | 7 | 2.6 | 125.4 | 0.22 | 5.3 | 2.8 | 4.5 | 429.26 |  |
| Site 8 | 31.5 | 6.83 | 21.6 | 23.2 | 7 | 10.9 | 1.4 | 1.62 | 96.4 | 3.5 | 3.8 | 3.2 | 4.4 | 3.8 | 3.4 | 7.5 | 230.05 |  |
| Site 9 | 38 | 3.47 | 6.6 | 8.9 | 31.6 | 19.5 | 16.9 | 13.83 | 105.3 | 8.8 | 11.4 | 73 | 13.4 | 3.8 | 14.2 | 17.6 | 386.3 |  |
| Site 10 | 172.3 | 1.23 | 39.6 | 4 | 16.2 | 17.1 | 33.7 | 5.42 | 129.5 | 29 | 10.2 | 35 | 6.4 | 8.1 | 7.1 | 6.2 | 521.05 |  |
| Site 11 | 86.1 | 8.53 | 21.6 | 11.2 | 56.6 | 23.4 | 57 | 49.4 | 281.1 | 50 | 65 | 11.6 | 45.6 | 9.6 | 37.6 | 53.2 | 867.53 |  |
| Site 12 | 57.4 | 2.68 | 16.2 | 2.2 | 6.6 | 7.9 | 3.3 | 1.88 | 89.8 | 5.3 | 12.8 | 113.1 | 6.9 | 4.8 | 3.5 | 3.3 | 337.66 |  |
| Site 13 | 0.5 | 0.35 | 1.8 | 1.8 | 3.1 | 4.4 | 1.6 | 0.17 | 44.6 | 5.3 | 4.5 | 15.2 | 0.22 | 3.5 | 0.19 | 0.22 | 87.45 |  |
| Site 14 | 116.7 | 10.59 | 41.2 | 4.4 | 16.3 | 19.1 | 14.2 | 13.54 | 238.7 | 23.8 | 44.7 | 327.3 | 33.8 | 7.4 | 10.4 | 13 | 935.13 |  |
| Site 15 | 0.5 | 0.35 | 2.6 | 2.5 | 13.3 | 6 | 0.3 | 23.48 | 40 | 28.9 | 2.5 | 17 | 0.22 | 3.2 | 5.4 | 0.22 | 146.47 |  |
| Site 16 | 47 | 0.35 | 15.4 | 3 | 7.8 | 13 | 2.2 | 1.91 | 59.4 | 19.4 | 29.4 | 71.1 | 23.3 | 5.1 | 2 | 3.1 | 303.46 |  |
| Site 1 | 1.6 | 1 | 55.6 | 3.6 | 11.6 | 75.1 | 81.5 | 8.1 | 11.3 | 12 | 5 | 72 | 6.7 | 0.3 | 1.1 | 1.2 | 347.7 | 37 |
| Site 2 | 0.3 | 0.3 | 2.5 | 175.9 | 172.2 | 0.3 | 2.5 | 9.3 | 4.6 | 0.3 | 25.8 | 8 | 16.3 | 8 | 23.6 | 11.3 | 461.2 |  |
| Site 3 | 0.3 | 0.3 | 7.7 | 5.1 | 21.1 | 0.3 | 14.6 | 1.4 | 0.3 | 0.3 | 1 | 12 | 0.3 | 0.3 | 0.3 | 0.3 | 65.6 |  |
| Site 4 | 178 | 71.4 | 156.2 | 73.2 | 283.1 | 132.6 | 60.6 | 181.3 | 142.1 | 107.9 | 121.4 | 285 | 98.1 | 147.7 | 55.8 | 138 | 2232.4 |  |
| Site 1 | 81.7 | 9.6 | 3.9 | 29.8 | 184.6 | 14.7 | 146.3 | 100.9 | 62 | 127.5 | 70 | 18.2 | 32.5 | 38.3 | 16.1 | 36.5 | 972.6 | 5 |
| Site 2 | 5.8 | 1.2 | 0.3 | 1.8 | 4.7 | 0.8 | 0.4 | 0.4 | 0.06 | 0.3 | 0.4 | 0.06 | 0.06 | 0.06 | 0.06 | 0.2 | 16.6 |  |
| Site 3 | 59.6 | 3.8 | 1.8 | 8.8 | 57.7 | 5.6 | 7.9 | 12.1 | 3.8 | 25 | 12.2 | 2.4 | 6 | 4 | 4.8 | 6.2 | 221.7 |  |
| Site 4 | 23.6 | 0.6 | 0.3 | 0.6 | 3 | 0.4 | 1.3 | 1.1 | 0.5 | 1.3 | 0.6 | 0.2 | 0.3 | 0.2 | 0.06 | 0.3 | 34.36 |  |
| Site 5 | 4.5 | 1 | 0.06 | 2 | 7.2 | 0.8 | 0.5 | 0.4 | 0.06 | 0.2 | 0.2 | 0.06 | 0.06 | 0.06 | 0.06 | 0.2 | 17.36 |  |
| Site 6 | 35 | 0.6 | 0.5 | 1 | 4.2 | 0.6 | 3.4 | 3.6 | 1.3 | 3.5 | 1.7 | 0.5 | 0.8 | 0.7 | 0.4 | 1 | 58.8 |  |
| Site 7 | 28.5 | 2.6 | 1.6 | 4.8 | 19.2 | 1.9 | 10 | 9.1 | 1.3 | 4.3 | 1.2 | 0.3 | 0.4 | 0.06 | 0.06 | 0.3 | 85.62 |  |
| Site 8 | 20.1 | 1.5 | 0.8 | 4.5 | 18.7 | 1.5 | 4.3 | 5.1 | 1.3 | 4 | 2.2 | 0.7 | 1 | 1.3 | 0.5 | 1.4 | 68.9 |  |
| Site 9 | 14.8 | 0.8 | 0.4 | 4 | 14.8 | 1 | 2.9 | 2.4 | 0.06 | 3.5 | 4.4 | 0.9 | 1.3 | 1.7 | 0.8 | 2.4 | 56.16 |  |
| Site 10 | 12.6 | 1 | 1 | 5.2 | 23.1 | 2.2 | 8.5 | 5.4 | 2.5 | 7.2 | 9.3 | 2.1 | 3.1 | 3.8 | 1.8 | 4.2 | 93 |  |
| Site 11 | 21.9 | 1.8 | 1.2 | 8.3 | 36 | 4 | 8.9 | 7.7 | 4.2 | 10.4 | 14.4 | 3.1 | 5.1 | 6.5 | 2.9 | 7.5 | 143.9 |  |
| Site 12 | 40.2 | 13.3 | 2.5 | 25.1 | 88.4 | 16.7 | 27 | 21.6 | 10.6 | 21.8 | 26.2 | 6.1 | 11.4 | 11.9 | 5.1 | 13.7 | 341.6 |  |
| Site 13 | 12.6 | 1.6 | 1.1 | 3.4 | 15.4 | 2 | 9.5 | 7.1 | 2.9 | 8 | 10.1 | 2.5 | 3.2 | 3.9 | 1.3 | 3.8 | 88.4 |  |
| Site 14 | 35.7 | 9.8 | 2 | 17.9 | 86.3 | 14.4 | 117.5 | 83.4 | 47.2 | 82.1 | 48.1 | 15.1 | 27 | 19.1 | 6.3 | 22.6 | 634.5 |  |
| Site 15 | 13.7 | 1 | 0.8 | 2.7 | 8.8 | 1.4 | 6.4 | 5.6 | 2.4 | 5.2 | 2.9 | 0.9 | 1.3 | 1.4 | 0.6 | 1.7 | 56.8 |  |
| Site 16 | 16.9 | 1.9 | 1.3 | 8.1 | 29.8 | 4.9 | 24.9 | 17.5 | 6.6 | 18.8 | 9.4 | 2.2 | 2.8 | 3.9 | 1.9 | 4.4 | 155.3 |  |
| Site 17 | 90.1 | 7.8 | 12 | 16.2 | 127.7 | 20.7 | 217.4 | 152.3 | 91.4 | 199.6 | 125.6 | 121.6 | 47.9 | 56.3 | 20.4 | 51 | 1358 |  |
| Site 18 | 11.6 | 0.8 | 0.3 | 2.6 | 9.3 | 1.8 | 1 | 0.8 | 0.2 | 0.7 | 1.1 | 0.3 | 0.3 | 0.5 | 0.2 | 0.6 | 32.1 |  |
| Site 19 | 17.3 | 5.1 | 0.9 | 5.7 | 13 | 2.3 | 3.5 | 3.1 | 1.4 | 2.9 | 4.6 | 1.2 | 1.8 | 2 | 1 | 2.3 | 68.1 |  |
| Site 1 | 32.76 | 4.923 | 1.522 | 15.03 | 48.95 | 12.04 | 47.29 | 28.65 | 8.997 | 9.616 | 22.34 | 19.68 | 17.39 | 0.5 | 0.5 | 0.5 | 270.688 | 38 |
| Site 2 | 25.52 | 4.95 | 2.007 | 57.3 | 78.71 | 10.78 | 21.16 | 11.45 | 0.5 | 3.271 | 7.227 | 0.5 | 6.797 | 0.5 | 0.5 | 0.5 | 231.672 |  |
| Site 3 | 27.95 | 8.077 | 7.744 | 89.22 | 136.1 | 28.26 | 72.86 | 41.81 | 15.62 | 12.6 | 33.44 | 24.39 | 10.84 | 0.5 | 0.5 | 12.65 | 522.561 |  |
| Site 1 | 2.8 | 1.9 | 1.2 | nd | 110 | 23.4 | 260 | 192.6 | 29.7 | 2.6 | 135.2 | nd | 131.7 | 73.3 | 10.7 | 122.3 | 1097.4 | 39 |
| Site 2 | 4.6 | 0.3 | 0.6 | 3.4 | 70.5 | 0.3 | 138.1 | 111 | 19.3 | 1.9 | 165.1 | nd | 52.7 | 112.9 | 29.9 | 85.5 | 796.1 |  |
| Site 3 | 0.8 | 1.3 | 0.6 | 3 | 66.9 | 17.5 | 155.9 | 140.5 | 7.2 | 88.2 | 113.1 | nd | 135 | 21.8 | 0.3 | 276.7 | 1028.8 |  |
| Site 4 | 6.9 | 1.6 | 5.5 | 5.3 | 95.2 | 89.3 | 189.9 | 244 | 21 | 1.1 | 115.3 | nd | 61.4 | 58.8 | 18.1 | 101.8 | 1015.2 |  |
| Site 5 | 0.9 | 1.9 | 0.8 | 4 | 71.5 | 7.1 | 207.9 | 183.3 | 18.7 | 0 | 40.3 | nd | 48.5 | nd | nd | 98.8 | 683.7 |  |
| Site 1 | nd | nd | nd | nd | 2.2 | 0.01 | 6.5 | 4.2 | 2.1 | 5.6 | 1.8 | 0.05 | 0.7 | nd | nd | 0.05 | 23.21 | 40 |
| Site 2 | nd | nd | nd | 1.1 | 7.4 | 0.3 | 15.5 | 9.5 | 4.2 | 8.9 | 0.05 | 3.6 | 10.5 | 27.8 | 4.5 | 22.5 | 115.85 |  |
| Site 3 | nd | nd | 0.1 | 18.4 | 43.5 | 0.7 | 49.3 | 26.7 | 8.6 | 21.8 | 0.49 | 0.6 | 11.8 | 2 | 0.05 | 0.05 | 184.09 |  |
| Site 4 | nd | 1.3 | 0.3 | 7.3 | 91.4 | 6.3 | 121.7 | 90.5 | 45.2 | 81 | 24.5 | 2.3 | 23.7 | 0.05 | 0.05 | 0.05 | 495.65 |  |
| Site 5 | 1.4 | 0.5 | 0.1 | 4.2 | 2.9 | 0.7 | 8 | 16.2 | 11.8 | 42.6 | 1.7 | 0.05 | 2.6 | 5.2 | 0.05 | 8.3 | 106.3 |  |
| Site 6 | nd | nd | 0.1 | 0.05 | 10.8 | 1.5 | 13.1 | 22.7 | 7.2 | 12.2 | 3.3 | 0.4 | 4 | nd | nd | nd | 75.35 |  |
| Site 7 | 2.7 | nd | 1.1 | 59.5 | 237.1 | 18.9 | 303.2 | 211.4 | 108.9 | 149 | 35.9 | 0.05 | 41.5 | 2.7 | nd | nd | 1171.95 |  |
| Site 8 | nd | nd | nd | nd | 42.4 | 5.1 | 54.4 | 0.05 | 17.5 | 44.5 | 8 | 1.5 | 7.6 | nd | nd | nd | 181.05 |  |
| Site 9 | 22.7 | nd | 0.8 | nd | 94.1 | 15.3 | 315 | 221.3 | 238.4 | 97.3 | 49.1 | 11.2 | 55.3 | 5.7 | nd | 3.5 | 1129.7 |  |
| Site 10 | 0.1 | 2.4 | 4.5 | 2.8 | 80.9 | 27.6 | 217.4 | 157.5 | 102.6 | 158 | 47.7 | 11.9 | 69.4 | 2.5 | 1.8 | 7.1 | 894.2 |  |
| Site 11 | nd | nd | nd | 86.3 | 47.8 | 4.3 | 105.5 | 76.8 | 29.5 | 54 | 11.7 | 2.9 | 9.4 | 59.2 | nd | 33 | 520.4 |  |
| Site 1 | 5.5 | nd | 0.6 | 36.8 | 84.6 | 6.1 | 80.3 | 59 | 23.2 | 43.4 | 12.4 | 0.05 | 10.1 | 38.9 | nd | nd | 400.95 | 41 |
| Site 2 | 10.35 | 16.85 | 37.28 | 4.72 | 48.64 | 16.66 | 184.99 | 149.69 | 102.09 | 106.15 | 204.62 | 32.62 | 165.55 | 249.02 | 58.32 | 194.69 | 1582.24 |  |
| Site 3 | 1.88 | 2.69 | 5.25 | 0.96 | 3.33 | 0.15 | 4.57 | 4.56 | 1.78 | 3.63 | 5.47 | 1.35 | 3.74 | 3.86 | 1.15 | 6.10 | 50.47 |  |
| Site 4 | 2.46 | 4.91 | 10.64 | 1.83 | 6.77 | 0.15 | 9.44 | 10.30 | 3.44 | 7.39 | 5.20 | 4.08 | 7.66 | 0.49 | 0.67 | 13.05 | 88.46 |  |
| Site 5 | 12.54 | 20.92 | 77.51 | 8.07 | 66.45 | 22.38 | 0.05 | 0.05 | 85.92 | 102.00 | 26.20 | 31.25 | 42.54 | 0.13 | 0.66 | 14.67 | 511.34 |  |
| Site 6 | 12.36 | 21.53 | 41.29 | 4.92 | 37.15 | 9.79 | 0.05 | 57.64 | 73.09 | 88.82 | 51.46 | 41.25 | 64.46 | 0.01 | 0.17 | 39.09 | 543.06 |  |
| Site 7 | 15.23 | 31.19 | 44.09 | 4.74 | 37.67 | 11.91 | 0.05 | 0.05 | 109.28 | 126.27 | 163.80 | 134.04 | 184.56 | 37.55 | 15.02 | 217.63 | 1133.09 |  |
| Site 8 | 13.46 | 25.59 | 39.10 | 4.58 | 37.92 | 12.10 | 0.05 | 79.67 | 112.91 | 128.30 | 282.13 | 69.40 | 232.41 | 381.09 | 87.00 | 294.42 | 1800.13 |  |
| Site 9 | 11.30 | 24.75 | 39.45 | 4.22 | 31.64 | 9.38 | 66.43 | 143.65 | 98.66 | 115.33 | 267.32 | 33.68 | 462.14 | 353.62 | 86.77 | 276.27 | 2024.62 |  |
| Site 10 | 14.70 | 31.49 | 58.61 | 6.25 | 40.65 | 12.84 | 0.05 | 0.05 | 107.62 | 125.81 | 284.19 | 63.57 | 223.84 | 388.66 | 93.45 | 292.55 | 1744.32 |  |
| Site 1 | 6.90 | 92.00 | 237.00 | 475.00 | 1461.00 | 443.00 | 1319.00 | 1077.00 | 654.00 | 807.00 | 828.00 | 586.00 | 707.00 | 586.00 | 375.00 | 554.00 | 10207.9 | 42 |
| Site 2 | 213.00 | 29.00 | 58.00 | 261.00 | 358.00 | 39.00 | 181.00 | 197.00 | 96.00 | 197.00 | 261.00 | 328.00 | 101.00 | 25.00 | 17.00 | 72.00 | 2433 |  |
| Site 3 | 1.80 | 11.00 | 23.00 | 68.00 | 203.00 | 34.00 | 130.00 | 206.00 | 92.00 | 168.00 | 119.00 | 92.00 | 76.00 | 43.00 | 60.00 | 1.00 | 1327.8 |  |
| Site 4 | 60.00 | 17.00 | 17.00 | 50.00 | 87.00 | 12.00 | 39.00 | 48.00 | 27.00 | 27.00 | 61.00 | 41.00 | 41.00 | 29.00 | 8.00 | 33.00 | 597 |  |
| Site 5 | 57.00 | 19.00 | 19.00 | 76.00 | 105.00 | 11.00 | 48.00 | 62.00 | 31.00 | 73.00 | 105.00 | 66.00 | 61.00 | 39.00 | 33.00 | 50.00 | 855 |  |
| Site 6 | 38.00 | 1.00 | 1.00 | 26.00 | 53.00 | 5.00 | 21.00 | 24.00 | 16.00 | 40.00 | 45.00 | 31.00 | 35.00 | 35.00 | 4.00 | 35.00 | 410 |  |
| Site 7 | 74 | 8 | 8 | 45 | 99 | 3 | 61 | 13 | 8 | 61 | 346 | 273 | 346 | 146 | 76 | 1 | 1568 |  |
| Site 8 | 18 | nd | nd | 37 | 30 | 1 | 13 | 10 | 8 | 28 | 128 | 164 | 146 | 55 | 18 | 55 | 711 |  |
| Site 9 | 94 | 18 | nd | 46 | 96 | 3 | 70 | 28 | 8 | 67 | 386 | 258 | 331 | 92 | 74 | 18 | 1589 |  |
| Site 10 | 165 | 26 | 4 | 26 | 96 | 3 | 61 | 76 | 15 | 61 | 345 | 255 | 18 | 145 | 91 | 182 | 1569 |  |
| Site 11 | 56 | 11 | nd | 54 | 109 | 10 | 34 | 26 | 23 | 62 | 55 | 31 | 31 | 34 | 26 | 36 | 598 |  |
| Site 12 | 11 | nd | nd | nd | 11 | nd | 6 | 6 | 6 | 11 | 23 | 23 | 11 | 23 | 6 | 23 | 160 |  |
| Site 13 | 33 | nd | nd | 15 | 60 | 6 | 20 | 33 | 15 | 35 | 29 | 18 | 18 | 21 | 10 | 26 | 339 |  |
| Site 14 | 40 | nd | nd | 65 | 65 | 5 | 21 | 18 | 9 | 40 | 131 | 65 | 44 | 87 | 44 | 98 | 732 |  |
| Site 15 | 44 | 44 | nd | 87 | 66 | 6 | 26 | 14 | 9 | 37 | 206 | 131 | 75 | 112 | 56 | 94 | 1007 |  |
| Site 16 | 43 | 43 | nd | 87 | 65 | 5 | 25 | 14 | 9 | 37 | 204 | 130 | 74 | 111 | 56 | 93 | 996 |  |
| Site 17 | 56 | 4 | 7 | 32 | 137 | 21 | 248 | 222 | 163 | 232 | 1787 | 1326 | 1492 | 1326 | 958 | 1216 | 9227 |  |
| Site 1 | 35.52 | 40.63 | 44.38 | 9.4 | 17.26 | 5.06 | 6.71 | 39.62 | 1.6 | 16.14 | 5.99 | 27.96 | 29.79 | 14.82 | 92.92 | 19.86 | 407.66 | 43 |
| Site 2 | 12.1 | 70.96 | 17.61 | 114.04 | 9.11 | 2.96 | 23.55 | 20.92 | 4.26 | 25.69 | 21.66 | 34.65 | 38.06 | 8.37 | 14.79 | 9.25 | 427.98 |  |
| Site 3 | 11.65 | 44.1 | 7.68 | 34.69 | 1.44 | 2.76 | 0.58 | 6.31 | 4.23 | 5 | 1.65 | 6.1 | 7.76 | 3.15 | 14.35 | 4.81 | 156.26 |  |
| Site 1 | 4.86 | 1.54 | 7.73 | 3.88 | 12.44 | 1.16 | 36.41 | 41.81 | 35.53 | 78.75 | 67.98 | 12.46 | 35.45 | 77.45 | 243 | 25.23 | 685.68 | 44 |
| Site 2 | 1269.8 | 7.78 | 12.77 | 8.37 | 15.69 | 35.31 | 81.77 | 119.51 | 33.41 | 32.67 | 6.29 | 19.12 | 74.27 | 161.08 | 207.43 | 203.01 | 2288.28 |  |
| Site 3 | 18.88 | 0 | 8.37 | 16.54 | 11.36 | 11.22 | 7.2 | 11.79 | 12.93 | 25 | 8.35 | 0.86 | 14.28 | 70.45 | 60.07 | 90 | 367.3 |  |
| Site 4 | 65.95 | 1.88 | 2.63 | 11.09 | 2.93 | 7.76 | 20.58 | 24.89 | 16.14 | 47.84 | 2.93 | 1.09 | 30.93 | 82.31 | 87.81 | 92.48 | 499.24 |  |
| Site 1 | 30.56 | 9.28 | 4.84 | 31.1 | 65.12 | 8.66 | 28.94 | 32.34 | 29.98 | 29.69 | 10.36 | 4.4 | 14.65 | 13.2 | 15.4 | 10.66 | 339.18 | 45 |
| Site 2 | 39.88 | 3.4 | 1.83 | 22.93 | 51.47 | 3.43 | 27.11 | 17.91 | 8.82 | 10.75 | 4.82 | 1.86 | 7.44 | 9.97 | 15.07 | 8 | 234.69 |  |
| Site 3 | 278.4 | 4.35 | 19.33 | 127.45 | 297.13 | 221.35 | 179.67 | 172.41 | 47.35 | 161.22 | 69.41 | 16.82 | 62.08 | 56.49 | 32.4 | 4.2 | 1750.06 |  |
| Site 4 | 176.9 | 14.63 | 13.21 | 39.76 | 18.36 | 133.96 | 68.13 | 4.14 | 18.46 | 21.23 | 5.68 | 3.39 | 41.99 | 31.23 | 15.69 | 26.89 | 633.65 |  |
| Site 5 | 225.86 | 14.55 | 14.59 | 72.03 | 226.76 | 188.9 | 75.03 | 7.82 | 21.9 | 33.17 | 2.99 | 1.58 | 25.91 | 9.06 | 3.15 | 20.39 | 943.69 |  |
| Site 6 | 34.65 | 3.47 | 6.69 | 9.4 | 36.7 | 31.16 | 27.92 | 15.19 | 5.24 | 8.84 | 5.58 | 1.92 | 8.68 | 10.98 | 15.36 | 8.08 | 229.86 |  |
| Site 7 | 68.15 | 4.9 | 5.12 | 30.29 | 72.21 | 8.97 | 74.27 | 17.13 | 16.72 | 18.47 | 19.76 | 1.51 | 19.44 | 9.09 | 15.04 | 9.08 | 390.15 |  |
| Site 8 | 48.56 | 3.29 | 2.35 | 16.45 | 40.27 | 34.1 | 32.06 | 16.72 | 7.05 | 2.56 | 4.07 | 2.56 | 8.6 | 9.75 | 15.14 | 7.61 | 251.14 |  |
| Site 9 | 100.51 | 10.02 | 14.11 | 52.27 | 106.22 | 88.99 | 128.8 | 59.52 | 19.31 | 31.61 | 18.34 | 12.39 | 22.8 | 29.71 | 17.4 | 26.5 | 738.5 |  |
| Site 1 | 178.86 | 23.57 | 17.02 | 117 | 250.5 | 208.4 | 140.72 | 150.53 | 26.72 | 84.38 | 13.04 | 2.79 | 68.21 | 9.27 | 9.97 | 30.16 | 1331.14 | 46 |
| Site 2 | 211 | 14.81 | 0.75 | 84.99 | 364.6 | 34.16 | 87.61 | 61.19 | 29.76 | 44.07 | 41.47 | 20.79 | 42.22 | 35.34 | 10.49 | 44.31 | 1127.56 |  |
| Site 3 | 114.7 | 4.51 | 1.85 | 16.22 | 45.04 | 4.92 | 13.29 | 11.4 | 6.2 | 7.5 | 3.05 | 1.86 | 9.11 | 7.45 | 2.22 | 9.46 | 258.78 |  |
| Site 4 | 306.70 | 26.33 | 18.91 | 102.40 | 377.20 | 53.80 | 359.40 | 275.80 | 163.90 | 222.90 | 255.50 | 97.57 | 242.70 | 257.40 | 78.38 | 285.50 | 3124.39 |  |
| Site 1 | 202.10 | 22.22 | 8.81 | 98.44 | 245.20 | 30.90 | 103.00 | 77.53 | 43.77 | 54.96 | 95.26 | 33.62 | 53.56 | 73.31 | 20.75 | 92.61 | 1256.04 | 47 |
| Site 2 | 172.90 | nd | nd | 29.77 | 87.59 | nd | 21.24 | 16.07 | 0.01 | 12.31 | 35.90 | 0.75 | 12.83 | 12.17 | 0.75 | 15.35 | 417.64 |  |
| Site 3 | 610.30 | 14.08 | 0.75 | 48.81 | 128.80 | 13.36 | 63.68 | 38.93 | 63.97 | 32.44 | 53.81 | 18.12 | 27.66 | 25.39 | 0.75 | 35.56 | 1176.41 |  |
| Site 4 | 185.90 | 14.88 | 17.90 | 44.40 | 172.00 | 20.27 | 158.30 | 120.60 | 70.02 | 85.05 | 90.33 | 45.35 | 105.00 | 88.54 | 30.01 | 97.46 | 1346.01 |  |
| Site 5 | 202.10 | 14.81 | 0.75 | 48.81 | 172.00 | 20.27 | 87.61 | 61.19 | 43.77 | 44.07 | 53.81 | 20.79 | 42.22 | 35.34 | 10.49 | 44.31 | 902.34 |  |
| Site 6 | 257.66 | 14.55 | 8.92 | 60.72 | 202.92 | 23.20 | 115.22 | 85.93 | 54.66 | 65.60 | 82.19 | 31.76 | 70.44 | 71.37 | 21.69 | 82.89 | 1249.72 |  |
| Site 7 | 183.20 | 20.14 | 7.98 | 89.22 | 222.20 | 28.01 | 93.38 | 70.27 | 39.67 | 49.81 | 86.34 | 30.47 | 48.54 | 66.44 | 18.80 | 83.93 | 1138.4 |  |
| Site 8 | 482.30 | 25.20 | 10.60 | 89.56 | 288.10 | 27.40 | 160.80 | 111.60 | 86.45 | 127.80 | 166.20 | 54.53 | 118.70 | 109.40 | 44.86 | 137.30 | 2040.8 |  |
| Site 9 | 238.40 | 23.28 | 0.00 | 75.31 | 283.00 | 39.48 | 222.60 | 187.30 | 125.00 | 174.90 | 185.90 | 83.35 | 183.10 | 163.10 | 53.13 | 186.00 | 2223.85 |  |
| Site 10 | 639.50 | 42.69 | 172.30 | 521.00 | 977.60 | 264.20 | 1195.00 | 801.40 | 462.10 | 440.90 | 529.90 | 227.40 | 375.90 | 304.50 | 84.26 | 321.20 | 7359.85 |  |
| Site 11 | 592.80 | 121.00 | 76.56 | 316.70 | 1205.00 | 339.90 | 1183.00 | 1005.00 | 704.20 | 830.80 | 1199.00 | 394.70 | 1065.00 | 887.60 | 301.40 | 1074.00 | 11296.66 |  |
| Site 12 | 150.20 | 13.63 | nd | 18.21 | 63.02 | nd | 19.10 | 12.73 | nd | nd | nd | nd | nd | nd | nd | nd | 276.89 |  |
| Site 13 | 250.20 | nd | nd | 40.12 | 136.10 | 10.40 | 35.88 | 26.08 | 12.91 | 18.07 | 37.67 | 12.92 | 21.61 | 18.71 | 0.75 | 26.15 | 647.57 |  |
| Site 14 | 254.20 | nd | nd | 39.07 | 109.90 | 10.54 | 43.82 | 31.55 | 19.51 | 28.52 | 51.01 | 20.77 | 27.31 | 30.70 | 10.31 | 37.33 | 714.54 |  |
| Site 15 | 125.10 | nd | nd | 24.72 | 86.17 | 12.00 | 52.92 | 38.10 | 20.16 | 22.84 | 35.43 | 15.91 | 38.77 | 34.06 | 0.75 | 38.57 | 545.5 |  |
| Site 16 | 250.20 | 20.14 | nd | 75.31 | 222.20 | 27.40 | 93.38 | 70.27 | 39.67 | 49.81 | 86.34 | 30.47 | 48.54 | 66.44 | 18.80 | 83.93 | 1182.9 |  |
| Site 17 | 323.99 | 28.99 | 32.49 | 134.88 | 374.57 | 81.88 | 334.06 | 253.78 | 163.89 | 188.74 | 255.16 | 93.89 | 209.33 | 179.95 | 58.64 | 212.16 | 2926.4 |  |
| Site 18 | 157.50 | 11.13 | 10.72 | 29.04 | 113.70 | 9.21 | 68.86 | 46.83 | 20.45 | 30.63 | 36.36 | 19.23 | 37.07 | 32.86 | 10.07 | 34.80 | 668.46 |  |
| Site 19 | 201.10 | 18.50 | nd | 62.02 | 171.50 | 19.08 | 79.94 | 52.78 | 30.38 | 40.93 | 61.93 | 24.01 | 47.39 | 51.42 | 17.30 | 65.52 | 943.8 |  |
| Site 20 | 246.90 | 6.38 | 6.74 | 75.88 | 300.20 | 119.80 | 236.60 | 209.70 | 96.44 | 149.20 | 144.50 | 91.03 | 103.60 | 109.60 | 24.34 | 114.80 | 2035.71 |  |
| Site 21 | 161.70 | 23.43 | 7.03 | 43.19 | 123.80 | 12.74 | 39.55 | 30.21 | 16.89 | 21.88 | 34.24 | 13.80 | 27.33 | 28.49 | 7.93 | 32.15 | 624.36 |  |
| Site 22 | 284.10 | 34.80 | 13.90 | 155.30 | 432.80 | 73.35 | 245.80 | 197.20 | 96.40 | 134.10 | 160.30 | 51.97 | 118.50 | 119.70 | 44.57 | 152.40 | 2315.19 |  |
| Site 23 | 222.20 | 11.79 | nd | 31.20 | 94.87 | 20.64 | 24.66 | 20.52 | nd | nd | nd | nd | nd | nd | nd | nd | 425.88 |  |
| Site 24 | 213.00 | 10.38 | nd | 40.13 | 142.60 | 12.82 | 51.46 | 41.07 | 23.79 | 39.28 | 63.03 | 17.47 | 37.03 | 28.63 | 10.13 | 35.22 | 766.04 |  |
| Site 25 | 220.50 | 15.42 | nd | 36.91 | 106.40 | 11.63 | 33.05 | 23.92 | 11.51 | 12.80 | 26.81 | 10.51 | 16.72 | 11.02 | 0.75 | 12.73 | 550.68 |  |
| Site 26 | 216.75 | 13.61 | 5.87 | 41.66 | 133.20 | 15.95 | 60.16 | 43.95 | 22.12 | 34.96 | 49.15 | 18.35 | 37.05 | 30.75 | 10.10 | 35.01 | 768.64 |  |
| Site 27 | 213.38 | 16.48 | 7.30 | 59.21 | 185.73 | 34.91 | 97.49 | 77.78 | 37.61 | 54.23 | 66.52 | 29.13 | 49.08 | 48.34 | 15.54 | 56.58 | 1049.31 |  |
| Site 1 | 2.10 | 1.40 | 0.80 | 7.40 | 20.80 | nd | 65.90 | 47.60 | nd | nd | 20.10 | 18.70 | 18.10 | 33.60 | 25.20 | 32.40 | 294.1 | 48 |
| Site 2 | 3.90 | nd | nd | nd | 31.40 | 7.23 | 54.30 | 47.20 | 11.20 | nd | 17.20 | 11.30 | 18.40 | 27.60 | 31.20 | 12.70 | 273.63 |  |
| Site 3 | 5.50 | nd | 0.60 | 16.50 | 37.30 | 30.10 | 61.70 | 48.30 | nd | 5.30 | 40.30 | nd | 45.20 | 21.30 | 32.70 | 15.90 | 330.71 |  |
| Site 4 | nd | nd | 1.40 | 5.30 | 73.00 | nd | 126.30 | 123.90 | nd | 2.90 | 3.70 | 2.10 | 20.20 | 19.50 | 35.80 | 22.30 | 436.4 |  |
| Site 5 | 1.50 | nd | nd | 2.50 | 23.90 | 10.20 | 173.20 | 111.20 | 23.40 | nd | 4.10 | 3.90 | 44.80 | 13.60 | 25.10 | 61.50 | 498.9 |  |
| Site 6 | 0.90 | nd | nd | 4.20 | 50.90 | 10.10 | 103.40 | 99.30 | nd | nd | nd | 4.50 | 30.20 | 21.40 | 27.50 | 30.60 | 383 |  |
| Site 7 | 4.60 | 0.70 | nd | 3.50 | 69.20 | 50.30 | 141.20 | 111.50 | nd | 1.90 | 107.20 | nd | 50.50 | 101.40 | 29.90 | 85.70 | 757.6 |  |
| Site 8 | 2.80 | 1.90 | 1.30 | nd | 110.40 | 15.70 | 209.70 | 178.50 | 25.40 | nd | 120.60 | nd | 81.70 | 60.20 | 55.40 | 80.70 | 944.3 |  |
| Site 1 | 140.00 | 10.00 | 2.20 | 12.90 | 66.30 | 5.00 | 37.40 | 36.60 | 4.10 | 12.80 | 7.90 | 2.40 | 2.40 | 1.50 | 4.00 | 3.50 | 349 | 49 |
| Site 2 | 153.80 | 4.90 | 2.90 | 13.90 | 50.10 | 38.70 | 64.40 | 83.10 | 98.10 | 1106.70 | 66.30 | 0.00 | 89.50 | 51.20 | 95.50 | 342.30 | 2261.4 |  |
| Site 3 | 100.80 | 17.60 | 9.70 | 24.10 | 256.50 | 97.50 | 203.40 | 752.90 | 202.00 | 957.50 | 253.80 | 56.20 | 183.20 | 104.50 | 83.30 | 325.30 | 3628.3 |  |
| Site 4 | 100.20 | 21.20 | 10.50 | 12.40 | 82.00 | 30.80 | 71.20 | 229.90 | 53.50 | 419.90 | 123.60 | 28.80 | 119.00 | 58.40 | 75.80 | 215.60 | 1652.8 |  |
| Site 1 | 98.66 | 13.35 | 2.41 | 12.12 | 47.14 | 5.00 | 24.26 | 24.39 | 5.23 | 6.13 | 4.04 | 2.21 | 4.19 | 7.08 | 0.40 | 53.05 | 309.649 | 50 |
| Site 2 | 87.25 | 29.60 | 5.56 | 23.61 | 51.43 | 2.96 | 7.09 | 17.03 | 1.29 | 1.78 | 0.40 | 0.40 | 0.40 | 5.15 | 0.40 | 75.29 | 309.635 |  |
| Site 3 | 197.7 | 8.026 | 10.62 | 38.76 | 195 | 29.13 | 201.6 | 178.9 | 94.32 | 96.8 | 100.9 | 33.07 | 104.7 | 77.22 | 9.226 | 90.69 | 1466.662 |  |
| Site 4 | 130.1 | 15.79 | 24.1 | 16.03 | 192.9 | 39.81 | 196.9 | 191.1 | 80.75 | 49.73 | 102.9 | 37.48 | 96.31 | 79.37 | 9.127 | 131.8 | 1394.197 |  |
| Site 5 | 276 | 21.05 | 19.61 | 37.53 | 344.3 | 44.66 | 332.2 | 248.6 | 147.9 | 94.14 | 217.9 | 52.91 | 173.1 | 140.5 | 16.37 | 188.2 | 2354.97 |  |
| Site 6 | 191 | 15.79 | 68.63 | 116.3 | 322.1 | 68.45 | 338.7 | 298.8 | 164.1 | 119.9 | 211.9 | 103.6 | 182.9 | 157.7 | 23.81 | 241.3 | 2624.98 |  |
| Site 7 | 130.9 | 21.05 | 1.838 | 14.62 | 48.57 | 5.194 | 13.53 | 35.57 | 5.103 | 7.06 | 4.843 | 2.205 | 5.304 | 6.113 | 0.4 | 47.91 | 350.21 |  |
| Site 8 | 83.17 | 19.08 | 3.717 | 9.866 | 60.71 | 6.845 | 12.5 | 32.52 | 5.491 | 7.06 | 4.641 | 2.072 | 5.165 | 7.615 | 0.4 | 70.16 | 331.012 |  |
| Site 9 | 139.4 | 32.24 | 5.801 | 24.49 | 67.86 | 7.67 | 9.145 | 27.44 | 7.235 | 9.325 | 4.036 | 3.307 | 5.863 | 12.23 | 0.893 | 75.29 | 432.225 |  |
| Site 10 | 210.4 | 28.95 | 8.987 | 30.66 | 174.3 | 11.65 | 13.06 | 32.52 | 25.84 | 23.98 | 16.55 | 10.36 | 33.5 | 34.32 | 4.365 | 133.5 | 792.942 |  |
| Site 11 | 191.6 | 31.58 | 7.761 | 21.49 | 122.9 | 16.5 | 8.772 | 34.55 | 16.8 | 17.76 | 9.282 | 3.086 | 15.08 | 19.31 | 2.183 | 112.9 | 631.554 |  |
| Site 1 | 106.3 | 227 | 37.2 | 36.4 | 26.3 | 18 | 72 | 38 | 26 | 62 | 325 | 46 | 232 | 92 | nd | 82.3 | 1426.5 | 51 |
| Site 2 | 169.2 | 154 | 53.6 | 72.5 | 62.6 | 27 | 89 | 28 | 63 | 23 | 462 | 72 | 348 | 168 | 62 | 106.5 | 1960.4 |  |
| Site 3 | 354.2 | 342 | 69.8 | 63.5 | 39.4 | 46 | 156 | 126 | 65 | 64 | 568 | 206 | 281 | 132 | 35 | 131.8 | 2679.7 |  |
| Site 4 | 296.5 | 562 | 87.2 | 75.2 | 163.2 | 28 | 189 | 85 | 87 | 72 | 551 | 167 | 542 | 157 | 132 | 79.3 | 3273.4 |  |
| Site 5 | 462.5 | 589 | 163.5 | 77.9 | 242.9 | 72 | 262 | 152 | 106 | 63 | 625 | 89 | 627 | 142 | 76 | 127.6 | 3877.4 |  |
| Site 6 | 58.7 | 136 | 85.6 | 23.6 | 86 | 32 | 36 | 73 | nd | nd | 82 | 23 | 24 | 132 | nd | 25.2 | 817.1 |  |
| Site 7 | 184.3 | 264 | 72.3 | 82.3 | 92 | 62 | 75 | 79 | 67 | 27 | 164 | 64 | 136 | 241 | 87 | 85.6 | 1782.5 |  |
| Site 8 | 287.5 | 278 | 126.5 | 72.5 | 162 | 75 | 162 | 123 | 72 | 85 | 258 | 72 | 236 | 164 | 163 | 72.3 | 2408.8 |  |
| Site 9 | 56.2 | 172 | nd | 16.5 | 6.4 | nd | 21 | 32 | 2.5 | 2.5 | 32 | 20 | 124 | 26 | nd | nd | 511.1 |  |
| Site 10 | 262.3 | 263 | 168.5 | 86.5 | 132 | 75 | 79 | 62 | 85 | 12 | 365 | 69 | 236 | 72 | 27 | 56.3 | 2050.6 |  |
| Site 11 | 254.3 | 274 | 59.6 | 72.9 | 187 | 69 | 82 | 72 | 117 | 34 | 372 | 65 | 284 | 132 | 64 | 72.6 | 2211.4 |  |
| Site 12 | 156.3 | 165 | 26.3 | 16.3 | 21.2 | 62 | 25 | 69 | nd | nd | 178 | 78 | 165 | 96 | 2.5 | 2.5 | 1063.1 |  |
| Site 1 | 193.1 | / | 247.5 | 119.8 | 653.3 | 51.8 | 125.6 | 1063.6 | 42.7 | 9.7 | 34.8 | 7 | 10.9 | 12.7 | 13.6 | 19.4 | 2605.5 | 52 |
| Site 2 | 71.4 | / | 82.6 | 4.6 | 74.4 | 17.5 | 75.4 | 440.2 | 21.8 | 13.7 | 22.3 | 11.1 | 19.6 | 13.9 | 7.2 | 7.7 | 883.4 |  |
| Site 3 | 70.6 | / | 53.5 | 5 | 33.4 | 9.2 | 45.6 | 210.3 | 10.7 | 5.5 | 11.7 | 5 | 9 | 7.9 | 6.3 | 6.9 | 490.6 |  |
| Site 4 | 72.8 | / | 56.8 | 3.4 | 37.6 | 14.2 | 69.1 | 361.9 | 19.7 | 13.3 | 22.7 | 11.1 | 22 | 18.1 | 7.5 | 9.6 | 739.8 |  |
| Site 1 | 152 | / | 1.81 | 3.15 | 11.66 | 2.26 | 41.25 | 2.06 | 5.7 | 2.36 | 5.8 | 0.91 | 0.396 | 5 | 0.79 | 26.67 | 261.816 | 53 |
| Site 2 | 58 | / | 0.113 | 1.27 | 4.01 | 0.74 | 3.07 | 0.61 | 0.27 | 0.81 | 0.71 | 0.37 | 0.51 | 0.52 | 0.051 | 6.49 | 77.544 |  |
| Site 1 | 45.6 | / | 8.3 | 27.5 | 101.2 | 35.4 | 111.4 | 118 | 110.6 | 66.6 | 97 | 23.4 | 59.2 | 114.7 | 7.2 | 151 | 1077.1 | 54 |
| Site 2 | 10.8 | / | 6.8 | 28.4 | 239 | 33.8 | 116.2 | 125.6 | 134.7 | 83.2 | 118.5 | 28.4 | 75.6 | 178.2 | 10.2 | 121.4 | 1310.8 |  |
| Site 3 | 8.7 | / | 6.4 | 18.3 | 92.5 | 26.2 | 95.8 | 107.7 | 133.1 | 55.4 | 91.8 | 25.4 | 60.1 | 141.5 | 9.3 | 152.2 | 1024.4 |  |
| Site 4 | 11.8 | / | 5.7 | 14.8 | 69 | 23.2 | 85.9 | 92.1 | 94.9 | 59 | 86.1 | 23.4 | 57.6 | 59.1 | 9.1 | 100.6 | 792.3 |  |
| Site 5 | 8.2 | / | 6.4 | 17.3 | 98.3 | 26.1 | 91.1 | 108.8 | 129.6 | 52.3 | 92 | 23.6 | 58.1 | 113.7 | 9.9 | 135.5 | 970.9 |  |
| Site 6 | 5.9 | / | 5.9 | 18.8 | 86.2 | 23.8 | 81.9 | 93.2 | 115.2 | 49 | 87.7 | 22.2 | 54.9 | 171.1 | 8.6 | 168.5 | 992.9 |  |
| Site 7 | 4.3 | / | 6.2 | 19.8 | 96.7 | 25.1 | 88.7 | 94.7 | 94.2 | 59.8 | 86.1 | 23.6 | 55 | 57.2 | 11.2 | 95.2 | 817.8 |  |
| Site 8 | 14.6 | / | 5.2 | 17.7 | 76.8 | 23.7 | 80.8 | 87.5 | 89.7 | 55.1 | 83.5 | 21.4 | 54.1 | 107.1 | 7.7 | 122.5 | 847.4 |  |
| Site 9 | 7 | / | 6.5 | 15.7 | 95.3 | 16.1 | 84.4 | 89.4 | 77.3 | 52.2 | 66.4 | 19.3 | 44.7 | 45.3 | 7.3 | 72.1 | 699 |  |
| Site 10 | 8.4 | / | 5.3 | 9.5 | 33.8 | 13.3 | 44.6 | 46.9 | 49.1 | 34 | 46.1 | 13 | 33.6 | 31.5 | 5.4 | 53.1 | 427.6 |  |
| Site 11 | 2.5 | / | 6.4 | 12.8 | 66.9 | 14.5 | 69.8 | 74.6 | 73.4 | 34.6 | 60.9 | 17.5 | 39.4 | 72 | 6.5 | 72.7 | 624.5 |  |
| Site 12 | 5.4 | / | 5.7 | 11 | 52.6 | 14.8 | 60.7 | 60.2 | 52.6 | 25.8 | 39.1 | 11.8 | 25.2 | 25.9 | 4.8 | 28.3 | 423.9 |  |
| Site 13 | 7.7 | / | 5 | 9 | 38 | 9.9 | 44.2 | 48.6 | 53.7 | 26.3 | 43.7 | 11.2 | 26 | 44.3 | 4.8 | 39.8 | 412.2 |  |
| Site 14 | 39.4 | / | 14.1 | 34.2 | 120.1 | 34.2 | 117.6 | 132.2 | 144.7 | 63.5 | 121.9 | 28.2 | 67.8 | 76.7 | 8.4 | 129.1 | 1132.1 |  |
| Site 15 | 9.1 | / | 5.7 | 13.9 | 65.4 | 12.5 | 91.1 | 99.1 | 106 | 49 | 118.5 | 21.9 | 48.6 | 131.4 | 7.4 | 110.5 | 890.1 |  |
| Site 16 | 17.5 | / | 6.5 | 15.4 | 103.7 | 15.8 | 93.4 | 114.6 | 122.1 | 49.1 | 99.3 | 20.9 | 44.2 | 45.5 | 8 | 66.4 | 822.4 |  |
| Site 17 | 24.3 | / | 7.5 | 18.2 | 129.6 | 17.2 | 93 | 114.9 | 141.7 | 40.5 | 93 | 20.2 | 49.4 | 51.6 | 6.9 | 53.5 | 861.5 |  |
| Site 18 | 19.1 | / | 5.5 | 13.6 | 105.3 | 11.8 | 67.2 | 106.7 | 97.4 | 24.9 | 100.3 | 15 | 30.7 | 37.1 | 4.7 | 53.3 | 692.6 |  |
| Site 19 | 8.8 | / | 4.3 | 8.4 | 35.3 | 8.9 | 45.2 | 47.2 | 42.2 | 21.1 | 36.6 | 10.6 | 21.4 | 27.6 | 5 | 27.3 | 349.9 |  |
| Site 20 | 13.3 | / | 8.8 | 12.5 | 71.8 | 13.1 | 71.1 | 68.7 | 51.7 | 27.7 | 36.8 | 12.8 | 27.6 | 29.1 | 5 | 32 | 482 |  |
| Site 21 | 8 | / | 4.4 | 6.7 | 25.7 | 8.7 | 41.1 | 40.7 | 49.3 | 24.4 | 59.5 | 19.4 | 40.9 | 43.3 | 9.3 | 49.7 | 431.1 |  |
| Site 22 | 2.5 | / | 3.8 | 6.7 | 31.1 | 7.6 | 33.7 | 38.9 | 48.1 | 18.3 | 21.7 | 7.7 | 15.1 | 21.1 | 2.9 | 37.1 | 296.3 |  |
| Site 23 | 4.7 | / | 4.7 | 7 | 59.4 | 8 | 40.9 | 68.8 | 134.7 | 16.1 | 15.9 | 9.9 | 18.7 | 22 | 2.7 | 14 | 427.5 |  |
| Site 24 | 2.5 | / | 12.5 | 5.4 | 19.2 | 6 | 28.8 | 28.2 | 30.1 | 16.2 | 23.5 | 8.5 | 17.6 | 24.1 | 4.2 | 21.3 | 248.1 |  |
| Site 25 | 3.5 | / | 3.7 | 7.8 | 83.8 | 8.8 | 41.9 | 41.4 | 36.5 | 20.3 | 29.2 | 10 | 22.3 | 26.6 | 4.5 | 27.2 | 367.5 |  |
| Site 26 | 3.2 | / | 4.3 | 7 | 53.8 | 8.8 | 48.1 | 46 | 44.1 | 22.4 | 36.3 | 13.2 | 24.9 | 29.8 | 6 | 30.6 | 378.5 |  |
| Site 27 | 4 | / | 3.8 | 6.1 | 67.6 | 7.4 | 24.3 | 24.2 | 20.4 | 12.1 | 17.4 | 6 | 12.5 | 19.2 | 3 | 13.5 | 241.5 |  |
| Site 28 | 6.1 | / | 4.9 | 13.2 | 105.3 | 13.2 | 59.2 | 60.8 | 63.1 | 31.1 | 53.3 | 15.4 | 36.4 | 82.8 | 5.6 | 59.7 | 610.1 |  |
| Site 29 | 6.6 | / | 6.1 | 12.5 | 70.4 | 12.2 | 58.6 | 60.6 | 66.6 | 27.2 | 38.9 | 11.9 | 25.4 | 30.6 | 3.3 | 49.7 | 480.6 |  |
| Site 30 | 6.1 | / | 4.6 | 12.6 | 70.9 | 17.1 | 72.5 | 80 | 85.7 | 38.7 | 65 | 17.5 | 38.6 | 46.9 | 7.6 | 54.3 | 618.1 |  |
| Site 31 | 8.2 | / | 5.4 | 12.3 | 63.6 | 13.9 | 63.3 | 70.9 | 72.9 | 30.7 | 52.7 | 15.2 | 31.4 | 110 | 2.8 | 114 | 667.3 |  |
| Site 32 | 7.6 | / | 5.3 | 10.6 | 80.4 | 10.9 | 50.1 | 69.2 | 56.5 | 23 | 36.3 | 11.4 | 24.4 | 96.9 | 6.5 | 31.5 | 520.6 |  |
| Site 33 | 12.9 | / | 5.3 | 8.5 | 45.3 | 9.8 | 52 | 49.1 | 41 | 32.5 | 35.9 | 12.1 | 27 | 27.1 | 3.4 | 36 | 397.9 |  |
| Site 34 | 7.2 | / | 5.1 | 12.2 | 73.7 | 12.2 | 60.5 | 62.9 | 53.4 | 23.5 | 44.6 | 13.3 | 28.7 | 34 | 5.7 | 52.8 | 489.8 |  |
| Site 35 | 7.5 | / | 6.5 | 15.9 | 81 | 19.7 | 76.7 | 82 | 91.7 | 41.8 | 75.3 | 19.6 | 47.4 | 57.2 | 7.8 | 84.8 | 714.9 |  |
| Site 36 | 11 | / | 5.4 | 8.1 | 41.5 | 9.1 | 40.7 | 50.6 | 48.2 | 20.3 | 33.9 | 8.8 | 19.3 | 22.9 | 3.8 | 17.7 | 341.3 |  |
| Site 37 | 4.7 | / | 4.9 | 7.9 | 55.7 | 9.1 | 47.7 | 55.6 | 52.9 | 27.8 | 37.8 | 11.7 | 25.2 | 55 | 7.1 | 58.4 | 461.5 |  |
| Site 1 | 13.87 | 2.29 | 6.26 | 10.44 | 45.71 | 8.57 | 101.36 | 103.4 | 22.31 | 16.18 | 9.13 | 0.62 | 9.61 | 7.6 | 5.35 | 9.11 | 371.81 | 11 |
| Site 2 | 31.18 | 2.95 | 7.79 | 12 | 27.57 | 8.81 | 65.25 | 66.81 | 40.19 | 6.52 | 1.31 | 7.61 | 7.06 | 6.87 | 3.21 | 10.61 | 305.74 |  |
| Site 3 | 2.34 | 0.23 | 2.81 | 2.6 | 4.21 | 1.03 | 5.83 | 9.15 | 7.74 | 13.86 | 3.1 | 1.51 | 2.93 | 6.92 | 7.35 | 8.62 | 80.23 |  |
| Site 4 | 16.95 | 1.57 | 5.45 | 7.05 | 7.54 | 2.45 | 6.9 | 9.32 | 2.26 | 1.67 | 4.03 | 4.68 | 6.32 | 6.72 | 2.58 | 10.3 | 95.79 |  |
| Site 5 | 4.03 | 0.1 | 1.35 | 1.1 | 3.08 | 0.64 | 6.68 | 7.67 | 2.06 | 1.98 | 0.63 | 4.98 | 5.09 | 6.9 | 2.39 | 11.1 | 59.78 |  |
| Site 6 | 5.54 | 4.46 | 5.32 | 6.16 | 30.39 | 6.22 | 68.93 | 52.45 | 87.25 | 103.16 | 18.91 | 11.63 | 19.2 | 6.98 | 4.32 | 88.24 | 519.16 |  |
| Site 7 | 0.97 | 1.26 | 3.07 | 7.41 | 19.21 | 2.57 | 53.65 | 50.53 | 37.94 | 19.55 | 3.59 | 1.84 | 4.31 | 6.83 | 1.23 | 16.55 | 230.51 |  |
| Site 8 | 1.34 | 2.94 | 5.76 | 6.67 | 12.78 | 1.95 | 42.68 | 41.72 | 37.25 | 17.49 | 2.7 | 4.16 | 3.98 | 6.61 | 3.52 | 12.66 | 204.21 |  |
| Site 9 | 1.23 | 3.96 | 7.31 | 10.36 | 22.71 | 13.17 | 77.76 | 68.32 | 55.54 | 20.47 | 7.56 | 3.14 | 5.88 | 6.6 | 3.82 | 14.83 | 322.66 |  |
| Site 10 | 5.75 | 6.16 | 62.12 | 34.02 | 79.6 | 25.99 | 254.89 | 197.66 | 28.43 | 76.35 | 8.98 | 1.7 | 5.77 | 6.62 | 12.36 | 10.22 | 816.62 |  |
| Site 11 | 2.98 | 2.17 | 5.82 | 6.71 | 7.87 | 2.53 | 18 | 10.25 | 21.1 | 13.49 | 1.95 | 1.41 | 2.6 | 6.52 | 1.63 | 11.71 | 116.74 |  |
| Site 12 | 8.04 | 2.61 | 11.22 | 11.11 | 15.31 | 6.39 | 40.03 | 34.81 | 19.59 | 12.08 | 1.74 | 1.67 | 1.92 | 6.49 | 1.36 | 12.08 | 186.45 |  |
| Site 1 | 5471.03 | 362.74 | 596.65 | 1375.19 | 6088.85 | 1417 | 4561.28 | 2445.43 | 551.79 | 810.02 | 419.74 | 81.47 | 201.04 | 247.71 | 123.41 | 110.06 | 24863.41 | 55 |
| Site 2 | 2243.57 | 280.64 | 864.54 | 1294.31 | 5212.3 | 1421.04 | 4118.1 | 3105.03 | 581.22 | 729.51 | 400.02 | 52.02 | 210.79 | 224.46 | 69.23 | 90.77 | 20897.55 |  |
| Site 3 | 902.07 | 360.77 | 800.8 | 1329.11 | 5535.47 | 1450.31 | 3708.63 | 2800.96 | 582.73 | 655.46 | 337.02 | 59.24 | 210.95 | 226.81 | 90.81 | 107.4 | 19158.54 |  |
| Site 4 | 3110.92 | 391.17 | 432.07 | 862.14 | 2534.39 | 831.89 | 2619.07 | 1611.62 | 331.94 | 470.52 | 257.72 | 37.61 | 193.92 | 148.27 | 60 | 106.31 | 13999.56 |  |
| Site 5 | 186.09 | 59.62 | 100.28 | 46.69 | 51.44 | 48.7 | 108.77 | 72.34 | 10.33 | 9.49 | 3.47 | 0.15 | 0.79 | 8.97 | 12.55 | 55.12 | 774.8 |  |
| Site 6 | 211.98 | 41.52 | 72.33 | 100.22 | 38.51 | 34.78 | 175.59 | 47.27 | 1.66 | 0.85 | 3.47 | 0.98 | 0.37 | 17.52 | 54.84 | 35.15 | 837.04 |  |
| Site 7 | 296.21 | 70.18 | 30.02 | 77.21 | 44.32 | 53.2 | 105.83 | 49.17 | 10.18 | 8.11 | 1.4 | 0.35 | 1.75 | 14.52 | 16.34 | 13.62 | 792.41 |  |
| Site 8 | 306.47 | 88.22 | 14.46 | 81.13 | 52.16 | 48.26 | 165.61 | 52.85 | 12.27 | 9.47 | 0.82 | 0.65 | 0.36 | 9.11 | 7.51 | 8.45 | 857.8 |  |
| Site 9 | 451.12 | 102.66 | 318.98 | 285.43 | 117.83 | 54.54 | 371.02 | 180.31 | 20.52 | 14.66 | 6.93 | 0.99 | 0.16 | 9.69 | 45.33 | 6.74 | 1986.91 |  |
| Site 10 | 854.31 | 227.01 | 280.5 | 906.3 | 1062.44 | 983.09 | 1522.74 | 967.02 | 67.67 | 56.6 | 25.02 | 10.75 | 11.56 | 16.74 | 25.55 | 95.74 | 7113.04 |  |
| Site 11 | 1302.51 | 242.49 | 55.91 | 572.12 | 186.2 | 78.95 | 1065.49 | 683.45 | 42.36 | 26.02 | 21.52 | 11.51 | 8.99 | 13.27 | 27.26 | 85.33 | 4423.38 |  |
| Site 12 | 235.12 | 132.14 | 47.56 | 25.65 | 80.53 | 38.32 | 134 | 79.11 | 6.51 | 4.02 | 2.46 | 6.23 | 4.51 | 10.12 | 18.02 | 62.35 | 886.65 |  |
| Site 1 | 8.8 | 1 | 1.6 | 7.3 | 33.2 | 0.8 | 60.6 | 33.9 | 25 | 44.9 | 47.7 | 36.4 | 30.5 | 37 | 7.6 | 35.6 | 411.9 | 56 |
| Site 2 | 1.34 | 0.6 | 0.7 | 4.8 | 35.1 | 0.6 | 77 | 48.6 | 30.8 | 54 | 51.3 | 38.3 | 30.3 | 29.1 | 4.8 | 27.6 | 434.94 |  |
| Site 3 | 8.9 | 1.1 | 2 | 8.2 | 39 | 1.3 | 93.6 | 33.6 | 42.4 | 70 | 66.6 | 49.9 | 45.9 | 53.3 | 10 | 50.3 | 576.1 |  |
| Site 4 | 0.1 | 0.4 | 0.7 | 5.2 | 31.5 | 0.9 | 58.2 | 20.5 | 15.2 | 25.7 | 18.3 | 14.6 | 11 | 9 | 1.9 | 9.8 | 223 |  |
| Site 5 | 3.6 | 2.2 | 3.3 | 10.8 | 70 | 3.1 | 136.3 | 32 | 86.7 | 117.3 | 123.9 | 104.5 | 119 | 112.7 | 28.1 | 119.2 | 1072.7 |  |
| Site 6 | 27.1 | 3.3 | 5.8 | 20.2 | 84.7 | 3.4 | 209.5 | 35.1 | 143.7 | 174.9 | 202.8 | 152.3 | 185.5 | 191.9 | 45.5 | 183.6 | 1669.3 |  |
| Site 7 | 10.2 | 4 | 4.1 | 18 | 80.4 | 18.5 | 195.7 | 142.7 | 107.7 | 169.2 | 177.4 | 135.8 | 128.6 | 158.7 | 36.6 | 148.9 | 1536.5 |  |
| Site 8 | 5.9 | 2.3 | 2.6 | 12.2 | 58.3 | 12.4 | 140.8 | 88.7 | 73.4 | 117.4 | 139.8 | 98.9 | 95.4 | 123 | 25.2 | 112.1 | 1108.4 |  |
| Site 9 | 11.4 | 1.7 | 3.4 | 12.8 | 52.2 | 1.6 | 148.3 | 37 | 54.6 | 86.8 | 70.3 | 57 | 43.8 | 45.4 | 9.6 | 45 | 680.9 |  |
| Site 10 | 9 | 1 | 2.1 | 7.1 | 34.8 | 0.6 | 81.6 | 32.4 | 36.7 | 55.5 | 54.1 | 43.3 | 41.2 | 35.7 | 7.1 | 35.8 | 478 |  |
| Site 11 | 5.4 | 1.1 | 2.2 | 9.8 | 50.3 | 0.8 | 91.9 | 26.5 | 24.7 | 43.6 | 27.5 | 22.8 | 17.2 | 12.6 | 2.5 | 13.4 | 352.3 |  |
| Site 12 | 2 | 1.2 | 3.2 | 12.8 | 53.2 | 1.4 | 117.1 | 32.1 | 40.7 | 65.3 | 50.6 | 40.5 | 31.1 | 22.8 | 5.2 | 19.9 | 499.1 |  |
| Site 13 | 9.5 | 1.8 | 4.2 | 13.6 | 61.1 | 1.9 | 123 | 32.2 | 59.8 | 84.5 | 82.1 | 65.6 | 63.8 | 65.3 | 15.1 | 69 | 752.5 |  |
| Site 14 | 0.3 | 0.7 | 0.7 | 6.7 | 42 | 0.8 | 110.9 | 30.7 | 39.4 | 66.3 | 73 | 57.1 | 48.7 | 53.8 | 10.7 | 55.3 | 597.1 |  |
| Site 15 | 0.5 | 0.8 | 0.8 | 5.5 | 25.2 | 0.5 | 51.6 | 28.4 | 17.4 | 33.8 | 39.6 | 29.1 | 23.7 | 32.2 | 6 | 35 | 330.1 |  |
| Site 16 | 8.3 | 0.7 | 1.2 | 6.7 | 28.8 | 0.6 | 63.8 | 27.5 | 23.3 | 40.7 | 50.6 | 34.7 | 27.2 | 32 | 6 | 33 | 385.1 |  |
| Site 17 | 5.5 | 0.6 | 1 | 5.6 | 27.8 | 0.5 | 44.6 | 21.1 | 14.3 | 27.6 | 29.2 | 22.8 | 16.8 | 21.4 | 4.1 | 22.1 | 265 |  |
| Site 18 | 0.1 | 0.2 | 0.2 | 2.4 | 20.3 | 0.4 | 47.9 | 26.6 | 17.6 | 33.3 | 33.9 | 23.8 | 20.1 | 21.1 | 4.3 | 20.6 | 272.8 |  |
| Site 19 | 11.4 | 2.5 | 1.4 | 8.8 | 56.1 | 9.7 | 138.4 | 93.6 | 38.9 | 96.5 | 89.9 | 86.2 | 54.4 | 58.6 | 11 | 62.6 | 820 |  |
| Site 20 | 5 | 0.7 | 0.7 | 4 | 24.5 | 0.3 | 53.8 | 34.8 | 18.5 | 39.1 | 39.5 | 25.7 | 17.2 | 20.6 | 4 | 20 | 308.4 |  |
| Site 21 | 1.6 | 0.4 | 0.34 | 2.8 | 14.2 | 0.2 | 32 | 32.6 | 10.2 | 22.8 | 24.8 | 14.8 | 9.2 | 13.6 | 2.2 | 13.1 | 194.84 |  |
| Site 22 | 0.2 | 0.6 | 0.6 | 5.2 | 23.8 | 0.4 | 55.6 | 28.9 | 17.6 | 33.7 | 31.2 | 22.5 | 14.8 | 20.1 | 3.6 | 20.3 | 279.1 |  |
| Site 23 | 9.3 | 1.5 | 5.9 | 7.4 | 30.7 | 5.7 | 63 | 41.9 | 17.8 | 36.1 | 37.1 | 26.4 | 20.5 | 26 | 6.3 | 25 | 360.6 |  |
| Site 24 | 1.7 | 0.9 | 3 | 12.4 | 47.4 | 8.5 | 24.7 | 18.9 | 42.8 | 19.4 | 12.6 | 16.3 | 12.8 | 11.1 | 6.8 | 10.8 | 250.1 |  |
| Site 25 | 8 | 0.7 | 0.9 | 4.5 | 24 | 0.5 | 65.1 | 36 | 24.7 | 36.9 | 25.8 | 19.8 | 15.2 | 12.1 | 1.89 | 12.4 | 288.49 |  |
| Site 26 | 8.2 | 0.7 | 0.9 | 4.8 | 26.2 | 0.4 | 54.6 | 33.7 | 17.5 | 36.8 | 39 | 25 | 18.2 | 27 | 4.2 | 25.2 | 322.4 |  |
| Site 27 | 1.6 | 0.5 | 0.8 | 3.6 | 20.2 | 0.3 | 36.1 | 25.2 | 8.9 | 19.3 | 18.5 | 12 | 10 | 10 | 1.8 | 10.7 | 179.5 |  |
| Site 28 | 4.3 | 0.7 | 0.7 | 3.8 | 21 | 0.4 | 41.9 | 37.5 | 14.8 | 31.3 | 37.2 | 23.4 | 18.4 | 21.6 | 3.8 | 23.5 | 284.3 |  |
| Site 1 | 72.9 | nd | 25.7 | nd | 37.4 | 9.2 | 31.6 | 31.1 | 137 | 10.7 | 13.1 | 13.1 | nd | nd | nd | nd | 400 | 57 |
| Site 2 | 12.5 | 9.5 | 17.4 | 4.2 | 13.7 | 3.6 | 29 | 36 | 227 | 14 | 30 | 27 | 56 | 12 | 3 | 22 | 525 |  |
| Site 3 | 13.5 | 4.4 | 10 | 9.5 | 20.8 | 6.3 | 86.1 | 81 | 212 | 36.7 | 256 | 43.6 | 75.4 | 12 | nd | 30.7 | 901 |  |
| Site 4 | 5.3 | 2.5 | 4.3 | 16 | 269 | 18.1 | 30.6 | 362 | 451 | 91.5 | 306 | 58.8 | 77.8 | 127 | 420 | 332 | 2585 |  |
| Site 5 | 1.8 | nd | 12.1 | nd | 120 | 34.7 | 77.9 | 74.6 | 288 | 31.6 | 108 | 51.3 | 33.4 | 7.8 | nd | 39.6 | 886 |  |
| Site 6 | 20.3 | 4.2 | 21 | 24 | 164 | 12.3 | 237 | 287 | 256 | 133 | 220 | 101 | 113 | 47.5 | 135 | 93.2 | 1876 |  |
| Site 7 | 14.2 | 8.3 | 20.1 | 9 | 60.3 | 23.9 | 76.5 | 146 | 369 | 23 | 286 | 70.5 | 89.4 | 47.2 | 9.1 | 37.5 | 1310 |  |
| Site 8 | 7.4 | 4.4 | 9.3 | 1.9 | 15.8 | 17.9 | 102 | 93.6 | 311 | 31 | 16.5 | 151 | 126 | 82.8 | 21.8 | 147 | 1151 |  |
| Site 9 | 6.9 | nd | 14 | 3.3 | 63.1 | 3.8 | 117 | 116 | 268 | 31.7 | 238 | 43.1 | 71.9 | 61.3 | 2.3 | 52.3 | 1101 |  |
| Site 10 | 44.9 | 6.6 | 25 | 10.7 | 91.6 | 4.3 | 136 | 161 | 105 | 63.2 | 277 | 48.3 | 91.1 | 42.6 | 21.5 | 80.9 | 1211 |  |
| Site 11 | 40.7 | 4.9 | 27.7 | 17.1 | 114 | 30.9 | 98.6 | 117 | 258 | 24.3 | 273 | 43.9 | 74.2 | 16.9 | nd | 40.4 | 1192 |  |
| Site 12 | 42.2 | 0.6 | 19.1 | 2.7 | 189 | 50.5 | 125 | 178 | 406 | 24.2 | 250 | 55.7 | 74.8 | 19 | nd | 78.3 | 1518 |  |
| Site 13 | 34.4 | 5.5 | 7.7 | 10.3 | 55.5 | 6.1 | 120 | 208 | 362 | 25.4 | 221 | 48.9 | 67.7 | 20.4 | nd | 73.2 | 1270 |  |
| Site 14 | 36.7 | nd | 16.4 | nd | 384 | 86.5 | 201 | 305 | 205 | 22.8 | 105 | 13.6 | 40.3 | 7.5 | nd | 4.4 | 1428 |  |
| Site 15 | 23 | nd | 7.9 | nd | 51.2 | 28.3 | 64.4 | 116 | 119 | 23.3 | 78.4 | 15.7 | 8.6 | nd | nd | nd | 536 |  |
| Site 16 | 5.5 | nd | 10.5 | nd | 166 | 73.1 | 689 | 164 | 461 | 15.6 | 126 | 25.1 | 20.7 | nd | nd | 15.1 | 1785 |  |
| Site 17 | 317 | 23.5 | 130 | 36.1 | 1511 | 487 | 3566 | 1187 | 2883 | 112 | 686 | 164.3 | 306 | 185 | 81.3 | 198 | 11902 |  |
| Site 18 | 565 | 110 | 314 | 79.5 | 1203 | 248 | 2962 | 1098 | 4176 | 98.9 | 2657 | 369 | 951 | 827 | 249 | 793 | 16700 |  |
| Site 19 | 345 | nd | 132 | nd | 87.5 | 19.2 | 87.9 | 145 | 146 | 19.3 | 54.9 | 13.2 | 50.3 | nd | nd | nd | 1134 |  |
| Site 20 | 10.5 | nd | 14.5 | nd | 6.1 | 55.5 | 38.1 | 147 | 6.9 | 1.1 | nd | nd | 46.4 | nd | nd | nd | 326 |  |
| Site 21 | 4.5 | nd | 0.9 | nd | 8.3 | 2.7 | 11.4 | 14.2 | nd | 25.5 | 15.3 | 12.7 | 2.2 | nd | nd | nd | 98 |  |
| Site 22 | 5.2 | nd | nd | nd | 13.6 | 2.7 | 6.8 | 24.8 | 99.8 | 10 | 25.9 | 16.4 | 23.2 | nd | nd | nd | 238 |  |
| Site 23 | 15.7 | 5.3 | 4.9 | 4.5 | 51.1 | 6.6 | 65.6 | 96.7 | 179 | 16.3 | 238 | 42.2 | 105 | 48 | nd | 90.6 | 976 |  |
| Site 24 | 45.3 | 6.4 | 6.4 | 17.3 | 121 | 13.9 | 198 | 233 | 111 | 196 | 489 | 151 | 168 | 141 | 20.1 | 112 | 2063 |  |
| Site 25 | 4.6 | 2.4 | 2.3 | 0.6 | 24.5 | 3.6 | 40.2 | 60.3 | 146 | 66.9 | 216 | 65.8 | 48.1 | 13.3 | nd | 38 | 735 |  |
| Site 26 | 59.8 | 4.8 | 15.1 | 31.7 | 114 | 11.3 | 104 | 125 | 156 | 178 | 71.9 | 44.5 | 43.8 | 14.2 | 20.5 | 5.6 | 1040 |  |
| Site 27 | 2.4 | nd | nd | 1.1 | 15.7 | 2.4 | 14.9 | 19.4 | 19 | 14.3 | 136 | 12.5 | 30.6 | 8.1 | nd | 21.2 | 302 |  |
| Site 28 | 9.2 | 2.4 | 3.8 | 5.8 | 37.3 | 4.4 | 45.8 | 45.4 | 125 | 52.7 | 201 | 62.7 | 48.6 | 18.8 | nd | 43.7 | 711 |  |
| Site 29 | 15.1 | 4.7 | 5.2 | 8 | 35.5 | 5.5 | 49.7 | 58.9 | 148 | 87.8 | 270 | 84.5 | 104 | nd | 6.9 | 54.6 | 948 |  |
| Site 30 | 21.5 | 7.8 | 7.7 | 6.7 | 56.6 | 7.2 | 73 | 92.7 | 245 | 94.6 | 285 | 92.8 | 78.6 | 46.3 | nd | 79.5 | 1209 |  |
| Site 31 | 28.3 | 1.9 | 8.2 | 8.8 | 53.8 | 6.8 | 46.5 | 52.5 | 50.7 | 49.3 | 209 | 65.2 | 39.5 | 11.1 | 12.3 | 31.9 | 688 |  |
| Site 32 | 7.4 | 0.5 | 5.7 | 3.6 | 24.7 | 2.5 | 27.6 | 30.2 | 49.3 | 37.3 | 54.6 | 17 | 29.1 | nd | nd | 4.5 | 300 |  |
| Site 33 | 11.2 | nd | 3.8 | 1.7 | 21.4 | 15.4 | 19.6 | 21 | 50.1 | 25.1 | 38.5 | 12.2 | 24.7 | nd | nd | nd | 253 |  |
| Site 34 | 23 | nd | 5.8 | 5.1 | 38.7 | 18.9 | 35 | 42 | 47.7 | 34 | 72.8 | 19.2 | 39 | nd | nd | nd | 391 |  |
| Site 35 | 3.7 | nd | 3.2 | nd | nd | nd | nd | nd | 8.8 | 3.9 | 7.5 | 2.5 | 4.2 | nd | nd | nd | 34 |  |
| Site 36 | 21.5 | 2.3 | 8.7 | 4 | 39.3 | 13.5 | 36.1 | 35.1 | 79.1 | 35.7 | 3.2 | 24.3 | 6.7 | nd | nd | 4.5 | 324 |  |
| Site 37 | 295 | 55.6 | 193 | 163 | 598 | 217 | 660 | 567 | 1332 | 570 | 2094 | 650 | 616 | 321 | 102 | 352 | 8904 |  |
| Site 38 | 2.3 | nd | nd | nd | 10.1 | 5.8 | 3.7 | 12.1 | 25.4 | 12.8 | 3.5 | 9.2 | 9.5 | nd | nd | nd | 94 |  |
| Site 39 | 35.8 | 1.4 | 1.8 | 1.4 | 56.5 | 40.2 | 44.6 | 39.9 | 57.9 | 27.6 | 5.4 | 27.9 | 21.9 | nd | nd | nd | 375 |  |
| Site 40 | 13.3 | 7.8 | 3.8 | nd | 59.7 | 41.7 | 160 | 165 | 534 | 87.6 | 133 | 46 | 60.7 | 25 | nd | 31.4 | 1373 |  |
| Site 41 | 11.6 | nd | nd | nd | 61.3 | 22.7 | 21.2 | 31.7 | 133.4 | 11.5 | 1.4 | 0.8 | 21.7 | nd | nd | nd | 327 |  |
| Site 42 | 0.68 | 1.2 | 0.07 | 0.12 | 1.1 | 0.12 | 4.4 | 19.6 | 6.5 | 3.6 | 30.1 | 4.6 | 15.1 | 11.2 | 3 | 15.1 | 117 |  |
| Site 43 | 13.8 | nd | 2.7 | nd | 16.9 | 10.9 | 26.2 | 43.7 | 60.9 | 25.3 | 52.1 | 7.8 | 9.3 | nd | nd | nd | 278 |  |
| Site 44 | 64.9 | 0.8 | nd | 10.3 | 115.3 | 28.2 | 146 | 190 | 90.5 | 63.1 | 104 | 64.7 | 76.1 | 12.3 | 141 | 71.6 | 1196 |  |
| Site 45 | 17.5 | 5.5 | nd | nd | 53.3 | 14.9 | 126 | 551 | 367 | 357 | 1007 | 97.9 | 308 | 166 | 51.6 | 170 | 3307 |  |
| Site 46 | 79.9 | 9.6 | 12.7 | 4.8 | 200 | 33.8 | 172 | 223 | 334 | 38.8 | 221 | 22.5 | 23.5 | 8 | 9.6 | 18.4 | 1425 |  |
| Site 47 | nd | nd | nd | nd | 62.2 | 18 | 31.5 | 57.2 | 120 | 4.8 | 19.9 | 13.2 | 8 | nd | nd | nd | 335 |  |
| Site 48 | 20.6 | 3 | nd | 4.8 | 167.7 | 23.6 | 162 | 229 | 479 | 25.9 | 174 | 22.4 | 57.1 | 19.6 | nd | 43.2 | 1432 |  |
| Site 49 | 8.3 | nd | nd | nd | 32.5 | 15.7 | 17.3 | 30.6 | 74.3 | 6.8 | 29.4 | 5.2 | 12.8 | nd | nd | nd | 233 |  |
| Site 50 | 61 | 9.1 | 22.7 | 10.4 | 72.2 | 48.8 | 91.5 | 82.9 | 46.4 | 50.4 | 230 | 27.1 | 62.1 | 45.6 | 13.2 | 60.2 | 947 |  |
| Site 51 | 19.9 | nd | 26 | nd | 27.1 | 27.8 | 16 | 35.3 | 98.1 | 20.6 | 11.7 | 1.8 | 16.4 | nd | nd | nd | 317 |  |
| Site 52 | 43.6 | 3.6 | nd | 1 | 41.8 | 38 | 62.2 | 38.7 | 166 | 18.8 | 148 | 21.3 | 38.1 | 33.5 | nd | 6.2 | 675 |  |
| Site 53 | 22.9 | nd | nd | nd | 63.7 | 24.4 | 23.4 | 40.1 | 108.9 | 14.7 | 30.4 | 3.4 | 45 | nd | nd | nd | 380 |  |
| Site 54 | 77.2 | nd | nd | nd | 49.3 | 20.2 | 61.7 | 81.2 | 95.3 | 21.6 | 3 | 0.7 | 2.1 | nd | nd | nd | 431 |  |
| Site 55 | 70.3 | 7 | nd | 6.6 | 98.1 | 166 | 313.5 | 94.1 | 603 | 23.3 | 72.6 | 56 | 34.6 | 48.7 | nd | 60.4 | 1672 |  |
| Site 56 | 16.5 | nd | nd | nd | 26.8 | 10.2 | 30.6 | 12.4 | 81.1 | 16.7 | 3.3 | 1.2 | 20.6 | nd | nd | nd | 219 |  |
| Site 57 | 350 | 30.3 | 94.7 | 94.6 | 680 | 319 | 319.7 | 484 | 161 | 177 | 736 | 76.4 | 236 | 74.4 | 12.5 | 139 | 4127 |  |
| Site 58 | 31.1 | 7.8 | 6.7 | 10.4 | 62.9 | 11.5 | 98.4 | 145 | 232 | 87.4 | 148 | 23.9 | 29.7 | 10.5 | nd | 58.7 | 973 |  |
| Site 59 | 41.3 | nd | 8.6 | 2.5 | 20.1 | 27.8 | 68.3 | 119 | 46.4 | 41.2 | 181 | 38.9 | 38.1 | nd | nd | 14.8 | 674 |  |
| Site 60 | 6.7 | nd | nd | nd | nd | 27.8 | 8.5 | 9.7 | nd | nd | 4.6 | 1.4 | 11.3 | nd | nd | nd | 70 |  |
| Site 61 | 25.3 | 8.1 | nd | nd | 66.6 | 25 | 254.2 | 258.4 | 455 | 45.8 | 22.4 | 7.8 | 48.3 | nd | nd | nd | 1243 |  |
| Site 62 | 9.9 | nd | nd | nd | 21.5 | 36.5 | 24.1 | 64.8 | 196 | 24 | 203 | 22.5 | 37.4 | nd | nd | 41.1 | 680 |  |
| Site 63 | 1.4 | nd | nd | nd | 6.9 | 28.3 | 67.8 | 121.1 | 174 | 38.7 | 291 | 73.5 | 68 | nd | nd | 162.5 | 1035 |  |
| Site 64 | 118 | 11.7 | 4.9 | 18.8 | 232 | 115 | 332 | 367 | 450 | 72.9 | 519 | 42.3 | 152 | 34.5 | 41.1 | 86.8 | 2624 |  |
| Site 65 | 13.4 | nd | nd | 12.2 | 35.5 | 8.7 | 66.6 | 148.7 | 108 | 48.9 | 279 | 24.3 | 24.1 | 22.7 | nd | 26.4 | 826 |  |
| Site 66 | 37.7 | 10.1 | 13.6 | 29.4 | 149 | 38.7 | 215 | 220 | 168 | 143 | 293 | 27.9 | 57.4 | 18 | nd | 39.1 | 1511 |  |
| Site 67 | 13.4 | nd | nd | nd | 33.4 | 45.2 | 95.7 | 81.1 | 301 | 10 | 88 | 8.4 | 12.6 | nd | nd | 11 | 703 |  |
| Site 68 | 4.2 | nd | nd | nd | nd | nd | nd | nd | 228 | 6 | 2.4 | 0.9 | 5 | nd | nd | nd | 247 |  |
| Site 69 | 22.7 | nd | 12.6 | 1.5 | 66.5 | 48.1 | 76.7 | 204 | 356 | 32.3 | 108 | 14.5 | 30.5 | 17.6 | 3.3 | 21.4 | 1018 |  |
| Site 70 | 14.6 | nd | 4 | 2.4 | 35.9 | 7.3 | 69.9 | 101 | 219 | 21.6 | 77.7 | 35 | 54.3 | 54.8 | 4.1 | 5.6 | 710 |  |
| Site 71 | 26.2 | nd | nd | nd | 12.1 | 46.8 | 49.5 | 112 | 237 | 20.2 | 16.2 | 5.1 | 4 | nd | nd | nd | 529 |  |
| Site 72 | 42.5 | 1.3 | nd | nd | 11.4 | 31.3 | 138 | 172 | 160 | 45.4 | 166 | 23.2 | 6.8 | nd | nd | 2.6 | 812 |  |
| Site 73 | 0.1 | nd | nd | nd | 3.8 | 1.5 | nd | 49.3 | 216 | 13.9 | 10.6 | 3.7 | 3.6 | nd | nd | nd | 302 |  |
| Site 74 | 12.3 | nd | 1.5 | nd | 7.7 | 73.5 | 154 | 217 | 263 | 26.9 | 196 | 33.8 | 25.1 | nd | nd | nd | 1021 |  |
| Site 75 | 10.4 | nd | nd | nd | nd | nd | nd | nd | nd | nd | 4.5 | 1.3 | 28.3 | nd | nd | nd | 45 |  |
| Site 76 | 14.6 | nd | nd | nd | 15.2 | 3.9 | 15 | 21.5 | 43.9 | 4.7 | 2.5 | 1.2 | 15.7 | nd | nd | nd | 144 |  |
| Site 77 | 274 | 9.6 | 59.7 | 64.1 | 13.6 | 215 | 341 | 404 | 555 | 57.9 | 709 | 55.1 | 195 | 39.8 | nd | nd | 3076 |  |
| Site 78 | 62.9 | nd | nd | nd | 93.3 | 35.8 | 55.7 | 69.4 | 158 | 33.8 | 11.8 | 6.3 | 98.4 | nd | nd | nd | 651 |  |
| Site 79 | 8.9 | nd | nd | nd | 110 | 42.1 | 53.8 | 53.2 | nd | nd | 13.5 | 6 | 10.4 | nd | nd | nd | 298 |  |
| Site 1 | / | / | / | 4.51 | 23.1 | 3.75 | 34.1 | 17.1 | 9.98 | / | 11.3 | 0.08 | 5.6 | 6.79 | 7.54 | 9.1 | 133 | 12 |
| Site 2 | / | / | / | 1.27 | 8.44 | nd | 4.4 | 2.72 | 1.84 | / | 1.08 | 0.97 | 0.8 | 3.87 | 3.08 | 2.5 | 31 |  |
| Site 3 | / | / | / | 12.5 | 40.6 | 16.5 | 4.27 | 2.08 | 1.25 | / | 0.7 | 0.4 | 0.55 | nd | nd | nd | 78.9 |  |
| Site 4 | / | / | / | 18 | 48 | 11.6 | 4.93 | 3.09 | 1.45 | / | 1.32 | 0.7 | 1.09 | 8.41 | 2.48 | 1.6 | 103 |  |
| Site 5 | / | / | / | 17.9 | 60.6 | 15.3 | 4.3 | 2.59 | 1.71 | / | 0.8 | 0.63 | 1.19 | 5.75 | 3.16 | 2.95 | 117 |  |
| Site 6 | / | / | / | 3.44 | 15.2 | 2.37 | 3.45 | 2.82 | 2.03 | / | 1.68 | 1.07 | 1.31 | 5.81 | 2.48 | 2.06 | 43.7 |  |
| Site 7 | / | / | / | 15.3 | 114 | 13.9 | 62.1 | 81.8 | 124 | / | 191 | 43.1 | 33.4 | 53.1 | 41.4 | 130 | 903 |  |
| Site 8 | / | / | / | 4.29 | 3.08 | 0.5 | 2.82 | 3.36 | 1.05 | / | 1.15 | 0.3 | 0.43 | nd | nd | nd | 17 |  |
| Site 9 | / | / | / | 20 | 53.6 | 14 | 25.1 | 11.3 | 4.15 | / | 2.31 | 2.69 | 2.05 | 20.4 | 2.49 | 3.75 | 162 |  |
| Site 10 | / | / | / | 19.5 | 107 | 8.26 | 35.2 | 34.2 | 25.5 | / | 34.9 | 15.3 | 14.5 | 67.1 | 16.5 | 23.6 | 401 |  |
| Site 11 | / | / | / | 1.48 | 3.45 | 0.4 | 3.49 | 1.87 | 1.41 | / | 0.75 | 0.72 | 1.13 | 1.89 | 2.25 | 1.67 | 20.5 |  |
| Site 1 | nd | 12.7 | 4.08 | 19.26 | 48.93 | 2.04 | 3.07 | 2.88 | 0.49 | 0.91 | 0.17 | / | nd | nd | / | / | 94.53 | 16 |
| Site 2 | 32.96 | 12 | 2.55 | 38.47 | 57.98 | 5.09 | 9.11 | 9.6 | 1.62 | 9.86 | 2.81 | / | 14.28 | 0.66 | / | / | 196.99 |  |
| Site 3 | 4.71 | 6.4 | 0.67 | 17.16 | 21.71 | 4.41 | 2.86 | 2.95 | nd | 0.69 | 0.35 | / | 5.39 | nd | / | / | 67.29 |  |
| Site 1 | 33.08 | 8.11 | 5.35 | 25.07 | 89.97 | 21.05 | 124.50 | 95.98 | 66.91 | 86.12 | 110.43 | 39.82 | 66.43 | 57.31 | 15.81 | 58.87 | 904.81 | 13 |
| Site 2 | 20.28 | 6.19 | 5.35 | 17.87 | 83.72 | 14.95 | 108.71 | 76.26 | 55.34 | 69.44 | 93.78 | 35.32 | 56.03 | 49.12 | 14.68 | 52.06 | 759.08 |  |
| Site 3 | 13.40 | 2.83 | 3.11 | 8.13 | 38.85 | 9.05 | 56.61 | 41.14 | 28.12 | 28.88 | 44.54 | 19.82 | 25.64 | 22.43 | 10.81 | 25.17 | 378.53 |  |
| Site 4 | 9.86 | 4.22 | 7.86 | 20.30 | 133.56 | 34.31 | 218.08 | 153.42 | 119.86 | 160.10 | 189.88 | 60.98 | 102.73 | 79.02 | 20.36 | 82.90 | 1397.45 |  |
| Site 5 | 7.36 | 13.86 | 25.35 | 42.73 | 479.18 | 91.92 | 983.27 | 582.36 | 338.63 | 414.20 | 556.07 | 177.27 | 335.65 | 235.06 | 44.97 | 256.74 | 4584.60 |  |
| Site 6 | 6.20 | 4.94 | 4.46 | 16.71 | 80.17 | 18.78 | 109.07 | 70.68 | 50.03 | 62.94 | 87.55 | 33.10 | 52.69 | 46.92 | 14.07 | 49.40 | 707.70 |  |
| Site 7 | 6.36 | 2.52 | 2.99 | 6.94 | 38.91 | 9.61 | 65.09 | 45.21 | 36.70 | 33.67 | 59.31 | 23.88 | 34.87 | 28.51 | 11.91 | 31.76 | 438.23 |  |
| Site 8 | 11.78 | 8.27 | 9.89 | 46.01 | 355.81 | 502.10 | 1072.18 | 683.15 | 669.73 | 633.68 | 340.74 | 284.41 | 651.24 | 428.52 | 36.57 | 36.23 | 5770.30 |  |
| Site 9 | 9.96 | 8.67 | 9.46 | 32.57 | 126.75 | 54.01 | 210.42 | 163.65 | 108.01 | 148.42 | 181.45 | 58.72 | 101.59 | 84.79 | 20.74 | 87.29 | 1406.48 |  |
| Site 10 | 5.34 | 49.00 | 8.37 | 23.35 | 84.19 | 27.20 | 150.09 | 123.80 | 74.03 | 94.87 | 115.33 | 40.60 | 75.69 | 60.64 | 16.25 | 60.66 | 1009.40 |  |
| Site 1 | 198.3 | 47.32 | 77.71 | 353.61 | 309.71 | 63.6 | 389.21 | 449.5 | 268.94 | 330.24 | 371.53 | 97.05 | 294.43 | 220.23 | 67.48 | 289.69 | 3828.58 | 8 |
| Site 2 | 68.23 | 15.19 | 12.58 | 29.29 | 61.91 | 8.68 | 20.95 | 19.45 | 14.09 | 20.56 | 23.44 | 6.78 | 25.51 | 13.04 | 6.37 | 12.48 | 358.54 |  |
| Site 3 | 78.53 | 31.11 | 28.17 | 136.78 | 142.19 | 15.91 | 34.85 | 40.15 | 30.16 | 36.96 | 49.17 | 42.33 | 35.17 | 31.2 | 13.92 | 30.69 | 777.29 |  |
| Site 4 | 16.51 | 14.26 | 24.72 | 52.47 | 61.58 | 8.62 | 8.89 | 12.34 | 14.17 | 13.57 | 15.69 | 7.86 | 23.57 | 9.42 | 7.24 | 9 | 299.91 |  |
| Site 5 | 10.2 | 12.19 | 10.69 | 16.89 | 80.09 | 12.11 | 31.3 | 42.51 | 27.89 | 30.43 | 61.52 | 11.19 | 36.82 | 37.46 | 9.3 | 29.5 | 460.09 |  |
| Site 6 | 8.52 | 10.02 | 12.78 | 77.66 | 68.86 | 10.77 | 29.24 | 42.37 | 27.89 | 27.71 | 19.2 | 12.99 | 46.92 | 18.19 | 8.12 | 17.46 | 448.71 |  |
| Site 7 | 5.58 | 7.68 | 7.88 | 16.84 | 49.79 | 5.8 | 30.28 | 19.32 | 21.11 | 26.42 | 29.89 | 7.1 | 19.64 | 19.18 | 7.7 | 12.19 | 286.41 |  |
| Site 8 | 8.2 | 7.46 | 10.07 | 46.09 | 82.11 | 10.05 | 32.08 | 28.72 | 20.16 | 19.85 | 28.11 | 8.71 | 31.06 | 18.88 | 13.16 | 60.18 | 424.91 |  |
| Site 9 | 73.15 | 12.33 | 8.23 | 19.02 | 37.86 | 8.62 | 23.73 | 23.47 | 26.57 | 23.72 | 29.13 | 7.4 | 30.7 | 15.88 | 9.28 | 12.68 | 361.78 |  |
| Site 10 | 68.54 | 12.21 | 9.6 | 63.44 | 108.06 | 12.76 | 36.69 | 48.58 | 13.44 | 25.17 | 20.47 | 8.32 | 28.18 | 19.59 | 13.37 | 18.66 | 507.07 |  |
| Site 11 | 24.27 | 4.61 | 4.91 | 15.47 | 32.88 | 4.36 | 4.76 | 9.68 | 10.58 | 19.24 | 19.25 | 7.73 | 22.97 | 12.45 | 6.81 | 12.94 | 212.91 |  |
| Site 12 | 14.37 | 32.16 | 12.23 | 176.11 | 117.79 | 10.27 | 26.66 | 27.84 | 11.69 | 14.88 | 17.79 | 6.54 | 29.61 | 14.62 | 8.35 | 16.71 | 537.6 |  |
| Site 13 | 3.28 | 8.96 | 11.17 | 64.9 | 53.76 | 6.11 | 25.4 | 20.55 | 12.22 | 12.58 | 15.83 | 6.63 | 25.38 | 9 | 6.78 | 8.02 | 290.57 |  |
| Site 14 | 17 | 6.62 | 12.17 | 18.14 | 69.58 | 9.03 | 34.9 | 31.25 | 12.33 | 21.6 | 20.59 | 10.64 | 25.71 | 15.1 | 7.9 | 14.38 | 326.93 |  |
| Site 15 | 8.82 | 9.62 | 8.05 | 16.23 | 31.36 | 6.9 | 24.8 | 21.35 | 16.05 | 13.3 | 12.36 | 7.78 | 15.05 | 6.74 | 10.65 | 8.3 | 217.38 |  |
| Site 16 | 8.7 | 3.67 | 6.57 | 18.91 | 27.09 | 2.7 | 10.2 | 7.14 | 6.4 | 6.76 | 6.31 | 4.82 | nd | 4.32 | 6.38 | 6.1 | 126.08 |  |
| Site 17 | 3.06 | 4.81 | 8.33 | 11.83 | 28.29 | 3.93 | 12.44 | 9.72 | 5.54 | 5.72 | 8.36 | 4.63 | nd | 6.16 | nd | 6.66 | 119.49 |  |
| Site 1 | 8.7 | 2.7 | 1.7 | 7.3 | 68 | 0.4 | 8.4 | 11.4 | 1.4 | 3.2 | 2.1 | / | 0.8 | 1.5 | 0.3 | 1.4 | 119.3 | 3 |
| Site 2 | 8.3 | 1.6 | 2.3 | 6.4 | 51.9 | 4.8 | 29.5 | 39.1 | 11 | 18.6 | 17.4 | / | 12.6 | 14.1 | 2.7 | 19.4 | 239.6 |  |
| Site 3 | 8 | 3 | 3.1 | 15.4 | 97.9 | 6.6 | 34.4 | 43.3 | 11.7 | 22.7 | 16.7 | / | 11 | 13.6 | 2.9 | 21.2 | 311.6 |  |
| Site 4 | 6.8 | 3.7 | 3 | 6.7 | 61.4 | 6 | 39.4 | 50.8 | 10.9 | 24.9 | 18.3 | / | 13.7 | 15.3 | 3.5 | 23.8 | 288.3 |  |
| Site 5 | 5.7 | 2.9 | 2.4 | 7.1 | 62.9 | 5.6 | 36.8 | 45.5 | 19.9 | 19.9 | 17.7 | / | 13.5 | 14.2 | 3.1 | 21.8 | 271.8 |  |
| Site 6 | 1.6 | 1.7 | 1.2 | 3.9 | 55.3 | 2.9 | 31.7 | 37.4 | 14.2 | 14.2 | 13.6 | / | 10.8 | 13.8 | 2.4 | 13.8 | 214 |  |
| Site 7 | 9.1 | 2 | 2.4 | 7.6 | 41.3 | 4.3 | 26.2 | 29 | 15.9 | 15.9 | 15.2 | / | 8.6 | 11.3 | 1.8 | 15.6 | 197.9 |  |
| Site 8 | 6.7 | 2.1 | 1.9 | 5.3 | 51.6 | 4.1 | 29.6 | 36.8 | 20.2 | 20.2 | 15.1 | / | 11.2 | 11.6 | 1.9 | 16.7 | 222.3 |  |
| Site 9 | 2.7 | 0.8 | 0.7 | 2.5 | 20.4 | 1.8 | 8.2 | 9.1 | 4.5 | 4.5 | 3.2 | / | 1.2 | 2.3 | 0.2 | 2.2 | 61.7 |  |
| Site 10 | 3.3 | 0.6 | 0.8 | 0.7 | 19.9 | 1.9 | 10.1 | 14.2 | 5.7 | 5.7 | 5.2 | / | 2.9 | 3.9 | 0.4 | 4.2 | 76.6 |  |
| Site 11 | 3.2 | 1.6 | 1.3 | 2.9 | 39.8 | 2.9 | 18.6 | 20.8 | 9 | 9 | 10.1 | / | 8.8 | 8 | 1.2 | 8.9 | 142.3 |  |
| Site 12 | 6.3 | 1.9 | 1.9 | 3.1 | 43.8 | 3.1 | 21.2 | 27.7 | 12.9 | 12.9 | 9.9 | / | 6.3 | 5.6 | 1.1 | 10.2 | 160.8 |  |
| Site 13 | 16.96 | 2.6 | 3.6 | 9 | 56.7 | 4.5 | 28.2 | 32.6 | 18.3 | 18.3 | 14.3 | / | 8.2 | 9.2 | 1.6 | 14.2 | 227.6 |  |
| Site 14 | 1.3 | 1.5 | 1.1 | 2.9 | 14.1 | 1.2 | 5.1 | 5.1 | 3.5 | 3.5 | 3.6 | / | 2.6 | 4.1 | 1.3 | 2.9 | 53.4 |  |
| Site 15 | 8.3 | 2.2 | 2.2 | 6.3 | 58.6 | 4.5 | 26.4 | 42.6 | 18.3 | 18.3 | 17.8 | / | 10.4 | 12.3 | 1.9 | 17.5 | 238.7 |  |
| Site 16 | 6.8 | 1.6 | 1.2 | 3 | 25.7 | 2.1 | 13.6 | 34 | 9.3 | 9.3 | 7.3 | / | 4.2 | 5.9 | 1.2 | 7.7 | 128.3 |  |
| Site 17 | 12.3 | 1.8 | 2.8 | 8.7 | 59 | 5.9 | 26.8 | 29.3 | 16.7 | 16.7 | 13 | / | 8.7 | 10.1 | 2.1 | 13.9 | 220.8 |  |
| Site 18 | 4.6 | 2 | 1.4 | 3.2 | 33 | 2.5 | 18.7 | 33 | 11.1 | 11.1 | 9.2 | / | 5.3 | 7.3 | 1.5 | 10 | 149 |  |
| Site 19 | 3.7 | 1.1 | 1.2 | 3.1 | 33.7 | 3.1 | 18.6 | 20.2 | 9.3 | 9.3 | 7.7 | / | 4.5 | 5.7 | 1.2 | 7.3 | 125.7 |  |
| Site 20 | 7.3 | 2.2 | 2.4 | 7.3 | 162.3 | 3.7 | 30.8 | 63.8 | 17.8 | 17.8 | 14.2 | / | 8 | 10.7 | 2.4 | 16 | 357.1 |  |
| Site 21 | 5.5 | 2.1 | 1.2 | 4.1 | 21.3 | 2.8 | 14.8 | 15.7 | 10.9 | 10.9 | 8.2 | / | 6.3 | 7.6 | 2.5 | 8.6 | 117.1 |  |
| Site 22 | 9.4 | 1.3 | 1.5 | 6.8 | 69.6 | 2.1 | 22.1 | 47.2 | 12.2 | 12.2 | 10.6 | / | 5.8 | 9.2 | 1.7 | 9.9 | 215.4 |  |
| Site 23 | 1.8 | 0 | 0.4 | 1.5 | 17.5 | 0.7 | 5.1 | 5.8 | 4 | 4 | 4.4 | / | 6.9 | 4.9 | 0.5 | 5.1 | 60.4 |  |
| Site 24 | 156.3 | 11.7 | 15.2 | 40.5 | 208.1 | 34.9 | 209.2 | 252 | 119.7 | 119.7 | 78.3 | / | 55 | 47 | 10.8 | 65.8 | 1369.1 |  |
| Site 25 | 6.6 | 1.6 | 2.8 | 4.8 | 42.2 | 5.8 | 42.1 | 44.4 | 25.2 | 25.2 | 23 | / | 18.5 | 18.9 | 4.2 | 22.4 | 280.1 |  |
| Site 26 | 15.8 | 2.6 | 1.9 | 7.6 | 43 | 5 | 32.7 | 45.3 | 25 | 25 | 17.1 | / | 10.4 | 11.5 | 2 | 17.3 | 247.3 |  |
| Site 27 | 19.6 | 4.3 | 3.5 | 10.4 | 54.7 | 9.9 | 63.6 | 74.4 | 44.5 | 44.5 | 37 | / | 23.7 | 36.3 | 8.4 | 36.5 | 450.1 |  |
| Site 28 | 2.1 | 0.7 | 0.7 | 3.7 | 41.5 | 2.2 | 25.4 | 17 | 9.6 | 9.6 | 11.5 | / | 4.2 | 10.3 | 1.4 | 10.1 | 145.6 |  |
| Site 29 | 4.1 | 0 | 0.4 | 1.5 | 18.3 | 0 | 9.1 | 40.4 | 3.9 | 3.9 | 3.6 | / | 1.6 | 3.2 | 0.5 | 2.7 | 91.6 |  |
| Site 30 | 4.5 | 1.8 | 1.1 | 2.9 | 24.7 | 1.7 | 15.1 | 30.8 | 6.4 | 6.4 | 6.5 | / | 4.1 | 6.7 | 1.7 | 5.9 | 118.1 |  |
| Site 31 | 13.6 | 3.3 | 4.8 | 12.4 | 84.1 | 6.5 | 49.8 | 79.7 | 35 | 35 | 23 | / | 16.1 | 19.7 | 5.2 | 23.4 | 390.5 |  |
| Site 1 | 16.4 | 6.8 | 6.6 | 7.4 | 88.5 | 16.2 | 38.3 | 28.6 | 18.3 | 36 | 54.8 | 15.6 | 15.1 | 19.7 | 0.1 | 20.4 | 388.7 | 6 |
| Site 2 | 14.7 | 8.5 | 6.9 | 9.5 | 76.5 | 23 | 58.1 | 46.3 | 32.5 | 28.2 | 44.5 | 20.3 | 21.4 | 19 | 14.7 | 15.1 | 439.2 |  |
| Site 3 | 12.9 | 5.9 | 6.5 | 10.6 | 105.6 | 21.8 | 67.7 | 52.9 | 20.2 | 18.8 | 51.7 | nd | 18.5 | 9.8 | 14 | 15.5 | 432.4 |  |
| Site 4 | 5.1 | nd | nd | nd | 30.8 | 5.2 | 8.7 | 6.1 | 5.9 | 9.8 | 14.1 | nd | 13.9 | nd | nd | nd | 99.6 |  |
| Site 5 | 9.5 | nd | nd | 5.8 | 52.7 | 18.8 | 18.4 | 13.8 | 8.5 | 13.8 | 20.6 | nd | 4.5 | 5.7 | 7.8 | 9.3 | 199.2 |  |
| Site 6 | 10.1 | 8.5 | 0.1 | 5 | 64.9 | 21.1 | 37.7 | 21.8 | 19 | 11.7 | 60.5 | nd | 15.3 | 0.1 | 0.1 | 9.6 | 285.2 |  |
| Site 7 | 15.8 | 8.2 | 0.1 | 0.1 | 65.1 | 23.5 | 48.7 | 32.4 | 18.4 | 12.9 | 50.1 | 15 | 14.2 | 16.2 | 6.6 | 14.8 | 341.9 |  |
| Site 8 | 27.1 | 7.4 | 12.4 | 5.5 | 74.1 | 15.7 | 50.5 | 37.3 | 24.7 | 15.5 | 41.3 | 18.6 | 13 | 18.7 | 12.5 | 17.8 | 392.1 |  |
| Site 9 | 7.9 | 6.2 | 10.4 | 0.1 | 72.4 | 11.9 | 26.3 | 22.1 | 9.2 | 20 | 23.6 | 6.9 | 9.4 | 17.2 | 0.1 | 15.3 | 258.8 |  |
| Site 10 | 18.1 | 7.5 | 8.6 | 6.4 | 70.5 | 20.2 | 84.6 | 47.7 | 13.3 | 10.1 | 76.4 | 22.5 | 6.8 | 20.7 | 17.8 | 22.7 | 453.9 |  |
| Site 11 | 16.9 | 9.1 | 8.2 | 6.8 | 88.1 | 23.9 | 88.3 | 86.9 | 39.7 | 26.4 | 54.4 | 13.8 | 18 | 25.5 | 9.5 | 20.6 | 536.1 |  |
| Site 12 | 22.1 | 11.1 | 5.1 | 10.9 | 69.4 | 17.3 | 103.8 | 95.4 | 24.9 | 30.2 | 60.5 | 17.8 | 15.9 | 16.1 | 36.5 | 9.1 | 556.1 |  |
| Site 13 | 30.9 | 29.5 | 42.6 | 31.8 | 109.5 | 37 | 80.4 | 40.5 | 35 | 55.9 | 64.9 | 27.2 | 26 | 23.4 | 26.9 | 22.9 | 684.4 |  |
| Site 14 | 7.4 | 10.7 | 9.8 | 6.1 | 56.7 | 15.4 | 36.4 | 57.3 | 19.2 | 10.2 | 48.8 | 14.8 | 14.4 | 16.1 | 6.3 | 11.5 | 341.1 |  |
| Site 15 | 9.8 | 7.2 | 10.6 | 11.9 | 61.6 | 15 | 34.9 | 67.4 | 21.8 | 15.3 | 55.3 | 15.1 | 14 | 12.4 | 10.4 | 9.4 | 372.1 |  |
| Site 16 | 8 | 8 | 11.6 | 9.1 | 77.3 | 14.2 | 33.3 | 63.8 | 23.9 | 20.1 | 57.1 | 17.6 | 15.9 | 10.2 | 9.7 | 8.3 | 388.1 |  |
| Site 17 | 12.1 | 7.4 | 10.1 | 10.9 | 55.6 | 18.2 | 72.5 | 50.8 | 24.8 | 15 | 64.7 | 19.2 | 21.6 | 18.9 | 12.9 | 17.8 | 432.5 |  |
| Site 18 | 25.3 | 7.6 | 0.1 | 5.6 | 64.8 | 16.4 | 43.7 | 38.1 | 27.6 | 17.5 | 53.7 | 0.1 | 0.1 | 19.6 | 11.5 | 17.1 | 348.5 |  |
| Site 19 | 19.9 | 9.5 | 0.1 | 5.8 | 59.8 | 17.8 | 79 | 34.8 | 30.1 | 16.4 | 51.6 | 17.2 | 16.3 | 19.8 | 8.9 | 17.1 | 404 |  |
| Site 20 | 19.5 | 7.3 | 5.7 | 6.7 | 76.4 | 23.2 | 71.7 | 49.6 | 24.8 | 13.3 | 53.1 | 0.1 | 20.9 | 24.7 | 0.1 | 0.1 | 396.9 |  |
| Site 21 | 20.6 | 8.1 | 10.5 | 9.4 | 64.9 | 19.4 | 46.8 | 23.4 | 15.3 | 30.6 | 74.4 | 24.5 | 17.2 | 22.7 | 7.6 | 18.9 | 414.3 |  |
| Site 22 | 13.3 | 6.9 | 5.7 | 6.3 | 86 | 36.1 | 71.8 | 30.9 | 10.8 | 24.7 | 53.4 | 37.3 | 19.7 | 21.5 | 0.1 | 0.1 | 424.4 |  |
| Site 23 | 13.6 | 6.2 | 8.5 | 8.4 | 68.6 | 39.3 | 74.9 | 44.5 | 28.1 | 23.3 | 15.4 | 18.2 | 16.4 | 18.2 | 17.6 | 15.7 | 406.9 |  |
| Site 24 | 24.6 | 10.4 | 6.3 | 6.8 | 85.6 | 32.8 | 73 | 62.1 | 31.9 | 29.9 | 49.7 | 24.8 | 20.9 | 22.5 | 0.1 | 19.6 | 500.9 |  |
| Site 1 | 3.8 | 0.4 | 10 | 2.1 | 3.9 | 5.2 | 0.3 | 0.3 | 79.1 | 3.4 | 1.2 | 99.5 | 0.3 | 3.6 | 1.4 | 3.7 | 216.9 | 9 |
| Site 2 | 86.4 | 32.29 | 22.3 | 25.2 | 12 | 16 | 3.5 | 4.27 | 164.1 | 24.4 | 30.9 | 104.8 | 24 | 16 | 16 | 14.4 | 596.6 |  |
| Site 3 | 30.6 | 4.38 | 54.6 | 9.6 | 109.9 | 114.1 | 8.2 | 14.92 | 419.9 | 27.5 | 11.5 | 15 | 9.7 | 24.5 | 13.9 | 9.3 | 877.5 |  |
| Site 4 | 0.4 | 0.4 | 4.4 | 0.2 | 1.7 | 2.1 | 0.3 | 0.3 | 34.5 | 2.7 | 1.2 | 44.9 | 0.3 | 2.3 | 0.3 | 1.6 | 95.2 |  |
| Site 5 | 19 | 0.4 | 14 | 0.2 | 7.1 | 7.4 | 3.2 | 2.14 | 110 | 7.6 | 12.7 | 35.2 | 6.4 | 5.6 | 3.9 | 3.8 | 238.2 |  |
| Site 6 | 0.3 | 0.4 | 7.8 | 1.5 | 2.7 | 3.6 | 2.5 | 1.41 | 55.5 | 8.1 | 11.4 | 34.8 | 9 | 3.5 | 1.2 | 0.3 | 143 |  |
| Site 7 | 44.2 | 0.4 | 27.1 | 30.2 | 10.8 | 18.4 | 0.3 | 2.29 | 147.8 | 7 | 2.6 | 125.4 | 0.3 | 5.3 | 2.8 | 4.5 | 428.3 |  |
| Site 8 | 31.5 | 6.83 | 21.6 | 23.2 | 7 | 10.9 | 1.4 | 1.62 | 96.4 | 3.5 | 3.8 | 3.2 | 4.4 | 3.8 | 3.4 | 7.5 | 230 |  |
| Site 9 | 38 | 3.47 | 6.6 | 8.9 | 31.6 | 19.5 | 16.9 | 13.83 | 105.3 | 8.8 | 11.4 | 73 | 13.4 | 3.8 | 14.2 | 17.6 | 386.2 |  |
| Site 10 | 172.3 | 1.23 | 39.6 | 4 | 16.2 | 17.1 | 33.7 | 5.42 | 129.5 | 29 | 10.2 | 35 | 6.4 | 8.1 | 7.1 | 6.2 | 521.2 |  |
| Site 11 | 86.1 | 8.53 | 21.6 | 11.2 | 56.6 | 23.4 | 57 | 49.4 | 281.1 | 50 | 65 | 11.6 | 45.6 | 9.6 | 37.6 | 53.2 | 867.7 |  |
| Site 12 | 58.4 | 2.68 | 16.2 | 2.2 | 6.6 | 7.9 | 3.3 | 1.88 | 89.8 | 5.3 | 12.8 | 113.1 | 6.9 | 4.8 | 3.5 | 3.3 | 338.7 |  |
| Site 13 | 0.3 | 0.4 | 1.8 | 1.8 | 3.1 | 4.4 | 1.6 | 0.3 | 44.6 | 5.3 | 4.5 | 15.2 | 0.3 | 3.5 | 0.3 | 0.3 | 85.7 |  |
| Site 14 | 116.7 | 10.59 | 41.2 | 4.4 | 16.3 | 19.1 | 14.2 | 13.54 | 238.7 | 23.8 | 44.7 | 327.3 | 33.8 | 7.4 | 10.4 | 13 | 935.2 |  |
| Site 15 | 0.3 | 0.4 | 2.6 | 2.5 | 13.3 | 6 | 0.3 | 23.48 | 40 | 28.9 | 2.5 | 17 | 0.3 | 3.2 | 5.4 | 0.3 | 144.8 |  |
| Site 16 | 47 | 0.4 | 15.4 | 3 | 7.8 | 13 | 2.2 | 1.91 | 59.4 | 19.4 | 29.4 | 71.1 | 23.3 | 5.1 | 2 | 3.1 | 303.2 |  |
| Site 1 | 1.6 | 1 | 3.6 | 11.6 | 81.5 | 8.1 | 72 | 55.6 | 75.1 | 11.3 | 12 | 5 | 6.7 | 0.1 | 1.1 | 1.2 | 347.4 | 15 |
| Site 2 | 81.1 | 42.1 | 152.1 | 114.3 | 567.9 | 301.8 | 465.9 | 204.8 | 29.6 | 39.8 | 39.9 | 15.8 | 2.1 | 0.9 | 1.9 | 1.5 | 2061.5 |  |
| Site 3 | 124.7 | 127.8 | 38.9 | 12.4 | 217.7 | 181.6 | 414.8 | 222.2 | 31.8 | 49.4 | 14.9 | 20.5 | 4.4 | 0.1 | 0.6 | 0.9 | 1462.6 |  |
| Site 4 | 0.1 | 0.1 | 175.9 | 172.2 | 2.5 | 9.3 | 8 | 2.5 | 0.1 | 4.6 | 0.1 | 25.8 | 16.3 | 8 | 23.6 | 11.3 | 460 |  |
| Site 5 | 56.1 | 0.1 | 20.1 | 68.7 | 91.6 | 115.4 | 20.3 | 10.5 | 94.6 | 0.1 | 69.5 | 29.6 | 92.5 | 0.1 | 79.6 | 21 | 770.5 |  |
| Site 6 | 0.1 | 0.1 | 5.1 | 21.1 | 14.6 | 1.4 | 12 | 7.7 | 0.1 | 0.1 | 0.1 | 1 | 0.1 | 0.1 | 0.1 | 0.1 | 62.9 |  |
| Site 7 | 178 | 71.4 | 73.2 | 283.1 | 60.6 | 181.3 | 285 | 156.2 | 132.6 | 142.1 | 107.9 | 121.4 | 98.1 | 147.7 | 55.8 | 138 | 2322.4 |  |
| Site 1 | 31.69 | 0.06 | 0.87 | 6.86 | 30.59 | 1.72 | 7.6 | 4.14 | 1.38 | 3.82 | 4.96 | 0.86 | nd | 1.59 | 0.65 | 1.49 | 98.29 | 4 |
| Site 2 | 11.91 | 1.94 | 2.33 | 12.33 | 36.02 | 5.6 | 10.05 | 8.28 | 3.77 | 4.49 | 4.21 | 1.35 | nd | 8.09 | 3.89 | 5.54 | 119.78 |  |
| Site 3 | 32.51 | 1.91 | 5.41 | 16.55 | 58.15 | 7.46 | 18.34 | 16.27 | 6.55 | 12.92 | 10.49 | 4.15 | nd | 5.46 | 0.73 | 5.72 | 202.63 |  |
| Site 4 | 22.96 | 0.06 | 1.3 | 4.04 | 23.92 | 1.73 | 7.25 | 3.72 | 1.65 | 4.11 | 4.38 | 1.34 | nd | 2.04 | 0.25 | 2.06 | 80.81 |  |
| Site 5 | 16.07 | 0.05 | 0.35 | 1.85 | 9.52 | 0.18 | 10.46 | 4.28 | 1.87 | 4.7 | 4.65 | 1.32 | nd | 1.63 | 0.04 | 2.11 | 59.09 |  |
| Site 6 | 36.2 | 0.79 | 4.45 | 12.95 | 76.39 | 10.13 | 15.63 | 10.43 | 0.96 | 6.87 | 7.6 | 3.32 | nd | 2.64 | 1 | 2.8 | 192.14 |  |
| Site 7 | 29.51 | 0.09 | 0.37 | 2.25 | 17.7 | 1.87 | 11.94 | 3.93 | 2.04 | 3.22 | 2.19 | 1.81 | nd | 1.44 | 0.64 | 1.47 | 80.47 |  |
| Site 8 | 11.09 | 0.09 | 1.02 | 1.52 | 9.52 | 0.27 | 10.95 | 8.2 | 3.95 | 8.08 | 7.58 | 3.04 | nd | 4.08 | 0.18 | 4.48 | 74.04 |  |
| Site 9 | 22.6 | 2.07 | 1.39 | 0.74 | 2.5 | 1.79 | 4.08 | 2.84 | 0.03 | 2.09 | 3.87 | 0.28 | nd | 0.6 | 1.06 | 1.46 | 47.4 |  |
| Site 10 | 39.55 | 1.87 | 4.23 | 1.78 | 16.04 | 0.12 | 2.19 | 13.25 | 2.87 | 9.16 | 7.14 | 4.66 | nd | 3.76 | 0.99 | 4.56 | 112.18 |  |
| Site 11 | 13.78 | 0.11 | 0.15 | 0.98 | 6.94 | 0.09 | 6.65 | 4.86 | 2.08 | 4.22 | 4.38 | 1.63 | nd | 2.25 | 0.52 | 2.09 | 50.74 |  |
| Site 12 | 48.06 | 4.6 | 6.29 | 0.45 | 45.92 | 6.95 | 5.91 | 6.47 | 0.69 | 4.91 | 3.92 | 0.5 | nd | 1.67 | 1.52 | 1.77 | 139.63 |  |
| Site 13 | 11.09 | 0.09 | 1.02 | 1.52 | 9.52 | 0.27 | 10.95 | 8.2 | 3.95 | 8.08 | 7.58 | 3.04 | nd | 4.08 | 0.18 | 4.48 | 74.04 |  |
| Site 14 | 5.38 | 1.58 | 0.49 | 1.55 | 5.44 | 4.39 | 2.17 | 4.57 | 0.03 | 3.29 | 5.31 | 1.11 | nd | 2.05 | 2.55 | 1.81 | 41.72 |  |
| Site 15 | 38.33 | 0.01 | 3.62 | 3.35 | 6.47 | 0.69 | 1.99 | 0.05 | 3.8 | 1.58 | 8.62 | 0.18 | nd | 1.28 | 0.3 | 1.33 | 71.61 |  |
| Site 16 | 174.4 | 4.52 | 20.58 | 28.88 | 58.72 | 10.37 | 5.3 | 8.23 | 0.51 | 3.67 | 4.07 | 3.25 | nd | 1.41 | 1.51 | 2.1 | 327.53 |  |
| Site 17 | 52.99 | 4.67 | 6.02 | 0.55 | 18.97 | 9.15 | 12.02 | 9.02 | 0.88 | 5.9 | 8.47 | 0.16 | nd | 3.52 | 1.9 | 3.5 | 137.72 |  |
| Site 18 | 135.95 | 5.84 | 10.74 | 23.01 | 4.37 | 5.28 | 12.07 | 10.83 | 1.64 | 5.68 | 7.52 | 1.41 | nd | 2.76 | 1.81 | 2.93 | 231.85 |  |
| Site 19 | 55.9 | 1.58 | 2.84 | 10.01 | 25.45 | 1.33 | 2.61 | 0.01 | 0.03 | 0.13 | 0.21 | 0.28 | nd | 0.18 | 0.31 | 0.15 | 100.83 |  |
| Site 20 | 11.61 | 1.52 | 0.73 | 0.71 | 2.25 | 0.29 | 2.12 | 0.2 | 0.06 | 0.4 | 0.22 | 2.05 | nd | 0.05 | 0.8 | 0.03 | 23.03 |  |
| Site 21 | 3.67 | 2.48 | 1.15 | 7.26 | 15.86 | 2.48 | 2.06 | 0.54 | 0.02 | 1.42 | 1.65 | 0.4 | nd | 0.07 | 1.13 | 0.37 | 40.56 |  |
| Site 22 | 4.67 | 2.32 | 3.01 | 14.77 | 45.09 | 8.23 | 8.77 | 6.23 | 0.43 | 5.2 | 6.13 | 1.46 | nd | 2.56 | 1.24 | 2.72 | 112.82 |  |
| Site 23 | 133.06 | 5.9 | 9.26 | 11.32 | 56.62 | 13.29 | 14.84 | 11.01 | 2.13 | 9.56 | 6.19 | 7.54 | nd | 4.08 | 3.9 | 3.96 | 292.65 |  |
| Site 24 | 58.83 | 5.22 | 6.52 | 0.55 | 4.89 | 5.58 | 3.96 | 7.51 | 1.1 | 7.55 | 6.16 | 0.84 | nd | 3.12 | 1.88 | 3.45 | 117.14 |  |
| Site 25 | 74.16 | 7.66 | 8.07 | 0.33 | 1.62 | 11.69 | 10.8 | 13.16 | 1.11 | 6.26 | 6.95 | 1.66 | nd | 3.02 | 3.08 | 2.8 | 152.38 |  |
| Site 26 | 84.79 | 4.78 | 9.95 | 25.82 | 42.76 | 6.3 | 10.5 | 4.95 | 0.69 | 1.94 | 3.65 | 0.06 | nd | 0.95 | 1.36 | 0.53 | 199.03 |  |
| Site 27 | 4.71 | 1.36 | 6.62 | 29.15 | 48.3 | 3.28 | 14.27 | 10.11 | 7.75 | 8.42 | 7.76 | 1.76 | nd | 4.09 | 2.18 | 3.08 | 152.84 |  |
| Site 28 | 27.02 | 4.7 | 6.23 | 4.22 | 4.31 | 5.2 | 0.06 | 5.44 | 1.07 | 2.84 | 2.14 | 0.01 | nd | 0.71 | 2.15 | 1.16 | 67.26 |  |
| Site 1 | 17.6 | 40.4 | 0.4 | 0.4 | 3 | 12 | 14 | 2 | 11.6 | 9.2 | 5.8 | 6.2 | 11.4 | 4 | 6.4 | 9 | 153.4 | 58 |
| Site 2 | 6.2 | 28.2 | 0.4 | 0.6 | 3.6 | 12 | 11.4 | 1.6 | 7.8 | 7.4 | 0.8 | 2.4 | 5.4 | 1.4 | 1.6 | 1 | 91.8 |  |
| Site 3 | 14.4 | 34 | 0.6 | 0.6 | 5.4 | 12 | 23.8 | 3.6 | 11.4 | 8.4 | 3.6 | 5.2 | 10.6 | 4.6 | 5 | 7.4 | 150.6 |  |
| Site 4 | 15 | 38.4 | 1.4 | 1.2 | 5.8 | 12 | 26.2 | 10.6 | 24.2 | 24.4 | 7.4 | 8.8 | 15 | 4.2 | 5.8 | 6.2 | 206.6 |  |
| Site 5 | 4.6 | 21.6 | 0.6 | 0.6 | 5.6 | 12 | 16.2 | 4 | 16.4 | 14 | 3.8 | 5.4 | 9.6 | 2.4 | 3 | 4.2 | 124 |  |
| Site 6 | 17.6 | 38.4 | 0.8 | 1 | 5.4 | 12 | 21.8 | 3 | 11 | 9.2 | 1.2 | 3.8 | 6.4 | 1.4 | 2.8 | 3.2 | 139 |  |
| Site 7 | 19.2 | 31 | 1 | 1.6 | 5.2 | 12 | 24.4 | 4.6 | 24 | 21.4 | 7.2 | 4.4 | 7.6 | 2 | 3.8 | 2.2 | 171.6 |  |
| Site 8 | 18.8 | 46.8 | 1 | 1 | 2.6 | 12 | nd | 2.8 | 23.2 | 19.2 | 8.4 | 9.4 | 15.8 | 7.4 | 7.8 | 6 | 182.2 |  |
| Site 9 | 2.4 | 3.2 | nd | 0.4 | 1.4 | 12 | 6.4 | 0.8 | 2 | 1.8 | 1 | 0.8 | 1.8 | 0.6 | 1.6 | 1 | 37.2 |  |
| Site 10 | 4.2 | 21.4 | 0.2 | 0.4 | 3.8 | 12 | 16 | 1.6 | 9 | 5.8 | 2 | 1.2 | 4 | 0.8 | 2 | 1.2 | 85.6 |  |

nd: not detectable

/: no investigation

# Table S5 Emission factors of sixteen PAHs for fifteen emission divisions from nine sources (g t^-1^)

| **Sources** | **Divisions** | **Nap** | **Acy** | **Ace** | **Flo** | **Phe** | **Ant** | **Flu** | **Pyr** | **BaA** | **Chr** | **BbF** | **BkF** | **BaP** | **IcdP** | **DahA** | **BghiP** |
| --- | --- | --- | --- | --- | --- | --- | --- | --- | --- | --- | --- | --- | --- | --- | --- | --- | --- |
| Coking production | mechanical-uncontrol | 2.0E+01 | 5.5E+00 | 5.4E-01 | 2.7E+00 | 7.6E+00 | 2.0E+00 | 3.3E+00 | 4.4E+00 | 9.5E-01 | 9.0E-01 | 5.8E-01 | 4.9E-01 | 7.5E-01 | 3.0E-01 | 4.3E-02 | 3.4E-01 |
|  | mechanical-control | 1.6E+00 | 5.6E-01 | 2.7E-02 | 4.0E-01 | 4.9E-01 | 2.0E-01 | 2.3E-01 | 3.5E-01 | 3.8E-02 | 4.4E-02 | 2.7E-02 | 2.4E-02 | 3.8E-02 | 1.4E-02 | 2.3E-03 | 1.6E-02 |
| Iron-steel industry | uncontrol | 2.4E+00 | 9.5E-01 | 6.8E-02 | 1.4E-01 | 1.7E-01 | 2.1E-02 | 2.6E-02 | 2.3E-02 | 1.3E-02 | 1.5E-02 | 2.2E-02 | 1.3E-02 | 1.6E-02 | 4.2E-02 | 2.0E-02 | 4.2E-02 |
|  | control | 3.3E-01 | 1.8E-02 | 1.1E-02 | 2.3E-02 | 1.6E-02 | 1.9E-03 | 2.3E-03 | 1.5E-03 | 6.6E-04 | 4.4E-04 | 4.9E-04 | 3.8E-04 | 1.8E-04 | 5.7E-04 | 5.9E-04 | 7.1E-04 |
| primary Al production | uncontrol | 5.5E-02 | 2.9E-03 | 3.9E-02 | 6.4E-02 | 4.6E+00 | 5.1E-01 | 1.2E+01 | 9.5E+00 | 2.5E+00 | 6.4E+00 | 2.9E+00 | 8.9E-01 | 7.6E-01 | 6.3E-01 | 2.6E-01 | 6.3E-01 |
|  | control | 7.0E-03 | 5.5E-03 | 3.0E-03 | 2.7E-01 | 7.5E+00 | 1.5E-02 | 1.9E+00 | 3.4E-01 | 2.9E-02 | 2.8E-01 | 2.7E-01 | 5.6E-02 | 2.9E-02 | 1.1E-01 | 2.7E-02 | 2.6E-01 |
| Industry coal combustion | uncontrol | 5.2E+00 | 4.0E-02 | 9.7E-02 | 2.0E-02 | 3.2E+00 | 2.4E-01 | 8.1E-01 | 1.2E-01 | 7.7E-03 | 3.1E-02 | 2.0E-02 | 6.3E-03 | 4.0E-02 | 1.0E-02 | 3.7E-03 | 1.1E-02 |
|  | control | 7.2E-01 | 7.6E-03 | 1.6E-02 | 3.3E-03 | 3.0E-01 | 2.2E-03 | 7.1E-02 | 7.6E-03 | 4.0E-04 | 9.0E-04 | 4.3E-04 | 1.8E-04 | 4.6E-04 | 1.4E-04 | 1.1E-04 | 1.9E-04 |
| Straw burning | traditional stove | 3.7E+01 | 5.2E+00 | 5.3E+00 | 6.1E+00 | 7.7E+00 | 9.9E+00 | 1.7E+00 | 7.3E+00 | 7.0E+00 | 1.2E+00 | 1.2E+00 | 1.0E+00 | 9.6E-01 | 9.2E-01 | 1.5E-01 | 6.9E-01 |
|  | improved stove | 5.0E+00 | 6.0E-01 | 1.3E+00 | 4.7E-01 | 2.7E+00 | 9.1E-01 | 6.0E+00 | 1.8E+00 | 1.3E-01 | 3.5E-01 | 3.1E-01 | 8.4E-01 | 5.8E-01 | 2.7E-01 | 7.2E-02 | 1.2E-01 |
| Firewood burning | Traditional woodstove | 1.2E+02 | 9.4E+01 | 2.5E+01 | 1.0E+01 | 3.0E+01 | 5.3E+00 | 7.5E+00 | 8.8E+00 | 6.3E+00 | 2.6E+00 | 1.6E+00 | 7.0E-01 | 1.5E+00 | 8.7E-01 | 5.7E-01 | 1.1E+00 |
|  | improved woodstove | 1.5E+01 | 1.0E+01 | 5.6E+00 | 7.6E-01 | 7.9E+00 | 2.8E+00 | 6.0E+00 | 2.2E+00 | 6.4E-01 | 7.4E-01 | 4.6E-01 | 5.9E-01 | 5.6E-01 | 2.5E-01 | 2.6E-01 | 1.8E-01 |
| Domestic coal combustion |  | 1.3E-01 | 2.9E-02 | 1.1E-02 | 1.0E-02 | 1.0E-01 | 6.1E-03 | 3.0E-02 | 2.2E-02 | 5.4E-03 | 1.3E-02 | 2.5E-03 | 1.4E-02 | 9.8E-04 | 8.9E-04 | 6.3E-04 | 1.8E-03 |
| Petroleum refineries |  | 2.6E+00 | 0.0E+00 | 0.0E+00 | 0.0E+00 | 8.4E-01 | 4.5E-03 | 4.3E-02 | 6.1E-02 | 0.0E+00 | 0.0E+00 | 0.0E+00 | 0.0E+00 | 1.1E-03 | 0.0E+00 | 0.0E+00 | 9.7E-04 |
| Transport petroleum |  | 2.16E+00 | 1.00E-02 | 4.65E-03 | 4.20E-02 | 1.82E-01 | 4.89E-02 | 4.92E-01 | 5.03E-02 | 1.33E-02 | 1.63E-02 | 1.63E-02 | 2.40E-02 | 2.04E-02 | 2.16E+00 | 1.00E-02 | 4.65E-03 |

Data cited from 80, 81.

# Table S6 Concentrations of sixteen individual PAH congeners and ∑PAHs in sediments sampled from globe excluding China

| **Sites** | **NaP** | **Acy** | **Ace** | **Flo** | **Phe** | **Ant** | **Flu** | **Pyr** | **BaA** | **Chr** | **BbF** | **BkF** | **BaP** | **IcdP** | **DahA** | **BghiP** | **ΣPAHs** | **Reference** |
| --- | --- | --- | --- | --- | --- | --- | --- | --- | --- | --- | --- | --- | --- | --- | --- | --- | --- | --- |
| Site1 | 121 | 413 | 816 | 125 | 614 | 121 | 818 | 142 | 182 | 165 | 710 | nd | 812 | nd | nd | 8 | 5047.03 | 59 |
| Site2 | 485 | 1637 | 1612 | 599 | 1992 | 265 | 1308 | 1416 | 373 | 397 | 1203 | nd | 2350 | nd | nd | nd | 13637.04 |  |
| Site3 | 1498 | 1197 | 2084 | 2126 | 6497 | 454 | 2340 | 2749 | 1244 | 1538 | 1500 | nd | 2389 | nd | nd | nd | 25616.04 |  |
| Site4 | 2024 | 1302 | 2154 | 2667 | 6266 | 446 | 2600 | 3032 | 1850 | 2117 | 2172 | nd | 4050 | nd | nd | 2365 | 33045.03 |  |
| Site5 | 2040 | 1106 | 1847 | 2508 | 6252 | 437 | 2438 | 3000 | 1723 | 1997 | 958 | nd | 3463 | nd | nd | 2194 | 29963.03 |  |
| Site1 | 3 | 0.5 | 0.5 | 27 | 52 | 0.5 | 98 | 81 | 40 | 13 | 19 | 23 | 34 | 33 | 8 | 9 | 441.5 | 60 |
| Site2 | 7 | 1 | 0.5 | 41 | 40 | 4 | 28 | 27 | 13 | 61 | 18 | 21 | 14 | 14 | 17 | 29 | 335.5 |  |
| Site3 | nd | nd | 1 | 21 | 28 | 2 | 75 | 59 | 23 | 17 | 26 | 46 | 38 | 31 | 4 | 10 | 382 |  |
| Site4 | 22 | nd | 7 | 7 | 289 | 57 | 482 | 487 | 316 | 446 | 464 | 239 | 269 | 159 | 128 | 156 | 3528.5 |  |
| Site5 | nd | nd | nd | 27 | 657 | 240 | 1402 | 1211 | 244 | 298 | 61 | 70 | 122 | 35 | 34 | 15 | 4417.5 |  |
| Site6 | 2 | nd | nd | 19 | 30 | 2 | 156 | 125 | 22 | 86 | 50 | 56 | 46 | 22 | 24 | 26 | 667 |  |
| Site7 | nd | nd | nd | 9 | 38 | 2 | 74 | 78 | 10 | 94 | 29 | 11 | 25 | 14 | 3 | 3 | 391.5 |  |
| Site8 | 1 | nd | 2 | 25 | 32 | 1 | 26 | 33 | 3 | 34 | 18 | 12 | 18 | 3 | 3 | 3 | 214.5 |  |
| Site1 | 162.3 | 101.1 | 21.7 | 36.1 | 96.7 | nd | 70.5 | 70.8 | 65.9 | 96.1 | 86.4 | 61.9 | 73.7 | 42.4 | 64.8 | 92.1 | 1142.6 | 61 |
| Site2 | 318.3 | nd | 182.6 | 128.3 | 106.8 | nd | 59.3 | 67 | 48.6 | 85.9 | 66.1 | 42.2 | 43.9 | 27.1 | 34.1 | 0.15 | 1210.55 |  |
| Site3 | 156.3 | nd | 14.4 | 11.7 | 25.1 | nd | 18.8 | 24.9 | 19.9 | 34.9 | 71 | 64.3 | 61.4 | 65.7 | 41.2 | 106.8 | 716.6 |  |
| Site4 | 1092.1 | 281.5 | 214.7 | 360.3 | 1680.6 | 482.6 | 626.5 | 632.9 | 337 | 425.6 | 339.8 | 320.8 | 77.9 | 310.7 | 288 | 468 | 7939 |  |
| Site5 | 652.2 | 303.4 | 59.9 | 126.2 | 791 | 77.2 | 329.9 | 364.5 | 105.4 | 205.1 | 202.1 | 129.1 | 137.9 | 94.9 | 149.3 | 302.8 | 4030.9 |  |
| Site6 | 115.2 | 28.2 | 17.7 | 24.8 | 208.8 | nd | 90.4 | 88.8 | 48.7 | 76.5 | 73.8 | 65.2 | 57.4 | 57.5 | 49.8 | 100 | 1102.9 |  |
| Site1 | 19.063 | 16.81 | nd | nd | 11.47 | 6.03 | 24.6 | 35.48 | 6.43 | 18.49 | 12.47 | 16.3 | 12.51 | nd | nd | 6.48 | 187.433 | 62 |
| Site2 | 13.7 | 11.1 | 27.65 | 30.33 | 3.83 | 2.68 | 14.87 | 17.82 | 3.79 | 13.37 | 9.78 | 11.76 | 11.3 | nd | nd | 2.1 | 174.38 |  |
| Site3 | 7.303 | 2.8 | 11.61 | 1.85 | 2.36 | 0.85 | 2.21 | 1.49 | 0.04 | 0.49 | 0.4 | 0.1 | 0.5 | nd | 0.64 | 0.15 | 32.993 |  |
| Site4 | 2.25 | 0.54 | 3.98 | 0.26 | 0.75 | 0.32 | 0.85 | 0.59 | 0.08 | 0.21 | 0.4 | 0.38 | 0.5 | nd | 0.11 | 0.15 | 11.57 |  |
| Site5 | 10.473 | 7.82 | 6.203 | 0.5 | 11.08 | 3.28 | 17.74 | 13.6 | 7.206 | 5.263 | 13.36 | 12.89 | 8.81 | nd | 3.246 | 9.37 | 131.041 |  |
| Site6 | 11.1 | 7.146 | 3.203 | 0.5 | 8.14 | 0.05 | 14.12 | 4.21 | 6.99 | 4.5 | 10.2 | 7.69 | 5.73 | nd | nd | 7.52 | 91.399 |  |
| Site1 | nd | 0.1 | nd | 4.7 | 7.3 | 2.1 | 24.3 | 41.3 | 12.6 | 26.1 | 2.3 | 19.2 | 9.6 | 112.1 | 2.1 | 44.3 | 308.3 | 63 |
| Site2 | nd | 0.3 | nd | 8.3 | 9.6 | 2.1 | 37.2 | 64.1 | 9.8 | 30.6 | 0.15 | 10.7 | 7.9 | 81.7 | 5.8 | 69.9 | 338.2 |  |
| Site3 | 0.2 | 0.1 | nd | 19.3 | 9.9 | 2.7 | 47.2 | 81.2 | 19.5 | 44.7 | 23.1 | 46.2 | 23.9 | 159.8 | 13.6 | 83.2 | 574.75 |  |
| Site4 | 0.05 | 0.1 | nd | 3.9 | 4.7 | 1.9 | 14.7 | 24.1 | 5.6 | 14.6 | 11.5 | 12.3 | 13.2 | 56.9 | 4.7 | 52.3 | 220.7 |  |
| Site5 | 0.7 | 0.1 | 0.5 | 13.4 | 11.1 | 3.1 | 34.2 | 59.9 | 16.9 | 31.1 | 17.4 | 28.2 | 34.5 | 144.8 | 4 | 75.7 | 475.6 |  |
| Site6 | 20.8 | 12.1 | 23.6 | 32.6 | 39.2 | 8.7 | 11.3 | 19.3 | 2.4 | 11.1 | 2.6 | 13.1 | 5.1 | 97.2 | 8.5 | 21.9 | 329.5 |  |
| Site7 | 13.9 | 8.2 | 21.6 | 29.4 | 68.9 | 18.2 | 7.1 | 11.9 | 1.5 | 2.9 | 4 | 1.7 | 6.4 | 19.3 | 0.7 | 7.1 | 222.8 |  |
| Site8 | 59.7 | 25.3 | 30.7 | 12.6 | 86.8 | 22.6 | 2.9 | 5.4 | 1 | 5.1 | 1 | 4.1 | 2.3 | 6 | 0.2 | 3.2 | 268.9 |  |
| Site9 | 62.9 | 20.9 | 18.7 | 6.3 | 79.5 | 19.8 | 3.1 | 5.1 | 2.1 | 4.5 | 0.3 | 2.7 | 1.1 | 60 | nd | 1.1 | 288.15 |  |
| Site10 | 0.8 | 12.1 | 2.3 | 5.8 | 23.8 | 5.6 | 19.8 | 38.9 | 14.6 | 27.9 | 5.8 | 27.6 | 4.6 | 56.4 | 0.6 | 23.5 | 270.1 |  |
| Site11 | 1.2 | 4.5 | 0.15 | 7.4 | 31.3 | 9.2 | 35.7 | 48.6 | 1.2 | 33.8 | 6.2 | 21.9 | 6.1 | 62.8 | 4.2 | 14.8 | 289.05 |  |
| Site12 | 3.4 | 16.8 | 4.1 | 13.7 | 48.9 | 17.9 | 48.9 | 93.7 | 16.9 | 41.4 | 27.9 | 59.2 | 13.4 | 89.9 | 6.7 | 46.9 | 549.7 |  |
| Site13 | 0.4 | 6.8 | 0.15 | 8.9 | 11.4 | 2.7 | 20.2 | 29.7 | 8.6 | 17.9 | 13.7 | 32.9 | 6.9 | 26.7 | 2.5 | 32.1 | 221.55 |  |
| Site14 | 3.9 | 29.1 | 5.2 | 10.9 | 43.3 | 14.6 | 42.9 | 87.9 | 21.4 | 36.2 | 13.6 | 24.9 | 5.9 | 76.5 | 1.7 | 49.2 | 467.2 |  |
| Site15 | 48.9 | 38.9 | 21.5 | 27.8 | 114.2 | 56.9 | 9.4 | 15.8 | 5.8 | 15.9 | 3.9 | 8.5 | 2.1 | 39.4 | 3.2 | 11.2 | 423.4 |  |
| Site16 | 63.9 | 53.9 | 19.6 | 39.6 | 205.9 | 96.3 | 6.9 | 13.8 | 2.4 | 3.8 | 3.4 | 2.3 | 1.7 | 7.7 | 0.2 | 4.1 | 525.5 |  |
| Site17 | 87.4 | 58.5 | 49.7 | 45.7 | 211.6 | 98.1 | 3.1 | 11.3 | 0.5 | 4.3 | 0.1 | 1.9 | 0.7 | 2.4 | nd | 0.3 | 575.55 |  |
| Site18 | 81.9 | 62.5 | 82.6 | 38.6 | 256.9 | 103.6 | 2.3 | 8.3 | 2.1 | 2.9 | 0.2 | 0.8 | 0.1 | 1.3 | nd | 0.2 | 644.35 |  |
| Site19 | nd | 0.2 | nd | 1.8 | 1.7 | 0.5 | 70.9 | 122.3 | 26.3 | 52.3 | 4.2 | 23.8 | 8.3 | 206.1 | 8 | 36.7 | 563.3 |  |
| Site20 | nd | 0.3 | nd | 2.7 | 2.8 | 0.2 | 110.3 | 190.4 | 19.4 | 59.8 | 2.1 | 16.3 | 11.3 | 115 | 12.3 | 29.4 | 572.5 |  |
| Site21 | 0.2 | 1.3 | nd | 11.5 | 3.2 | 1.4 | 139.1 | 244.1 | 39.8 | 82.5 | 35 | 51.8 | 46.1 | 187.4 | 29.9 | 49.6 | 923.05 |  |
| Site22 | 0.05 | 0.1 | 0.3 | 2.8 | 1.7 | 0.3 | 43.5 | 73.9 | 13.1 | 30.2 | 21.6 | 36.1 | 14.1 | 106.5 | 3.8 | 73.2 | 421.2 |  |
| Site23 | 0.3 | 2.3 | 0.7 | 9.5 | 3.6 | 1 | 104.2 | 181.7 | 34.1 | 64.9 | 33.9 | 47.4 | 50.1 | 244.2 | 14.3 | 28.6 | 820.8 |  |
| Site24 | 8.7 | 4.2 | 3.1 | 49.2 | 35.2 | 5.4 | 38.2 | 57.9 | 7.5 | 23.5 | 7.2 | 22.1 | 14.9 | 149.7 | 3.7 | 31.7 | 462.2 |  |
| Site25 | 15.6 | 10.7 | 12.3 | 58.2 | 59.8 | 12.9 | 23.6 | 33.8 | 3.8 | 6.3 | 9 | 13.5 | 11.8 | 89.8 | 4.9 | 18.5 | 384.5 |  |
| Site26 | 21.7 | 16.3 | 19.4 | 71.6 | 92.4 | 27.5 | 12.9 | 17.4 | 2.6 | 11.1 | 4.1 | 10.3 | 6.3 | 29.2 | 3.1 | 6.3 | 352.2 |  |
| Site27 | 29.1 | 14.1 | 34.7 | 67.9 | 90.1 | 31.7 | 15.7 | 21.2 | 4.8 | 10.7 | 3.5 | 6.6 | 7.9 | 34.3 | 1 | 5.8 | 379.1 |  |
| Site28 | nd | 0.2 | 0.15 | 7.9 | 10.8 | 4.1 | 27.4 | 45.6 | 11.8 | 46.2 | 2.3 | 11.4 | 4.5 | 178.3 | 3.8 | 28.9 | 383.4 |  |
| Site29 | nd | 0.3 | 0.8 | 2.3 | 10.6 | 2 | 32.8 | 59.6 | 13.1 | 52.4 | 2.1 | 9.6 | 3.6 | 132.8 | 5.3 | 64 | 391.35 |  |
| Site30 | 0.6 | 0.7 | 2.9 | 1.7 | 27.3 | 6.4 | 52.2 | 87.3 | 15.3 | 52.4 | 2.1 | 9.6 | 3.6 | 132.8 | 5.3 | 64 | 464.2 |  |
| Site31 | 0.05 | 0.2 | 0.3 | 6.9 | 5.1 | 1.7 | 24.4 | 33 | 10.3 | 43.7 | 13.6 | 12.8 | 9.2 | 78.9 | 2.5 | 55.3 | 297.95 |  |
| Site32 | 1 | 1.6 | 3.7 | 3.9 | 10.9 | 2.3 | 30.7 | 54.9 | 17.1 | 60.2 | 24.7 | 19.6 | 14.9 | 126.4 | 6.2 | 52 | 430.1 |  |
| Site33 | 27.2 | 18.4 | 12.1 | 26.7 | 59.8 | 22 | 7.5 | 15.3 | 8.9 | 38.4 | 5.9 | 5.9 | 3.8 | 74.3 | 17.8 | 20.9 | 364.9 |  |
| Site34 | 27.9 | 21.2 | 37.3 | 32.4 | 103.7 | 48.2 | 9.2 | 12.7 | 5.7 | 22.6 | 3.9 | 1.1 | 2.4 | 11.7 | 1.8 | 8.1 | 349.9 |  |
| Site35 | 62.5 | 23.4 | 32.9 | 30.8 | 119.4 | 56.9 | 4.1 | 7.4 | 2.3 | 12.4 | 2.3 | 2.1 | 1.8 | 4.8 | 1.3 | 3.9 | 368.3 |  |
| Site36 | 78.7 | 39.4 | 42.3 | 35.6 | 97.2 | 48.7 | 1.8 | 6.5 | 1.8 | 11.6 | 2.9 | 1.3 | 1.3 | 2.4 | nd | 4.7 | 376.25 |  |
| Site37 | nd | 0.6 | 1.3 | 3.9 | 26.9 | 6.7 | 13.4 | 27.8 | 6.2 | 27.9 | 4.2 | 9.1 | 2.9 | 58.9 | 1.9 | 16.6 | 208.35 |  |
| Site38 | nd | 0.9 | 1 | 1.4 | 31.9 | 7.8 | 11.3 | 24.6 | 7.1 | 31.2 | 2.4 | 6.3 | 1.8 | 33.9 | 3.7 | 22.2 | 187.55 |  |
| Site39 | nd | 0.5 | 3.6 | 10.1 | 41.9 | 11.5 | 38.1 | 75.6 | 16.9 | 43.9 | 11.8 | 19.5 | 7.4 | 51.2 | 2.8 | 28.1 | 362.95 |  |
| Site40 | nd | 0.5 | 3 | 4.2 | 19.8 | 3.4 | 12.4 | 27.9 | 4.8 | 22.7 | 16.9 | 6.3 | 3.1 | 24.6 | 5.3 | 14.8 | 169.75 |  |
| Site41 | nd | 0.8 | 6.4 | 9 | 39.4 | 8.5 | 39.5 | 79.3 | 13.5 | 42.8 | 19.2 | 21.9 | 5.6 | 69.3 | 5.7 | 32.9 | 393.85 |  |
| Site42 | 13.2 | 13.8 | 18.6 | 24.1 | 87.9 | 27.9 | 5.8 | 18.9 | 7.9 | 36.9 | 7.7 | 3.4 | 1.8 | 32.6 | 11.9 | 15.7 | 328.1 |  |
| Site43 | 14.7 | 19.5 | 41.9 | 42.2 | 184.2 | 57.8 | 6.3 | 19.8 | 4.4 | 22.3 | 6.9 | 0.3 | 1.6 | 6.2 | 1 | 4.6 | 433.7 |  |
| Site44 | 19.3 | 24.7 | 39.4 | 33.9 | 256.1 | 74.9 | 3.8 | 14.2 | 5 | 18.9 | 4.3 | 1.5 | 0.4 | 1.9 | 0.7 | 2.1 | 501.1 |  |
| Site45 | 17.1 | 28.2 | 27.9 | 41.4 | 283.2 | 91.7 | 1.1 | 5.3 | 2.9 | 6.6 | 1.6 | 0.15 | 0.7 | 2.6 | 0.3 | 0.7 | 511.3 |  |
| Site1 | / | nd | 0.99 | 0.8 | 4.3 | 1 | 11.9 | 11 | 10 | 7.3 | 3.6 | 2.8 | 4.3 | 1.1 | 0.82 | 0.08 | 60.04 | 64 |
| Site2 | / | nd | 0.1 | 1.9 | 9.3 | 0.9 | 5.6 | 5.8 | 4.7 | 2.9 | 0.08 | 0.05 | 0.1 | 0.94 | 0.9 | 0.08 | 33.4 |  |
| Site3 | / | 1 | 1 | 4.6 | 8.2 | 0.91 | 8.5 | 9.5 | 4.4 | 3.1 | 3.6 | 2.1 | 3 | 2 | 0.81 | 1.5 | 54.22 |  |
| Site4 | / | nd | 1.1 | 2.9 | 21 | 0.67 | 9.3 | 10 | 4 | 8.73 | 0.08 | 0.05 | 0.1 | 0.12 | 0.08 | 0.38 | 58.56 |  |
| Site5 | / | nd | 0.21 | 0.3 | 2.8 | 0.3 | 3.69 | 4.8 | 5.2 | 2.8 | 4.7 | 5.8 | 8.3 | 0.83 | 0.83 | 2.6 | 43.21 |  |
| Site6 | / | 0.54 | 0.82 | 1 | 4.8 | 1.6 | 15 | 18 | 17 | 13 | 19 | 16 | 20 | 9.3 | 6.8 | 13 | 155.86 |  |
| Site7 | / | 1.3 | 3.7 | 5.1 | 38 | 10 | 62 | 67 | 43 | 62 | 60 | 47 | 70 | 26 | 29 | 25 | 549.1 |  |
| Site8 | / | 1.2 | 1.5 | 2.3 | 39 | 7.5 | 115 | 123 | 67 | 34 | 37 | 36 | 41 | 17 | 7.1 | 24 | 552.6 |  |
| Site9 | / | 8.4 | 21 | 12 | 81 | 18 | 165 | 144 | 61 | 82 | 76 | 71 | 77 | 43 | 17 | 61 | 937.4 |  |
| Site10 | / | 0.71 | 0.53 | 0.58 | 3.1 | 0.81 | 5.5 | 5.8 | 4.2 | 6.3 | 15.4 | 4.93 | 14 | 1.4 | 1 | 0.08 | 64.34 |  |
| Site11 | / | nd | 0.16 | 0.35 | 3.5 | 0.43 | 2.5 | 2.9 | 21 | 20 | 2 | 1.8 | 3 | 2 | 1.7 | 4.3 | 65.68 |  |
| Site12 | / | 1 | 0.48 | 1.4 | 4.4 | 0.6 | 7.1 | 10 | 6 | 5 | 5.5 | 4.1 | 4.7 | 2.3 | 0.9 | 3.7 | 57.18 |  |
| Site13 | / | 0.59 | 1.3 | 1.1 | 5.8 | 0.92 | 8.9 | 8.8 | 3.1 | 5 | 5.5 | 3.9 | 4.1 | 2.7 | 1.7 | 4.7 | 58.11 |  |
| Site14 | / | nd | 0.31 | 1 | 8.3 | 0.58 | 13 | 12 | 5 | 13 | 10 | 5.6 | 0.1 | 0.1 | 0.08 | 0.08 | 69.2 |  |
| Site15 | / | nd | 0.1 | 14 | 3.43 | 1.07 | 0.92 | 1.1 | 1.31 | 7.24 | 0.08 | 0.05 | 0.1 | 0.1 | 0.08 | 0.08 | 29.71 |  |
| Site16 | / | 0.36 | 0.52 | 0.72 | 2.8 | 0.18 | 4.5 | 6.3 | 4.3 | 2.1 | 4.9 | 2.2 | 1.1 | 0.66 | 0.34 | 1.1 | 32.08 |  |
| Site17 | / | 3.59 | 10 | 6.8 | 33 | 5.9 | 66 | 58 | 31 | 43 | 44 | 33 | 33 | 1.3 | 13 | 37 | 418.59 |  |
| Site18 | / | 1.1 | 66 | 69 | 1160 | 254 | 1561 | 1360 | 470 | 822 | 319 | 309 | 303 | 188 | 153 | 285 | 7320.1 |  |
| Site19 | / | nd | 2.5 | 8.5 | 29 | 2.6 | 57 | 64 | 24 | 46 | 29 | 17 | 14 | 14 | 16 | 9.8 | 333.45 |  |
| Site20 | / | 1.6 | 0.7 | 2.2 | 23 | 2.4 | 37 | 44 | 26 | 23 | 30 | 19 | 15 | 5.9 | 2.75 | 14 | 246.55 |  |
| Site21 | / | 1.9 | 0.75 | 1.3 | 14 | 2.5 | 57 | 70 | 60 | 34 | 38 | 38 | 36 | 14 | 7.7 | 22 | 397.15 |  |
| Site22 | / | 2.4 | 11 | 10 | 66 | 16 | 137 | 136 | 94 | 68 | 52 | 55 | 59 | 20 | 8.7 | 31 | 766.1 |  |
| Site23 | / | 0.56 | 3.4 | 2.6 | 16 | 4.5 | 21 | 19 | 7.1 | 13 | 8.2 | 8.4 | 8 | 5.2 | 2.01 | 7.7 | 126.67 |  |
| Site24 | / | nd | 0.1 | 18 | 34 | 19 | 61 | 57 | 41 | 42 | 29 | 22 | 0.1 | 180 | 256 | 171 | 930.25 |  |
| Site25 | / | nd | 8.3 | 15 | 214 | 64 | 591 | 482 | 273 | 194 | 117 | 114 | 145 | 44 | 0.08 | 54 | 2315.43 |  |
| Site26 | / | 0.66 | 8.8 | 12 | 111 | 71 | 245 | 181 | 137 | 132 | 64 | 71 | 72 | 22 | 19 | 44 | 1190.46 |  |
| Site27 | / | 0.28 | 1.1 | 1.82 | 11 | 0.5 | 11 | 12 | 6.6 | 13 | 9.7 | 10 | 12 | 6.6 | 8.8 | 21 | 125.4 |  |
| Site28 | / | 0.49 | 0.95 | 0.74 | 6.6 | 0.58 | 20 | 23 | 13 | 11 | 11 | 14 | 6.6 | 3.8 | 0.98 | 5.9 | 118.64 |  |
| Site29 | / | 0.4 | 2.3 | 0.67 | 3.5 | 0.32 | 7 | 6.6 | 2.4 | 5.8 | 4.4 | 4.1 | 4.5 | 2.5 | 1.6 | 4.4 | 50.49 |  |
| Site30 | / | nd | 2.78 | 2.2 | 20 | 5.6 | 47 | 41 | 27 | 40 | 24 | 21 | 19 | 8.8 | 4.2 | 4.2 | 266.83 |  |
| Site31 | / | nd | 0.1 | 0.82 | 13 | 3.2 | 75 | 61 | 64 | 20 | 0.08 | 0.05 | 0.1 | 0.1 | 0.08 | 0.08 | 237.66 |  |
| Site32 | / | 0.87 | 4.5 | 2.8 | 18 | 2 | 43 | 45 | 29 | 27 | 26 | 19 | 18 | 8.5 | 5 | 13 | 261.67 |  |
| Site33 | / | 0.48 | 5 | 3 | 22 | 4.5 | 36 | 30 | 19 | 22 | 13 | 11 | 12 | 8.3 | 4.1 | 13 | 203.38 |  |
| Site34 | / | nd | 0.77 | 0.83 | 5.3 | 0.79 | 7.4 | 6.1 | 7.6 | 7.1 | 0.08 | 0.05 | 0.1 | 0.1 | 0.08 | 0.08 | 36.43 |  |
| Site35 | / | nd | 1.3 | 3 | 6.1 | 0.76 | 16 | 17 | 16 | 11 | 0.51 | 0.4 | 0.1 | 0.1 | 0.08 | 0.08 | 72.48 |  |
| Site36 | / | 0.51 | 0.1 | 2 | 3.4 | 0.32 | 8.6 | 9.4 | 16 | 4.9 | 8.3 | 9.3 | 8.2 | 4.5 | 2.4 | 5 | 82.93 |  |
| Site37 | / | 0.29 | 0.3 | 0.35 | 0.87 | 0.11 | 0.63 | 0.76 | 0.41 | 0.76 | 0.67 | 0.62 | 0.52 | 0.4 | 0.09 | 0.73 | 7.51 |  |
| Site38 | / | 0.45 | 2.4 | 4 | 33 | 4.6 | 54 | 48 | 70 | 30 | 53 | 48 | 65 | 3.2 | 1.1 | 3.9 | 420.65 |  |
| Site39 | / | 2.9 | 5.4 | 3.1 | 19 | 2.5 | 71 | 70 | 68 | 30 | 36 | 46 | 36 | 22 | 11 | 31 | 453.9 |  |
| Site40 | / | 25 | 6.7 | 5.9 | 67 | 21 | 143 | 140 | 48 | 40 | 21 | 21 | 27 | 26 | 12 | 40 | 643.6 |  |
| Site1 | 75 | 55 | 65 | 371 | 213 | 33 | 70 | 86 | 33 | 66 | 99 | 0.25 | 61 | 51 | / | 0.25 | 1278 | 65 |
| Site2 | 38 | 36 | 37 | 284 | 98 | 24 | 42 | 45 | 24 | 38 | 87 | 0.25 | 43 | 20 | / | 32 | 848 |  |
| Site3 | 37 | 34 | 32 | 228 | 155 | 25 | 81 | 94 | 51 | 46 | 85 | 12 | 54 | 14 | / | 23 | 971 |  |
| Site4 | 90 | 77 | 73 | 533 | 259 | 43 | 94 | 121 | 44 | 29 | 96 | 23 | 63 | 32 | / | 58 | 1635 |  |
| Site5 | 93 | 84 | 91 | 482 | 345 | 66 | 171 | 145 | 64 | 72 | 108 | 26 | 44 | 37 | / | 71 | 1899 |  |
| Site6 | 0.5 | 56 | 67 | 222 | 111 | 29 | 64 | 73 | 31 | 23 | 77 | 0.25 | 11 | 0.25 | / | 0.25 | 764 |  |
| Site7 | 68 | 54 | 85 | 358 | 72 | 155 | 183 | 140 | 63 | 75 | 70 | 28 | 46 | 24 | / | 0.25 | 1421 |  |
| Site8 | 40 | 35 | 41 | 205 | 207 | 42 | 64 | 69 | 16 | 13 | 55 | 0.25 | 21 | 0.25 | / | 0.25 | 808 |  |
| Site9 | 48 | 45 | 56 | 154 | 92 | 61 | 83 | 73 | 48 | 55 | 83 | 14 | 42 | 17 | / | 24 | 895 |  |
| Site10 | 0.5 | 46 | 41 | 281 | 137 | 23 | 60 | 75 | 19 | 38 | 59 | 0.25 | 32 | 11 | / | 19 | 841 |  |
| Site11 | 0.5 | 73 | 30 | 301 | 196 | 31 | 72 | 81 | 27 | 34 | 64 | 18 | 38 | 41 | / | 45 | 1051 |  |
| Site12 | 88 | 62 | 79 | 304 | 136 | 66 | 109 | 117 | 38 | 64 | 88 | 26 | 46 | 0.25 | / | 55 | 1278 |  |
| Site1 | 11.86 | 23.09 | nd | nd | 8.17 | nd | 18.32 | 3.53 | 7.56 | nd | 12.67 | 0.25 | 12.32 | nd | nd | 10.41 | 107.93 | 66 |
| Site2 | 14.74 | nd | nd | nd | nd | nd | 17.46 | 4.16 | 7.52 | 4.74 | 12.17 | 12.63 | 13.77 | nd | nd | 10.4 | 97.59 |  |
| Site3 | 13.95 | nd | 16.46 | nd | 8.42 | nd | 25.53 | 3.19 | 7.42 | 14.77 | 14.1 | 11.97 | 13.35 | nd | nd | 9.91 | 139.07 |  |
| Site4 | 16.56 | nd | 16.54 | nd | 8.24 | 5.43 | 157.7 | 3.75 | 7.78 | 14.73 | 12.79 | 11.57 | 12.77 | nd | nd | 9.91 | 277.77 |  |
| Site5 | 11.9 | nd | 16.49 | nd | 8.18 | nd | 16.45 | 4.41 | 7.5 | 14.73 | 11.51 | 11.64 | 12.44 | nd | nd | 10.05 | 125.3 |  |
| Site6 | 12.81 | nd | 16.45 | nd | 8.21 | nd | 29.51 | 3.6 | 7.45 | 14.73 | 11.33 | 11.77 | nd | nd | nd | 10.21 | 126.07 |  |
| Site7 | 12.66 | nd | nd | nd | 8.45 | nd | 0.8 | 9.87 | 7.38 | nd | 11.85 | 11.66 | 12.22 | nd | nd | 10.03 | 84.12 |  |
| Site8 | 21.83 | nd | nd | nd | 11.67 | 6.57 | 58.61 | 8.15 | nd | 19.78 | 16.71 | 18.86 | 12.32 | nd | nd | 12.29 | 191.71 |  |
| Site9 | 13.21 | nd | 16.59 | nd | 8.38 | 6.61 | 13.34 | 12.93 | 8.02 | 15.16 | 11.48 | 11.75 | 12.26 | nd | nd | 10.07 | 139.8 |  |
| Site10 | 11.95 | nd | nd | nd | nd | nd | 10.52 | 4.54 | 8.85 | 14.74 | nd | 11.53 | nd | nd | nd | 10.04 | 72.17 |  |
| Site11 | 12.22 | nd | nd | nd | 8.22 | nd | 11.45 | 4.98 | 7.73 | 14.81 | nd | 0.25 | 12.27 | nd | nd | 10.09 | 81.77 |  |
| Site1 | 67750 | nd | 373600 | 17 | 24070 | 4020 | 2740 | 25 | nd | 10450 | nd | nd | nd | / | / | / | 482797 | 67 |
| Site2 | 88650 | nd | 16 | 23 | 98120 | 27 | 12500 | 26760 | nd | 22290 | nd | nd | nd | / | / | / | 248511 |  |
| Site3 | 67270 | nd | 15460 | 12 | 19830 | 22750 | 21 | 21180 | nd | 18 | nd | nd | nd | / | / | / | 146666 |  |
| Site4 | 9170 | nd | 820 | 49 | 29840 | 4690 | 87 | 11 | nd | 21 | nd | nd | nd | / | / | / | 44813 |  |
| Site5 | 11920 | nd | 4100 | 3 | 25040 | 1 | 12500 | 10550 | nd | 22290 | nd | nd | nd | / | / | / | 86529 |  |
| Site6 | 251170 | nd | 100700 | 11350 | 5380 | 48720 | 86810 | 11420 | nd | 251170 | nd | nd | nd | / | / | / | 766845 |  |
| Site7 | 534760 | nd | 74170 | 26420 | 131857 | 154530 | 13 | 59400 | nd | 11 | nd | nd | nd | / | / | / | 981286 |  |
| Site8 | 2730 | nd | 41 | 2097 | 61 | 28 | 15 | 30 | nd | 12 | nd | nd | nd | / | / | / | 5139 |  |
| Site1 | nd | 17 | nd | 9 | 19 | 31 | 59 | 64 | 31 | 150 | 125 | / | 329 | 101 | / | 73 | 1008.75 | 68 |
| Site2 | nd | 20 | nd | 11 | 24 | 35 | 71 | 82 | 41 | 170 | 100 | / | 178 | 61 | / | 53 | 846.75 |  |
| Site3 | nd | 18 | nd | 8 | 29 | 30 | 76 | 86 | 68 | 192 | 126 | / | 254 | 95 | / | 70 | 1052.75 |  |
| Site4 | nd | 16 | nd | 0.3 | 15 | 30 | 53 | 59 | 46 | 121 | 84 | / | 146 | 75 | / | 37 | 683.05 |  |
| Site5 | nd | 31 | nd | 11 | 28 | 35 | 77 | 93 | 108 | 246 | 178 | / | 342 | 132 | / | 105 | 1386.75 |  |
| Site6 | nd | 15 | nd | 0.3 | 11 | 20 | 46 | 51 | 41 | 91 | 69 | / | 126 | 52 | / | 84 | 607.05 |  |
| Site7 | nd | 18 | nd | 10.3 | 17 | 37 | 55 | 58 | 78 | 102 | 91 | / | 147 | 64 | / | 42 | 720.05 |  |
| Site8 | nd | 16 | nd | 0.3 | 16 | 35 | 49 | 53 | 51 | 107 | 85 | / | 126 | 29 | / | 42 | 610.05 |  |
| Site9 | nd | 9 | nd | 0.3 | 17 | 19 | 32 | 34 | 40 | 103 | 85 | / | 126 | 29 | / | 42 | 537.05 |  |
| Site10 | nd | 23 | nd | 8 | 23 | 40 | 76 | 89 | 151 | 399 | 1.54 | / | 298 | 121 | / | 66 | 1296.29 |  |
| Site11 | nd | 11 | nd | 6 | 12 | 22 | 34 | 35 | 48 | 128 | 73 | / | 126 | 42 | / | 25 | 562.75 |  |
| Site12 | nd | 9 | nd | 5 | 4 | 24 | 23 | 23 | 35 | 92 | 51 | / | 78 | 28 | / | 17 | 389.75 |  |
| Site13 | nd | 13 | nd | 6 | 12 | 27 | 43 | 40 | 54 | 119 | 68 | / | 106 | 39 | / | 24 | 551.75 |  |
| Site14 | nd | 11 | nd | nd | 13 | 25 | 57 | 48 | 55 | 138 | 66 | / | 102 | 0.3 | / | 24 | 540.3 |  |
| Site1 | 18.4 | 19.7 | 21.5 | 23.1 | 27.9 | 16.2 | 21.2 | 24.3 | 44.3 | 29.5 | 49 | 34.5 | 68 | / | 98.9 | / | 496.5 | 69 |
| Site2 | 16 | 20 | 22.7 | 28.1 | 34.5 | 21.2 | 22.1 | 27.8 | 48.6 | 26.8 | 58.3 | 37.3 | 70.6 | / | 100.1 | / | 534.1 |  |
| Site3 | 14 | 19.5 | 20.6 | 20.6 | 23.4 | 12.3 | 21.9 | 24.3 | 47.5 | 41.2 | 49 | 39.6 | 68 | / | 86.6 | / | 488.5 |  |
| Site4 | 16.2 | 19.7 | 19.7 | 22.4 | 20.6 | 10.5 | 16.5 | 17.2 | 32.9 | 17.3 | 38.7 | 22.9 | 61 | / | 72.4 | / | 388 |  |
| Site5 | 15.2 | 18.7 | 18.6 | 21.4 | 19.6 | 9.5 | 15.5 | 16.2 | 31.9 | 16.3 | 37.7 | 21.9 | 60 | / | 71.4 | / | 373.9 |  |
| Site6 | 17.2 | 20.7 | 20.6 | 23.4 | 21.6 | 11.5 | 17.5 | 18.2 | 33.9 | 18.3 | 39.7 | 23.9 | 62 | / | 73.4 | / | 401.9 |  |
| Site7 | 15.3 | 20.5 | 23.4 | 26.2 | 19.7 | 12.4 | 17 | 18.8 | 31.9 | 16.1 | 37.8 | 21.3 | 59.1 | / | 68.1 | / | 387.6 |  |
| Site8 | 17.5 | 20.9 | 21.9 | 24.1 | 23.9 | 17.8 | 23.9 | 30.9 | 69.3 | 54 | 74.5 | 53 | 82.2 | / | 97.4 | / | 611.3 |  |
| Site9 | 16 | 20.7 | 20 | 22.6 | 20.4 | 11.6 | 16.9 | 17 | 30.5 | 16.3 | 36.8 | 21 | 59.3 | / | 66.6 | / | 375.7 |  |
| Site10 | 25.9 | 19.8 | 20.1 | 23.5 | 21.9 | 10.4 | 17 | 18.2 | 30.5 | 16.5 | 57.7 | 21.3 | 58 | / | 66.7 | / | 407.5 |  |
| Site11 | 22.8 | 19.8 | 19.7 | 25.4 | 24 | 20.6 | 17 | 21.7 | 34.9 | 17.7 | 43.6 | 20.9 | 59 | / | 67.1 | / | 414.2 |  |
| Site12 | 18.8 | 19.5 | 20 | 20.8 | 19.8 | 10.3 | 16.4 | 16.4 | 30.4 | 15.3 | 37.1 | 20.8 | 58.5 | / | 66.8 | / | 370.9 |  |
| Site1 | 7.5 | nd | 2.88 | 7.98 | 41.1 | 0.3 | 54.6 | 104 | 6.42 | 8.58 | 8.22 | 4.08 | 9.24 | 15.2 | 16.4 | 0.2 | 286.95 | 70 |
| Site2 | 7.02 | nd | 5.82 | 0.3 | 31.6 | 12.6 | 33.2 | 66.6 | 116 | 22.4 | 3.12 | 2.4 | 25.2 | 17.5 | 8.64 | 23.1 | 375.75 |  |
| Site3 | 14.3 | nd | 8.82 | 13.2 | 97.2 | 38.8 | 169 | 309 | 415 | 117 | 9.66 | 9.6 | 28.4 | 0.2 | 0.2 | 17.8 | 1248.43 |  |
| Site4 | 2.04 | nd | 5.94 | 14.2 | 191 | 85.8 | 126 | 235 | 15.6 | 19.4 | 9.9 | 5.58 | 20 | 26 | 17.8 | 15 | 789.51 |  |
| Site5 | 2.58 | nd | 6.42 | 0.3 | 13.3 | 4.74 | 73.2 | 97.2 | 121 | 63.6 | 0.15 | 0.2 | 0.2 | 0.2 | 0.2 | 14.6 | 398.14 |  |
| Site6 | 3.9 | nd | 5.46 | 0.3 | 82.8 | 42.7 | 50.7 | 116 | 173 | 112 | 14.3 | 8.52 | 19.3 | 10.9 | 11.6 | 15.9 | 667.63 |  |
| Site7 | 4.86 | nd | 6.12 | 4.2 | 68.4 | 27 | 93.6 | 134 | 13 | 12.2 | 12.8 | 3.3 | 18.4 | 7.14 | 5.52 | 61.2 | 471.99 |  |
| Site8 | 5.4 | nd | 9 | 10.2 | 90 | 24.6 | 36.6 | 120 | 5.4 | 5.4 | 5.4 | 2.4 | 3.6 | 9.6 | 13.2 | 16.2 | 357.25 |  |
| Site9 | 7.86 | nd | 8.58 | 12.6 | 205 | 67.2 | 109 | 212 | 9 | 10.8 | 9.9 | 2.64 | 5.46 | 12.4 | 3.12 | 14.3 | 690.11 |  |
| Site10 | 6.96 | nd | 3.78 | 12.4 | 192 | 104 | 163 | 208 | 330 | 129 | 7.56 | 2.4 | 11.7 | 5.4 | 7.56 | 19 | 1203.01 |  |
| Site11 | 16.4 | nd | 5.46 | 7.62 | 124 | 65.4 | 83.4 | 229 | 221 | 105 | 9.06 | 2.64 | 15.8 | 4.86 | 4.38 | 22.3 | 916.57 |  |
| Site12 | 1.5 | nd | 1.98 | 0.3 | 71.4 | 24.2 | 51.3 | 263 | 145 | 66 | 11.8 | 3.84 | 8.76 | 6.9 | 5.64 | 36 | 697.87 |  |
| Site13 | 21.4 | nd | 5.22 | 0.3 | 156 | 100 | 46.5 | 158 | 9.18 | 9.54 | 4.32 | 1.74 | 11.9 | 9.36 | 6.12 | 18.3 | 558.13 |  |
| Site14 | 17.8 | nd | 8.1 | 3.3 | 94.8 | 57.6 | 43.2 | 135 | 61.8 | 26 | 2.16 | 0.84 | 17.3 | 4.68 | 3.06 | 17.8 | 493.69 |  |
| Site15 | 14.4 | nd | 10.8 | 6.12 | 32.1 | 13.7 | 39.6 | 113 | 115 | 42 | 0.15 | 0.2 | 22.6 | 0.2 | 0.2 | 17.3 | 427.62 |  |
| Site16 | 64.8 | nd | 6.36 | 14.52 | 195 | 112.2 | 114 | 238.2 | 23.34 | 24.18 | 50.82 | 0.2 | 31.62 | 23.1 | 14.58 | 34.26 | 947.43 |  |
| Site17 | 31.56 | nd | 0.2 | 11.34 | 150 | 69 | 67.8 | 192.6 | 9.18 | 22.2 | 3.96 | 1.2 | 9.18 | 12.24 | 9.84 | 17.4 | 607.95 |  |
| Site1 | 36.32 | 1.24 | 81.3 | 32.14 | 9.77 | 46.72 | 36.23 | 19.42 | 68.66 | 85.08 | 59.68 | 51.1 | 80.1 | 30.36 | 18.19 | 83.15 | 739.46 | 71 |
| Site2 | 37.48 | 19.56 | 72.58 | 44.13 | 41.17 | 24.32 | 73.82 | 62.33 | 191.13 | 115.76 | 81.61 | 24.75 | 97.44 | 17.85 | 47.36 | 25.28 | 976.57 |  |
| Site3 | 14.48 | 4.59 | 6.55 | 3.28 | 12.19 | 29.97 | 96.75 | 91.14 | 118.16 | 3 | 62.49 | 28.84 | 42.83 | 16.57 | 13.07 | 10.26 | 554.17 |  |
| Site4 | 24.8 | 1.35 | 8.02 | 19.16 | 16.52 | 26.4 | 42.29 | 34.52 | 26.26 | 4.36 | 41.11 | 48.79 | 34.49 | 72.55 | 20.98 | 59.72 | 481.32 |  |
| Site5 | 26.3 | 7.3 | 38.1 | 21.7 | 20.9 | 30.9 | 48.8 | 52.3 | 91 | 52 | 61.9 | 36.3 | 60.1 | 12.3 | 21.9 | 63.2 | 645 |  |
| Site1 | 119.82 | 0.42 | 1.48 | 3.46 | 7.3 | 0.73 | 2.35 | 2.28 | 0.26 | 0.57 | 2 | 0.1 | 0.95 | 0.1 | 0.83 | 0.34 | 142.99 | 72 |
| Site2 | 74.15 | 0.25 | 0.9 | 2.21 | 4.66 | 0.46 | 2.32 | 1.97 | 0.33 | 0.73 | 10.25 | 6.82 | 1.77 | 0.12 | 1.29 | 0.14 | 108.37 |  |
| Site3 | 30.48 | 0.19 | 0.57 | 2.16 | 4.67 | 0.7 | 5.82 | 3.93 | 0.85 | 1.99 | 10.02 | 1.01 | 5.55 | 0.05 | 2.74 | 0.25 | 70.98 |  |
| Site4 | 34.02 | 0.23 | 0.69 | 1.92 | 5.29 | 0.71 | 3.38 | 3.06 | 0.57 | 1.14 | 6.65 | 40.04 | 9.38 | 0.07 | 1.68 | 0.76 | 109.59 |  |
| Site5 | 23.6 | 0.21 | 0.55 | 1.4 | 4.68 | 0.67 | 1.62 | 1.57 | 0.35 | 0.77 | 1.93 | 0.7 | 0.07 | 0.11 | 0.46 | 0.19 | 38.88 |  |
| Site6 | 118.7 | 0.28 | 1.05 | 2.22 | 6.08 | 0.74 | 1.94 | 1.51 | 0.16 | 0.45 | 2.22 | 0.61 | 0.48 | 0.14 | 0.2 | 0.21 | 136.99 |  |
| Site1 | 54.5 | 1.7 | 0.8 | 0.9 | 5.8 | 0.7 | 13.4 | 11.7 | 8.5 | 8 | 55.5 | / | 13.3 | 15.2 | 1.1 | 7.6 | 198.7 | 73 |
| Site2 | 0.15 | 0.3 | 0.05 | 0.05 | 3.6 | 0.4 | 5.2 | 4.9 | 2.4 | 3.2 | 7.1 | / | 3.9 | 3.3 | 0.2 | 3.2 | 37.95 |  |
| Site3 | 11.6 | 0.05 | 2 | 0.1 | 2.6 | 0.3 | 4.3 | 4.5 | 1.9 | 2.6 | 6.7 | / | 3.7 | 2.3 | 0.2 | 3.1 | 45.95 |  |
| Site4 | 3.6 | 0.2 | 1.4 | 2.5 | 42.2 | 6.6 | 72.4 | 72.9 | 42.5 | 47.6 | 95.1 | / | 47.7 | 45.8 | 4.6 | 37.2 | 522.3 |  |
| Site5 | 0.15 | 0.01 | 1.2 | 0.3 | 1.3 | 0.2 | 2.2 | 2.1 | 1.9 | 1 | 8.2 | / | 2.7 | 0.2 | 1.9 | 0.7 | 24.06 |  |
| Site6 | 25.5 | 0.2 | 3.9 | 0.4 | 1.6 | 0.2 | 4.3 | 3.6 | 3 | 1.8 | 4.2 | / | 0.9 | 2.2 | 0.5 | 3.6 | 55.9 |  |
| Site7 | 17.4 | 0.05 | 0.5 | 0.6 | 2.1 | 0.3 | 5.6 | 4.5 | 2.8 | 2.5 | 14.6 | / | 3.3 | 2.1 | 0.05 | 1.5 | 57.9 |  |
| Site8 | 6.9 | 0.1 | 1.9 | 0.2 | 2.3 | 0.2 | 4.6 | 5.7 | 1.7 | 2.5 | 10 | / | 1.9 | 2.5 | 0.5 | 2 | 43 |  |
| Site9 | 0.6 | 0.05 | 0.1 | 0.9 | 14.5 | 2.2 | 42.4 | 38.3 | 31.7 | 24.5 | 122.4 | / | 28.3 | 42.7 | 3.1 | 19.3 | 371.05 |  |
| Site10 | 50.5 | 0.05 | 1.1 | 0.5 | 6.5 | 0.9 | 14.9 | 11.2 | 8.4 | 7.1 | 34.1 | / | 7.1 | 7.5 | 0.7 | 4.5 | 155.05 |  |
| Site1 | 1.51 | 0.09 | 0.49 | 0.8 | 2.85 | nd | 0.33 | 0.28 | 0.01 | 0.15 | 0.01 | nd | nd | nd | 0.18 | 0.3 | 7.08 | 74 |
| Site2 | 0.46 | 0.05 | 0.22 | 0.25 | 1.01 | nd | 0.61 | 0.72 | 0.79 | 0.95 | 1.47 | 0.05 | 22.2 | 0.83 | 0.47 | 0.38 | 30.48 |  |
| Site3 | 2.56 | 0.15 | 1.12 | 2.02 | 8.44 | nd | 1.18 | 1.77 | 0.01 | 0.58 | 0.17 | 0.05 | 1.09 | 0.05 | 0.18 | 0.08 | 19.47 |  |
| Site4 | 0.82 | 0.06 | 0.51 | 0.58 | 5.49 | nd | 5.01 | 4.74 | 2.32 | 3.04 | 8.32 | 0.56 | 4.17 | 2.26 | 0.71 | 2.38 | 40.99 |  |
| Site5 | 0.27 | 0.04 | 0.12 | 0.14 | 0.8 | nd | 0.25 | 0.39 | 0.02 | 0.4 | 0.47 | 0.01 | 6.15 | 0.15 | 0.68 | 0.1 | 10.01 |  |
| Site1 | 143 | 486 | 67 | 301 | 598 | 34 | 284 | 364 | 46 | 613 | 681 | 44 | 147 | / | 173 | 94 | 4075 | 75 |
| Site2 | 33 | 45 | 42 | 58 | 93 | 37 | 86 | 259 | 51 | 81 | 77 | 23 | 55 | / | 63 | 0.2 | 1003.2 |  |
| Site3 | 89 | 48 | 12 | 21 | 61 | 20 | 97 | 133 | 42 | 55 | 60 | 0.2 | 41 | / | 38 | 19 | 736.2 |  |
| Site4 | 72 | 93 | 31 | 51 | 106 | 33 | 94 | 143 | 51 | 42 | 53 | 12 | 41 | / | 35 | 11 | 868 |  |
| Site5 | 2 | 33 | 16 | 31 | 75 | 23 | 64 | 122 | 25 | 21 | 31 | 0.2 | 35 | / | 27 | 0.2 | 505.4 |  |
| Site6 | 91 | 113 | 24 | 107 | 247 | 44 | 222 | 351 | 73 | 42 | 94 | 0.2 | 53 | / | 44 | 52 | 1557.2 |  |
| Site7 | 63 | 54 | 58 | 18 | 64 | 29 | 12 | 13 | 10 | 13 | 33 | 0.2 | 14 | / | 9 | 12 | 402.2 |  |
| Site8 | 76 | 68 | 81 | 29 | 101 | 45 | 77 | 56 | 33 | 30 | 44 | 11 | 23 | / | 17 | 0.2 | 691.2 |  |
| Site9 | 72 | 63 | 77 | 15 | 89 | 30 | 63 | 47 | 23 | 28 | 61 | 0.2 | 8 | / | 3 | 0.2 | 579.4 |  |
| Site10 | 32 | 26 | 29 | 21 | 33 | 9 | 34 | 36 | 4 | 6 | 15 | 6 | 8 | / | 7 | 9 | 275 |  |
| Site11 | 2 | 5 | 6 | 11 | 32 | 12 | 4 | 5 | 3 | 1 | 7 | 3 | 5 | / | 0.2 | 0.2 | 96.4 |  |
| Site12 | 75 | 55 | 65 | 371 | 213 | 33 | 70 | 86 | 33 | 66 | 99 | 0.2 | 61 | / | 51 | 0.2 | 1278.4 |  |
| Site13 | 38 | 36 | 37 | 284 | 98 | 24 | 42 | 45 | 24 | 38 | 87 | 0.2 | 43 | / | 20 | 32 | 848.2 |  |
| Site14 | 37 | 34 | 32 | 228 | 155 | 25 | 81 | 94 | 51 | 46 | 85 | 12 | 54 | / | 14 | 23 | 971 |  |
| Site15 | 90 | 77 | 73 | 533 | 259 | 43 | 94 | 121 | 44 | 29 | 96 | 23 | 63 | / | 32 | 58 | 1635 |  |
| Site16 | 93 | 84 | 91 | 482 | 345 | 66 | 171 | 145 | 64 | 72 | 108 | 26 | 44 | / | 37 | 71 | 1899 |  |
| Site17 | 2 | 56 | 67 | 222 | 111 | 29 | 64 | 73 | 31 | 23 | 77 | 0.2 | 11 | / | 0.1 | 0.2 | 766.4 |  |
| Site18 | 68 | 54 | 85 | 358 | 72 | 155 | 183 | 140 | 63 | 75 | 70 | 28 | 46 | / | 24 | 0.2 | 1421.2 |  |
| Site19 | 40 | 35 | 41 | 205 | 207 | 42 | 64 | 69 | 16 | 13 | 55 | 0.2 | 21 | / | 0.2 | 0.2 | 808.6 |  |
| Site20 | 48 | 45 | 56 | 154 | 92 | 61 | 83 | 73 | 48 | 55 | 83 | 14 | 42 | / | 17 | 24 | 895 |  |
| Site21 | 2 | 46 | 41 | 281 | 137 | 23 | 60 | 75 | 19 | 38 | 59 | 0.2 | 32 | / | 11 | 19 | 843.2 |  |
| Site22 | 2 | 73 | 30 | 301 | 196 | 31 | 72 | 81 | 27 | 34 | 64 | 18 | 38 | / | 41 | 45 | 1053 |  |
| Site23 | 88 | 62 | 79 | 304 | 136 | 66 | 109 | 117 | 38 | 64 | 88 | 26 | 46 | / | 0.2 | 55 | 1278.2 |  |
| Site24 | 75 | 62 | 77 | 203 | 195 | 44 | 165 | 241 | 75 | 46 | 96 | 18 | 34 | / | 23 | 42 | 1396 |  |
| Site25 | 2 | 54 | 73 | 101 | 77 | 18 | 38 | 42 | 47 | 39 | 75 | 12 | 44 | / | 37 | 0.2 | 659.2 |  |
| Site26 | 2 | 48 | 54 | 128 | 101 | 33 | 56 | 65 | 55 | 51 | 80 | 0.2 | 46 | / | 18 | 31 | 768.2 |  |
| Site27 | 86 | 79 | 84 | 193 | 175 | 35 | 72 | 66 | 45 | 53 | 79 | 21 | 38 | / | 0.2 | 30 | 1056.2 |  |
| Site28 | 30 | 37 | 32 | 160 | 123 | 74 | 36 | 42 | 132 | 71 | 36 | 0.2 | 82 | / | 8 | 0.2 | 863.4 |  |
| Site29 | 90 | 74 | 95 | 39 | 242 | 40 | 98 | 128 | 112 | 83 | 102 | 25 | 62 | / | 38 | 55 | 1283 |  |
| Site30 | 71 | 59 | 65 | 135 | 102 | 21 | 60 | 76 | 56 | 28 | 95 | 0.2 | 61 | / | 42 | 0.2 | 871.4 |  |
| Site1 | 4.91 | 47.95 | 0.82 | 23.97 | 53.59 | 18.12 | 13.8 | 16.68 | 8.2 | 13.25 | 19.49 | 3.33 | 1.54 | 0.27 | 10.01 | 0.73 | 236.66 | 76 |
| Site2 | 2.5 | 1.99 | 0.31 | 8.84 | 26 | 3.96 | 8.56 | 11.08 | 3.37 | 5.35 | 9.41 | 2.08 | 0.98 | 0.06 | 2.01 | 0.53 | 87.03 |  |
| Site3 | 19.09 | 39.51 | 2.02 | 13.82 | 30.62 | 7.93 | 9.52 | 13.25 | 6.7 | 11.97 | 7.4 | 0.35 | 0.58 | 0.75 | 0.96 | 2.18 | 166.65 |  |
| Site4 | 9.28 | 5.57 | 1.74 | 9.46 | 26.7 | 5.6 | 44.23 | 48.71 | 6.98 | 11.8 | 2.74 | 1.69 | 0.97 | 0.1 | 3.94 | 0.23 | 179.74 |  |
| Site5 | 5.04 | 0.25 | 1.81 | 56 | 53.5 | 8.44 | 63.43 | 108.9 | 1.78 | 14.63 | 0.86 | 0.64 | 0.18 | 5.85 | 4.27 | 0.65 | 326.23 |  |
| Site6 | 6.61 | 0.25 | 1.21 | 25.2 | 68.3 | 8.23 | 6.31 | 21.53 | 0.51 | 2.37 | 0.75 | 0.82 | 0.15 | 1.83 | 4.65 | 0.22 | 148.94 |  |
| Site7 | 3.48 | 0.05 | 2.48 | 2.58 | 3.98 | 0.69 | 250 | 107.45 | 15.13 | 21.21 | 1.62 | 0.12 | 0.1 | 3.03 | 0.7 | 5.65 | 418.27 |  |
| Site8 | 0.33 | 0.05 | 1.73 | 4.64 | 6.66 | 1.25 | 21.36 | 42.46 | 0.63 | 4.21 | 0.98 | 0.36 | 0.38 | 4.13 | 2.74 | 0.92 | 92.83 |  |
| Site9 | 5.78 | 17.61 | 84.87 | 215.59 | 252.54 | 47.99 | 44.66 | 46.41 | 8.34 | 16.39 | 11.02 | 4.72 | 14.58 | 11.2 | 71.34 | 15.04 | 868.08 |  |
| Site10 | 52.33 | 65.08 | 13.5 | 265.2 | 350.4 | 87 | 47.1 | 196.91 | 7.79 | 12.43 | 35.7 | 4.17 | 12.72 | 4.66 | 59.31 | 18.6 | 1232.9 |  |
| Site11 | 60.69 | 0.2 | 39.37 | 41.59 | 73.06 | 24.26 | 233.88 | 244.62 | 15.72 | 27.11 | 10.43 | 0.87 | 3.01 | 10.3 | 1.66 | 2.57 | 789.34 |  |
| Site12 | 0.04 | 0.26 | 0.84 | 1.19 | 0.82 | 0.37 | 0.9 | 1.02 | 0.03 | 0.1 | 0.25 | 0.23 | 0.05 | 0.2 | 1.95 | 0.07 | 8.32 |  |
| Site13 | 0.28 | 0.1 | 0.1 | 0.3 | 0.62 | 0.17 | 0.14 | 0.19 | 0.005 | 0.01 | 0.3 | 0.01 | 0.12 | 0.06 | 0.25 | 0.09 | 2.745 |  |
| Site14 | 472.22 | 50 | 52.6 | 718 | 532 | 73 | 502.27 | 539.68 | 38.96 | 177.81 | 348 | 15.8 | 101.2 | 68.75 | 14 | 55.81 | 3760.1 |  |
| Site15 | 124.36 | 2.07 | 12.47 | 150.33 | 135.46 | 16.88 | 216.83 | 258.34 | 3.46 | 11.39 | 9.66 | 0.65 | 9.66 | 17.72 | 9.56 | 4.68 | 983.52 |  |
| Site16 | 60.56 | 1.25 | 0.12 | 94.5 | 101 | 22.81 | 92.68 | 125.42 | 2.71 | 6.76 | 5.85 | 16.3 | 10.05 | 19.69 | 3.35 | 88.89 | 651.94 |  |
| Site17 | 3.53 | nd | nd | 0.05 | 3.36 | 1.33 | 21.61 | 26.95 | 1.01 | 3.75 | 1.66 | 0.09 | 0.42 | 0.69 | 1.28 | 102.78 | 168.57 |  |
| Site18 | 3.01 | nd | nd | 0.01 | 1.58 | 0.31 | 41.5 | 18.88 | 2.24 | 3.23 | 1.18 | 0.07 | 0.16 | 1.91 | 0.54 | 2.06 | 76.74 |  |
| Site19 | 1.17 | nd | nd | 0.84 | 0.99 | 0.18 | 19.25 | 7.9 | 1.66 | 2.02 | 0.5 | 0.11 | nd | 4.26 | 0.4 | 11.32 | 50.71 |  |
| Site20 | 3.94 | nd | nd | 1.85 | 3.32 | 1.31 | 8.35 | 2.6 | 2.44 | 1.64 | 0.9 | 0.27 | 0.33 | 2.08 | nd | 2.74 | 31.9 |  |
| Site21 | 0.64 | nd | nd | 0.78 | 2.62 | 0.51 | 5.34 | 2.33 | 1.07 | 0.82 | 0.3 | 0.08 | nd | 0.44 | nd | 0.72 | 15.83 |  |
| Site22 | 0.04 | nd | nd | 2.42 | 3.25 | 0.87 | 2.63 | 1.65 | 0.25 | 0.33 | nd | nd | nd | 0.72 | nd | 4.45 | 16.81 |  |
| Site23 | 0.55 | nd | 0.69 | 5.76 | 2.4 | 0.47 | 0.52 | 0.74 | 0.07 | 0.25 | 0.14 | 0.03 | nd | 3.88 | 0.92 | 1.47 | 17.99 |  |
| Site24 | 0.35 | nd | 0.5 | 2.66 | 2.76 | 1 | 1.9 | 0.88 | 0.12 | 0.37 | 0.13 | 0.08 | 0.14 | 0.22 | 0.3 | 13.61 | 25.07 |  |
| Site25 | 6.4 | 0.5 | 5.02 | 18.74 | 63.3 | 10.95 | 18.25 | 37.83 | 2.64 | 7.65 | 0.58 | 0.14 | 0.15 | 13.39 | 5.97 | 3.12 | 194.63 |  |
| Site26 | 0.83 | nd | 0.18 | 0.72 | 22.36 | 3.69 | 9.95 | 71.87 | 10.19 | 29.55 | 7.7 | 0.74 | 2.81 | 41.53 | 1.44 | 12.87 | 216.45 |  |
| Site27 | 2.28 | nd | 0.04 | 1.6 | 4.95 | 1.31 | 3.16 | 25.87 | 3.33 | 9.94 | 3.1 | 0.26 | 1.19 | 17.39 | 0.55 | 10.76 | 85.78 |  |
| Site28 | 0.04 | nd | 0.03 | 4.38 | 0.97 | 0.19 | 0.03 | 0.22 | 0.04 | 0.13 | nd | nd | nd | 1.08 | 0.07 | 0.2 | 7.5 |  |
| Site1 | / | 0.98 | 0.45 | 1.62 | 3.61 | 0.17 | 4.18 | 4.35 | 1.18 | 3.1 | 2.71 | 1.35 | 0.33 | 0.84 | / | 1.44 | 26.31 | 77 |
| Site2 | / | 2.16 | 0.08 | 1.9 | 3.1 | 0.17 | 2.27 | 2.54 | 0.26 | 0.71 | 0.36 | 0.3 | 0.12 | nd | / | nd | 13.99 |  |
| Site3 | / | 1.71 | 0.06 | 1.08 | 2.37 | 0.21 | 2.48 | 2.22 | 0.32 | 1.23 | 1.01 | 0.28 | 0.26 | nd | / | 0.48 | 13.72 |  |
| Site4 | / | 0.93 | 0.27 | 1.33 | 2.26 | 0.11 | 2.26 | 2.24 | 0.64 | 1.12 | 1.13 | 0.77 | 0.11 | 0.43 | / | 0.46 | 14.06 |  |
| Site5 | / | 1.76 | 0.68 | 2.62 | 3.61 | 0.38 | 3.52 | 3.62 | 1.48 | 2.93 | 3.43 | 1.53 | 0.84 | 1.45 | / | 2.59 | 30.44 |  |
| Site6 | / | 1.99 | 0.21 | 2.21 | 4.14 | 0.18 | 3.31 | 3.93 | 1.04 | 2.75 | 2.69 | 1.06 | 0.43 | 0.62 | / | 1.02 | 25.58 |  |
| Site7 | / | 2.1 | 0.18 | 2.07 | 3.73 | 0.2 | 2.78 | 4.93 | 0.66 | 2.63 | 2.09 | 0.86 | 0.39 | 0.48 | / | 1.16 | 24.26 |  |
| Site8 | / | 1.58 | 0.16 | 1.98 | 2.95 | 0.24 | 2.43 | 2.76 | 0.64 | 1.91 | 1.87 | 0.55 | 0.18 | 0.62 | / | 1.13 | 19 |  |
| Site9 | / | 1.82 | 0.14 | 2.5 | 4.16 | 0.2 | 2.29 | 2.31 | 0.36 | 0.97 | 1.2 | 0.28 | 0.23 | nd | / | 0.68 | 17.15 |  |
| Site10 | / | 1.72 | 0.12 | 1.76 | 3.11 | 0.15 | 2.59 | 2.88 | 0.48 | 1.35 | 1.06 | 0.53 | 0.11 | nd | / | nd | 15.88 |  |
| Site11 | / | 1.26 | 0.1 | 2.1 | 3.04 | 0.21 | 2.16 | 3.16 | 0.42 | 1.23 | 1.08 | 0.41 | 0.14 | nd | / | 0.59 | 15.91 |  |
| Site12 | / | 1.22 | 0.12 | 1.96 | 3.41 | 0.17 | 1.69 | 1.53 | 0.29 | 0.69 | 0.8 | 0.34 | 0.12 | nd | / | 0.42 | 12.77 |  |
| Site13 | / | 1.51 | 0.13 | 2.57 | 4.92 | 0.23 | 2.76 | 2.07 | 0.32 | 0.78 | 0.92 | 0.28 | 0.16 | nd | / | nd | 16.67 |  |
| Site14 | / | 1.71 | 0.13 | 2.09 | 2.83 | 0.17 | 2.05 | 2.42 | 0.24 | 0.56 | 0.5 | 0.15 | nd | nd | / | nd | 12.89 |  |
| Site15 | / | 1.11 | 0.1 | 2.37 | 3.18 | 0.2 | 1.88 | 1.76 | 0.3 | 0.68 | 0.47 | 0.28 | nd | nd | / | nd | 12.37 |  |
| Site16 | / | 1.81 | 0.12 | 1.9 | 2.99 | 0.16 | 1.9 | 2.14 | 0.13 | 0.46 | 0.37 | 0.14 | nd | nd | / | nd | 12.16 |  |
| Site1 | 6 | 0.25 | 106 | 10 | 131 | 45 | 390 | 369 | 123 | 173 | 164 | 85 | 207 | 87 | 108 | 128 | 2131 | 14 |
| Site2 | 0.12 | 0.25 | 6 | 3 | 36 | 11 | 86 | 109 | 41 | 51 | 51 | 25 | 56 | 4 | 23 | 50 | 551 |  |
| Site3 | 3 | 0.25 | 18 | 3 | 41 | 10 | 120 | 91 | 58 | 82 | 72 | 32 | 111 | 9 | 59 | 47 | 757 |  |
| Site4 | 5 | 0.25 | 347 | 0.02 | 334 | 120 | 798 | 1017 | 247 | 124 | 254 | 209 | 313 | 167 | 161 | 228 | 4323 |  |
| Site5 | 15 | 0.25 | 60 | 13 | 116 | 40 | 350 | 272 | 166 | 224 | 200 | 76 | 226 | 110 | 135 | 130 | 2134 |  |
| Site6 | 15 | 0.25 | 0.02 | 3 | 18 | 6 | 56 | 36 | 22 | 21 | 24 | 13 | 22 | 15 | 18 | 21 | 290 |  |
| Site7 | 29 | 0.25 | 134 | 40 | 440 | 180 | 1050 | 953 | 425 | 457 | 575 | 234 | 564 | 327 | 312 | 398 | 6118 |  |
| Site8 | 7 | 0.25 | 41 | 5 | 49 | 19 | 140 | 86 | 47 | 54 | 128 | 25 | 68 | 17 | 27 | 45 | 757 |  |
| Site9 | 16 | 0.25 | 73 | 10 | 114 | 38 | 215 | 144 | 86 | 108 | 82 | 36 | 121 | 10 | 48 | 62 | 1162 |  |
| Site1 | 120 | 2.8 | 77 | 31 | 113 | 22 | 156 | 118 | 69 | 79 | 83 | 40 | 14 | 47 | 23 | 81 | 1077 | 7 |
| Site2 | 125 | 2.8 | 83 | 37 | 56 | 18 | 28 | 31 | 13 | 14 | 13 | 12 | 14 | 3.2 | 23 | 15 | 487 |  |
| Site3 | 70 | 0.8 | 23 | 13 | 99 | 23 | 169 | 129 | 79 | 41 | 45 | 29 | 14 | 27 | 23 | 34 | 818 |  |
| Site4 | 62 | 6.4 | 23 | 47 | 327 | 60 | 900 | 590 | 370 | 215 | 336 | 181 | 31 | 185 | 77 | 276 | 3684 |  |
| Site5 | 106 | 2.8 | 64 | 16 | 80 | 15 | 124 | 94 | 56 | 40 | 60 | 42 | 14 | 19 | 23 | 51 | 806 |  |
| Site6 | 35 | 2.8 | 6.9 | 15 | 126 | 23 | 233 | 177 | 96 | 58 | 71 | 45 | 14 | 43 | 23 | 57 | 1026 |  |
| Site7 | 144 | 7.06 | 101 | 53 | 138 | 28 | 148 | 120 | 55 | 36 | 43 | 27 | 17 | 32 | 23 | 45 | 1018 |  |
| Site8 | 90 | 0.8 | 50 | 12 | 32 | 8 | 12 | 9.2 | 5.2 | 6.2 | 3.5 | 4 | 14 | 3.2 | 23 | 1.1 | 274 |  |
| Site9 | 59 | 0.8 | 23 | 5.4 | 30 | 7 | 25 | 9.2 | 14 | 14 | 17 | 9.5 | 14 | 11 | 23 | 15 | 276 |  |
| Site10 | 113 | 44 | 23 | 39 | 78 | 15 | 100 | 74 | 43 | 37 | 52 | 25 | 14 | 30 | 32 | 49 | 758 |  |
| Site11 | 241 | 101 | 196 | 280 | 184 | 37 | 239 | 212 | 141 | 90 | 53 | 29 | 14 | 95 | 32 | 155 | 2090 |  |
| Site12 | 160 | 49 | 113 | 40 | 79 | 17 | 85 | 95 | 40 | 25 | 49 | 30 | 14 | 34 | 32 | 50 | 903 |  |
| Site13 | 513 | 421 | 470 | 328 | 385 | 92 | 1086 | 787 | 373 | 242 | 60 | 33 | 45 | 185 | 77 | 272 | 5370 |  |
| Site14 | 256 | 92 | 210 | 52 | 208 | 54 | 480 | 359 | 202 | 127 | 54 | 32 | 28 | 96 | 23 | 140 | 2413 |  |
| Site15 | 123 | 117 | 76 | 120 | 58 | 19 | 103 | 80 | 47 | 30 | 54 | 33 | 14 | 21 | 23 | 28 | 947 |  |
| Site16 | 94 | 0.8 | 23 | 14 | 43 | 10 | 37 | 31 | 15 | 14 | 51 | 31 | 14 | 9.2 | 23 | 12 | 421 |  |
| Site17 | 80 | 0.8 | 23 | 17 | 106 | 23 | 105 | 82 | 47 | 32 | 44 | 32 | 14 | 20 | 23 | 28 | 676 |  |
| Site18 | 32 | 0.8 | 6.9 | 18 | 57 | 12 | 67 | 31 | 28 | 25 | 53 | 28 | 14 | 17 | 23 | 22 | 436 |  |
| Site1 | nd | nd | nd | 611.76 | 143.62 | 72.15 | 466.18 | 229.47 | 262.79 | 286.74 | / | 303.14 | 257.87 | nd | 8.27 | 568.79 | 3210.78 | 78 |
| Site2 | nd | nd | nd | 574.45 | 135.42 | 69.45 | 398.51 | 220.89 | 225.78 | 252.54 | / | 325.9 | 279.41 | nd | 5.43 | 582.48 | 3070.26 |  |
| Site3 | 503.26 | 178.52 | nd | 521.67 | 89.32 | 56.41 | 537.95 | 178.52 | 267.62 | 337.63 | / | 278.62 | 67.42 | 6.32 | 4.38 | 671.57 | 3699.21 |  |
| Site4 | 89.15 | 67.54 | nd | 457.56 | 93.23 | 45.67 | 632.67 | 158.45 | 378.41 | 368.52 | / | 384.6 | 250.67 | 5.28 | 17.45 | 361.49 | 3310.69 |  |
| Site5 | 78.31 | 56.31 | nd | 378.51 | 58.41 | 67.38 | 248.58 | 220.57 | 389.51 | 278.52 | / | 490.51 | 578.41 | 5.78 | 8.9 | 631.45 | 3491.15 |  |
| Site6 | 532.57 | 129.33 | nd | 1.9 | 3.3 | 3.95 | 10.93 | 9.02 | 372.45 | nd | / | nd | 291.48 | nd | nd | nd | 1354.93 |  |
| Site7 | 707.06 | 301.98 | nd | 14.27 | 5.64 | 7.06 | 133.22 | 69.22 | 26.66 | nd | / | 4.92 | nd | nd | 61.56 | nd | 1331.59 |  |
| Site8 | 653.54 | 126.63 | nd | 89.62 | 50.32 | 43.21 | 176.83 | 6.43 | 389.31 | 43.78 | / | 56.42 | 339.41 | 67.41 | 79.51 | 62.72 | 2185.14 |  |
| Site9 | 521.72 | 233.9 | nd | 75.21 | 32.89 | 34 | 67.32 | 56.27 | 173.59 | 43.89 | / | 65.21 | 231.52 | 52.61 | 69.21 | 89.72 | 1747.06 |  |
| Site10 | 421.57 | 189.46 | nd | 63.41 | 45.31 | 41.89 | 144.52 | 43.21 | 43.72 | 52.41 | / | 61.58 | 56.32 | 52.15 | 63.32 | 68.32 | 1347.19 |  |
| Site11 | 39.73 | 4.09 | nd | 2.89 | 3.99 | 2.63 | 4.47 | 2.67 | 8.19 | nd | / | nd | nd | nd | nd | nd | 68.66 |  |
| Site12 | 3.78 | 7.02 | nd | 2.74 | 21.73 | 13.23 | 6.94 | 3.98 | 7.45 | nd | / | nd | 6.33 | 4.54 | nd | nd | 77.74 |  |
| Site13 | 3.72 | 3.38 | nd | 3.08 | 32.87 | 6.5 | 9.33 | 7.87 | 2.81 | nd | / | nd | 7.04 | 17.37 | nd | nd | 93.97 |  |
| Site14 | 40.86 | 8.18 | nd | 35.11 | 51.15 | 10.67 | 22.11 | nd | 70.99 | nd | / | 133.21 | 37.96 | nd | nd | nd | 410.24 |  |
| Site15 | 3.02 | 2.46 | nd | 5.67 | 12.91 | 5.38 | 2.29 | 2.58 | nd | 5.38 | / | 3.02 | 2.46 | 12.81 | nd | 5.67 | 63.65 |  |
| Site16 | 35.5 | 4.13 | nd | 7.68 | 47.51 | 79.18 | 3.26 | 2.93 | 123.71 | 4.67 | / | nd | 6.85 | 5.08 | nd | 2.72 | 323.22 |  |
| Site17 | 110.59 | 11.98 | nd | 25.16 | 19.68 | 18.62 | 2.17 | 2.08 | 1.62 | nd | / | 6.25 | 7.79 | nd | nd | 2.92 | 208.86 |  |
| Site18 | 52.04 | 17.02 | nd | 7.45 | 14.56 | 18.95 | 3.78 | 3.3 | 4.11 | 7.48 | / | 8.87 | nd | nd | nd | nd | 137.56 |  |
| Site19 | 63.9 | 2.26 | nd | 51.29 | 202.11 | 66.25 | 6.18 | 6.75 | 3.79 | nd | / | nd | nd | nd | nd | 7.06 | 409.59 |  |
| Site20 | 24.13 | 4.14 | nd | 3.79 | 5.06 | 5.17 | 8.84 | 5.02 | 8.64 | nd | / | nd | nd | nd | nd | nd | 64.79 |  |
| Site21 | 37.18 | 54.36 | 2.87 | 8.75 | 55.29 | 7.79 | 38.68 | 5.36 | 7.64 | 5.62 | / | nd | 58.63 | nd | nd | nd | 282.17 |  |
| Site22 | 82.87 | 75.14 | nd | 74.69 | 123.83 | 73.12 | 183.76 | 6.92 | 6.94 | nd | / | 1154.33 | 298.53 | nd | nd | 66.95 | 2147.08 |  |
| Site23 | 6.71 | 7.59 | nd | 13 | 35.58 | 26.96 | 8.66 | 40.04 | nd | 17.9 | / | 1172.14 | 343.34 | 23.83 | 8.66 | 59.39 | 1763.8 |  |
| Site24 | 5440.4 | 401.39 | nd | 194.3 | 663.26 | 23.9 | 1500.75 | 1261.98 | 872.335 | 1075.8 | / | nd | nd | 1387.04 | 90.48 | 87.59 | 12999.23 |  |
| Site25 | 1605.24 | 2363.06 | nd | 226.67 | 3564.09 | 685.61 | 338.39 | 1333.05 | 558.58 | 969.54 | / | nd | nd | nd | nd | 177.43 | 11821.66 |  |
| Site26 | 106.27 | 181.01 | nd | 6.06 | 32.77 | 19.54 | 18.86 | 26.15 | 15.6 | 61.49 | / | nd | nd | nd | nd | 61.53 | 529.28 |  |
| Site27 | 273.56 | 92.48 | nd | 1.79 | 33.81 | 220.035 | 165.79 | 1099.83 | 77.55 | nd | / | nd | nd | nd | 710.82 | 9.9 | 2685.565 |  |
| Site28 | 841.19 | 428.06 | nd | 47.78 | 282.94 | 135.87 | 395.65 | 820.89 | 234.65 | 63.88 | / | nd | nd | nd | nd | 82.71 | 3716.67 |  |
| Site29 | 1335.49 | 2539.53 | nd | 566.62 | 4001.93 | 1963.71 | 1652.42 | 2677.34 | 324.9 | 1987.93 | / | nd | nd | nd | 702.36 | 662.27 | 18414.5 |  |
| Site30 | 350.03 | 936.11 | nd | 49.62 | 160.35 | 131.33 | 155.96 | 503.59 | 353.16 | 112.14 | / | nd | nd | nd | nd | 71.86 | 2824.15 |  |
| Site31 | 1290.47 | 187.35 | nd | 30.66 | 151.12 | 181.66 | 230.21 | 295.59 | 311.71 | nd | / | 139.17 | 352.16 | 100.76 | 242.34 | 83.87 | 3597.07 |  |
| Site32 | 329.44 | 214.61 | nd | 44.26 | 211.64 | 272.05 | 220.44 | 255.55 | 294.52 | nd | / | 21.4 | 267.62 | 79.49 | 269.12 | 134.43 | 2614.57 |  |
| Site33 | 926.58 | 446.88 | nd | 14.47 | 401.47 | 180.4 | 80.12 | 462.72 | 144.57 | nd | / | 83.81 | 379.1 | 61.32 | 152.08 | 51.27 | 3384.79 |  |
| Site34 | 462.04 | 145.58 | nd | 35.97 | 277.45 | 188.46 | 191.47 | 94.07 | 129.27 | nd | / | 112.11 | 244.71 | nd | 103.77 | 120.79 | 2105.69 |  |
| Site35 | 513.07 | 146.78 | nd | 22.61 | 255.99 | 256.49 | 257.32 | 83.92 | 184.02 | nd | / | 201.05 | 269.22 | nd | 137.57 | 163.71 | 2491.75 |  |
| Site36 | 571.9 | 301.38 | nd | 286.69 | 251.96 | 196.55 | 274.38 | 1581.37 | 266.92 | nd | / | 592.93 | 2780.88 | nd | 582.4 | 202.51 | 7889.87 |  |
| Site37 | 394.33 | 645.43 | nd | 19.85 | 124.11 | 127.5 | 339.45 | 1411.47 | 402.87 | 183.01 | / | 613.54 | 2608.36 | nd | 924.62 | 180.06 | 7974.6 |  |
| Site38 | 692.46 | 484.66 | nd | 22.24 | 223.69 | 818.77 | 792.34 | 3195.53 | 1691.91 | nd | / | 616.91 | 702.8 | 339.52 | 1465.43 | 236.77 | 11283.03 |  |
| Site39 | 903.99 | 324.16 | nd | 177.51 | 295.11 | 514.25 | 504.94 | 2842.89 | 1217.85 | nd | / | 92.18 | 4787.42 | 333.14 | 1063.24 | 177.47 | 13234.15 |  |
| Site40 | 421.94 | 173.15 | nd | nd | 63.65 | 130.33 | 610.34 | 1198.09 | 134.98 | nd | / | 701.35 | 767.02 | 309.85 | 267.09 | 166.715 | 4944.505 |  |
| Site1 | 35.03 | / | / | nd | 40.92 | 57.22 | 56.02 | 61.23 | 75.4 | 58.45 | 68.86 | 97.12 | / | nd | nd | 75.55 | 787.19 | 2 |
| Site2 | 27.08 | / | / | nd | 30.76 | 44.01 | 42.59 | 45.29 | 57.95 | 44.87 | nd | 75.04 | / | nd | nd | 58.13 | 425.72 |  |
| Site3 | 33.69 | / | / | 38.08 | 42.98 | 60.68 | 59.11 | 69.26 | 73.03 | 58.8 | 67.93 | 95.39 | / | 82.71 | 74.56 | 75.94 | 990.25 |  |
| Site4 | 32.62 | / | / | 37.09 | 40.31 | 57.49 | 55.64 | 64.46 | 70.77 | 56.4 | 65.86 | 92.37 | / | 80.03 | nd | 73.01 | 878.09 |  |
| Site5 | 28.56 | / | / | 32.33 | 36.88 | 47.45 | 51.14 | 62.15 | 61.95 | 50.39 | 57.7 | 81.29 | / | 69.63 | 63 | 64.26 | 839.24 |  |
| Site6 | 27.02 | / | / | 30.7 | 35.02 | 45.15 | 48.34 | 58.38 | 58.83 | 47.82 | 54.78 | 77.08 | / | 66.36 | 59.64 | 61.61 | 795.96 |  |
| Site7 | 31.29 | / | / | 34.85 | 37.86 | 50.96 | 51.49 | 57.68 | 66.37 | 52.83 | 61.54 | 86.17 | / | 74.98 | nd | 68.03 | 816.81 |  |
| Site8 | 28.99 | / | / | 31.58 | 32.64 | 46.19 | 45.76 | 50.07 | 60.47 | 47.53 | 55.56 | 78.47 | / | 68.56 | 62.11 | 61.41 | 799.57 |  |
| Site9 | 33.11 | / | / | 36.91 | 41.47 | 53.75 | 55.36 | 63.59 | 69.29 | 55.85 | 65.13 | 91.37 | / | 79.05 | 71.35 | 71.58 | 939.18 |  |
| Site10 | 32.97 | / | / | nd | 36.13 | 50.81 | 50.04 | 54.21 | 66.46 | 52.22 | 62.86 | 88.08 | / | 76.65 | 68.33 | 68.27 | 852.5 |  |
| Site11 | 18.3 | / | / | 19.17 | 22.54 | 28.04 | 27.43 | 30.72 | 36.58 | 29.23 | 33.39 | 47.93 | / | 41.05 | 37.35 | 36.42 | 486.36 |  |
| Site12 | 38.63 | / | / | nd | 43.49 | 61.95 | 61.07 | 67.13 | 81.77 | 63.36 | 74.99 | 105.77 | / | nd | nd | 82.17 | 855.83 |  |
| Site13 | 23.61 | / | / | nd | 26.83 | 37.57 | 37.16 | 40.92 | 49.83 | 38.65 | 45.49 | 63.99 | / | nd | nd | 49.67 | 520.15 |  |
| Site14 | 30 | / | / | nd | nd | nd | 47.14 | 50.61 | 64.08 | 49.6 | nd | 83.14 | / | nd | nd | 64.41 | 469.19 |  |
| Site15 | 25.9 | / | / | nd | 25.76 | 36.76 | 40.66 | 43.64 | 55.21 | 42.72 | nd | 71.72 | / | nd | nd | 32.2 | 374.57 |  |
| Site16 | 21.79 | / | / | nd | 24.7 | 35.96 | 34.17 | 36.67 | 46.33 | 35.84 | nd | 60.3 | / | nd | nd | nd | 295.75 |  |
| Site17 | 18.63 | / | / | nd | 21.21 | 31.02 | 29.78 | 31.82 | 40.66 | 31.5 | 37.26 | 52.71 | / | nd | nd | 40.71 | 422.81 |  |
| Site18 | 26.7 | / | / | 27.69 | 44.68 | 41.38 | 44.66 | 47.48 | 51.79 | 43.29 | 48.57 | 74.23 | / | 58.27 | 52.83 | 52.29 | 728.28 |  |
| Site19 | 27.28 | / | / | 28.48 | 48.01 | 42.45 | 46.96 | 50.31 | 53.81 | 49.82 | 48.99 | 78.64 | / | 59.74 | 54.11 | 52.94 | 755.24 |  |
| Site20 | 22.4 | / | / | nd | 24.83 | 35.95 | 35.2 | 37.64 | 47.29 | 37.13 | 44.01 | 61.45 | / | 53.75 | 48.67 | 48.18 | 598.62 |  |
| Site21 | 24.05 | / | / | nd | 26.71 | 39.06 | 37.77 | 40.22 | 51.04 | 39.89 | 47.27 | 66.6 | / | 58.17 | nd | 51.75 | 592.83 |  |
| Site22 | 26.78 | / | / | 26.33 | 27.59 | 38.53 | 37.18 | 39.67 | 50.41 | 39.03 | nd | 65.62 | / | nd | nd | nd | 414.3 |  |
| Site23 | 29.25 | / | / | nd | 32.49 | 47.49 | 46.04 | 49.37 | 62.4 | 48.37 | nd | 80.42 | / | nd | nd | nd | 395.84 |  |
| Site24 | 29.27 | / | / | 29.84 | 31.38 | 43.65 | 42.22 | 44.96 | 57.11 | 43.96 | nd | nd | / | nd | nd | nd | 322.39 |  |
| Site25 | 31.5 | / | / | nd | 35.74 | 50.75 | 49.62 | 53.11 | 66.3 | 51.92 | 61.25 | 86.09 | / | nd | nd | 66.83 | 696.36 |  |
| Site26 | 28.67 | / | / | nd | nd | nd | 44.53 | 47.32 | 60.65 | 46.96 | nd | nd | / | nd | nd | nd | 228.13 |  |
| Site27 | 28.84 | / | / | nd | 32.02 | 47.01 | 45.68 | 48.66 | 62.25 | 48.23 | 56.97 | 80.36 | / | nd | nd | nd | 506.19 |  |
| Site28 | 33.07 | / | / | nd | nd | nd | 51.53 | 54.73 | 69.98 | 54.21 | nd | 90.38 | / | nd | nd | nd | 353.91 |  |
| Site29 | 22.69 | / | / | 22.76 | 23.8 | 33.17 | 32.02 | 34.15 | 43.37 | 33.47 | 39.83 | 56.4 | / | nd | nd | nd | 435.22 |  |
| Site30 | 34.82 | / | / | nd | 37.56 | 54.97 | 54.2 | 58.12 | 72.59 | 55.52 | 66.11 | 93.42 | / | nd | nd | 73.59 | 755.99 |  |
| Site31 | 38.62 | / | / | 34.09 | 36.23 | 49.74 | 48.52 | 51.96 | 64.76 | 50.61 | 59.4 | 84.2 | / | 73.28 | nd | 65.12 | 796.84 |  |
| Site32 | 28.57 | / | / | nd | 32.07 | 45.59 | 44.75 | 48.07 | 59.86 | 46.69 | 55.07 | 77.96 | / | 68.02 | nd | 60.7 | 696.43 |  |
| Site1 | 12360 | 17133 | 48457 | 23542 | 99682 | 58069 | 57022 | 92934 | 34219 | 29770 | 27779 | **/** | 37677 | 29877 | 7160 | 24091 | 599771 | 79 |
| Site2 | 726 | 275 | 1104 | 550 | 2224 | 1659 | 1677 | 1772 | 1189 | 663 | 1250 | **/** | 872 | 295 | nd | 424 | 14681 |  |
| Site3 | 221 | 317 | 266 | 322 | 2707 | 1608 | 5149 | 4271 | 3743 | 1910 | 6447 | **/** | 3973 | 2534 | nd | 2355 | 35824 |  |
| Site4 | 125 | 268 | 168 | 211 | 1843 | 904 | 3608 | 3130 | 2500 | 1309 | 3000 | **/** | 1911 | 2601 | 508 | 1740 | 23827 |  |
| Site5 | 142 | 329 | 146 | 169 | 1510 | 863 | 2955 | 2620 | 2475 | 1165 | 3034 | **/** | 1823 | 2763 | nd | 1805 | 21798 |  |
| Site6 | 176 | 400 | 163 | 240 | 1809 | 1202 | 3551 | 3198 | 2930 | 1462 | 3860 | **/** | 2451 | 2884 | 552 | 2063 | 26941 |  |
| Site7 | 5468 | 657 | 15066 | 8627 | 50218 | 32075 | 51250 | 42743 | 29444 | 23667 | 42477 | **/** | 33047 | 28138 | 5678 | 16782 | 385339 |  |
| Site8 | 177 | 539 | 162 | 233 | 1837 | 1664 | 3998 | 4561 | 4221 | 2241 | 5263 | **/** | 3502 | 3859 | 761 | 2536 | 35552 |  |
| Site9 | 289 | 348 | 252 | 400 | 2449 | 1631 | 5029 | 4459 | 3614 | 2038 | 4599 | **/** | 3024 | 3217 | 589 | 2288 | 34226 |  |
| Site10 | 196 | 281 | 289 | 345 | 3182 | 1750 | 5849 | 5203 | 4473 | 2878 | 5579 | **/** | 3768 | 3615 | nd | 2554 | 39963 |  |
| Site11 | 186 | 351 | 204 | 315 | 2427 | 1457 | 5123 | 4412 | 3942 | 1929 | 6872 | **/** | 4214 | 3732 | 624 | 2761 | 38549 |  |
| Site12 | 153 | 293 | 130 | 176 | 1489 | 873 | 2992 | 2689 | 2257 | 1196 | 3150 | **/** | 2054 | 2402 | 408 | 1683 | 21945 |  |
| Site13 | 585 | 477 | 610 | 989 | 7826 | 2932 | 11838 | 9322 | 8209 | 3949 | 7235 | **/** | 4060 | 3896 | 968 | 2537 | 65434 |  |
| Site14 | 113 | 308 | 110 | 168 | 1553 | 901 | 3457 | 2948 | 2611 | 1342 | 4575 | **/** | 2952 | 2322 | 431 | 1925 | 25716 |  |
| Site15 | 209 | 312 | 163 | 238 | 1920 | 1201 | 3739 | 3347 | 3004 | 1551 | 5503 | **/** | 3413 | 3112 | 528 | 2234 | 30473 |  |
| Site16 | 56 | 10 | 327 | 222 | 2946 | 875 | 4142 | 3332 | 2675 | 1989 | 4098 | **/** | 3575 | 7349 | 881 | 2594 | 35069 |  |
| Site17 | 2454 | 143 | 7952 | 4654 | 43220 | 21789 | 74482 | 68224 | 60070 | 57196 | 93801 | **/** | 72155 | 173329 | 25153 | 65659 | 770279 |  |
| Site18 | 41 | 2 | 57 | 39 | 347 | 125 | 542 | 479 | 423 | 291 | 662 | **/** | 544 | 963 | 108 | 411 | 5034 |  |
| Site19 | 317 | 43 | 1200 | 1019 | 9569 | 3908 | 13287 | 11930 | 9525 | 7228 | 12285 | **/** | 11364 | 24948 | 3232 | 8211 | 118065 |  |
| Site20 | 405 | 45 | 995 | 543 | 5408 | 2149 | 12778 | 11620 | 10042 | 12031 | 21206 | **/** | 16606 | 34731 | 4190 | 10380 | 143128 |  |
| Site21 | 236 | 19 | 410 | 309 | 2802 | 939 | 4438 | 3728 | 3078 | 2165 | 4371 | **/** | 3605 | 6881 | 831 | 2578 | 36390 |  |
| Site22 | 177 | 11 | 489 | 276 | 2878 | 1128 | 5497 | 4912 | 4645 | 4208 | 7144 | **/** | 6042 | 12300 | 1481 | 4637 | 55826 |  |
| Site23 | 1240 | 85 | 3164 | 2196 | 21440 | 9677 | 47260 | 39178 | 38102 | 32262 | 57955 | **/** | 40059 | 73462 | 9834 | 31894 | 407808 |  |
| Site24 | 254 | 20 | 961 | 542 | 5614 | 2087 | 9517 | 8315 | 7351 | 5785 | 9268 | **/** | 7524 | 15501 | 1933 | 6225 | 80897 |  |
| Site25 | 1139 | 216 | 2102 | 1722 | 18459 | 8864 | 32759 | 30285 | 24141 | 23847 | 39128 | **/** | 32050 | 62188 | 10203 | 24539 | 311643 |  |
| Site26 | 1979 | 74 | 4212 | 2727 | 26289 | 12155 | 55270 | 52872 | 52186 | 50797 | 108799 | **/** | 83693 | 191257 | 27932 | 52813 | 723053 |  |
| Site27 | 2971 | 70 | 4695 | 3294 | 28274 | 12710 | 48344 | 44775 | 38549 | 37569 | 70104 | **/** | 56769 | 100538 | 17837 | 37751 | 504248 |  |
| Site28 | 4531 | 126 | 9407 | 5897 | 46997 | 27042 | 82403 | 76037 | 72243 | 70647 | 102837 | **/** | 98354 | 196372 | 41238 | 74885 | 909017 |  |
| Site29 | 713 | 375 | 2291 | 1400 | 13665 | 5527 | 22345 | 20367 | 17379 | 15760 | 30720 | **/** | 25159 | 46756 | 5550 | 17724 | 225731 |  |
| Site30 | 79 | 356 | 303 | 119 | 407 | 53 | 199 | 146 | 107 | 91 | 181 | **/** | 132 | 140 | 59 | 73 | 2446 |  |
| Site31 | 212 | 12 | 504 | 313 | 3210 | 1395 | 5812 | 5083 | 4065 | 3538 | 6526 | **/** | 5804 | 7378 | 930 | 3718 | 48501 |  |
| Site32 | 5101 | 86 | 8402 | 7041 | 58239 | 24059 | 96818 | 88360 | 70067 | 69727 | 100213 | **/** | 92981 | 152879 | 22089 | 69794 | 865855 |  |
| Site33 | nd | nd | 69 | 35 | 320 | 94 | 579 | 501 | 435 | 367 | 928 | **/** | 626 | 817 | 101 | 428 | 5299 |  |
| Site34 | 1128 | 32 | 3301 | 1915 | 17722 | 7835 | 32880 | 30141 | 27010 | 26050 | 62830 | **/** | 48494 | 71745 | 10070 | 34026 | 375179 |  |
| Site35 | 2279 | 134 | 7540 | 5461 | 51421 | 24698 | 95095 | 83817 | 72383 | 68366 | 102101 | **/** | 90502 | 141917 | 19357 | 65265 | 830334 |  |
| Site36 | 284 | 361 | 837 | 606 | 5684 | 2181 | 8582 | 7415 | 5753 | 5301 | 9512 | **/** | 7568 | 10008 | 1444 | 4581 | 70117 |  |
| Site37 | 11256 | 129 | 17462 | 15286 | 90100 | 49950 | 115210 | 104486 | 74192 | 73951 | 106278 | **/** | 99337 | 147579 | 21964 | 64667 | 991847 |  |
| Site38 | 868 | 62 | 1734 | 1121 | 11867 | 4663 | 27143 | 24157 | 21544 | 20209 | 47050 | **/** | 40676 | 39592 | 4876 | 19969 | 265530 |  |
| Site39 | 1036 | 17 | 1101 | 808 | 6735 | 2904 | 11699 | 10287 | 8656 | 8440 | 13772 | **/** | 12680 | 17189 | 2266 | 8044 | 105633 |  |
| Site40 | 78 | 9 | 236 | 194 | 1831 | 722 | 2514 | 2247 | 1658 | 1452 | 2571 | **/** | 2275 | 2785 | 380 | 1424 | 20375 |  |
| Site41 | 1491 | 105 | 6427 | 3063 | 33797 | 16643 | 69868 | 65166 | 59547 | 58128 | 97173 | **/** | 96400 | 152825 | 22336 | 63977 | 746947 |  |
| Site42 | 482 | 24 | 2070 | 1112 | 11615 | 5979 | 22301 | 20907 | 20551 | 17177 | 37948 | **/** | 28563 | 46792 | 8214 | 21655 | 245394 |  |
| Site43 | 4768 | 171 | 12124 | 7870 | 77441 | 39470 | 139583 | 126080 | 106037 | 114121 | 168894 | **/** | 168810 | 292160 | 42365 | 119357 | 1419251 |  |
| Site44 | 46 | nd | 141 | 85 | 908 | 284 | 1685 | 1438 | 1346 | 991 | 2375 | **/** | 1575 | 2385 | 272 | 1092 | 14624 |  |
| Site45 | 167 | nd | 238 | 166 | 1415 | 576 | 2901 | 2395 | 1947 | 1532 | 3594 | **/** | 2417 | 2954 | 417 | 1588 | 22308 |  |
| Site46 | 19496 | 384 | 29879 | 20965 | 140040 | 88978 | 201247 | 193997 | 191515 | 190242 | 264544 | **/** | 244526 | 610053 | 93647 | 190640 | 2480153 |  |
| Site47 | 9533 | 438 | 19057 | 15845 | 113845 | 87563 | 213528 | 190356 | 197924 | 168525 | 236561 | **/** | 213169 | 480298 | 70039 | 157130 | 2173811 |  |
| Site48 | 165 | 12 | 257 | 208 | 1711 | 751 | 2893 | 2379 | 2304 | 2095 | 3128 | **/** | 2823 | 4817 | 798 | 1702 | 26044 |  |
| Site49 | 145 | 76 | 199 | 167 | 1901 | 980 | 4834 | 4248 | 6561 | 3062 | 9183 | **/** | 5596 | 4173 | 3229 | 3255 | 47609 |  |
| Site50 | 260 | 116 | 843 | 588 | 7407 | 2695 | 22029 | 20579 | 20277 | 16188 | 33558 | **/** | 20448 | 36412 | 9962 | 16407 | 207769 |  |
| Site51 | 2636 | 496 | 10158 | 8074 | 84946 | 33954 | 187338 | 169316 | 161457 | 133253 | 252988 | **/** | 158140 | 289474 | 65129 | 135203 | 1692562 |  |
| Site52 | 582 | 289 | 2214 | 1712 | 29120 | 11180 | 94042 | 83319 | 75767 | 66120 | 123285 | **/** | 68149 | 110561 | 25206 | 51652 | 743198 |  |
| Site53 | 723 | 111 | 797 | 697 | 8575 | 2807 | 12373 | 9733 | 10333 | 8246 | 16886 | **/** | 12057 | 13890 | 4145 | 7456 | 108829 |  |
| Site54 | 401 | 149 | 1591 | 1260 | 13321 | 5885 | 37549 | 33877 | 33271 | 26390 | 50837 | **/** | 35153 | 53360 | 14702 | 26960 | 334705 |  |
| Site55 | 123 | 87 | 317 | 232 | 2887 | 1347 | 8404 | 7519 | 8521 | 6299 | 14646 | **/** | 8990 | 11069 | 3764 | 5821 | 80025 |  |
| Site56 | 1050 | 111 | 5451 | 4455 | 30365 | 22519 | 41991 | 39397 | 42278 | 31771 | 54372 | **/** | 56717 | 96244 | 32954 | 30902 | 490579 |  |
| Site57 | 347 | 163 | 255 | 200 | 2124 | 645 | 3713 | 3212 | 3148 | 2316 | 6753 | **/** | 2666 | 2683 | 696 | 2192 | 31112 |  |
| Site58 | 28 | 17 | 30 | 32 | 279 | 106 | 550 | 479 | 531 | 390 | 1157 | **/** | 629 | 479 | 110 | 421 | 5237 |  |
| Site59 | 20 | 12 | 7 | 10 | 53 | 11 | 27 | 23 | 12 | 4 | 17 | **/** | 12 | 11 | 3 | 10 | 229 |  |
| Site60 | 56 | 129 | 21 | 23 | 109 | 37 | 62 | 61 | 39 | 20 | 79 | **/** | 38 | 45 | 11 | 34 | 762 |  |
| Site61 | 882 | 617 | 367 | 523 | 3153 | 1281 | 3879 | 4943 | 2709 | 2037 | 5295 | **/** | 2857 | 3375 | 583 | 1750 | 34251 |  |
| Site62 | 928 | 1261 | 653 | 754 | 7069 | 2804 | 10794 | 12222 | 7110 | 5014 | 12479 | **/** | 6335 | 9540 | 1608 | 4322 | 82892 |  |
| Site63 | 829 | 931 | 278 | 339 | 2884 | 1432 | 4694 | 6195 | 3765 | 2315 | 7136 | **/** | 3427 | 5796 | 864 | 2833 | 43717 |  |
| Site64 | 4289 | 2098 | 4081 | 4526 | 38824 | 9738 | 42179 | 40250 | 22914 | 17639 | 32636 | **/** | 18380 | 27525 | 4269 | 12033 | 281382 |  |
| Site65 | 924 | 1544 | 542 | 923 | 7121 | 2465 | 8692 | 9423 | 5200 | 3870 | 9673 | **/** | 4896 | 7003 | 1164 | 3296 | 66735 |  |
| Site66 | 5011 | 46499 | 17702 | 19605 | 58588 | 60230 | 47090 | 74909 | 31639 | 32372 | 28671 | **/** | 32729 | 14877 | 3983 | 9169 | 483076 |  |
| Site67 | 265 | 1037 | 156 | 174 | 1471 | 1109 | 2756 | 2514 | 1457 | 1242 | 2456 | **/** | 1788 | 1610 | 295 | 1019 | 19348 |  |
| Site68 | 340 | 1997 | 151 | 145 | 1229 | 1645 | 2844 | 3507 | 2434 | 1872 | 3575 | **/** | 2982 | 2425 | 407 | 1502 | 27055 |  |
| Site69 | 262 | 1715 | 149 | 144 | 1234 | 1513 | 2622 | 3135 | 2268 | 1760 | 3230 | **/** | 2756 | 2424 | 482 | 1452 | 25146 |  |
| Site70 | 553 | 4389 | 165 | 215 | 1543 | 3114 | 3218 | 9253 | 5124 | 5139 | 6345 | **/** | 6389 | 3829 | 806 | 2416 | 52497 |  |
| Site71 | 308 | 1886 | 119 | 127 | 1076 | 1449 | 2147 | 2703 | 2007 | 1665 | 2928 | **/** | 2572 | 1843 | 324 | 1197 | 22352 |  |
| Site72 | 367 | 1442 | 168 | 288 | 1349 | 1571 | 3639 | 4312 | 2852 | 2628 | 4464 | **/** | 3513 | 2748 | 500 | 1899 | 31741 |  |
| Site73 | 1351 | 17361 | 658 | 781 | 4651 | 15401 | 19047 | 40603 | 17943 | 20461 | 21373 | **/** | 23310 | 11863 | 2730 | 7000 | 204532 |  |
| Site74 | 1020 | 8673 | 416 | 432 | 2756 | 5171 | 8711 | 12867 | 7938 | 7944 | 9931 | **/** | 9454 | 5830 | 1083 | 3783 | 86008 |  |
| Site75 | 979 | 8977 | 375 | 614 | 6273 | 7579 | 11613 | 17989 | 9300 | 9003 | 12143 | **/** | 11399 | 7771 | 1481 | 4883 | 110377 |  |
| Site76 | 592 | 9810 | 384 | 359 | 2401 | 5219 | 9320 | 19495 | 9480 | 10437 | 10096 | **/** | 10438 | 5347 | 1090 | 3652 | 98120 |  |
| Site77 | 1238 | 15376 | 1233 | 1309 | 6702 | 23677 | 12966 | 35970 | 14977 | 16992 | 16243 | **/** | 19502 | 7632 | 2085 | 4964 | 180868 |  |
| Site78 | 4433 | 16869 | 1278 | 1642 | 10596 | 15686 | 28929 | 28431 | 18695 | 19588 | 23713 | **/** | 22466 | 17995 | 4125 | 9438 | 223884 |  |
| Site79 | 6704 | 34272 | 21538 | 22952 | 69118 | 71077 | 40746 | 62385 | 26081 | 27570 | 23891 | **/** | 27905 | 11533 | 3062 | 7263 | 456098 |  |
| Site80 | 150815 | 398944 | 194879 | 284544 | 865712 | 709168 | 1145281 | 244726 | 138636 | 120376 | 96299 | **/** | 127480 | 49297 | 14049 | 29553 | 4569759 |  |
| Site81 | 3121 | 15844 | 484 | 557 | 2581 | 36422 | 35516 | 55701 | 21159 | 23665 | 21204 | **/** | 24880 | 12794 | 2797 | 7281 | 264007 |  |
| Site82 | 1141 | 17286 | 1037 | 1006 | 4704 | 11878 | 21716 | 36278 | 18542 | 20856 | 24048 | **/** | 25517 | 13529 | 2822 | 8487 | 208846 |  |
| Site83 | 602 | 3613 | 283 | 553 | 3169 | 7099 | 8997 | 7958 | 7350 | 6324 | 9274 | **/** | 6818 | 4630 | 889 | 2767 | 70325 |  |
| Site84 | 3118 | 16442 | 1035 | 1033 | 4185 | 12769 | 13313 | 24882 | 14739 | 15383 | 16778 | **/** | 18864 | 11809 | 3069 | 6431 | 163850 |  |
| Site85 | 862 | 11152 | 529 | 419 | 2056 | 7558 | 8122 | 20741 | 9903 | 11037 | 12942 | **/** | 14570 | 7245 | 1870 | 4449 | 113456 |  |
| Site86 | 787 | 4528 | 504 | 1297 | 6924 | 8197 | 10579 | 11148 | 6704 | 6539 | 7478 | **/** | 6746 | 4332 | 806 | 2529 | 79098 |  |
| Site87 | 1822 | 17639 | 671 | 627 | 2118 | 14255 | 16988 | 43995 | 17067 | 19675 | 19317 | **/** | 20869 | 12740 | 2612 | 7550 | 197945 |  |
| Site88 | 1010 | 13199 | 623 | 513 | 2635 | 9741 | 9888 | 22978 | 12913 | 14230 | 17105 | **/** | 19005 | 9053 | 2088 | 5753 | 140735 |  |
| Site89 | 463 | 8528 | 230 | 209 | 1090 | 4657 | 6405 | 14702 | 7321 | 7776 | 7958 | **/** | 9199 | 4547 | 1301 | 2703 | 77090 |  |
| Site90 | 517 | 7276 | 468 | 399 | 2064 | 7858 | 6132 | 10938 | 6336 | 5468 | 7013 | **/** | 7528 | 4713 | 885 | 2808 | 70404 |  |
| Site91 | 140 | 1052 | 152 | 112 | 750 | 885 | 1056 | 1385 | 1026 | 829 | 1358 | **/** | 1228 | 971 | 165 | 586 | 11695 |  |
| Site92 | 35606 | 5905 | 34511 | 15727 | 61385 | 25296 | 25845 | 33396 | 13694 | 12960 | 8352 | **/** | 13310 | 13861 | 3033 | 6713 | 309594 |  |
| Site93 | 829 | 226 | 448 | 311 | 1517 | 565 | 1370 | 1386 | 775 | 681 | 633 | **/** | 560 | 711 | 216 | 381 | 10609 |  |
| Site94 | 135359 | 16469 | 62617 | 36973 | 119006 | 64531 | 74692 | 76714 | 33151 | 34503 | 23112 | **/** | 34212 | 41451 | 9023 | 17195 | 779008 |  |
| Site95 | 4101 | 10721 | 6219 | 5858 | 35713 | 17678 | 40645 | 41430 | 21757 | 23211 | 17422 | **/** | 20818 | 34278 | 9475 | 12925 | 302250 |  |
| Site96 | 1173955 | 35140 | 391480 | 255618 | 1136797 | 391651 | 543329 | 524951 | 190749 | 209861 | 214636 | **/** | 195016 | 258186 | 87634 | 94910 | 5703912 |  |
| Site97 | 3952 | 891 | 932 | 609 | 2895 | 1102 | 3585 | 4435 | 2237 | 1661 | 2673 | **/** | 1828 | 2625 | 508 | 1240 | 31174 |  |
| Site98 | 8124 | 2398 | 5752 | 4082 | 12580 | 4384 | 9883 | 10083 | 4814 | 4471 | 4034 | **/** | 4044 | 10886 | 2033 | 4097 | 91665 |  |
| Site99 | 253 | 312 | 71 | 96 | 587 | 445 | 1083 | 1011 | 770 | 507 | 608 | **/** | 549 | 1597 | 342 | 544 | 8776 |  |
| Site100 | 944 | 1042 | 820 | 1054 | 12693 | 3909 | 24116 | 19276 | 12601 | 9959 | 9729 | **/** | 8291 | 21855 | 6369 | 7461 | 140118 |  |
| Site101 | 1298 | 1269 | 484 | 656 | 8669 | 2533 | 19858 | 15999 | 11039 | 7458 | 8407 | **/** | 6726 | 29095 | 5333 | 9072 | 127895 |  |
| Site102 | 961 | 782 | 322 | 471 | 6304 | 2054 | 14859 | 11639 | 8520 | 6073 | 7020 | **/** | 6000 | 16679 | 4657 | 5642 | 91983 |  |
| Site103 | 403 | 555 | 110 | 165 | 942 | 868 | 1819 | 1647 | 1236 | 878 | 1199 | **/** | 737 | 1484 | 274 | 632 | 12950 |  |
| Site104 | 242 | 283 | 67 | 90 | 562 | 384 | 1068 | 996 | 708 | 505 | 693 | **/** | 538 | 1050 | 175 | 464 | 7828 |  |
| Site105 | 96 | 37 | 108 | 59 | 536 | 201 | 427 | 523 | 180 | 152 | 247 | **/** | 160 | 112 | 58 | 88 | 2985 |  |
| Site106 | 371 | 228 | 146 | 160 | 838 | 525 | 1066 | 1226 | 748 | 614 | 1172 | **/** | 708 | 595 | 149 | 395 | 8943 |  |
| Site107 | 5116 | 1081 | 2070 | 1698 | 11262 | 6875 | 13953 | 12359 | 6498 | 7223 | 8846 | **/** | 6701 | 3931 | 775 | 2519 | 90905 |  |
| Site108 | 292 | 313 | 200 | 211 | 1823 | 1170 | 2315 | 2657 | 1227 | 1296 | 1576 | **/** | 1288 | 996 | 201 | 715 | 16279 |  |
| Site109 | 71045 | 35037 | 113870 | 103268 | 415648 | 293820 | 330142 | 309303 | 143293 | 157681 | 167647 | **/** | 140326 | 110469 | 22520 | 62592 | 2476662 |  |
| Site110 | 8206 | 2451 | 10946 | 8801 | 33800 | 19996 | 23955 | 22324 | 10736 | 13397 | 12881 | **/** | 10233 | 8871 | 1891 | 4606 | 193094 |  |
| Site111 | 121560 | 25773 | 132187 | 136753 | 462946 | 317697 | 306266 | 287721 | 144257 | 155211 | 172693 | **/** | 137424 | 100246 | 20403 | 54893 | 2576031 |  |
| Site112 | 1047185 | 106433 | 675407 | 546604 | 1442981 | 1202292 | 741293 | 737322 | 396328 | 372836 | 418004 | **/** | 339067 | 314210 | 72114 | 165274 | 8577348 |  |
| Site113 | 1793 | 1171 | 489 | 463 | 3677 | 2299 | 6538 | 6635 | 4006 | 4062 | 5103 | **/** | 3852 | 3904 | 801 | 2342 | 47135 |  |
| Site114 | 3431 | 1726 | 2158 | 1298 | 7876 | 7301 | 14868 | 19506 | 9320 | 10307 | 10572 | **/** | 9874 | 6327 | 1225 | 4327 | 110115 |  |
| Site115 | 7385 | 2875 | 8128 | 7972 | 38768 | 25953 | 41122 | 34661 | 16516 | 19496 | 19426 | **/** | 16481 | 13086 | 2724 | 6384 | 260978 |  |
| Site116 | 20976 | 11517 | 24499 | 24161 | 101141 | 80865 | 103191 | 89723 | 50851 | 53796 | 58466 | **/** | 49857 | 45356 | 11168 | 20773 | 746339 |  |
| Site117 | 4166 | 1967 | 3965 | 2832 | 14899 | 11828 | 21503 | 19988 | 11383 | 12693 | 14350 | **/** | 11182 | 8330 | 1534 | 4578 | 145198 |  |
| Site118 | 9570 | 4483 | 11251 | 8206 | 51679 | 44499 | 53097 | 55393 | 30322 | 31229 | 30640 | **/** | 25435 | 27466 | 6256 | 14193 | 403720 |  |
| Site119 | 3262 | 384 | 1654 | 1150 | 5233 | 3621 | 5103 | 4852 | 2636 | 3015 | 3026 | **/** | 2303 | 1640 | 364 | 902 | 39145 |  |
| Site120 | 1282 | 550 | 1282 | 1391 | 7664 | 5971 | 10150 | 8393 | 5395 | 6067 | 5799 | **/** | 4512 | 3580 | 813 | 1779 | 64629 |  |
| Site121 | 1385 | 348 | 801 | 686 | 3898 | 2459 | 5266 | 5099 | 2477 | 2954 | 2861 | **/** | 2269 | 1563 | 311 | 854 | 33231 |  |
| Site122 | 272 | 121 | 115 | 133 | 1082 | 528 | 1251 | 1414 | 834 | 767 | 1030 | **/** | 757 | 550 | 115 | 391 | 9360 |  |
| Site123 | 2853 | 361 | 1224 | 676 | 2642 | 2885 | 3245 | 3145 | 1564 | 1424 | 1628 | **/** | 1099 | 856 | 193 | 536 | 24331 |  |
| Site124 | 1197283 | 50220 | 546793 | 340465 | 956865 | 678986 | 514776 | 492043 | 224388 | 239002 | 259807 | **/** | 211176 | 175444 | 32006 | 95215 | 6014468 |  |
| Site125 | 105 | 76 | 57 | 77 | 396 | 293 | 1343 | 1114 | 610 | 732 | 1228 | **/** | 731 | 390 | 78 | 403 | 7632 |  |
| Site126 | 9458 | 7075 | 18412 | 16723 | 75774 | 48554 | 75539 | 63313 | 37168 | 46516 | 59241 | **/** | 46284 | 32481 | 5369 | 18007 | 559914 |  |
| Site127 | 1620 | 686 | 1374 | 1183 | 6163 | 4518 | 9748 | 8301 | 4623 | 5600 | 6642 | **/** | 5088 | 3321 | 502 | 1965 | 61334 |  |
| Site128 | 2996 | 1434 | 3768 | 3405 | 13905 | 9400 | 14602 | 13122 | 6799 | 8346 | 9717 | **/** | 7676 | 5380 | 864 | 3037 | 104451 |  |
| Site129 | 385 | 196 | 295 | 196 | 1051 | 645 | 1114 | 1022 | 401 | 420 | 595 | **/** | 340 | 198 | 34 | 170 | 7062 |  |
| Site130 | 93540 | 4658 | 47705 | 20769 | 67798 | 38089 | 20079 | 30389 | 11809 | 13074 | 10396 | **/** | 12420 | 5890 | 950 | 4332 | 381896 |  |
| Site131 | 33545 | 5330 | 37164 | 17418 | 58230 | 36097 | 18183 | 28578 | 11587 | 11501 | 7849 | **/** | 8497 | 4276 | 1130 | 2676 | 282062 |  |
| Site132 | 423618 | 99899 | 371204 | 254711 | 793400 | 531551 | 322546 | 381161 | 323407 | 235458 | 316694 | **/** | 291131 | 264117 | 74869 | 137356 | 4821122 |  |
| Site133 | 90197 | 103807 | 103050 | 98424 | 356810 | 264101 | 262414 | 351372 | 230776 | 184790 | 231497 | **/** | 200151 | 183911 | 51253 | 97664 | 2810216 |  |
| Site134 | 701566 | 131510 | 261418 | 155263 | 532797 | 402229 | 394479 | 512697 | 347912 | 226555 | 329907 | **/** | 302380 | 267407 | 88696 | 143196 | 4798013 |  |
| Site135 | 345 | 368 | 119 | 121 | 928 | 696 | 1776 | 1571 | 1242 | 747 | 1602 | **/** | 1010 | 1061 | 180 | 609 | 12375 |  |
| Site136 | 13707 | 3973 | 4209 | 7358 | 34905 | 13414 | 21073 | 20769 | 11110 | 11184 | 12136 | **/** | 9395 | 13519 | 2061 | 5533 | 184348 |  |
| Site137 | 225 | 195 | 195 | 206 | 751 | 514 | 962 | 894 | 534 | 453 | 455 | **/** | 437 | 366 | 67 | 256 | 6511 |  |
| Site138 | 67 | 36 | 47 | 38 | 88 | 83 | 102 | 119 | 62 | 46 | 69 | **/** | 68 | nd | nd | nd | 826 |  |
| Site139 | 99 | 44 | 78 | 78 | 386 | 208 | 513 | 438 | 263 | 162 | 318 | **/** | 187 | 156 | nd | 73 | 3005 |  |
| Site140 | 2748 | 1778 | 6491 | 6625 | 19259 | 12877 | 17610 | 17027 | 7322 | 6176 | 5265 | **/** | 4413 | 4219 | 701 | 3384 | 115894 |  |
| Site141 | 146 | 108 | 92 | 57 | 187 | 236 | 371 | 417 | 248 | 188 | 108 | **/** | 186 | 85 | nd | 109 | 2540 |  |
| Site142 | 84 | 50 | 110 | 88 | 216 | 142 | 250 | 227 | 166 | 96 | 210 | **/** | 119 | 56 | nd | 89 | 1904 |  |
| Site143 | 231 | 93 | 94 | 87 | 313 | 279 | 409 | 420 | 206 | 208 | 265 | **/** | 172 | 126 | nd | 85 | 2987 |  |
| Site144 | 277 | 439 | 148 | 197 | 1148 | 798 | 1990 | 1725 | 1218 | 1032 | 1655 | **/** | 1103 | 815 | nd | 492 | 13038 |  |
| Site145 | 209 | 170 | 327 | 326 | 982 | 659 | 865 | 921 | 332 | 278 | 237 | **/** | 215 | 18 | nd | 25 | 5564 |  |
| Site146 | 784 | 2786 | 1636 | 2448 | 10314 | 8727 | 8840 | 11662 | 4805 | 4002 | 3092 | **/** | 3015 | 2168 | 369 | 1832 | 66480 |  |
| Site147 | 1456 | 1280 | 1180 | 1892 | 9055 | 4835 | 9221 | 6834 | 3663 | 2962 | 4045 | **/** | 2878 | 2353 | 344 | 2027 | 54025 |  |
| Site148 | 973 | 2460 | 353 | 360 | 2745 | 4527 | 12374 | 10022 | 7340 | 8312 | 8120 | **/** | 7613 | 9691 | 2069 | 3685 | 80645 |  |
| Site149 | 322 | 425 | 309 | 392 | 1456 | 1007 | 2250 | 2010 | 1082 | 738 | 1367 | **/** | 815 | 961 | 179 | 530 | 13843 |  |
| Site150 | 180 | 493 | 211 | 302 | 1582 | 1347 | 2939 | 2639 | 1759 | 1172 | 1920 | **/** | 1314 | 1237 | 212 | 643 | 17950 |  |
| Site151 | 79 | 216 | 50 | 80 | 661 | 459 | 1259 | 1081 | 743 | 595 | 1158 | **/** | 644 | 498 | nd | 321 | 7844 |  |
| Site152 | 78 | 141 | 105 | 78 | 460 | 272 | 1083 | 962 | 655 | 414 | 940 | **/** | 553 | 823 | 93 | 427 | 7084 |  |
| Site153 | 179 | 261 | 634 | 451 | 3048 | 1407 | 2853 | 2797 | 1654 | 1449 | 1561 | **/** | 1295 | 1485 | 209 | 675 | 19956 |  |
| Site154 | 281 | 1624 | 1374 | 1123 | 5521 | 6732 | 6293 | 7778 | 4156 | 3195 | 3358 | **/** | 3500 | 1594 | 267 | 1219 | 48014 |  |
| Site155 | 45 | nd | 26 | 29 | 87 | 27 | 87 | 82 | 38 | 26 | 47 | **/** | 28 | 24 | 0 | 18 | 565 |  |
| Site156 | 17712 | 1854 | 13980 | 5692 | 21238 | 12729 | 6138 | 9038 | 4358 | 3414 | 3102 | **/** | 3625 | 1784 | 355 | 1187 | 106206 |  |
| Site157 | 30 | 58 | 25 | 42 | 296 | 161 | 336 | 308 | 193 | 137 | 238 | **/** | 154 | 166 | 23 | 96 | 2262 |  |
| Site158 | 206 | 109 | 236 | 261 | 1945 | 4908 | 23938 | 18758 | 67815 | 23960 | 64540 | **/** | 23833 | 25420 | 8481 | 18761 | 283170 |  |
| Site159 | 218 | 108 | 182 | 193 | 1402 | 2903 | 14200 | 12954 | 47883 | 16718 | 47912 | **/** | 16869 | 17841 | 6456 | 13736 | 199575 |  |
| Site160 | 250 | 198 | 4136 | 3952 | 23042 | 37184 | 121156 | 104900 | 200893 | 85008 | 188189 | **/** | 72429 | 107833 | 31619 | 57856 | 1038646 |  |
| Site161 | 392 | 139 | 2652 | 3638 | 11475 | 27046 | 91480 | 65839 | 141639 | 55185 | 135751 | **/** | 45871 | 61055 | 16073 | 33922 | 692156 |  |
| Site162 | 405 | 169 | 2577 | 4124 | 13926 | 51348 | 87796 | 65620 | 159031 | 64553 | 127291 | **/** | 49443 | 57239 | 17171 | 31395 | 732090 |  |
| Site163 | 355 | 50 | 771 | 970 | 4597 | 15084 | 12814 | 9369 | 58849 | 17922 | 56096 | **/** | 18999 | 24142 | 6104 | 14417 | 240538 |  |
| Site164 | 772 | 228 | 4803 | 8447 | 44864 | 83730 | 181280 | 140083 | 307886 | 142580 | 238665 | **/** | 103514 | 123193 | 38253 | 63711 | 1482008 |  |
| Site165 | 182 | 94 | 189 | 263 | 1703 | 3998 | 23297 | 17044 | 68521 | 25916 | 81526 | **/** | 28310 | 31489 | 8458 | 19342 | 310333 |  |
| Site166 | 95 | 79 | 67 | 57 | 346 | 411 | 1053 | 916 | 6348 | 1749 | 11405 | **/** | 2680 | 3744 | 798 | 2684 | 32431 |  |
| Site167 | 362 | 126 | 1566 | 3063 | 11368 | 25104 | 68063 | 53114 | 149752 | 56688 | 114816 | **/** | 40003 | 60165 | 17284 | 30302 | 631779 |  |
| Site168 | 632 | 215 | 7006 | 8075 | 42074 | 76209 | 172945 | 139312 | 253708 | 113483 | 237984 | **/** | 82214 | 105670 | 27066 | 63135 | 1329727 |  |
| Site169 | 2112 | 937 | 273 | 433 | 1758 | 527 | 833 | 635 | 260 | 302 | 326 | **/** | 286 | 299 | 57 | 141 | 9179 |  |
| Site170 | 5741 | 781 | 1182 | 701 | 2879 | 1409 | 1892 | 1544 | 695 | 767 | 846 | **/** | 739 | 749 | 128 | 357 | 20411 |  |
| Site171 | 42380 | 4097 | 35816 | 10768 | 49468 | 23179 | 23617 | 31211 | 10499 | 11059 | 10713 | **/** | 12786 | 11099 | 1521 | 6587 | 284800 |  |
| Site172 | 73186 | 65101 | 26695 | 61967 | 210074 | 185030 | 120043 | 129317 | 104895 | 71760 | 85315 | **/** | 75982 | 92097 | 22039 | 40161 | 1363663 |  |
| Site173 | 35389 | 2882 | 19456 | 9965 | 41100 | 22175 | 27963 | 24243 | 9653 | 11489 | 12165 | **/** | 11388 | 11541 | 1921 | 5362 | 246692 |  |
| Site174 | 1263316 | 377379 | 104637 | 272177 | 858889 | 441263 | 450116 | 480971 | 208796 | 236498 | 290930 | **/** | 260773 | 293673 | 51524 | 129485 | 5720427 |  |
| Site175 | 4692241 | 1370135 | 282063 | 785001 | 2992826 | 1751285 | 974773 | 890590 | 666818 | 613958 | 717526 | **/** | 683606 | 863870 | 189007 | 401264 | 17874963 |  |
| Site176 | 1731893 | 512648 | 151184 | 338149 | 1309824 | 642969 | 691673 | 680830 | 256674 | 292811 | 363685 | **/** | 350877 | 333383 | 77794 | 155193 | 7889588 |  |
| Site177 | 590013 | 151185 | 39665 | 110756 | 407809 | 181226 | 251371 | 216452 | 79720 | 104476 | 113211 | **/** | 100659 | 125859 | 18711 | 50082 | 2541196 |  |
| Site178 | 1904366 | 434097 | 283220 | 358019 | 1414738 | 846798 | 589878 | 641826 | 293614 | 320584 | 405005 | **/** | 391064 | 350806 | 80669 | 163644 | 8478327 |  |
| Site179 | 3082 | 1667 | 3152 | 2355 | 16649 | 9190 | 18407 | 16768 | 9871 | 10392 | 13657 | **/** | 10547 | 11655 | 2702 | 6037 | 136131 |  |
| Site180 | 1915 | 1892 | 2702 | 1602 | 6768 | 4446 | 7339 | 9299 | 5273 | 4994 | 5448 | **/** | 4992 | 4201 | 1150 | 2419 | 64439 |  |
| Site181 | 1829 | 2310 | 2278 | 1627 | 9381 | 5675 | 9455 | 10533 | 6575 | 5549 | 6935 | **/** | 5778 | 6798 | 1283 | 3170 | 79178 |  |
| Site182 | 6030 | 5832 | 16627 | 8486 | 34441 | 22668 | 22111 | 25445 | 14605 | 13870 | 14302 | **/** | 13210 | 13859 | 3480 | 6764 | 221731 |  |
| Site183 | 4043 | 4381 | 3415 | 2507 | 24951 | 11663 | 27895 | 24746 | 16049 | 14398 | 19387 | **/** | 14971 | 17573 | 4728 | 8883 | 199589 |  |
| Site184 | 716 | 885 | 1201 | 767 | 3416 | 2099 | 5187 | 4919 | 3192 | 2908 | 3983 | **/** | 3048 | 3686 | 655 | 1769 | 38431 |  |
| Site185 | 454 | 881 | 299 | 285 | 2209 | 1578 | 3634 | 3450 | 2367 | 2041 | 3351 | **/** | 2453 | 2626 | 634 | 1471 | 27734 |  |
| Site186 | 132 | 171 | 94 | 103 | 592 | 398 | 968 | 897 | 647 | 502 | 992 | **/** | 578 | 621 | 136 | 384 | 7216 |  |
| Site187 | 136 | 175 | 102 | 107 | 743 | 430 | 1125 | 1008 | 714 | 598 | 1068 | **/** | 647 | 744 | 166 | 415 | 8180 |  |
| Site188 | 2470 | 2349 | 1234 | 1144 | 9113 | 5521 | 14064 | 13259 | 8412 | 7391 | 10594 | **/** | 8024 | 9943 | 1557 | 4887 | 99962 |  |
| Site189 | 16655 | 22291 | 103229 | 63116 | 219870 | 191044 | 104547 | 140049 | 64886 | 65695 | 53828 | **/** | 67722 | 53835 | 14941 | 27452 | 1209159 |  |
| Site190 | 6944 | 5250 | 14053 | 9245 | 35396 | 30742 | 23004 | 30392 | 15689 | 15490 | 13304 | **/** | 14304 | 11934 | 3035 | 5815 | 234597 |  |
| Site191 | 1582 | 769 | 2085 | 1440 | 5696 | 4008 | 3522 | 5064 | 2821 | 2664 | 2251 | **/** | 2237 | 2007 | 453 | 1009 | 37608 |  |
| Site192 | 4154 | 2144 | 4279 | 3283 | 14201 | 10866 | 11734 | 17333 | 8799 | 8758 | 6899 | **/** | 7821 | 6116 | 1249 | 2981 | 110617 |  |
| Site193 | 9125 | 4466 | 20079 | 9229 | 36174 | 24051 | 21843 | 26426 | 14135 | 14082 | 13790 | **/** | 14095 | 14659 | 2637 | 6384 | 231177 |  |
| Site194 | 1006 | 4961 | 422 | 477 | 5927 | 5535 | 15027 | 23156 | 13782 | 8284 | 17166 | **/** | 10623 | 12333 | 3034 | 7267 | 129001 |  |
| Site195 | 631 | 3390 | 413 | 498 | 5300 | 3182 | 15865 | 22541 | 11164 | 9712 | 16275 | **/** | 10867 | 15517 | 2507 | 7119 | 124981 |  |
| Site196 | 4681 | 8035 | 20543 | 8216 | 58220 | 31606 | 46507 | 68244 | 31112 | 20236 | 32467 | **/** | 21737 | 25580 | 6047 | 14661 | 397894 |  |
| Site197 | 721 | 1804 | 847 | 438 | 4873 | 3784 | 13022 | 16996 | 7596 | 6801 | 12454 | **/** | 7590 | 9400 | 1462 | 4658 | 92447 |  |
| Site198 | 4415 | 11990 | 8905 | 1260 | 13471 | 21938 | 36542 | 66843 | 32678 | 23365 | 29411 | **/** | 25365 | 16152 | 3785 | 9208 | 305329 |  |
| Site199 | 680 | 2192 | 529 | 452 | 4443 | 4973 | 11971 | 14266 | 7630 | 6944 | 9563 | **/** | 7153 | 6984 | 1172 | 4322 | 83274 |  |
| Site200 | 512 | 3302 | 586 | 684 | 7061 | 6595 | 16920 | 18618 | 10678 | 10086 | 13073 | **/** | 10039 | 10031 | 1840 | 5931 | 115957 |  |
| Site201 | 422 | 1937 | 347 | 354 | 3911 | 3382 | 9701 | 10772 | 5924 | 5170 | 9019 | **/** | 8561 | 5432 | 1071 | 3108 | 69110 |  |
| Site202 | 242 | 554 | 381 | 294 | 3102 | 1708 | 5951 | 5803 | 2998 | 2536 | 4999 | **/** | 4156 | 2607 | 509 | 1409 | 37251 |  |
| Site203 | 708 | 4827 | 686 | 926 | 9416 | 8097 | 25943 | 27453 | 13864 | 12775 | 16572 | **/** | 12943 | 10095 | 2060 | 7264 | 153629 |  |
| Site204 | 2141 | 6866 | 320 | 537 | 7088 | 8470 | 17526 | 32147 | 19142 | 18169 | 18930 | **/** | 16298 | 16469 | 3683 | 10973 | 178759 |  |
| Site205 | 1363 | 6390 | 447 | 712 | 6469 | 7162 | 13595 | 18767 | 13277 | 11466 | 15992 | **/** | 15301 | 11838 | 2675 | 8888 | 134342 |  |
| Site206 | 1919 | 6561 | 498 | 654 | 6930 | 9631 | 17547 | 25993 | 16221 | 15444 | 19143 | **/** | 16646 | 11823 | 2903 | 10454 | 162370 |  |
| Site207 | 1259 | 5250 | 378 | 552 | 5660 | 6615 | 16181 | 26649 | 13507 | 12312 | 15837 | **/** | 14993 | 10403 | 2187 | 8779 | 140563 |  |
| Site208 | 794 | 730 | 273 | 419 | 5195 | 1405 | 11181 | 9229 | 6586 | 4841 | 8948 | **/** | 5176 | 6899 | 1776 | 3939 | 67390 |  |
| Site209 | 3228 | 678 | 841 | 1422 | 5091 | 2256 | 5744 | 6100 | 3092 | 3106 | 5654 | **/** | 3017 | 2617 | 590 | 1703 | 45139 |  |
| Site210 | 4812 | 10488 | 2810 | 7265 | 49321 | 13449 | 57683 | 66037 | 33929 | 28634 | 41145 | **/** | 23536 | 21882 | 5551 | 13198 | 379740 |  |
| Site211 | 2601 | 7891 | 2490 | 6492 | 38716 | 11421 | 72971 | 68853 | 38625 | 34141 | 53102 | **/** | 34842 | 30947 | 6700 | 18870 | 428662 |  |
| Site212 | 72 | 302 | 28 | 34 | 234 | 181 | 667 | 779 | 449 | 440 | 1217 | **/** | 560 | 569 | 109 | 395 | 6036 |  |
| Site213 | 744 | 4883 | 332 | 373 | 1870 | 2048 | 6074 | 9673 | 6170 | 6329 | 9564 | **/** | 8149 | 6058 | 1140 | 4173 | 67579 |  |
| Site214 | 682 | 5586 | 1060 | 985 | 5379 | 3658 | 9773 | 14019 | 7694 | 7994 | 11334 | **/** | 9392 | 7719 | 1836 | 4577 | 91687 |  |
| Site215 | 381158 | 25470 | 140343 | 19297 | 277791 | 97987 | 85055 | 152757 | 37582 | 45659 | 33100 | **/** | 41944 | 24276 | 5154 | 16873 | 1384445 |  |
| Site216 | 1049 | 16831 | 4684 | 1800 | 6555 | 10372 | 20066 | 39446 | 18721 | 21743 | 28263 | **/** | 33049 | 19677 | 4285 | 14018 | 240558 |  |
| Site217 | 4604 | 2036 | 5349 | 2739 | 11336 | 4000 | 4977 | 7216 | 2297 | 2438 | 3030 | **/** | 2255 | 938 | 230 | 1035 | 54478 |  |
| Site218 | 612 | 5648 | 11365 | 6971 | 27829 | 13848 | 19303 | 28918 | 10644 | 11116 | 10632 | **/** | 9189 | 6245 | 1287 | 4674 | 168279 |  |
| Site219 | 230 | 1013 | 348 | 796 | 6287 | 2109 | 8987 | 8441 | 4412 | 3899 | 8009 | **/** | 3759 | 2586 | 451 | 2146 | 53472 |  |
| Site220 | 71 | 637 | 97 | 69 | 432 | 500 | 1019 | 1284 | 751 | 817 | 952 | **/** | 970 | 743 | 114 | 404 | 8859 |  |
| Site221 | 17 | 73 | 41 | 21 | 145 | 81 | 208 | 188 | 107 | 112 | 212 | **/** | 186 | nd | 23 | 93 | 1507 |  |
| Site222 | nd | 48 | 26 | 20 | 107 | 58 | 160 | 153 | 115 | 106 | 178 | **/** | 144 | 92 | nd | 61 | 1268 |  |
| Site223 | 45 | 961 | 86 | 80 | 531 | 684 | 1275 | 1613 | 1097 | 1266 | 1380 | **/** | 1483 | 1287 | 191 | 612 | 12592 |  |
| Site224 | 670 | 2233 | 223 | 181 | 1111 | 1472 | 2406 | 3344 | 2198 | 2576 | 2813 | **/** | 3075 | 2576 | 400 | 1238 | 26515 |  |
| Site225 | 180 | 566 | 468 | 322 | 1726 | 1259 | 1657 | 2060 | 919 | 1055 | 1167 | **/** | 1078 | 922 | 141 | 463 | 13984 |  |
| Site226 | 977 | 7406 | 5133 | 1777 | 9426 | 8474 | 14543 | 20397 | 9536 | 10525 | 15714 | **/** | 14059 | 16832 | 2050 | 6042 | 142891 |  |
| Site227 | 1970 | 12556 | 471 | 183 | 960 | 6617 | 9991 | 26006 | 10400 | 10852 | 20701 | **/** | 21254 | 28164 | 3511 | 10029 | 163663 |  |
| Site228 | 9790 | 49806 | 60209 | 10798 | 97012 | 92431 | 104342 | 91337 | 69396 | 73995 | 80305 | **/** | 76929 | 122386 | 14507 | 37795 | 991039 |  |
| Site229 | 783 | 22813 | 632 | 192 | 1505 | 11247 | 29623 | 55467 | 28101 | 33759 | 55325 | **/** | 64113 | 86794 | 9555 | 35586 | 435496 |  |
| Site230 | 32218 | 6540 | 1483 | 1708 | 8021 | 5223 | 10496 | 15295 | 9254 | 6386 | 12464 | **/** | 9937 | 11040 | 1742 | 6923 | 138730 |  |
| Site231 | 15942 | 11218 | 11423 | 7441 | 41100 | 20029 | 39220 | 41999 | 23953 | 17057 | 28811 | **/** | 21902 | 24740 | 3631 | 13734 | 322199 |  |
| Site232 | 73038 | 1875 | 27673 | 8389 | 23938 | 7430 | 23778 | 20122 | 11566 | 8879 | 15139 | **/** | 9934 | 12054 | 1832 | 6511 | 252156 |  |
| Site233 | 2003890 | 159054 | 1003212 | 405432 | 1260173 | 787805 | 350200 | 525411 | 229324 | 206177 | 171831 | **/** | 178131 | 149882 | 36884 | 84283 | 7551688 |  |
| Site234 | 407380 | 1717 | 164414 | 28574 | 74894 | 26171 | 4569 | 5345 | 1655 | 1335 | 1396 | **/** | 1114 | 1121 | 238 | 741 | 720664 |  |
| Site235 | 31229 | 11540 | 5743 | 12350 | 51569 | 31172 | 37052 | 33174 | 15179 | 16212 | 18254 | **/** | 16144 | 16108 | 2211 | 7718 | 305655 |  |
| Site236 | 1192 | 1047 | 3202 | 2508 | 10348 | 6221 | 6084 | 7879 | 4525 | 4137 | 3436 | **/** | 4009 | 3896 | 792 | 1914 | 61190 |  |
| Site237 | nd | 64 | 31 | 11 | 59 | 53 | 141 | 165 | 220 | 193 | 476 | **/** | 379 | 443 | 63 | 208 | 2505 |  |
| Site238 | 203428 | 24901 | 183153 | 133023 | 521416 | 289672 | 339848 | 308880 | 128096 | 142311 | 168899 | **/** | 152908 | 202864 | 25959 | 87433 | 2912793 |  |
| Site239 | nd | 26 | 15 | 8 | 78 | 10 | 97 | 88 | 105 | 90 | 246 | **/** | 170 | 155 | 25 | 88 | 1201 |  |
| Site240 | 40 | 63 | 80 | 49 | 240 | 118 | 240 | 267 | 245 | 234 | 511 | **/** | 380 | 342 | 49 | 195 | 3054 |  |
| Site241 | 28 | 45 | 37 | nd | 111 | 67 | 156 | 181 | 222 | 154 | 275 | **/** | 226 | 234 | 40 | 112 | 1887 |  |
| Site242 | 480018 | 120759 | 105092 | 143986 | 527388 | 363680 | 341707 | 324319 | 146074 | 153419 | 154778 | **/** | 155184 | 220295 | 35223 | 88678 | 3360599 |  |
| Site243 | 203 | 208 | 111 | 98 | 988 | 1277 | 3518 | 3872 | 3322 | 3252 | 3022 | **/** | 2535 | 2219 | 531 | 1008 | 26164 |  |
| Site244 | 16 | 79 | 31 | 27 | 194 | 105 | 351 | 422 | 334 | 299 | 411 | **/** | 377 | 364 | 69 | 183 | 3264 |  |
| Site245 | 514044 | 65185 | 406768 | 239798 | 793996 | 554517 | 355149 | 432700 | 264515 | 178908 | 201735 | **/** | 211376 | 274260 | 53499 | 121710 | 4668163 |  |
| Site246 | nd | 23 | 37 | nd | 180 | 61 | 355 | 314 | 215 | 165 | 320 | **/** | 231 | 274 | 37 | 138 | 2351 |  |
| Site247 | 20 | 138 | 18 | nd | 174 | 160 | 500 | 605 | 572 | 482 | 768 | **/** | 655 | 729 | 118 | 333 | 5272 |  |
| Site248 | 412 | 776 | 291 | 235 | 995 | 1425 | 3614 | 3938 | 2501 | 2347 | 2390 | **/** | 2269 | 2374 | 413 | 1141 | 25121 |  |
| Site249 | 176 | 358 | 332 | 198 | 963 | 883 | 2088 | 2286 | 1489 | 1296 | 1651 | **/** | 1262 | 1409 | 229 | 730 | 15349 |  |
| Site250 | 482 | 681 | 1789 | 1172 | 4061 | 3136 | 5544 | 4736 | 2198 | 2397 | 2872 | **/** | 2446 | 2428 | 307 | 1154 | 35403 |  |
| Site251 | 1052 | 2555 | 2993 | 2072 | 9487 | 9369 | 17041 | 19029 | 9290 | 9388 | 9385 | **/** | 8567 | 7653 | 1327 | 3939 | 113147 |  |
| Site252 | 92 | 29 | 440 | 843 | 3032 | 1614 | 2888 | 2302 | 1439 | 1344 | 1539 | **/** | 1141 | 1294 | 428 | 554 | 18980 |  |
| Site253 | nd | 18 | 17 | nd | 39 | 18 | 82 | 99 | 97 | 79 | 174 | **/** | 126 | 123 | 24 | 72 | 968 |  |
| Site254 | 488 | 1528 | 138 | 105 | 2051 | 5624 | 12456 | 11276 | 6314 | 5396 | 3811 | **/** | 4125 | 5238 | 1332 | 3214 | 63097 |  |
| Site255 | 240 | 739 | 385 | 731 | 3276 | 3928 | 3417 | 2997 | 1913 | 1294 | 1033 | **/** | 1173 | 1566 | 315 | 949 | 23956 |  |
| Site256 | 374 | 458 | 1389 | 374 | 1788 | 2839 | 3162 | 2980 | 1657 | 1126 | 937 | **/** | 1076 | 1594 | 355 | 1003 | 21114 |  |
| Site257 | 506 | 498 | 135 | 122 | 857 | 1478 | 1681 | 1679 | 1385 | 866 | 1054 | **/** | 1099 | 1889 | 365 | 1199 | 14812 |  |
| Site258 | 3247 | 7648 | 574 | 5272 | 18287 | 12456 | 23145 | 19409 | 12244 | 12690 | 19497 | **/** | 10811 | 15880 | 4215 | 5268 | 170640 |  |
| Site259 | 6290 | 18355 | 1411 | 11230 | 65520 | 47766 | 106234 | 75119 | 39574 | 54993 | 76397 | **/** | 44430 | 33128 | 9894 | 18914 | 609255 |  |
| Site260 | 1507 | 6329 | 277 | 2052 | 13412 | 8894 | 24821 | 20316 | 13353 | 13783 | 19814 | **/** | 11021 | 20030 | 5198 | 5852 | 166659 |  |
| Site261 | 333 | 1458 | 173 | 303 | 1434 | 612 | 3325 | 2600 | 1783 | 1409 | 2812 | **/** | 1337 | 2352 | 369 | 835 | 21134 |  |
| Site262 | 141 | 176 | 42 | 53 | 329 | 170 | 200 | 612 | 447 | 355 | 749 | **/** | 354 | 437 | 85 | 214 | 4366 |  |
| Site263 | 13548 | 49107 | 2422 | 25483 | 131751 | 95814 | 173948 | 133195 | 69852 | 100791 | 145016 | **/** | 93815 | 70792 | 19711 | 33601 | 1158846 |  |
| Site264 | 5555 | 20612 | 1619 | 10160 | 46061 | 31035 | 99689 | 73654 | 34566 | 54645 | 72980 | **/** | 47714 | 29707 | 8414 | 16845 | 553257 |  |
| Site265 | 36732 | 79350 | 3815 | 64436 | 196105 | 222972 | 178295 | 170488 | 114395 | 131453 | 185182 | **/** | 130432 | 132822 | 37332 | 48406 | 1732214 |  |
| Site266 | 2018 | 1901 | 1042 | 815 | 2497 | 1579 | 7922 | 6968 | 4355 | 4196 | 7273 | **/** | 3871 | 4612 | 906 | 2513 | 52468 |  |
| Site267 | 80 | 84 | 27 | 44 | 347 | 272 | 932 | 836 | 513 | 472 | 795 | **/** | 426 | 422 | 76 | 262 | 5588 |  |
| Site268 | 65 | 50 | 27 | 40 | 331 | 121 | 581 | 496 | 421 | 309 | 554 | **/** | 350 | 462 | 71 | 259 | 4137 |  |
| Site269 | 822 | 1589 | 166 | 1010 | 4327 | 3035 | 7656 | 5478 | 3272 | 4142 | 4473 | **/** | 3157 | 2862 | 520 | 1301 | 43812 |  |
| Site270 | 712456 | 84254 | 20019 | 306195 | 812604 | 549482 | 440760 | 389831 | 214968 | 268360 | 215897 | **/** | 176463 | 223158 | 48064 | 86258 | 4548769 |  |
| Site271 | 622 | 745 | 80 | 426 | 2256 | 1256 | 3607 | 2698 | 1863 | 1909 | 2554 | **/** | 1646 | 1878 | 317 | 884 | 22742 |  |
| Site272 | 3384 | 825 | 139 | 1520 | 4891 | 2855 | 4574 | 3178 | 1786 | 2231 | 2499 | **/** | 1699 | 1716 | 293 | 743 | 32331 |  |
| Site273 | 3386 | 4655 | 437 | 3967 | 15288 | 11164 | 20374 | 14724 | 9286 | 12087 | 12354 | **/** | 9284 | 10860 | 1866 | 3696 | 133427 |  |
| Site274 | 5329 | 5947 | 738 | 9558 | 33587 | 34953 | 29443 | 20860 | 13186 | 17194 | 15347 | **/** | 11854 | 13776 | 2530 | 5087 | 219389 |  |
| Site275 | 10406 | 12286 | 1168 | 16810 | 65083 | 39437 | 55705 | 39605 | 23621 | 31805 | 26537 | **/** | 20556 | 29398 | 5174 | 9004 | 386595 |  |
| Site276 | 1401 | 1064 | 111 | 980 | 4818 | 2524 | 5483 | 3852 | 2601 | 3173 | 3852 | **/** | 2322 | 2320 | 430 | 999 | 35932 |  |
| Site277 | 303 | 179 | 283 | 261 | 2296 | 587 | 2819 | 2151 | 1193 | 873 | 1428 | **/** | 835 | 1032 | 151 | 526 | 14917 |  |
| Site278 | 144 | 218 | 145 | 90 | 769 | 426 | 1430 | 1137 | 903 | 791 | 1268 | **/** | 777 | 917 | 140 | 451 | 9606 |  |
| Site279 | 458 | 906 | 166 | 279 | 2338 | 1338 | 4303 | 3423 | 2437 | 2495 | 3271 | **/** | 2178 | 2484 | 432 | 1150 | 27658 |  |
| Site280 | 1211 | 1741 | 162 | 1608 | 8388 | 4011 | 8590 | 6546 | 3822 | 4360 | 5029 | **/** | 3345 | 3341 | 562 | 1614 | 54331 |  |
| Site281 | 1158 | 2065 | 180 | 1710 | 8906 | 4817 | 10358 | 7321 | 4500 | 5424 | 5844 | **/** | 3906 | 4536 | 1848 | 1860 | 64431 |  |
| Site282 | 2321 | 2660 | 304 | 3142 | 11575 | 7261 | 12196 | 9464 | 6190 | 7233 | 8010 | **/** | 5417 | 5401 | 929 | 2568 | 84673 |  |
| Site283 | 115 | 72 | 79 | 68 | 684 | 240 | 1229 | 981 | 763 | 569 | 1129 | **/** | 691 | 828 | 113 | 434 | 7993 |  |
| Site284 | 2611 | 4114 | 282 | 2059 | 10903 | 16325 | 16918 | 12355 | 8226 | 9950 | 10077 | **/** | 7351 | 8539 | 1639 | 3301 | 114650 |  |
| Site285 | 521 | 1128 | 103 | 482 | 2728 | 1961 | 4279 | 3381 | 2748 | 2987 | 2992 | **/** | 2366 | 3649 | 619 | 1194 | 31137 |  |
| Site286 | 1484 | 4151 | 284 | 3613 | 20811 | 12589 | 25381 | 18404 | 12418 | 15032 | 15978 | **/** | 11767 | 13719 | 2749 | 5553 | 163930 |  |
| Site287 | 365 | 682 | 103 | 510 | 3066 | 1574 | 4046 | 2973 | 2182 | 2141 | 2879 | **/** | 1884 | 2321 | 419 | 1069 | 26213 |  |
| Site288 | 488 | 1649 | 121 | 699 | 4775 | 3578 | 7372 | 5379 | 3909 | 4614 | 4590 | **/** | 3369 | 4567 | 775 | 1489 | 47375 |  |
| Site289 | 2410 | 1008 | 14998 | 6722 | 7406 | 5154 | 5749 | 8154 | 1892 | 1510 | 1162 | **/** | 745 | 581 | 117 | 380 | 57987 |  |
| Site290 | 5255 | 224 | 6513 | 2197 | 1367 | 1211 | 1076 | 1504 | 99 | 94 | 46 | **/** | 51 | 18 | nd | 14 | 19670 |  |
| Site291 | 2924 | 775 | 10229 | 6693 | 6101 | 5280 | 3768 | 5198 | 723 | 811 | 508 | **/** | 574 | 259 | 41 | 187 | 44071 |  |
| Site292 | 3689 | 9907 | 37924 | 24731 | 31874 | 45547 | 23780 | 34832 | 14520 | 16553 | 11213 | **/** | 13326 | 6486 | 1093 | 3690 | 279165 |  |
| Site293 | 2663 | 806 | 28745 | 13380 | 13911 | 12060 | 4671 | 5967 | 1075 | 852 | 631 | **/** | 377 | 314 | 43 | 194 | 85688 |  |
| Site294 | 5303 | 8147 | 50551 | 25430 | 49601 | 37913 | 19371 | 28345 | 11023 | 11997 | 7855 | **/** | 9694 | 6009 | 1080 | 2915 | 275234 |  |
| Site295 | 2711 | 625 | 13001 | 6291 | 5885 | 5097 | 2244 | 3011 | 552 | 518 | 372 | **/** | 320 | 237 | 44 | 134 | 41040 |  |
| Site296 | 149 | 166 | 773 | 247 | 786 | 401 | 1075 | 980 | 539 | 510 | 750 | **/** | 508 | 495 | 58 | 253 | 7691 |  |
| Site297 | 3839 | 444 | 16161 | 4978 | 8994 | 4232 | 1678 | 2085 | 653 | 649 | 643 | **/** | 509 | 370 | nd | 209 | 45447 |  |
| Site298 | 6293 | 338 | 16807 | 5594 | 11159 | 5430 | 2278 | 3006 | 607 | 536 | 531 | **/** | 396 | 280 | 53 | 189 | 53497 |  |
| Site299 | 2444 | 2805 | 1259 | 5404 | 22118 | 10283 | 22470 | 20686 | 12194 | 11495 | 15607 | **/** | 12994 | 12168 | 2817 | 5757 | 160501 |  |
| Site300 | 38215 | 2440 | 12335 | 6751 | 18170 | 12639 | 7314 | 12197 | 5146 | 5013 | 3461 | **/** | 4046 | 2286 | 433 | 1406 | 131852 |  |
| Site301 | 16939 | 628 | 2432 | 1466 | 4010 | 2884 | 1774 | 3013 | 1386 | 1350 | 1016 | **/** | 1115 | 579 | 117 | 391 | 39099 |  |
| Site302 | 1610 | 220 | 3846 | 278 | 828 | 540 | 570 | 774 | 418 | 296 | 740 | **/** | 545 | 503 | 151 | 246 | 11565 |  |
| Site303 | 63984 | 4046 | 25274 | 11915 | 32453 | 22623 | 18004 | 25398 | 12074 | 11359 | 10674 | **/** | 10147 | 8552 | 1433 | 4403 | 262339 |  |
| Site304 | 42315 | 2260 | 12871 | 7175 | 17758 | 12761 | 7869 | 12629 | 5252 | 5069 | 3302 | **/** | 3945 | 2342 | 411 | 1279 | 137236 |  |
| Site305 | 696 | 230 | 502 | 174 | 542 | 302 | 661 | 688 | 393 | 301 | 609 | **/** | 360 | 445 | 61 | 243 | 6206 |  |
| Site306 | 203 | 30 | 1328 | 220 | 369 | 116 | 113 | 109 | 32 | 23 | 34 | **/** | 20 | 11 | nd | 10 | 2617 |  |
| Site307 | 305 | 410 | 11754 | 289 | 3077 | 1515 | 2888 | 5180 | 1318 | 1118 | 1437 | **/** | 1016 | 1187 | nd | 629 | 32124 |  |
| Site308 | 15127 | 4276 | 65603 | 21968 | 37526 | 25522 | 31211 | 29633 | 19269 | 18556 | 20717 | **/** | 14955 | 17036 | 4381 | 6849 | 332631 |  |

nd: not detectable

/: no investigation

# Table S7 Emission (t a^-1^) of individual PAHs in China from 2000 to 2017.

|  | **Nap** | **Acy** | **Ace** | **Flo** | **Phe** | **Ant** | **Flu** | **Pyr** | **BaA** | **Chr** | **BbF** | **BkF** | **BaP** | **IcdP** | **DahA** | **BghiP** |
| --- | --- | --- | --- | --- | --- | --- | --- | --- | --- | --- | --- | --- | --- | --- | --- | --- |
| 2000 | 2.04E+04 | 1.09E+04 | 3.68E+03 | 2.98E+03 | 8.31E+03 | 2.09E+03 | 3.93E+03 | 3.07E+03 | 1.51E+03 | 7.96E+02 | 8.66E+02 | 5.86E+02 | 8.67E+02 | 4.58E+02 | 1.51E+02 | 4.85E+02 |
| 2001 | 2.17E+04 | 1.18E+04 | 3.96E+03 | 3.14E+03 | 8.77E+03 | 2.21E+03 | 4.14E+03 | 3.23E+03 | 1.59E+03 | 8.43E+02 | 9.07E+02 | 6.14E+02 | 9.05E+02 | 4.80E+02 | 1.61E+02 | 5.09E+02 |
| 2002 | 2.46E+04 | 1.36E+04 | 4.53E+03 | 3.53E+03 | 9.85E+03 | 2.48E+03 | 4.65E+03 | 3.64E+03 | 1.80E+03 | 9.57E+02 | 1.03E+03 | 6.87E+02 | 1.02E+03 | 5.41E+02 | 1.83E+02 | 5.76E+02 |
| 2003 | 2.58E+04 | 1.42E+04 | 4.67E+03 | 3.76E+03 | 1.05E+04 | 2.62E+03 | 4.97E+03 | 3.89E+03 | 1.91E+03 | 1.03E+03 | 1.13E+03 | 7.40E+02 | 1.12E+03 | 5.88E+02 | 1.95E+02 | 6.33E+02 |
| 2004 | 2.74E+04 | 1.50E+04 | 4.87E+03 | 4.03E+03 | 1.13E+04 | 2.80E+03 | 5.34E+03 | 4.18E+03 | 2.05E+03 | 1.11E+03 | 1.24E+03 | 7.99E+02 | 1.23E+03 | 6.40E+02 | 2.09E+02 | 6.96E+02 |
| 2005 | 2.90E+04 | 1.53E+04 | 4.95E+03 | 4.54E+03 | 1.25E+04 | 3.11E+03 | 6.03E+03 | 4.73E+03 | 2.28E+03 | 1.25E+03 | 1.47E+03 | 9.28E+02 | 1.45E+03 | 7.48E+02 | 2.30E+02 | 8.19E+02 |
| 2006 | 3.09E+04 | 1.63E+04 | 5.16E+03 | 4.96E+03 | 1.35E+04 | 3.35E+03 | 6.57E+03 | 5.18E+03 | 2.48E+03 | 1.38E+03 | 1.66E+03 | 1.02E+03 | 1.63E+03 | 8.34E+02 | 2.51E+02 | 9.23E+02 |
| 2007 | 3.04E+04 | 1.62E+04 | 4.92E+03 | 5.13E+03 | 1.38E+04 | 3.39E+03 | 6.80E+03 | 5.41E+03 | 2.56E+03 | 1.46E+03 | 1.82E+03 | 1.09E+03 | 1.78E+03 | 8.97E+02 | 2.60E+02 | 1.01E+03 |
| 2008 | 2.90E+04 | 1.51E+04 | 4.55E+03 | 4.86E+03 | 1.34E+04 | 3.22E+03 | 6.54E+03 | 5.15E+03 | 2.42E+03 | 1.40E+03 | 1.76E+03 | 1.04E+03 | 1.72E+03 | 8.61E+02 | 2.47E+02 | 9.75E+02 |
| 2009 | 2.86E+04 | 1.47E+04 | 4.31E+03 | 4.90E+03 | 1.35E+04 | 3.21E+03 | 6.59E+03 | 5.21E+03 | 2.43E+03 | 1.41E+03 | 1.82E+03 | 1.06E+03 | 1.78E+03 | 8.86E+02 | 2.49E+02 | 1.01E+03 |
| 2010 | 2.80E+04 | 1.43E+04 | 4.03E+03 | 5.00E+03 | 1.36E+04 | 3.22E+03 | 6.76E+03 | 5.36E+03 | 2.47E+03 | 1.47E+03 | 1.95E+03 | 1.10E+03 | 1.89E+03 | 9.34E+02 | 2.53E+02 | 1.08E+03 |
| 2011 | 2.78E+04 | 1.40E+04 | 3.80E+03 | 5.20E+03 | 1.41E+04 | 3.30E+03 | 7.04E+03 | 5.60E+03 | 2.56E+03 | 1.55E+03 | 2.11E+03 | 1.16E+03 | 2.04E+03 | 9.97E+02 | 2.62E+02 | 1.16E+03 |
| 2012 | 2.65E+04 | 1.32E+04 | 3.48E+03 | 5.09E+03 | 1.37E+04 | 3.20E+03 | 6.91E+03 | 5.51E+03 | 2.50E+03 | 1.54E+03 | 2.12E+03 | 1.15E+03 | 2.05E+03 | 9.96E+02 | 2.57E+02 | 1.17E+03 |
| 2013 | 2.56E+04 | 1.28E+04 | 3.21E+03 | 5.19E+03 | 1.37E+04 | 3.20E+03 | 7.02E+03 | 5.65E+03 | 2.54E+03 | 1.60E+03 | 2.24E+03 | 1.20E+03 | 2.15E+03 | 1.04E+03 | 2.61E+02 | 1.23E+03 |
| 2014 | 2.44E+04 | 1.20E+04 | 2.89E+03 | 5.06E+03 | 1.35E+04 | 3.10E+03 | 6.94E+03 | 5.56E+03 | 2.48E+03 | 1.61E+03 | 2.26E+03 | 1.18E+03 | 2.15E+03 | 1.04E+03 | 2.57E+02 | 1.24E+03 |
| 2015 | 2.31E+04 | 1.12E+04 | 2.66E+03 | 4.79E+03 | 1.29E+04 | 2.93E+03 | 6.67E+03 | 5.31E+03 | 2.34E+03 | 1.56E+03 | 2.18E+03 | 1.13E+03 | 2.05E+03 | 1.00E+03 | 2.45E+02 | 1.20E+03 |
| 2016 | 2.25E+04 | 1.08E+04 | 2.51E+03 | 4.74E+03 | 1.27E+04 | 2.88E+03 | 6.60E+03 | 5.26E+03 | 2.32E+03 | 1.56E+03 | 2.19E+03 | 1.13E+03 | 2.05E+03 | 1.00E+03 | 2.42E+02 | 1.20E+03 |
| 2017 | 2.15E+04 | 1.02E+04 | 2.31E+03 | 4.59E+03 | 1.23E+04 | 2.77E+03 | 6.43E+03 | 5.12E+03 | 2.24E+03 | 1.53E+03 | 2.16E+03 | 1.10E+03 | 2.01E+03 | 9.84E+02 | 2.35E+02 | 1.19E+03 |

# Table S8 Emission (t a^-1^) of individual PAHs in other countries emitted in the year corresponding to the sampling time of surface sediments (Table S6).

| **Countries** | **Emitted year** | **Nap** | **Acy** | **Ace** | **Flo** | **Phe** | **Ant** | **Flu** | **Pyr** | **BaA** | **Chr** | **BbF** | **BkF** | **BaP** | **IcdP** | **DahA** | **BghiP** |
| --- | --- | --- | --- | --- | --- | --- | --- | --- | --- | --- | --- | --- | --- | --- | --- | --- | --- |
| India | 2013 | 21547.0 | 10108.0 | 3987.0 | 2011.0 | 5897.0 | 1168.0 | 2039.0 | 1996.0 | 942.0 | 658.0 | 401.0 | 298.0 | 381.0 | 208.0 | 106.0 | 224.0 |
| Malaysia | 2011 | 1324.0 | 246.0 | 108.0 | 92.0 | 241.0 | 68.0 | 94.0 | 64.0 | 32.0 | 18.0 | 31.0 | 16.0 | 25.1 | 26.7 | 12.6 | 29.9 |
| Japan | 2014 | 1386.0 | 197.0 | 61.0 | 84.0 | 205.0 | 92.0 | 106.0 | 145.0 | 48.0 | 49.0 | 32.0 | 20.0 | 28.0 | 16.0 | 10.6 | 22.0 |
| Italy | 2011 | 765.6 | 198.0 | 66.0 | 39.6 | 120.1 | 29.0 | 48.8 | 42.2 | 23.0 | 21.5 | 57.5 | 68.4 | 79.8 | 85.2 | 108.3 | 88.5 |
| Turkey | 2012 | 3718.0 | 726.7 | 473.2 | 354.9 | 929.5 | 165.6 | 405.6 | 287.3 | 99.7 | 118.3 | 76.1 | 64.2 | 59.2 | 42.3 | 40.6 | 64.2 |
| United States | 2015 | 5502.0 | 2096.0 | 772.9 | 327.5 | 1061.1 | 196.5 | 419.2 | 340.6 | 157.2 | 110.0 | 62.9 | 48.5 | 56.3 | 47.2 | 36.7 | 47.2 |
| Nigeria | 2011 | 8502.0 | 3706.0 | 1417.0 | 959.2 | 2398.0 | 457.8 | 1199.0 | 915.6 | 316.1 | 218.0 | 163.5 | 141.7 | 174.4 | 109.0 | 43.6 | 97.0 |
| Tunisia | 2011 | 342.0 | 114.0 | 37.6 | 28.5 | 69.5 | 12.5 | 27.4 | 21.7 | 8.8 | 5.5 | 4.0 | 3.2 | 3.9 | 2.5 | 1.1 | 2.6 |
| Columbia | 2015 | 1524.6 | 492.8 | 146.3 | 83.2 | 246.4 | 147.0 | 171.5 | 75.5 | 117.2 | 110.0 | 104.9 | 47.2 | 44.1 | 64.7 | 65.9 | 62.6 |
| Iran | 2012 | 269.4 | 259.5 | 196.9 | 134.9 | 141.5 | 57.1 | 51.2 | 10.9 | 42.9 | 46.5 | 40.0 | 53.8 | 54.2 | 67.4 | 82.4 | 84.0 |
| Republic of Djibouti | 2015 | 186.0 | 143.2 | 40.9 | 15.6 | 5.0 | 10.0 | 16.0 | 14.5 | 9.7 | 4.5 | 2.8 | 15.1 | 2.6 | 14.7 | 10.4 | 16.7 |
| Norway | 2009 | 3534.0 | 1881.0 | 1195.9 | 1048.8 | 2964.0 | 866.4 | 444.6 | 1987.0 | 125.4 | 353.4 | 239.4 | 66.1 | 114.0 | 94.6 | 21.7 | 102.6 |
| Spain | 2014 | 4867.2 | 2059.2 | 1026.5 | 567.8 | 1789.3 | 330.7 | 619.3 | 508.6 | 215.3 | 235.6 | 145.1 | 106.1 | 110.8 | 59.5 | 90.5 | 129.5 |
| South Africa | 2015 | 3120.0 | 1320.0 | 658.0 | 364.0 | 1147.0 | 212.0 | 397.0 | 326.0 | 138.0 | 151.0 | 93.0 | 68.0 | 71.0 | 38.2 | 58.0 | 83.0 |
| Russia | 2013 | 2184.9 | 1070.4 | 453.8 | 196.8 | 782.9 | 126.7 | 408.4 | 422.1 | 85.4 | 315.7 | 174.4 | 79.8 | 94.0 | 82.9 | 57.5 | 72.9 |
| Hungary | 2014 | 167.4 | 63.4 | 51.0 | 42.1 | 208.7 | 17.1 | 96.9 | 60.6 | 30.9 | 28.6 | 22.0 | 36.7 | 39.4 | 71.8 | 86.9 | 74.2 |
| Cuba | 2011 | 379.5 | 197.7 | 208.9 | 17.9 | 110.0 | 83.1 | 7.1 | 9.5 | 34.7 | 27.1 | 22.3 | 30.2 | 35.7 | 47.7 | 58.5 | 52.6 |
| Pakistan | 2012 | 1781.2 | 535.2 | 383.6 | 480.0 | 1200.0 | 240.0 | 171.0 | 450.0 | 24.9 | 31.6 | 14.1 | 34.7 | 22.9 | 35.4 | 43.9 | 37.6 |
| Germany | 2012 | 2132.6 | 585.1 | 283.4 | 231.8 | 620.1 | 108.6 | 283.4 | 198.7 | 57.0 | 51.5 | 35.0 | 32.1 | 33.1 | 64.1 | 79.1 | 67.8 |
| South Korea | 2011 | 741.2 | 69.8 | 16.4 | 28.3 | 67.6 | 18.5 | 25.1 | 28.3 | 12.0 | 10.6 | 7.7 | 17.7 | 18.2 | 37.1 | 45.9 | 39.3 |
| UK | 2014 | 490.2 | 54.7 | 31.9 | 26.2 | 79.8 | 13.7 | 30.8 | 25.1 | 12.6 | 11.1 | 8.0 | 18.5 | 19.0 | 38.9 | 48.0 | 41.1 |

# Table S9 List of emission sources with technology splits.

| **Sources** | **Divisions** | **X(t)** | **China** | | | | **The United States and Canada** | | | | **Other developed countries** | | | | **Other developing countries** | | | |
| --- | --- | --- | --- | --- | --- | --- | --- | --- | --- | --- | --- | --- | --- | --- | --- | --- | --- | --- |
|  |  |  | **X_0_** | **X_f_** | **t_0_** | **s** | **X_0_** | **X_f_** | **t_0_** | **s** | **X_0_** | **X_f_** | **t_0_** | **s** | **X_0_** | **X_f_** | **t_0_** | **s** |
| ^*^Coking production | a. beehive | a/(a+b+c) | / | / | / | / | 1 | 0.002 | 1904 | 12 | 1 | 0.002 | 1894 | 17 | 1 | 0.002 | 1930 | 23 |
|  | b. mechanical-uncontrol  c. mechanical-control | b/(b+c) | 1 | 0 | 1979 | 25 | 1 | 0 | 1907 | 41 | 1 | 0 | 1915 | 55 | 1 | 0 | 1945 | 52 |
| Iron-steel industry | a. uncontrol  b. control | a/(a+b) | 1 | 0 | 1979 | 25 | 1 | 0 | 1907 | 41 | 1 | 0 | 1915 | 55 | 1 | 0 | 1945 | 52 |
| Primary Al production |  |  |  |  |  |  |  |  |  |  |  |  |  |  |  |  |  |  |
| Industry coal combustion |  |  |  |  |  |  |  |  |  |  |  |  |  |  |  |  |  |  |
| Straw burning | a. traditional stove  b. improved stove | a/(a+b) | 1 | 0 | 1988 | 21 | 1 | 0 | 1988 | 21 | 1 | 0 | 1988 | 21 | 1 | 0 | 1988 | 21 |
| Firewood burning | a. fireplace | c/(b+c) | 1 | 0 | 1985 | 23 | 1 | 0 | 1988 | 21 | 1 | 0 | 1985 | 20 | 1 | 0 | 1985 | 23 |
|  | b. traditional woodstove  c. improved woodstove | a/(a+b+c) |  |  |  |  |  |  |  |  |  |  |  |  |  |  |  |  |

^*^Beehive coke production beyond 2010 was assumed to be 0. The fractions of beehive in China have been reported annually since 1949 (China Energy Group. China Energy Databook Version 7.0; Lawrence Berkeley Lab. and Energy Res. Inst.: Berkeley, California, 2008.)

# Fig. S1 Relationships of C_Nap_ (a), C_Ace_ (b), C_Phe_ (c) and C_BaA_ (d) in sediments sampled from China with unstandardized residuals.

# Fig. S2 Relationship between mean concentrations of thirteen PAHs congeners in sediments sampled from globe and their mean EFs in fifteen emission divisions. Dashed line in plot is the linear regression.

# Fig. S3 Relationships between log*K*_ow_ and biodegradation half-life of four characteristic PAHs congeners. Dashed line in the plot is linear regressions.

# References

1. Zheng, X. Contamination and release kinetics of polycyclic aromatic hydrocarbons (PAHs) in Beijing-Hangzhou Grand Canal (Subei Section). China Mining University (2010).

2. Chiu, T. R.; Khan, M. F. & Latif, M. T. Distribution of Polycyclic Aromatic Hydrocarbons (PAHs) in Surface Sediments of Langkawi Island, Malaysia. *Sains Malays.* **47,** 871-882 (2018).

3. Pintado-Herrera, M. G.; Wang, C. & Lu, J. Distribution, mass inventories, and ecological risk assessment of legacy and emerging contaminants in sediments from the Pearl River Estuary in China. *J. Hazard. Mater.* **323,** 128-138 (2017).

4. Liu, Z. F. Source apportionment of PAHs in sediments from the Yellow River Estuary and Laizhou Bay. Ocean University of China (2008).

5. Sun, J.; Wang, G.; Chai, Y.; Zhang, G.; Li, J. & Feng, J. Distribution of polycyclic aromatic hydrocarbons (PAHs) in Henan Reach of the Yellow River, Middle China. *Ecotox. Environ. Safe.* **72,** 1614-1624 (2009).

6. Wang, J.; Lin, R. Z. & Wang, Z. S. PAHs’ distribution and source analysis on river sediments in Yanshan area of Beijing. *Journal of Yangtze University (Nature Science Edition) Sci. Eng.* **4,** 40-43 (2007).

7. Pheiffer, W.; Quinn, L. P. & Bouwman, H. Polycyclic aromatic hydrocarbons (PAHs) in sediments from a typical urban impacted river: application of a comprehensive risk assessment. *Ecotoxicology* **27,** 336-351 (2018).

8. Zhang, J. D.; Wang, Y. S. & Cheng, H. Distribution and sources of the polycyclic aromatic hydrocarbons in the sediments of the Pearl River estuary, China. *Ecotoxicology* **24,** 1643-1649 (2015).

9. Feng, J.; Zhai, M. & Sun, J. Distribution and sources of polycyclic aromatic hydrocarbons (PAHs) in sediment from the upper reach of Huaihe River, East China. *Environ. Sci. Pollut. Res.* **19,** 1097-1106 (2012).

10. Bai, Y. J.; Li, X. Q.; Liu, W. X.; Tao, S.; Wang, L. G. & Wang, J.F. Polycyclic aromatic hydrocarbon (PAH) concentrations in the dissolved, particulate, and sediment phases in the Luan River watershed, China. *J. Environ. Sci. Health. A Tox. Hazard. Subst. Environ. Eng.* **43,** 365-374 (2008).

11. Guo, W.; He, M.; Yang, Z.; Lin, C.; Quan, X. & Wang, H. Distribution of polycyclic aromatic hydrocarbons in water, suspended particulate matter and sediment from Daliao River watershed, China. *Chemosphere* **68,** 93-104 (2007).

12. Li, G.; Xia, X.; Yang, Z.; Wang, R. & Voulvoulis, N. Distribution and sources of polycyclic aromatic hydrocarbons in the middle and lower reaches of the Yellow River, China. *Environ. Pollut.* **144,** 985-993 (2006).

13. Yan, Z.; Yang, H. & Dong, H. Occurrence and ecological risk assessment of organic micropollutants in the lower reaches of the Yangtze River, China: A case study of water diversion. *Environ. Pollut.* **239,** 223-232 (2018).

14. Pozo, K.; Perra, G. & Menchi, V. Levels and spatial distribution of polycyclic aromatic hydrocarbons (PAHs) in sediments from Lenga Estuary, central Chile. *Mar. Pollut. Bull.* **62,** 1572-1576 (2011).

15. Fu, J.; Ding, Y. H. & Li, L. Polycyclic aromatic hydrocarbons and ecotoxicological characterization of sediments from the Huaihe River, China. *J. Environ. Monit.* **13,** 597-604 (2012).

16. Xiang, N.; Jiang, C. & Yang, T. Occurrence and distribution of Polycyclic aromatic hydrocarbons (PAHs) in seawater, sediments and corals from Hainan Island, China. Ecotox. *Environ. Safe.* **152,** 8-15 (2018).

17. Lu, X. X.; Zhang, S.; Chen, C.Q.; Hou, Z. & Yang, J.J. Concentration characteristics and ecological risk of persistent organic pollutants in the surface sediments of Tianjin coastal area. *Environ. Sci.* **33,** 3426-3433 (2012).

18. Hu, G. C. et al. Distribution, sources, and risk assessment of polycyclic aromatic hydrocarbons (PAHs) in surface sediments from Baiyangdian Lake. *Res. Environ. Sci.* **22,** 321-326 (2009). (in Chinese)

19. Zhuang, X. E.; Wang, X. X.; Yao, W. S.; Yang, L.; Song, X. K. & Gong, Z. B. Distribution and pollution source of polycyclic aromatic hydrocarbons in the surface sediments of Quanzhou Bay, China. *Environ. Chem.* **30,** 928-934 (2011).

20. Li, Y.; Dong, X.; Zhang, S. F. & Chen, Z. Ecological risk assessment of PAHs in surface sediments from Baihua Reservoir, Guizhou Province. *Hubei Agricultural Sciences* **52,** 1280-1283 (2013).

21. Zhao, J.; Zhou, H. D.; Fu, G.; Zhao, G. F.; Lu, J.; Wang, Y. C. & Yuan, H. Distributions, sources and ecological risk assessment of polycyclic aromatic hydrocarbons in sediments from Xidayang Reservoir, Hebei Province. *J. Lake Sci.* **23,** 701-707 (2011). (in Chinese)

22. Zhao, J.; Zhou, H. D.; Lu, J. & Wang, Y. C. Distribution, sources and ecological risk assessment of polycyclic aromatic hydrocarbons in sediments from Wangkuai Reservoir, Hebei Province. *J. Lake Sci.* **21,** 647-653 (2009). (in Chinese)

23. Hao, Z. N.; Hu, P.; Yu, Y.; Li, F. S. & Sun, H. W. Distribution and source analysis of classic persistent organic pollutants in sediments from Dagu Drainage Canal, Tianjin, China. *Journal of Agro-Environment Science* **30,** 2106-2112 (2011). (in Chinese)

24. He, J. J.; Lu, G. H.; Ding, J. N. & Xie, Z. X. Distribution, sources and risk assessment of PAHs, PBDEs and PCBs in surface sediments from northern Taihu Lake. *J. Environ. Sci. Health.* **30,** 699-702 (2013). (in Chinese)

25. Shu, W. X. & Li, S. J. Characteristics and sources of polycyclic aromatic hydrocarbons in sediments from Tianmu Lake, Jiangsu Province. *Resources and Environment in the Yangtze Basin* **18,** 27-32 (2009). (in Chinese)

26. Gu, C.; Yuan, P. Y.; Liang, L. C. & Chen, Z. Distribution, and ecological risk assessment of polycyclic aromatic hydrocarbons in surface sediments of Aha Reservoir. *Journal of Henan Agricultural Sciences* **43,** 58-61 (2014). (in Chinese)

27. Zhang, M.; Tang, F. L.; Wu, Z. X.; Chen, F.; Cheng, X. L.; Xu, J. F. & Yu, Y. Y. Pollution characteristics and ecological risk assessment of polycyclic aromatic hydrocarbons (PAHs) in surface sediments from Xin’anjiang Reservoir. *China Environmental Science* **34,** 253-258 (2014). (in Chinese)

28. Zheng, H. L. Study on distribution of polycyclic aromatic hydrocarbon contaminations in surface sediments from Haihe River in Tianjin. Tianjin University (2007).

29. Du, J.; Wu, H. H.; Yuan, M. & Guan, Y. F. Study on concentrations and sources of PAHs in the surface sediments from Pearl River. *Eco. Environ. Sci.* **19,** 766-770 (2010). (in Chinese)

30 Lu, T. T.; Lin, Q.; Ke, C. L. & Sun, Y. X. Polycyclic aromatic hydrocarbons and risk assessment in the surface sediments from Lingdingyang, Pearl River Estuary. *Journal of Fishery Sciences of China* **19,** 336-347 (2012). (in Chinese)

31. Han, F.; Guo, B. D. & Wang, Y. Y. Distribution, sources and ecological risk assessment of polycyclic aromatic hydrocarbons in surface sediments of Liaohe River. *Environmental Protection and Circular Economy* **30,** 62-66 (2010). (in Chinese)

32. Yang, X. Z.; Fan, S. X.; Tang, L. L.; Huang, H. L.; Xie, X. J. & Li, F. Characterization and change of polycyclic aromatic hydrocarbons in sediment from Waiqinhuai River. Res. *Environ. Sci.* **21,** 114-118 (2008). (in Chinese)

33. Chen, X. Y.; Huang, K.; Yan, J. P. & Kuang, J. J. Pollution Characteristics of PAHs in Sediments from the Huaihe River Basin. *Eco. Environ. Sci.* **19,** 762-765 (2010). (in Chinese)

34. Zhang, Z. H. et al. Polycyclic aromatic hydrocarbon compounds in sediments from various rivers in Tianjin. *Acta Scientiae Circumstantiae* **25**, 79-88 (2005). (in Chinese)

35. Hu, X. X.; Zhou, Y. K.; Han, Z. H.; Zhao, W. C.; Ma, Y. G. & Wang, W.H. Distribution and sources of polycyclic aromatic hydrocarbons (PAHs) in surface sediments of Huangpu River. *Environ. Chem.* **24,** 74-77 (2005).

36. Hui, Y.; Zheng, M.; Liu, Z. & Gao, L. Distribution of polycyclic aromatic hydrocarbons in sediments from Yellow River Estuary and Yangtze River Estuary, China. *J. Environ. Sci.* **21,** 1625-1631 (2009). (in Chinese)

37 Fu, J. Polycyclic aromatic hydrocarbons in surface sediments of Huaihe River and application of red mud in dye wastewater treatment. Nanjing University (2011).

38. Li, H.; Gao, H.; Zhu, C.; Li, G.; Yang, F.; Gong, Z. & Lian, J. Spatial and temporal distribution of polycyclic aromatic hydrocarbons (PAHs) in sediments of the Nansi Lake, China. *Environ. Monit. Assess.* **154,** 469-478 (2009).

39. Tian, Y.; Zheng, T. L. & Wang, X. H. Concentration, distribution and source of polycyclic aromatic hydrocarbons in surface sediments of Xiamen Western Harbor. *Oceanologia Et Limnologia Sinica* **35,** 15-20 (2004). (in Chinese)

40. Zhang, Z. L.; Hong, H. S.; Zhou, J. L.; Huang, J. & Yu, G. Fate and assessment of persistent organic pollutants in water and sediment from Minjiang River Estuary, Southeast China. *Chemosphere* **52,** 1423-1430 (2003).

41. Yu, J. Y. Concentration and distribution and sources of PCBs and PAHs in water and sediments from the source of the Qian Tang River, China. Zhejiang University of Technology (2013).

42. Mai, B. X. et al. Chlorinated and polycyclic aromatic hydrocarbons in riverine and estuarine sediments from Pearl River Delta, China. *Environ. Pollut.* **117,** 457-74 (2002).

43. Mei, W. P. Distribution and ecological risk assessment of PAHs and PCBs in the surface sediments of Dishui Lake watershed. Shanghai Ocean University (2014).

44. Wang, X. Comparative of measuring methods of persistent organic pollutants bioavailability and risk assessment in sediment in Hunhe River. Beijing Jiaotong University (2015).

45. Liu, X.; Xu, M.; Yang, Z.; Sun, T.; Cui, B.; Wang, L. & Wu, D. Sources and risk of polycyclic aromatic hydrocarbons in Baiyangdian Lake, North China. *J. Environ. Sci. Health. A Tox. Hazard. Subst. Environ. Eng.* **45,** 413-20 (2010).

46. Li, L. R.; Wang, Y. L.; Gao, J. Y.; Lin, D.; Zhang, Z. Y.; Shi, T. Y. & Wei, E. Q. Source and risk assessment of PAHs in surface sediments from rivers and lakes of China. *Environmental Monitoring in China* **29,** 92-98 (2013). (in Chinese)

47. Liu, F.; Liu, J. L.; Chen, Q. Y.; Wang, B. B. & Cao, Z. G. Pollution characteristics and ecological risk of polycyclic aromatic hydrocarbons (PAHs) in surface sediments of the southern part of the Haihe River system in China. *Chinese Science Bulletin* **58,** 1109-1116 (2013). (in Chinese)

48. Luo, S. X.; Zhu, H. W.; Zhang, X. Y. & Guan, X. M. Distribution and sources of polycyclic aromatic hydrocarbons in surface sediments from Hongfeng Lake. *Journal of Jiangxi Normal University (Nature Science)* **33,** 119-123 (2009).

49. Wang, B.; Li, Z. Y.; Fu, M. Z. & Ding, X. R. Distribution and ecological risk assessment of PAHs in surface sediments from the Yangtze Estuary and its adjecent areas. *Periodical of Ocean University of China* **37,** 83-87 (2007b).

50. Zhu, L. Z.; Cai, X. F. & Wang, J. PAHs in aquatic sediment in Hangzhou, China: Analytical methods, pollution pattern, risk assessment and sources. *J. Environ. Sci.* **17,** 748-755 (2005).

51. Liu, B. L.; Dong, D. M.; Hua, X. Y. & Li, M. Pollution characteristics and exposure risk assessment of polycyclic aromatic hydrocarbons (PAHs) in surface sediments of second Songhua River Basin. *Journal of Jilin University Science Edition* **52,** 151-157 (2014). (in Chinese)

52. Li, J.; Shang, X.; Zhao, Z.; Tanguay, R. L.; Dong, Q. & Huang, C. Polycyclic aromatic hydrocarbons in water, sediment, soil, and plants of the Aojiang River waterway in Wenzhou, China. *J. Hazard. Mater.* **173,** 75-81 (2010).

53. Liu, Z. Y. The pollution character and ecological risk assessment of Shenyang Xihe water. Shenyang University (2011).

54. Zhang, L.; Fan, C. X.; Qin, B. Q.; Zhu, G. W.; Qu, W. C. & Wang, J. J. Source of polycyclic aromatic hydrocarbons (PAHs) of the Yili River system in Lake Taihu. *Geochemistry* **32,** 124-130 (2003).

55. Jiang, B.; Zheng, H. L.; Huang, G. Q.; Ding, H.; Li, X. G.; Suo, H. T. & Li, R. Characterization and distribution of polycyclic aromatic hydrocarbon in sediments of Haihe River, Tianjin, China. *J. Environ. Sci.* **19,** 306-311 (2007). (in Chinese)

56. Lei, B.; Kang, J.; Wang, X.; Yu, Y.; Zhang, X.; Wen, Y. & Wang, Y. The levels of PAHs and aryl hydrocarbon receptor effects in sediments of Taihu Lake, China. *Environ. Sci. Pollut. Res.* **21,** 6547-6557 (2014).

57. Chen C. F.; Chen C. W.; Dong, C. D. & Kao, C. M. Assessment of toxicity of polycyclic aromatic hydrocarbons in sediments of Kaohsiung Harbor, Taiwan. *Sci. Total Environ.* **463-464,** 1174-1181 (2013).

58. Qian, X.; Liang, B.; Fu, W.; Liu, X. & Cui, B. Polycyclic aromatic hydrocarbons (PAHs) in surface sediments from the intertidal zone of Bohai Bay, Northeast China: Spatial distribution, composition, sources and ecological risk assessment. *Mar. Pollut. Bull.* **112,** 349–358 (2016).

59. Sanil Kumar, K. S.; Nair, S. M.; Salas, P. M.; Prashob Peter, K. J. & Ratheesh Kumar, C. S. Aliphatic and polycyclic aromatic hydrocarbon contamination in surface sediment of the Chitrapuzha River, South West India. *Chemistry and Ecology* **32,** 117-135 (2016).

60. Bajt, O. Aliphatic and polycyclic aromatic hydrocarbons in gulf of Trieste sediments (Northern Adriatic): potential impacts of maritime traffic. *B. Environ. Contam. Tox.* **93,** 299-305 (2014).

61. Keshavarzifard, M. et al. Baseline distributions and sources of polycyclic aromatic hydrocarbons (PAHs) in the surface sediments from the Prai and Malacca Rivers, Peninsular Malaysia. *Mar. Pollut. Bull.* **88,** 366-372 (2014).

62. Khazaali, A.; Kunzmann, A.; Bastami, K. D. & Baniamam, M. Baseline of polycyclic aromatic hydrocarbons in the surface sediment and sea cucumbers ( Holothuria leucospilota and Stichopus hermanni ) in the northern parts of Persian Gulf. *Mar. Pollut. Bull.* **110,** 539-545 (2016).

63. Shilla, D. J. Distribution and behaviour of polycyclic aromatic hydrocarbons in subtropical river estuary, Okinawa, Japan. *Chemistry and Ecology* **32,** 472-491 (2016).

64. Bergamasco, A.; Culotta, L.; De Stefano, C.; Orecchio, S.; Sammartano, S. & Barreca, S. Composition, distribution, and sources of polycyclic aromatic hydrocarbons in sediments of the Gulf of Milazzo (Mediterranean Sea, Italy). *Polycycl. Aromat. Comp.* **34,** 397-424 (2014).

65 Mirza, R.; Abedi, E.; Mohammady, M.; Faghiri, I.; Fakhri, R. & Azimi, A. Distribution and Sources of Polycyclic Aromatic Hydrocarbons (PAHs) in Surface Sediments from the Northern Part of the Persian Gulf (Hormuzgan Province). *Polycycl. Aromat. Comp.* **34,** 343–355 (2014a).

66. Rahmanpoor, S.; Ghafourian, H.; Hashtroudi, S. M. & Bastami, K. D. Distribution and sources of polycyclic aromatic hydrocarbons in surface sediments of the Hormuz strait, Persian Gulf. *Mar. Pollut. Bull.* **78**, 224-229 (2014).

67. Dudhagara, D. R.; Rajpara, R. K.; Bhatt, J. K.; Gosai, H. B.; Sachaniya, B. K. & Dave, B. P. Distribution, sources and ecological risk assessment of PAHs in historically contaminated surface sediments at Bhavnagar coast, Gujarat, India. *Environ. Pollut.* **213,** 338-346 (2016).

68. Neira, C.; Cossaboon, J.; Mendoza, G.; Hoh, E. & Levin, L. A. Occurrence and distribution of polycyclic aromatic hydrocarbons in surface sediments of San Diego Bay marinas. *Mar. Pollut. Bull.* **114,** 466-479 (2017).

69. Mehdinia, A.; Aghadadashi, V. & Fumani, N.S. Origin, distribution and toxicological potential of polycyclic aromatic hydrocarbons in surface sediments from the Bushehr coast, The Persian Gulf. *Mar. Pollut. Bull.* **90,** 334-338 (2015).

70. Aghadadashi, V.; Mehdinia, A. & Molaei, S. Origin, toxicological and narcotic potential of sedimentary PAHs and remarkable even/odd n-alkane predominance in Bushehr Peninsula, the Persian Gulf. *Mar. Pollut. Bull.* **114,** 494-504 (2017).

71. Keshavarzifard, M. & Zakaria, M. P. Polycyclic aromatic hydrocarbon (PAH) contamination of surface sediments from port Dickson, Malaysia: distribution, sources and ecological risk assessment. *Environ. Forensics* **16,** 322-332 (2015).

72. Sojinu, O. S.; Sonibare, O. O. & Zeng, E. Y. Polycyclic aromatic hydrocarbons (PAHs) in sediments from the Ologe Lagoon, Nigeria. *Energy Sources Part A-Recovery Utilization and Environmental Effects* **35,** 1524-1531 (2013).

73. Nouira, T.; Tagorti, M. A.; Budzinski, H.; Etchebert, H. & Boussetta, H. Polycyclic aromatic hydrocarbons (PAHs) in surface sediments of Monastir Bay (Tunisia, Central Mediterranean): distribution, origin and seasonal variations. *Int. J. Environ. An. Ch.* **93,** 1470-1483 (2013).

74. Burgos-Nunez, S.; Navarro-Frometa, A.; Marrugo-Negrete, J.; Enamorado-Montes, G. & Urango-Cardenas, I. Polycyclic aromatic hydrocarbons and heavy metals in the Cispata Bay, Colombia: A marine tropical ecosystem. *Mar. Pollut. Bull.* **120,** 379-386 (2017).

75. Mirza, R.; Mohammadi, M.; Faghiri, I.; Abedi, E.; Fakhri, A.; Azimi, A. & Zahed, M. A. Source identification of polycyclic aromatic hydrocarbons (PAHs) in sediment samples from the northern part of the Persian Gulf, Iran. *Environ. Monit. Assess.* **186,** 7387-7398 (2014b).

76. Mahdi Ahmed, M.; Doumenq, P.; Awaleh, M. O.; Syakti, A. D.; Asia, L. & Chiron, S. Levels and sources of heavy metals and PAHs in sediment of Djibouti-city (Republic of Djibouti). *Mar. Pollut. Bull.* **120,** 340-346 (2017).

77. Xue, R.; Chen, L.; Lu, Z.; Wang, J.; Yang, H.; Zhang, J. & Cai, M. Spatial distribution and source apportionment of PAHs in marine surface sediments of Prydz Bay, East Antarctica. *Environ. Pollut.* **219,** 528-536 (2016).

78. Gosai, H. B.; Sachaniya, B. K. & Dudhagara, D. R. Concentrations, input prediction and probabilistic biological risk assessment of polycyclic aromatic hydrocarbons (PAHs) along Gujarat coastline. *Environ. Geochem. Health.* **40,** 653-665 (2018).

79. Arp, H. P. H.; Azzolina, N. A.; Cornelissen, G. & Hawthorne, S. B. Predicting pore water EPA-34 PAH concentrations and toxicity in pyrogenic-impacted sediments using pyrene content. *Environ. Sci. Technol.* **45,** 5139-5146 (2011).

80. Shen, H. et al. Global atmospheric emissions of polycyclic aromatic hydrocarbons from 1960 to 2008 and future predictions. *Environ. Sci. Technol.* **47,** 6415−6424 (2013).

81. Li, B.; Zhou, S.; Wang, T.; Sui, X.; Jia, Z.; Li, Y.; Wang, J. & Wu, S. An improved gridded polycyclic aromatic hydrocarbon emission inventory for the lower reaches of the Yangtze River Delta region from 2001 to 2015 using satellite data. *J. Hazard. Mater.* **360,** 329–339 (2018).
